# Synthesis of β^2,2^-Amino Acids by Stereoselective Alkylation of Isoserine Derivatives Followed by Nucleophilic Ring Opening of Quaternary Sulfamidates

**DOI:** 10.1021/acs.joc.2c01034

**Published:** 2022-06-22

**Authors:** Pablo Tovillas, Claudio D. Navo, Paula Oroz, Alberto Avenoza, Francisco Corzana, María M. Zurbano, Gonzalo Jiménez-Osés, Jesús H. Busto, Jesús M. Peregrina

**Affiliations:** † Departamento de Química, Centro de Investigación en Síntesis Química, Universidad de La Rioja, 26006 Logroño, La Rioja, Spain; ‡ Center for Cooperative Research in Biosciences (CIC bioGUNE), Basque Research and Technology Alliance (BRTA), Bizkaia Technology Park, Building 800, 48160 Derio, Spain; ∥ Ikerbasque, Basque Foundation for Science, 48013 Bilbao, Spain

## Abstract

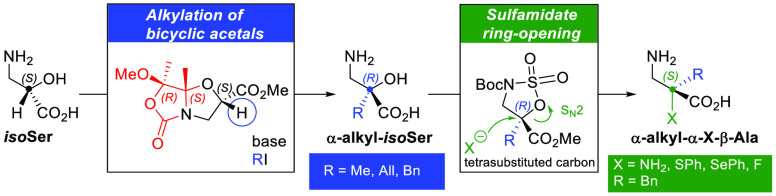

Chiral bicyclic *N,O*-acetal isoserine derivatives have been synthesized by an acid-catalyzed tandem *N*,*O*-acetalization/intramolecular transcarbamoylation reaction between conveniently protected l-isoserine and 2,2,3,3-tetramethoxybutane. The delicate balance of the steric interactions between the different functional groups on each possible diastereoisomer controls their thermodynamic stability and hence the experimental product distribution. These chiral isoserine derivatives undergo diastereoselective alkylation at the α position, proceeding with either retention or inversion of the configuration depending on the relative configuration of the stereocenters. Quantum mechanical calculations revealed that a concave-face alkylation is favored due to smaller torsional and steric interactions at the bicyclic scaffold. This synthetic methodology gives access to chiral β^2,2^-amino acids, attractive compounds bearing a quaternary stereocenter at the α position with applications in peptidomimetic and medicinal chemistry. Thus, enantiopure α-alkylisoserine derivatives were produced upon acidic hydrolysis of these alkylated scaffolds. In addition, α-benzylisoserine was readily transformed into a five-membered ring cyclic sulfamidate, which was ring opened regioselectively with representative nucleophiles to yield other types of enantiopure β^2,2^-amino acids such as α-benzyl-α-heterofunctionalized-β-alanines and α-benzylnorlanthionine derivatives.

## Introduction

**Scheme 1:**
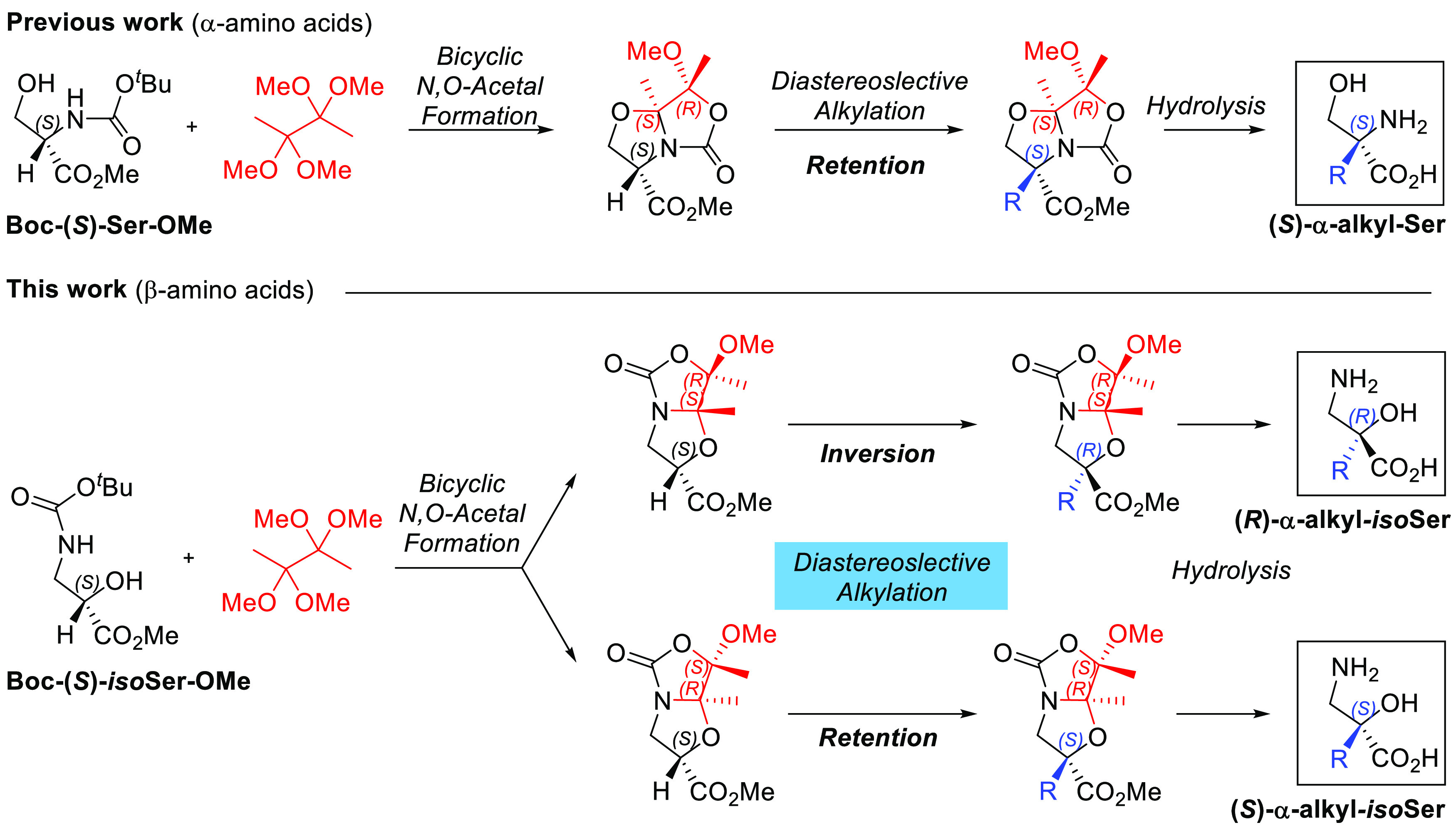
Synthesis of (*S*)-α-Alkylserine from Protected l-Ser (previous work) and (*R*)- and (*S*)-α-Alkylisoserines from Protected l-isoSer (this work) via Diastereoselective Formation of Bicyclic Acetals and Alkylation Followed by Hydrolysis

The synthesis of enantiomerically and diastereomerically pure compounds is still one of the main challenges faced by organic chemists. In this context, the use of chiral auxiliaries that are covalently bound to the substrate and subsequently removed is an effective strategy in asymmetric synthesis.^1,2^ Both chiral oxazolidinones (Evans’ oxazolidinones) and oxazolidines (*N*,*O*-acetals) have been extensively used as efficient chiral auxiliaries in asymmetric synthesis.^3−5^ By combining both strategies, we designed a chiral oxazolidine–-oxazolidinone-fused bicyclic scaffold, readily accessible from *N*-Boc-protected serine and threonine esters by diastereoselective reaction with 2,2,3,3-tetramethoxybutane (TMB) and catalytic amounts of *p*-toluenesulfonic acid (TsOH·H_2_O).^6^ These chiral derivatives displayed exceptional diastereoselectivities in the alkylation at their α position with different electrophiles. The alkylation occurs with total retention of the configuration due to the high pyramidalization of the enolate intermediate and allows the synthesis of a wide variety of chiral quaternary α-alkylserine and threonine derivatives (Scheme 1).^7,8^ Conversely, when these scaffolds were synthesized from unusual (*allo*-threonine) or unnatural (α-methylserine) amino acids, the reaction under the same conditions resulted in a complete loss of stereoselectivity toward *N*,*O*-acetals formation.^9^ As inferred from the computational studies, slight variations on the three-dimensional arrangement of the exocyclic substituents of the bicyclic compounds notably affect the thermodynamical stability of the corresponding isomers and hence determine the stereochemical outcome. With this in mind, we envisioned the use of non-natural amino acid isoserine to form the corresponding bicyclic *N*,*O*-acetals and therefore provide access to chiral quaternary β^2,2^-amino acids (Scheme 1). During the past decades, β-amino acids have become research targets in the chemical biology field,^10−13^ and thus, there has been continuous interest in the synthetic chemistry.^14−17^ However, despite the many reported methods to obtain enantioenriched β^2^- and β^3^-amino acids, only a few synthetic routes for the asymmetric synthesis of β^2,2^-amino acids have been reported,^18,19^ and the synthesis remains a challenge in organic synthesis.^20,21^ The importance of this type of amino acid is due to the existence of a quaternary stereocenter at the α position, which plays a significant role in the conformational behavior with implications in their use as peptidomimetic units and as key targets in the synthesis of Taxol analogues and β-lactams with antibiotic activity.^20,21^ In this regard, the nucleophilic ring opening of α-methylisoserine sulfamidates has been extensively used by our group to access a wide variety of β^2,2^-amino acid derivatives.^22,23^

## Results and Discussion

### Formation of Bicyclic *N*,*O*-Acetal Isoserine Derivatives

**Scheme 2:**
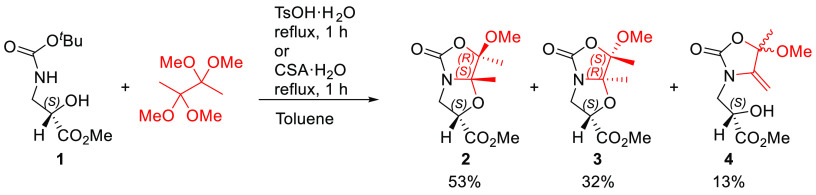
Formation of Bicyclic *N*,*O*-Acetal Isoserine Derivatives **2** and **3**

*N*-Boc-l-isoserine methyl ester (Boc-l-isoSer-OMe, **1**) was readily synthesized from commercially available l-isoserine.^24^ The reaction of Boc-l-isoSer-OMe with 2,2,3,3-tetramethoxybutane (TMB), freshly prepared from butan-2,3-dione,^25^ was then assayed in the presence of catalytic amounts of diverse acids under different reaction conditions (Scheme 2 and Supporting Information). In all cases, four different products were obtained in different ratios, corresponding to bicyclic *N*,*O*-acetal diastereomers **2** and **3**, and a mixture of two nonseparable methylene-oxazolidinone isomers **4**. This byproduct is likely formed by an in situ acid-catalyzed elimination reaction from compounds **2** and **3**, as previously observed in the formation of *allo*-threonine-derived bicyclic *N*,*O*-acetal.^9^ The optimized conditions for *N*,*O*-acetal formation required treatment with 0.2 equiv of *p*-toluenesulfonic acid (TsOH·H_2_O) or camphorsulfonic acid (CSA·H_2_O), affording isolated bicyclic compounds **2** and **3** in moderate yields (55% and 32%, respectively) and diastereomeric ratios (∼2:1). The conditions using CSA were applied to scale up the reaction starting from 13.7 mmol of **1** to obtain bicyclic systems **2**/**3** in 85% yield with a 63:37 ratio, respectively (entry 15, Table S1, Supporting Information). Because these two bicyclic compounds **2** and **3** are the key derivatives to start the synthetic routes that allow the synthesis of important β^2,2^-amino acids, we need to have them not only in large quantities but also in sufficient diastereomeric purity. Therefore, we achieved a suitable chromatographic separation that led to a high diastereomeric purity (dr 98:2) for each of them, measured by ^1^H NMR (Supporting Information).

**Figure 1:**
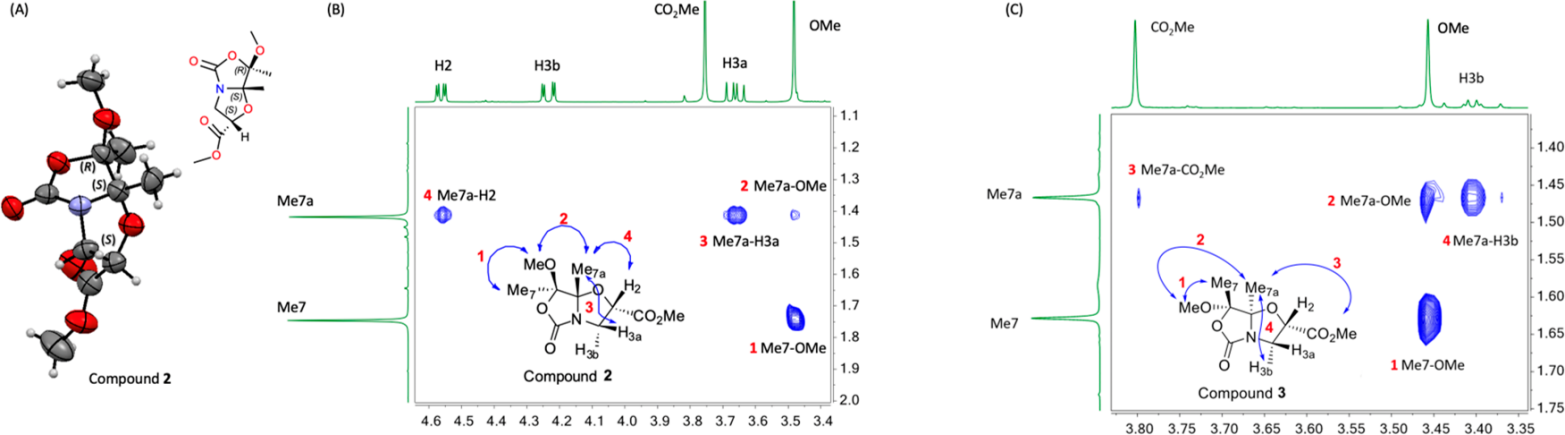
(A) ORTEP3 diagram of compound **2** obtained by X-ray diffraction analysis showing thermal ellipsoids at the 75% probability level, and 2D NOESY NMR (400 MHz) experiments for compounds **2** (B) and **3** (C) in CDCl_3_ at 298 K.

Bicyclic compounds **2** and **3** were assessed by complete NMR analysis, including 2D NOESY experiments, which allowed us to determine the absolute configuration of the new stereocenters created upon the bicyclic acetal formation. Considering that the configuration of the α-carbon of the starting isoserine derivative remains unaffected, the medium-size NOE cross-peaks observed for the bridgehead methyl group linked to the C7a carbon (Me_7a_) with H2 and H3a protons as well as with the methoxy group linked to C7 carbon (OMe) confirmed that bicyclic compound **2** exhibited a (2*S*,7*R*,7a*S*)-configuration. This finding was corroborated by X-ray analysis of a monocrystal of compound **2** obtained by slow crystallization in CH_2_Cl_2_/hexane (Supporting Information). On the other hand, the NOE cross-peaks observed for Me_7a_ with the methyl ester and the methoxy (OMe) groups confirmed the (2*S*,7*S*,7a*R*)-configuration of bicyclic compound **3** (Figure 1). Of note, the major compound **2** displays an absolute configuration equivalent to that of the major products obtained from natural amino acids (Ser and Thr), whereas the minor compound **3** exhibits reverse configurations at both C7 and C7a.

### Mechanism of the Formation of Compounds **2**–**4**

**Figure 2:**
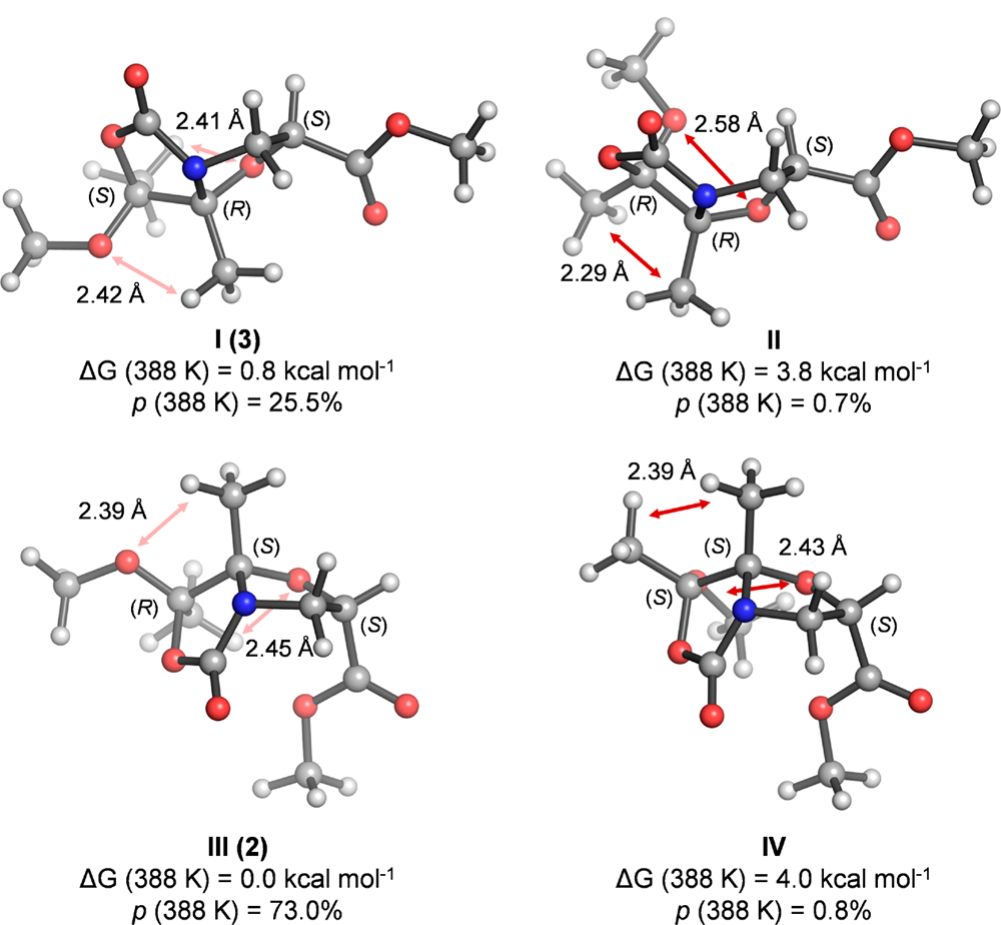
Lowest energy structures for the four possible bicyclic diastereomers (**I–IV**) obtained upon reaction of Boc-l-*iso*Ser-OMe **1** with TMB calculated with PCM(toluene)/M06-2X/6-31+G(d,p). Relative free Gibbs energies at 388 K (Δ*G*) are given in kcal mol^–1^, and relative populations (*p*) at the same temperature derived from Δ*G* are shown in parentheses. Dark and light red arrows indicate high or low steric hindrance, respectively.

The proposed mechanism for this reaction is similar to that described for the thermodynamically controlled formation of related bicyclic *N,O*-acetals^6,9^ from Boc-l-Ser-OMe: acid-catalyzed formation of the five-membered *N*,*O*-acetal followed by formation of the fused *O*,*O*-acetalic carbamate driven by *tert*-butyl group cleavage. To provide a rationale for the experimental outcome, we evaluated the thermal stability of all of the possible stereoisomers of the final bicyclic compounds using quantum mechanics calculations. Bicyclic diastereoisomers **I–IV** were optimized in implicit toluene solvent (see computation details and Supporting Information); the calculated minimum-energy structures along with their relative free Gibbs energies (Δ*G*) and populations (*p*) are depicted in Figure 2. Consistent with our previous observations and with the 2D NOESY NMR experiments, all of the calculated isoserine-derived bicyclic diastereoisomers **I–IV** show a highly pyramidalized bridgehead N atom resulting from the conformational restrains imposed by the bicyclic structure. Structures **III** and **I**, corresponding to compounds **2** and **3**, respectively, are ca. 3–4 kcal mol^–1^ more stable than structures **II** and **IV** due to the smaller steric interactions between the OMe group and the *N*,*O*-acetalic oxygen (O1) as well as between Me_7_ and Me_7a_. On the other hand, structure **III** (compound **2**) is just 0.8 kcal mol^–1^ more stable than structure **I** (compound **3**), reflecting their very similar thermostability as observed experimentally. In fact, a theoretical ∼3:1 ratio of compounds **2** and **3** was predicted from the Boltzmann distribution of all conformers and stereoisomers calculated at the experimental reaction temperature (115 °C) from their free energies, which matches the experimentally observed ratio. In accordance with their comparable energies, both disatereomers, **I** and **III**, display very similar three-dimensional arrangements, the main difference being the outward and inward presentation of the ester group with respect to the bicyclic scaffold, respectively.

The acid-catalyzed elimination process leading to enecarbamates **4** was also investigated computationally (see Supporting Information). Protonation at O1 in **I** (compound **3**) and **III** (compound **2**) was found to promote the spontaneous cleavage of the O1–C7a bond and formation of an 2-oxo-2,5-dihydrooxazol-3-ium cation; subsequent deprotonation of the methyl group adjacent to that carbocation yields the experimentally observed methylene-oxazolidinones **4**.

### Diastereoselective Alkylation of Derivatives **2** and **3**

**Scheme 3:**
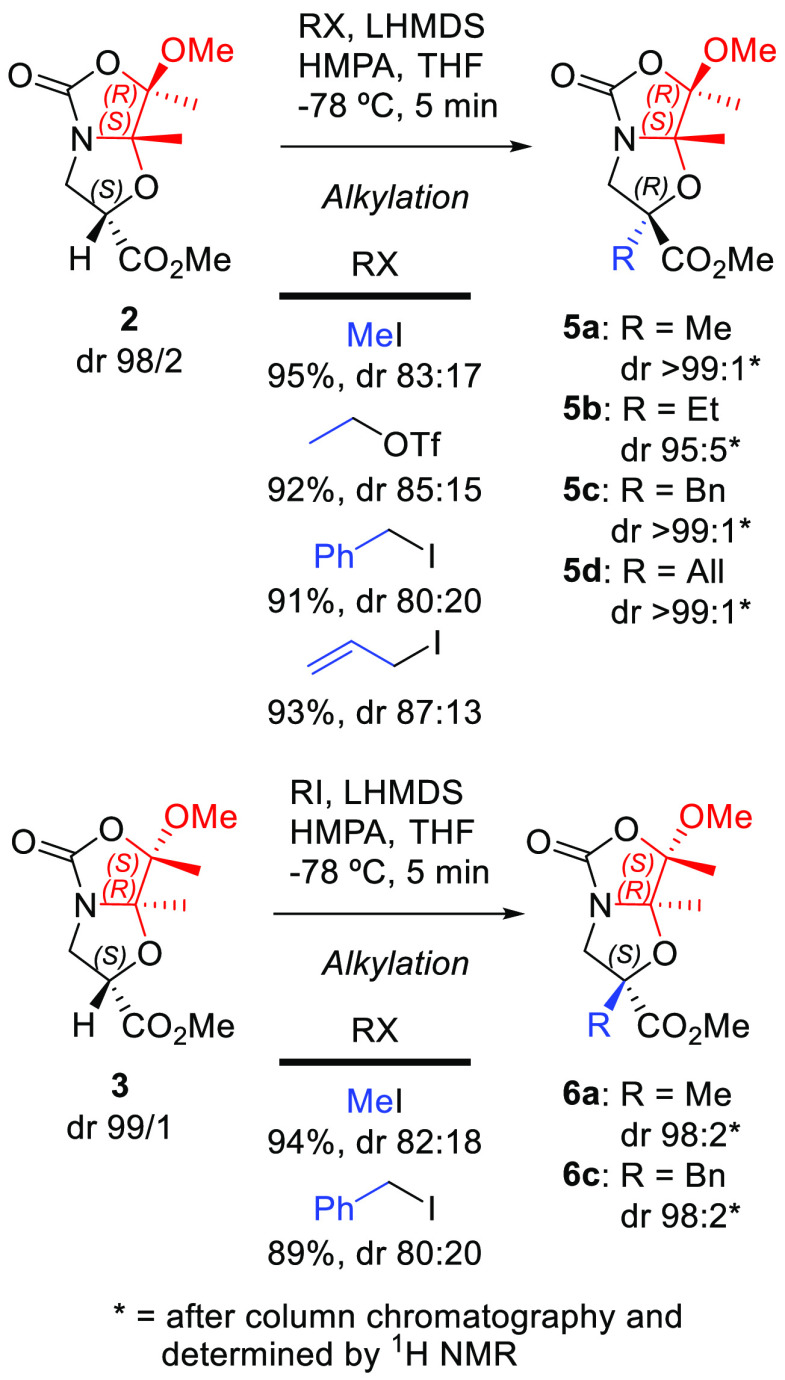
Diastereoselective Alkylation of Chiral Bicyclic Acetals **2** and **3**

Considering the good results obtained with bicyclic *N*,*O*-acetal acids derived from serine and threonine,^6^ we assayed the alkylation of both chiral isoserine-derived compounds **2** and **3** as an entry to quaternary α-alkyl-β^2^-amino acids. Optimized conditions required treatment of **2** with methyl iodide (MeI, **a**) at low temperature in the presence of lithium hexamethyldisilazide (LHMDS) as a base and hexamethylphosphoramide (HMPA) as an additive to obtain α-methylated derivative **5a** in good yield (95%) as a 83:17 mixture of diastereoisomers (Scheme 3 and Supporting Information).

**Figure 3:**
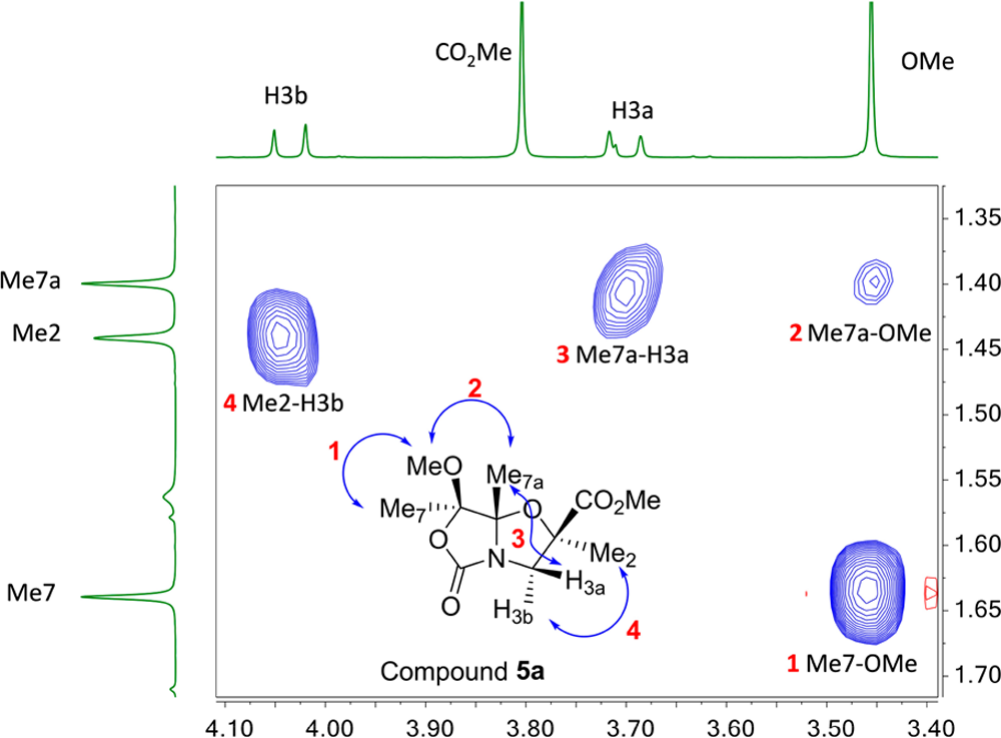
Two-dimensional NOESY NMR experiment for compounds **5a** or **6a** performed with 400 MHz equipment using CDCl_3_ as solvent at 298 K.

The absolute configuration of α-methylated compound **5a** was determined by a 2D NOESY NMR experiment (Figure 3). The medium-size NOE cross-peaks observed for the Me_7a_ group with a H3a proton and OMe along with the cross-peak Me_2_-H3b confirmed that bicyclic **5a** displays a (2*R*,7*R*,7a*S*)-configuration. Thus, methylation of compound **2** occurs with inversion of the configuration at the C2 carbon, contrary to the alkylation of bicyclic *N*,*O*-acetals derived from Ser/Thr.^6^ On the other hand, the diastereoselective alkylation of chiral building block **3** with MeI (**a**) under the same conditions led to α-methylated bicyclic compound **6a** in 5 min in a good yield (94%) and with a similar diastereoselectivity (82:18, Scheme 3). Surprisingly, methylated bicyclic compound **6a** displays the same spectroscopy data (including 2D NOESY) as bicyclic compound **5a**, but it showed the opposite sign in its specific rotation value. Therefore, a (2*S*,7*S*,7a*R*)-configuration is inferred from these data (Figure 3), indicating that, in this case, methylation occurs with retention of configuration at the C2 carbon. Hence, the major alkylation products from **2** and **3** (compounds **5a** and **6a**) are enantiomers to each other.

The scope of the diastereoselective alkylation of compound **2** was expanded using ethyl triflate (**b**), benzyl iodide (**c**), and allyl iodide (**d**) to obtain alkylated bicyclic compounds **5b** (92%, dr 85:15), **5c** (91%, dr 80:20), and **5d** (93%, dr 87:13) with good yields and diastereoselectivities. Similarly, the diastereoselective alkylation of compound **3** was also carried out with benzyl iodide (**c**), giving alkylated bicyclic compound **6c** in an 89% yield and a diastereomeric ratio of 80:20. As in the alkylation of compounds **2** and **3** with MeI to give the methylated bicyclic compounds **5a** and **6a**, respectively, the benzylated compounds **5c** and **6c** are also enantiomers. Importantly, the final alkylated compounds **5a**–**d** and **6a,c** were purified using the corresponding chromatographic columns to achieve high diastereomeric purity for all of them (Scheme 3).

### Stereochemical Outcome of the Alkylation Reaction

**Figure 4:**
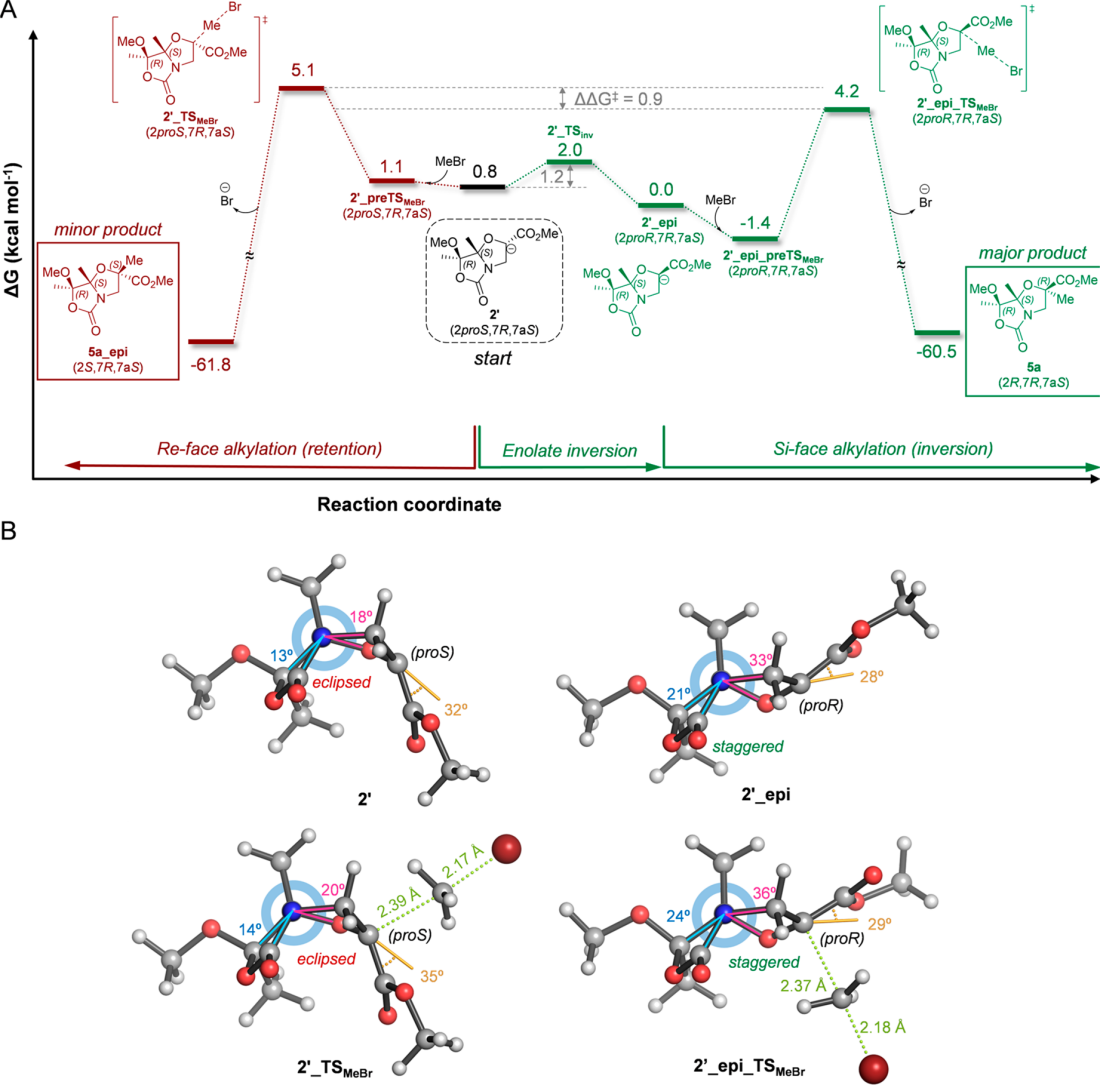
Minimum-energy pathways for the alkylation reaction of enolates **2′** (A) with bromomethane calculated with PCM(THF)/M06-2X/6-31+G(d,p). Free Gibbs energies (Δ*G*) calculated at 195 K are given in kcal mol^–1^. (B) Newman projections from N4 to C7a of the lowest energy structures for enolates **2′** and **2′_epi** and transition states **2′_TS**_**MeBr**_ and **2′_epi_TS**_**MeBr**_. Torsional strain is represented through the dihedral angles highlighted in cyan and magenta. Dihedral angles closer to 60° correspond to more staggered conformations. Pyramidalization is represented through the out-of-plane angle (in light brown) between the C2–CO_2_Me bond and the O1–C2–C3 plane. Angles close to 0° correspond to planarity.

The stereochemical course of the alkylation reactions involves an inversion of the configuration at the reacting carbon for bicyclic compound **2** and a retention of configuration at the same position for bicyclic compound **3**. This perplexing behavior observed for apparently very similar substrates was analyzed quantum mechanically (Figure 4A and Figure S3 in the Supporting Information) using bromomethane as a computationally tractable alkylating reagent in implicit THF solvent (see computation details and Supporting Information). Due to the experimental usage of HMPA, which has a superior ability to effectively solvate lithium cations,^26^ bare enolates were considered. Similarly to serine-derived *N*,*O*-acetal enolate,^6^ enolate **(2***proS***,7***R***,7a***S***)**-**2′** displays a noticeable nonplanar (pyramidalized) character (α = 32°) according to the out-of-plane angle between the C2–CO_2_Me bond and the O1–C2–C3 plane (Figure 4B). This feature usually leads to retention of configuration of the stereocenter upon alkylation, so it is reasonable to think that the highly pyramidalized enolate must invert prior to alkylation to fulfill the experimental observation. In fact, the inverted enolate **2′_epi**, which also exhibits a highly pyramidalized character (α = 28°), showed a slightly higher stability (ΔΔ*G* = −0.8 kcal mol^–1^) than **2′** due to the release of torsional strain on the bicyclic scaffold upon deprotonation. Inspection of the Newman projection along the N4–C7a bonds revealed that both rings in enolate **2′** are more eclipsed than those in **2′_epi**, as reflected by the smaller dihedral angles (Figure 4B). The activation barrier calculated for the pyramidal inversion of enolate **2′** (**2′_TS**_**inv**_) was exceedingly small (1.2 kcal mol^–1^), indicating a very fast interconversion between both enolates. The geometries of the minimum-energy transition structures (TS) for the *C*-alkylation of both enolates by the convex (**2′_TS**_**MeBr**_) and concave (**2′_epi_TS**_**MeBr**_) faces revealed a significant pyramidalization for both cases (α = 35° and 29°, respectively). Considering the rapid interconversion between reactant enolates **2′** and **2′_epi** and the irreversible formation of products, the Curtin–Hammett principle can be applied. In this context, the difference in transition state energies (ΔΔ*G*^‡^ = 0.9 kcal mol^–1^) indicates a preference for the sterically more-hindered concave (*Si*) face (**2′_epi_TS**_**MeBr**_). This difference leads to a theoretical kinetic 93:7 ratio for products **5a** and **5a_epi**, predicted from the Boltzmann distribution of all calculated alkylation TSs at the experimental reaction temperature (−78 °C), which is in good agreement with the experimental results. This preference for the apparently more hindered concave (*Si*) face can also be rationalized by the lower torsional strain around the bridgehead atoms upon enolate formation and alkylation, which is a common trend in fused five-membered bicyclic compound scaffolds.^6,27−29^

On the other hand, deprotonation of compound **3** leads to enolate **(2***proS***,7***S***,7a***R***)-3′**, which turns out to be enantiomer of enolate **(2***proR***,7***R***,7a***S*)-2′_epi (Figure S3 in the Supporting Information). Therefore, both minimum-energy pathways starting from either **3′** or **2′** are geometrically and energetically equivalent (see Figure 4A and Figure S3 in the Supporting Information), and an analogous conclusion can be drawn. Since enolate **3′** is the most stable intermediate in this case, the alkylation reaction proceeds with retention of the configuration toward compound **6a** (enantiomer of **5a**), and the epimeric compound **6a_epi** is formed as a minor product under Curtin–Hammett conditions.

### Synthesis of α-Substituted Isoserines

**Scheme 4:**
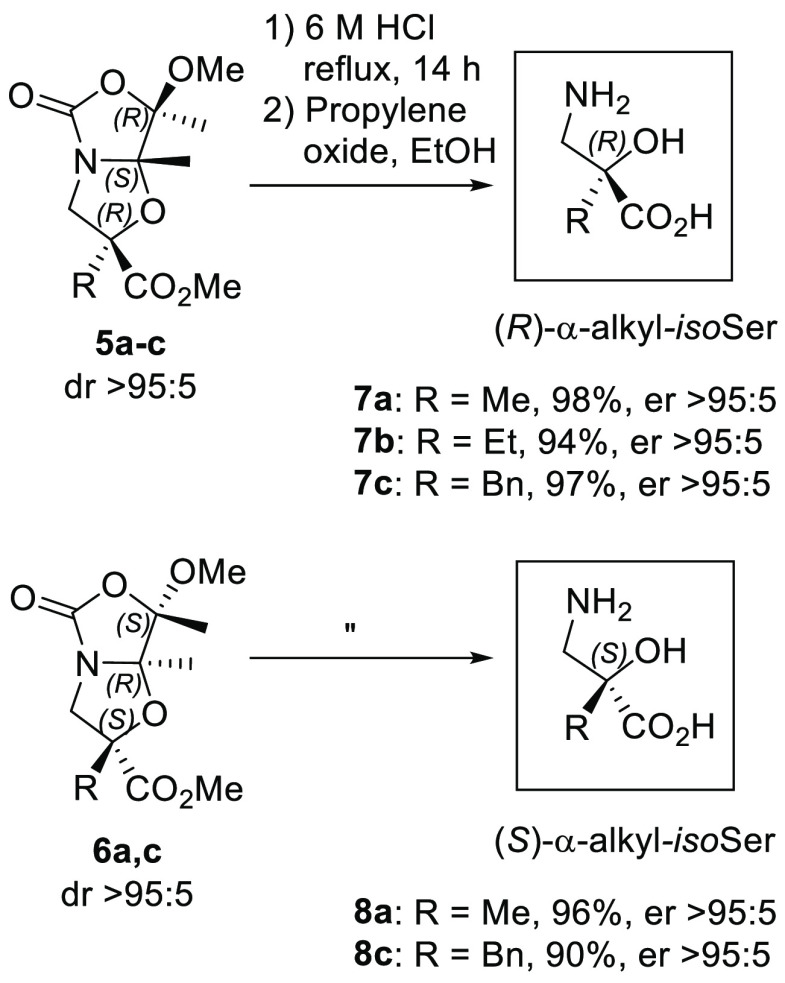
Hydrolysis of Chiral Bicyclic Acetals **5a**–**c** and **6a,c** To Obtain Enantiomerically Pure (*R*)- and (*S*)-α-Alkylisoserines

Representative α-alkylated bicyclic acetals **5a**, **5b**, **5c**, **6a**, and **6c** were subjected to acidic hydrolysis with 6 M HCl to obtain β-amino acid (*R*)-α-methylisoserine **7a**, (*R*)-α-ethylisoserine **7b**, (*R*)-α-benzylisoserine **7c**, (*S*)-α-methylisoserine **8a**, and (*S*)-α-benzylisoserine **8c**, respectively, in good yields as hydrochloride salts (Scheme 4). In some cases (**7a**–**c** and **8a,c**), the corresponding amino acid hydrochlorides were treated with propylene oxide to obtain free β-amino acids to compare their physical data with previously published data. Thus, the experimental data obtained for free (*R*)- and (*S*)-α-methylisoserine **7a** and **8a** as well as for β-amino acids **7b** and **7c** agree with those previously reported in the literature,^30^ confirming the stereochemical outcome of the diastereoselective alkylation reactions. We tried to determine the enantiomeric purity of these Cα-tetrasubstituted β-amino acids using chiral HPLC without success. Fortunately, in the case of amino acids **7c** and **8c** (enantiomers), the enantiomeric purity could be measured by NMR using a chiral lanthanide shift reagent. In particular, a samarium(III) complex with (*S*,*S*)-ethylenediamine-*N*,*N*′-disuccinate allowed the separation of the signal (doublet) corresponding to a benzylic proton, demonstrating that the enantiomeric purity was >95:5 (Figure S20 in the Supporting Information). Considering the ability of non-natural β^2,2^-amino acids,^30^ particularly α-methylisoserine, to induce folded conformations when incorporated into peptides and the few reported methods to synthesize them,^22^ the methodology reported herein represents a valuable alternative.

### α-Substituted Isoserines as Precursors of β^2,2^-Amino Acids via Sulfamidate Chemistry

**Scheme 5:**
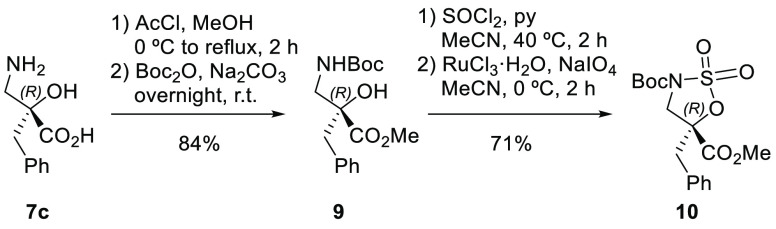
Conversion of (*R*)-α-Benzylisoserine **7c** into Cyclic Sulfamidate **10**

**Figure 5:**
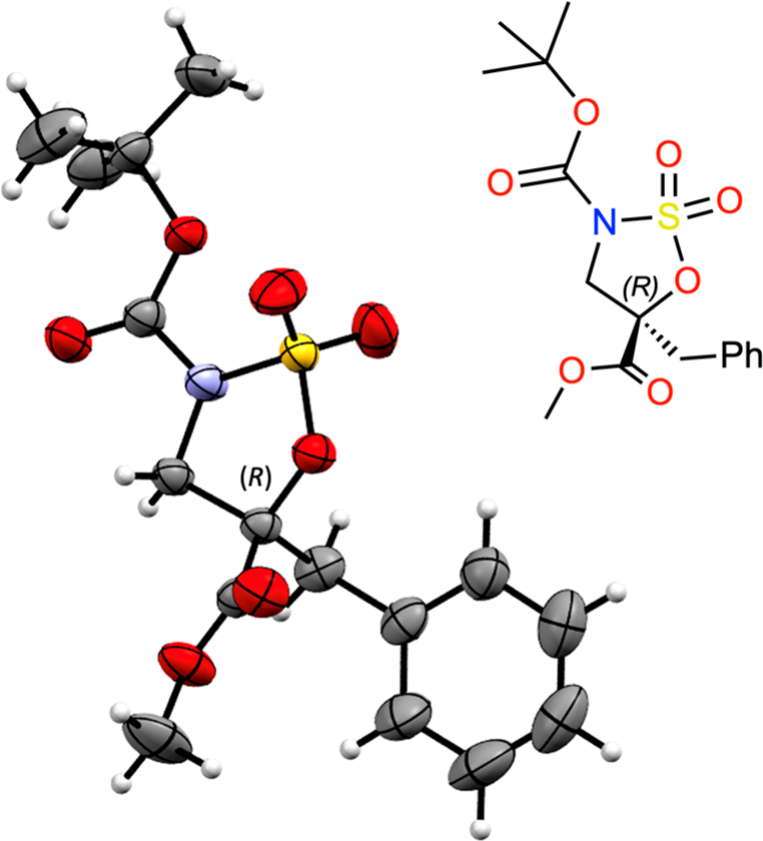
ORTEP3 diagram of sulfamidate **10** obtained by X-ray diffraction analysis showing thermal ellipsoids at the 75% probability level.

Five-membered cyclic sulfamidates are well-known valuable synthetic intermediates in organic chemistry for the regio- and stereoselective synthesis of a wide variety of chemicals.^31^ Although the synthesis and reactivity of sulfamidates have been described in detail,^32,33^ such derivatives are mostly monosubstituted or 1,2-disubstituted. In contrast, little is known about hindered sulfamidates. Our group has widely studied sulfamidates derived not only from serine,^34^ isoserine, and α-methylserine^24^ but also from α-methylisoserine.^22^ Those building blocks were subjected to further nucleophilic ring-opening reactions to obtain unnatural amino acid derivatives, glycosyl amino acids, peptides, and glycopeptides.^23,34,35^ On this basis, we envisioned to further explore the scope of a new class of hindered sulfamidates in ring-opening reactions with a variety of nucleophiles. As a representative example, the amino and acid groups of α-benzylisoserine (α-Bn-isoSer, **7c**) were conveniently protected as a *tert*-butyl carbamate and a methyl ester, respectively, to obtain compound **9**. Sulfamidate **10** was then generated using a modified protocol^22^ involving the use of thionyl chloride (SOCl_2_) and pyridine (py) in acetonitrile (MeCN) as a solvent followed by oxidation of the cyclic sulfamidite intermediate with ruthenium tetraoxide (RuO_4_), generated in situ from ruthenium trichloride monohydrate (RuCl_3_·H_2_O) and sodium periodate (NaIO_4_) (Scheme 5). The structure of sulfamidate **10** was determined by X-ray analysis (Figure 5 and Supporting Information).

**Scheme 6:**
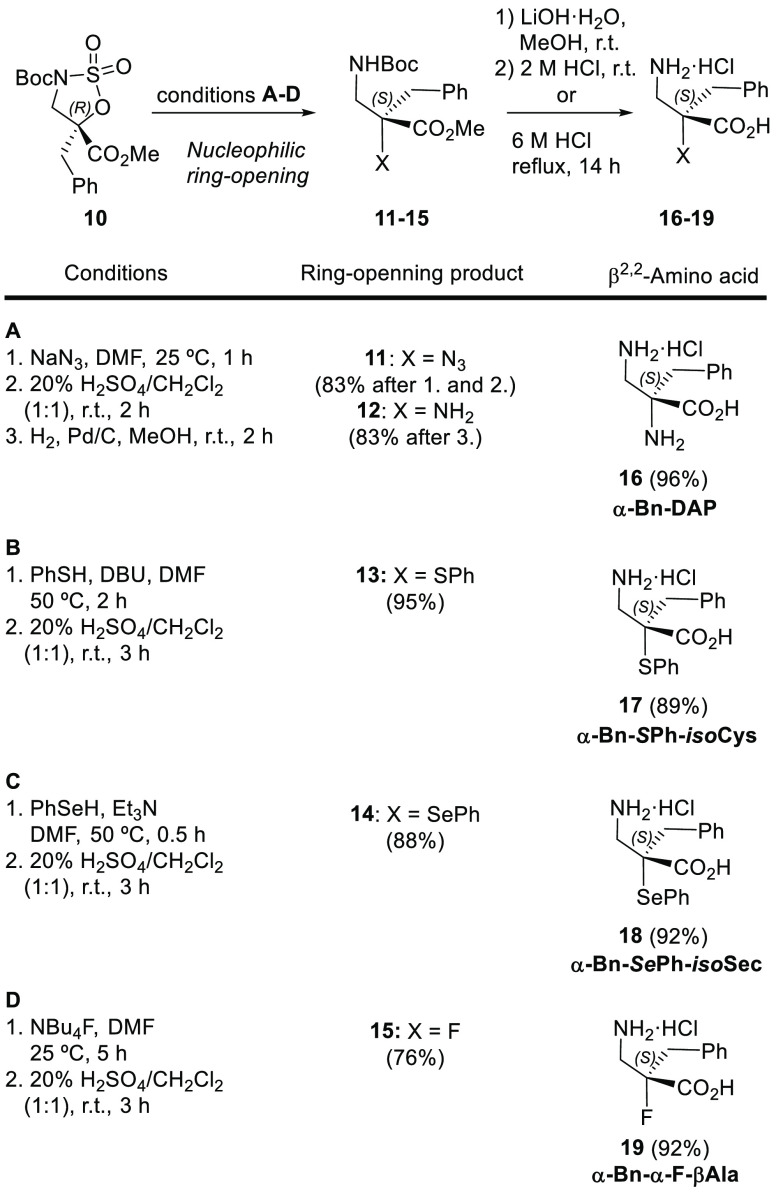
Nucleophilic Ring-Opening Reactions of Sulfamidate **10** Followed by Acid Hydrolysis To Obtain β^2,2^-Amino Acids **16–19**

Chiral sulfamidate **10** was ring opened with different nucleophiles as an entry to various α-benzyl-β^2^-amino acids (Scheme 6). Reaction with sodium azide (NaN_3_) in DMF at room temperature followed by treatment with an aqueous 20% H_2_SO_4_ solution gave protected α-azido-α-benzyl-β-amino acid derivative **11**, which was then hydrogenated to obtain *N*-Boc-protected methyl α-benzyl-2,3-diaminopropanoate **12**. Finally, acidic hydrolysis of compound **12** with 6 M HCl yielded α-benzyl-α,β-diaminopropanoic acid **16** (α-Bn-DAP) as a hydrochloride salt. Using a similar protocol, when sulfamidate **10** was treated with phenylthiolate (PhS^–^), phenylselenolate (PhSe^–^), and fluoride (F^–^) followed by addition of 20% H_2_SO_4_, the corresponding ring-opening products **13**, **14**, and **15** were readily obtained in good yields. The competitive elimination reaction frequently observed in α-methylisoserine-derived sulfamidates^22,35^ occurred only when fluoride was used as a nucleophile, and a phenyl acrylate derivative was obtained as a byproduct (21%). Ring-opening products **13**, **14**, and **15** were hydrolyzed with 6 M HCl to give β^2,2^-amino acids **17** (α-Bn-*S*Ph-isoCys), **18** (α-Bn-*Se*Ph-isoSec), and **19** (α-Bn-α-F-β-Ala), respectively, as hydrochloride salts (Scheme 6). The absolute configuration of the ring-opening products and β^2,2^-amino acids was determined by comparing their optical properties with those reported in the literature,^36−40^ demonstrating that the ring-opening reactions occur with inversion of the configuration at the chiral tetrasubstituted carbon center.

**Scheme 7:**
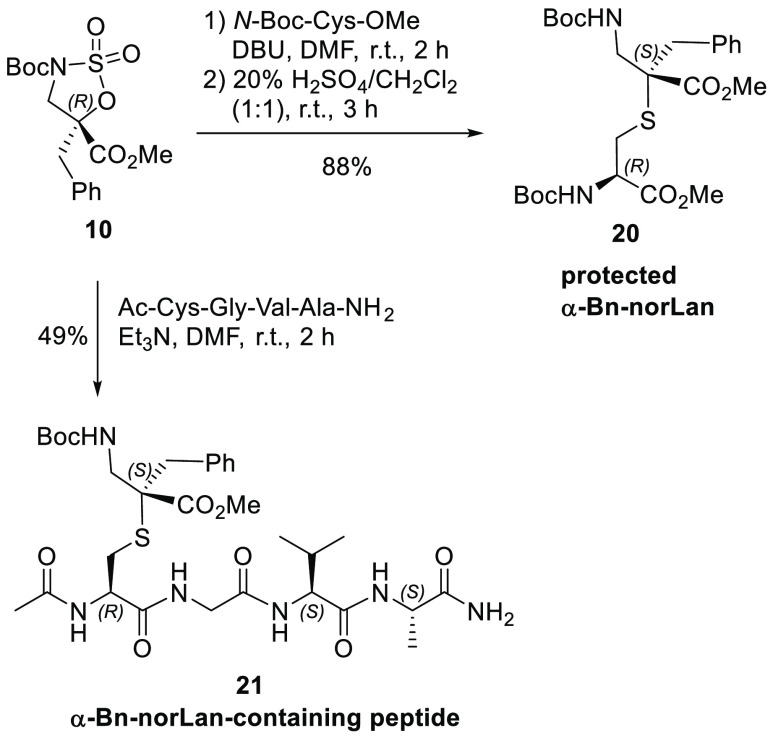
Nucleophilic Ring-Opening Reactions of Sulfamidate **10** To Obtain α-Bn-norLan **20** and α-Bn-norLan-Containing Peptide **21**

Finally, sulfamidate **10** was reacted with Boc-Cys-OMe and a Cys-containing model tetrapeptide (Ac-Cys-Gly-Val-Ala-NH_2_) to obtain protected α-benzylnorlanthionine derivative **20** and a modified tetrapeptide **21** in 88% and 49% yields, respectively (Scheme 7). The good yield obtained for α-benzylnorlanthionine derivative **20** from cyclic sulfamidate **10** derived from α-Bn-isoSer using DBU as a base is similar to other ring-opening reactions of cyclic sulfamidates such as α-Me-isoSer derivatives. On the other hand, the yield of the synthesis of tetrapeptide **21** decreases, probably due to the large size of the nucleophile used to carry out the ring-opening reaction of cyclic sulfamidate **10**. The α-benzylnorlanthionine scaffold is a mimetic of naturally occurring cross-linker bis-amino acid lanthionine, commonly found in peptidoglycans of certain *Fusobacterium* species^41^ and antimicrobial lanthipeptides.^42,43^

## Conclusion

This report covers the synthesis of a diversity of enantiomerically pure β^2,2^-amino acids, considered challenging in organic synthesis, using a straightforward synthetic methodology from l-isoserine. The strategy involves the formation and subsequent diastereoselective alkylation of chiral bicyclic *N,O*-acetals to obtain α-alkylisoserine derivatives. Remarkably, these derivatives are alkylated with either retention or inversion of configuration depending on the relative configuration of the stereocenters. The alkylation mechanism involves a highly pyramidalized chiral enolate, which can undergo a fast pyramidal inversion. Alkylation occurs preferably by the ostensibly most-hindered concave face due to the reduced torsional strain at the bicyclic scaffold in the alkylation transition structure. As a synthetic application, a variety of enantiomerically pure quaternary α-alkylisoserines were synthesized. Further, α-benzylisoserine served as a template to generate a chiral sulfamidate scaffold that was adequately prepared to undergo stereospecific nucleophilic ring-opening reactions with inversion of the configuration at the stereogenic tetrasubstituted carbon center. This sulfamidate provided easy access to four representative β^2,2^-amino acids derived from α-benzyl-β-alanine incorporating amino, phenylthio, phenylselenyl, or fluoro groups at the α position as well as the bis-amino acid α-benzylnorlanthionine and a α-benzylnorlanthionine-containing peptide.

## Experimental Section

### General and Experimental Methods

Commercial reagents were used without further purification. Analytical thin layer chromatography (TLC) was performed on Macherey-Nagel precoated aluminum sheets with a 0.20 mm thickness of silica gel 60 with fluorescent indicator UV254. TLC plates were visualized with UV light and by staining with a potassium permanganate solution (0.75 g f KMnO_4_, 5 g of K_2_CO_3_, and 0.63 mL of 10% NaOH in 100 mL of water) or a ninhydrin solution (1.5 g of ninhydrin in 100 mL of *n*-butanol and 3.0 mL of acetic acid). Column chromatography was performed on silica gel (230–400 mesh). ^1^H and ^13^C{^1^H} NMR spectra were measured with a 300 or 400 MHz spectrometer with TMS as the internal standard. Multiplicities are quoted as singlet (s), broad singlet (br s), doublet (d), doublet of doublets (dd), triplet (t), or multiplet (m). Spectra were assigned using COSY and HSQC experiments. The results of these experiments were processed with MestreNova software. High-resolution electrospray mass (ESI) spectra were recorded on a microTOF spectrometer; accurate mass measurements were achieved using sodium formate as an external reference.

### Two-Dimensional NMR Experiments

Spectra were assigned using COSY and edited-HSQC experiments (blue color for CH_2_ and red color for CH and CH_3_ groups). NOESY experiments were recorded on a 400 MHz spectrometer at 298 K. The experiments were conducted using phase-sensitive ge-2D NOESY spectra. The number of scans used was 16, and the mixing time was 800 ms.

#### Disatereoselective Formation of Bicyclic *N*,*O*-Acetals **2** and **3**

In a round-bottom flask, (*S*)-*N-*Boc-isoserine methyl ester (200 mg, 0.91 mmol) was dissolved in toluene (4 mL). Then, TMB (330 mg, 1.82 mmol) and CSA·H_2_O (46 mg, 0.18 mmol) were added. The solution was stirred under reflux in an oil bath for 1 h, until the starting materials disappeared. The reaction mixture was cooled to room temperature, diluted with diethyl ether (10 mL), and quenched with an aqueous saturated NaHCO_3_ solution (10 mL). The aqueous phase was extracted with diethyl ether (2 × 10 mL), and the organic layers were combined and dried over anhydrous Na_2_SO_4_. The solvent was removed, and the crude product was purified by column chromatography (hexane/EtOAc, 7:3) to give bicyclic *N*,*O*-acetals **2** (123 mg, 55%) and **3** (74 mg, 33%) together with compounds **4** (29 mg, 13%) as yellow oils. This synthetic procedure was scaled up to obtain *N*,*O*-acetals **2** and **3** in gram quantities after column chromatography; 1.78 g of **2** (53%, dr 98:2, *R*_f_ = 0.33) and 1.07 g of **3** (32%, dr 99:1, *R*_f_ = 0.27) using the following conditions: (*S*)-*N-*Boc-isoserine methyl ester (3.00 g, 13.7 mmol), toluene (60 mL), TMB (4.97 g, 27.4 mmol), and CSA·H_2_O (693 mg, 2.74 mmol). The solution was stirred under reflux for 1 h, until the starting materials disappeared.

##### Methyl (2*S*,7*R*,7a*S*)-7-Methoxy-7,7a-dimethyl-5-oxotetrahydro-5*H*-oxazolo[4,3-*b*]oxazole-2-carboxylate (**2**)

[α]_D_^25^ = −127.3 (*c* 1.00, CHCl_3_). HRMS (ESI) *m*/*z* [M + H]^+^ calcd for C_10_H_16_NO_6_: 246.0972. Found: 246.0973. ^1^H NMR (CDCl_3_, 400 MHz): δ (ppm) 4.56 (dd, *J* = 8.6, 2.7 Hz, 1H, H^2^), 4.23 (dd, *J* = 12.7, 2.7 Hz, 1H, H^3^), 3.75 (s, 3H, CO_2_CH_3_), 3.66 (dd, *J* = 12.7, 8.6 Hz, 1H, H^3^), 3.48 (s, 3H, OCH_3_), 1.75 (s, 3H, CH_3_), 1.42 (s, 3H, CH_3_). ^13^C{^1^H} NMR (CDCl_3_, 100 MHz): δ (ppm) 171.2 (*C*O_2_Me), 160.1 (C^5^), 107.8 (C^7^), 102.7 (C^7a^), 73.3 (C^2^), 52.6 (CO_2_*C*H_3_), 51.1 (OCH_3_), 49.6 (C^3^), 18.3 (CH_3_), 15.9 (CH_3_).

##### Methyl (2*S*,7*S*,7a*R*)-7-Methoxy-7,7a-dimethyl-5-oxotetrahydro-5*H*-oxazolo[4,3-*b*]oxazole-2-carboxylate (**3**)

[α]_D_^25^ = −27.6 (*c* 1.00, CHCl_3_). HRMS (ESI) *m*/*z* [M + H]^+^ calcd for C_10_H_16_NO_6_: 246.0972. Found: 246.0966. ^1^H NMR (CDCl_3_, 400 MHz): δ (ppm) 4.44–4.37 (m, 2H, H^2^, H^3^), 3.8 (s, 3H, CO_2_CH_3_), 3.46 (s, 3H, OCH_3_), 3.44–3.36 (m, 1H, H^3^), 1.63 (s, 3H, CH_3_), 1.47 (s, 3H, CH_3_). ^13^C{^1^H} NMR (CDCl_3_, 100 MHz): δ (ppm) 170.9 (*C*O_2_Me), 160.4 (C^5^), 107.7 (C^7^), 102.4 (C^7a^), 73.3 (C^2^), 52.7 (CO_2_*C*H_3_), 51.1 (OCH_3_), 49.0 (C^3^), 18.0 (CH_3_), 15.7 (CH_3_).

##### Methyl (2*S*)-2-Hydroxy-3-((5*S*)-5-methoxy-5-methyl-4-methylene-2-oxooxazolidin-3-yl)propanoate and Ethyl (2*S*)-2-Hydroxy-3-((5*R*)-5-methoxy-5-methyl-4-methylene-2-oxooxazolidin-3-yl)propanoate (**4**)

HRMS (ESI) *m*/*z* [M + Na]^+^ calcd for C_10_H_15_NNaO_6_: 268.0792. Found: 268.0803. ^1^H NMR (CDCl_3_, 400 MHz): δ (ppm) 4.56 (dd, 1H, *J* = 8.2, 3.1 Hz, CH_2_=C), 4.52–4.44 (m, 1H, H^2^), 4.39 (t, 1H, *J* = 3.1 Hz, CH_2_=C), 3.92–3.74 (m, 5H, CO_2_CH_3_, OH, H^3^), 3.26 (s, 3H, OCH_3_), 3.15 (dd, 1H, *J* = 10.0 Hz, *J* = 4.0 Hz, H^3^), 1.67 (s, 3H, CH_3_). ^13^C{^1^H} NMR (CDCl_3_, 100 MHz): δ (ppm) 173.0 (*C*O_2_Me), 154.7 and 154.8 (C^5^), 143.7 and 143.8 (*C*H_2_=C), 106.0 and 106.1 (C^7^), 84.6 and 84.8 (*C*H_2_=C), 67.8 (C^2^), 53.1 and 53.2 (CO_2_*C*H_3_), 50.6 and 50.7 (OCH_3_), 44.7 (C^3^), 26.0 (CH_3_).

#### General Procedure for Diastereoselective Alkylation of Bicyclic *N*,*O*-Acetals

In a Schlenk flask, the bicyclic *N*,*O*-acetal (**2** or **3**) (100 mg, 0.4 mmol) was dissolved in dry THF (10 mL) under inert atmosphere conditions. Then, HMPA (285 μL, 1.65 mmol) was added, and the mixture was cooled to −78 °C. Afterward, the alkylating agent (1.25 mmol) was charged, and LHDMS (815 μL, 0.8 mmol) was added dropwise. After 5 min, the reaction was quenched with an aqueous saturated NH_4_Cl solution (10 mL) and warmed up to room temperature. The mixture was diluted with Et_2_O (25 mL), and the aqueous phase was extracted with Et_2_O (2 × 25 mL). The combined organic phase was dried over anhydrous Na_2_SO_4_, and the solvent was evaporated. The crude reaction corresponds to a mixture of both diastereoisomers, which were subjected to purification by column chromatography to obtain the major product as a colorless oil.

##### Methyl (2*R*,7*R*,7a*S*)-7-Methoxy-2,7,7a-trimethyl-5-oxotetrahydro-5*H*-oxazolo[4,3-*b*]oxazole-2-carboxylate (**5a**)

Yield of the alkylation of compound **2** with MeI was 95% with a diastereoselectivity of 83:17. After column chromatography (CHCl_3_/toluene/EtOAc 8:1.75:0.25), compound **5a** was obtained in an 86% yield (89 mg) with a dr > 99:1. [α]_D_^25^ = −118.2 (*c* 1.00, CHCl_3_). HRMS (ESI) *m*/*z* [M + Na]^+^ calcd for C_11_H_17_NNaO_6_: 282.0948. Found: 282.0946. ^1^H NMR (CDCl_3_, 400 MHz): δ (ppm) 4.04 (d, 1H, *J* = 12.5 Hz, H^3^), 3.80 (s, 3H, CO_2_CH_3_), 3.70 (d, 1H, *J* = 12.5 Hz, H^3^), 3.46 (s, 3H, OCH_3_), 1.64 (s, 3H, CH_3_), 1.44 (s, 3H, CH_3_), 1.40 (s, 3H, CH_3_). ^13^C{^1^H} NMR (CDCl_3_, 100 MHz): δ (ppm) 173.1 (*C*O_2_Me), 160.9 (C^5^), 107.5 (C^7^), 102.6 (C^7a^), 81.5 (C^2^), 54.4 (C^3^), 52.9 (CO_2_*C*H_3_), 51.1 (OCH_3_), 25.1 (CH_3_), 17.8 (CH_3_), 16.0 (CH_3_).

##### Methyl (2*S*,7*S*,7a*R*)-7-Methoxy-2,7,7a-trimethyl-5-oxotetrahydro-5*H*-oxazolo[4,3-*b*]oxazole-2-carboxylate (**6a**)

Yield of the alkylation of compound **3** with MeI was 94% with a diastereoselectivity of 82:18. After column chromatography (CHCl_3_/toluene/EtOAc 8:1.75:0.25), compound **6a** (enantiomer of **5a**) was obtained in an 86% yield (87 mg) with a dr of 98:2. [α]_D_^25^ = +97.9 (*c* 1.00, CHCl_3_). HRMS (ESI) *m*/*z* [M + Na]^+^ calcd for C_11_H_17_NNaO_6_: 282.0944. Found: 282.0948. ^1^H NMR (CDCl_3_, 400 MHz): δ (ppm) 4.04 (d, 1H, *J* = 12.5 Hz, H^3^), 3.80 (s, 3H, CO_2_CH_3_), 3.70 (d, 1H, *J* = 12.5 Hz, H^3^), 3.46 (s, 3H, OCH_3_), 1.64 (s, 3H, CH_3_), 1.44 (s, 3H, CH_3_), 1.40 (s, 3H, CH_3_). ^13^C{^1^H} NMR (CDCl_3_, 100 MHz): δ (ppm) 173.1 (*C*O_2_Me), 160.9 (C^5^), 107.5 (C^7^), 102.6 (C^7a^), 81.5 (C^2^), 54.4 (C^3^), 52.9 (CO_2_*C*H_3_), 51.1 (OCH_3_), 25.1 (CH_3_), 17.8 (CH_3_), 16.0 (CH_3_).

##### Methyl (2*R*,7*R*,7a*S*)-2-Ethyl-7-methoxy-7,7a-dimethyl-5-oxotetrahydro-5*H*-oxazolo[4,3-*b*]oxazole-2-carboxylate (**5b**)

Yield of the alkylation of compound **2** with ethyl trifluoromethanesulfonate was 92% with a diastereoselectivity of 85:15. After column chromatography (CHCl_3_/toluene/EtOAc 8:1.75:0.25), compound **5b** was obtained in an 81% yield (22 mg, from 0.1 mmol of **2**) with a dr of 95:5. [α]_D_^25^ = −82.3 (*c* 1.00, CHCl_3_). HRMS (ESI) *m*/*z* [M + Na]^+^ calcd for C_12_H_19_NO_6_Na: 296.1105. Found: 296.1105. ^1^H NMR (CDCl_3_, 400 MHz): δ (ppm) 4.05 (d, 1H, *J* = 12.6 Hz, H^3^), 3.81 (s, 3H, CO_2_CH_3_), 3.71 (d, 1H, *J* = 9.2 Hz, H^3^), 3.46 (s, 3H, OCH_3_), 1.89–1.84 (m, 1H, C*H*_2_CH_3_), 1.70–1.58 (m, 1H, C*H*_2_CH_3_), 1.67 (s, 3H, CH_3_), 1.39 (s, 3H, CH_3_), 0.88 (t, 3H, *J* = 7.4 Hz, C*H*_3_CH_2_). ^13^C{^1^H} NMR (CDCl_3_, 100 MHz): δ (ppm) 173.0 (*C*O_2_Me), 160.7 (C^5^), 107.3 (C^7^), 102.3 (C^7a^), 85.2 (C^2^), 53.0 (C^3^), 52.7 (CO_2_*C*H_3_), 51.0 (OCH_3_), 31.7 (*C*H_2_CH_3_), 17.6 (CH_3_), 16.0 (CH_3_), 8.4 (*C*H_3_CH_2_).

##### Methyl (2*R*,7*R*,7a*S*)-2-Benzyl-7-methoxy-7,7a-dimethyl-5-oxotetrahydro-5*H*-oxazolo[4,3-*b*]oxazole-2-carboxylate (**5c**)

Yield of the alkylation of compound **2** with benzyl iodide was 91% with a diastereoselectivity of 80:20. After column chromatography (CHCl_3_/toluene/EtOAc 8:1.75:0.25), compound **5c** was obtained in a 73% yield (101 mg) with a dr > 99:1. [α]_D_^25^ = −89.7 (*c* 1.00, CHCl_3_). HRMS (ESI) *m*/*z* [M + Na]^+^ calcd for C_17_H_21_NO_6_Na: 358.1262. Found: 358.1276. ^1^H NMR (CDCl_3_, 400 MHz): δ (ppm) 7.30–7.24 (m, 3H, *Ph*CH_2_), 7.12 (dd, *J* = 7.8, 1.8 Hz, 2H, *Ph*CH_2_), 4.24 (d, 1H, *J* = 12.8 Hz, H^3^), 3.74 (s, 3H, CO_2_CH_3_), 3.67 (d, 1H, *J* = 8.9 Hz, H^3^), 3.46 (s, 3H, OCH_3_), 3.09 (d, 1H, *J* = 12.0 Hz, C*H*_2_Ph), 2.99 (d, 1H, *J* = 12.0 Hz, C*H*_2_Ph), 1.71 (s, 3H, CH_3_), 1.37 (s, 3H, CH_3_). ^13^C{^1^H} NMR (CDCl_3_, 100 MHz): δ (ppm) 172.4 (*C*O_2_Me), 160.7 (C^5^), 134.4, 129.8, 128.5, 127.4, (Ph), 107.2 (C^7^), 102.7 (C^7a^), 85.1 (C^2^), 52.7 (C^3^), 52.6 (CO_2_*C*H_3_), 51.0 (OCH_3_), 44.6 (Ph*C*H_2_), 17.5 (CH_3_), 16.0 (CH_3_).

##### Methyl (2*S*,7*S*,7a*R*)-2-Benzyl-7-methoxy-7,7a-dimethyl-5-oxotetrahydro-5*H*-oxazolo[4,3-*b*]oxazole-2-carboxylate (**6c**)

The yield of the alkylation of compound **3** with benzyl iodide was 89% with a diastereoselectivity of 80:20. After column chromatography (CHCl_3_/toluene/EtOAc 8:1.75:0.25), compound **6c** (enantiomer of **5c**) was obtained in a 68% yield (95 mg) with a dr of 98:2. [α]_D_^25^ = +78.5 (*c* 1.00, CHCl_3_). HRMS (ESI) *m*/*z* [M + Na]^+^ calcd for C_17_H_21_NO_6_Na: 358.1262. Found: 358.1263. ^1^H NMR (CDCl_3_, 400 MHz): δ (ppm) 7.30–7.24 (m, 3H, *Ph*CH_2_), 7.12 (dd, *J* = 7.8, 1.8 Hz, 2H, *Ph*CH_2_), 4.23 (d, 1H, *J* = 12.9 Hz, H^3^), 3.73 (s, 3H, CO_2_CH_3_), 3.69 (d, 1H, *J* = 8.9 Hz, H^3^), 3.46 (s, 3H, OCH_3_), 3.09 (d, 1H, *J* = 13.8 Hz, C*H*_2_Ph), 2.97 (d, 1H, *J* = 13.8 Hz, C*H*_2_Ph), 1.71 (s, 3H, CH_3_), 1.36 (s, 3H, CH_3_).

##### Methyl (2*R*,7*R*,7a*S*)-2-Allyl-7-methoxy-7,7a-dimethyl-5-oxotetrahydro-5*H*-oxazolo[4,3-*b*]oxazole-2-carboxylate (**5d**)

Yield of the alkylation of compound **2** with allyl iodide was 93% with a diastereoselectivity of 87:13. After column chromatography (CHCl_3_/toluene/EtOAc 8:1.75:0.25), compound **5d** was obtained in an 85% yield (25 mg, from 0.1 mmol of **2**) with a dr > 99:1. [α]_D_^25^ = −184.3 (*c* 1.00, CHCl_3_). HRMS (ESI) *m*/*z* [M + Na]^+^ calcd for C_13_H_19_NO_6_Na: 308.1105. Found: 308.1109. ^1^H NMR (CDCl_3_, 400 MHz): δ (ppm) 5.68–5.59 (m, 1H, CH_2_C*H*=CH_2_), 5.17–5.12 (m, 2H, CH_2_CH=C*H*_2_), 4.11 (d, 1H, *J* = 12.7 Hz, H^3^), 3.80 (s, 3H, CO_2_CH_3_), 3.68 (d, 1H, *J* = 12.7 Hz, H^3^), 3.46 (s, 3H, OCH_3_), 2.55–2.38 (m, 2H, C*H*_2_CH=CH_2_), 1.66 (s, 3H, CH_3_), 1.39 (s, 3H, CH_3_). ^13^C{^1^H} NMR (CDCl_3_, 100 MHz): δ (ppm) 172.4 (*C*O_2_Me), 160.5 (C^5^), 130.7 (CH_2_*C*H=CH_2_), 120.5 (CH_2_CH=CH_2_), 107.4 (C^7^), 102.6 (C^7a^), 84.4 (C^2^), 52.8 (CO_2_*C*H_3_), 52.6 (C^3^), 51.1 (OCH_3_), 42.7 (*C*H_2_CH=CH_2_), 17.7 (CH_3_), 16.1 (CH_3_).

#### General Procedure for Hydrolysis of Alkylated Bicyclic *N*,*O*-Acetals

The corresponding alkylated compound (0.4 mmol of **5a**–**c** or **6a,c**) was charged in a round-bottom flask with an aqueous 6 M solution of HCl (5 mL). The mixture was stirred for 14 h under reflux in an oil bath. The solvent was evaporated; the residue was dissolved in water (10 mL) and extracted with EtOAc (10 mL). The aqueous phase was evaporated, and the amino acid hydrochloride salt was obtained and treated with ethanol/propylene oxide (3:1, 4 mL) to give the free amino acid as a white solid.

##### (*R*)-3-Amino-2-hydroxy-2-methylpropanoic Acid (**7a**)

Yield 98% (46 mg), ee 96%. [α]_D_^25^ = −7.2 (*c* 1.00, H_2_O). HRMS (ESI) *m*/*z* [M + H]^+^ calcd for C_4_H_10_NO_3_: 120.0655. Found: 120.0659. ^1^H NMR (D_2_O, 400 MHz): δ (ppm) 3.36 (d, 1H, *J* = 13.2 Hz, H^β^), 3.26 (d, 1H, *J* = 13.2 Hz, H^β^), 1.54 (s, 3H, CH_3_). ^13^C{^1^H} NMR (D_2_O, 100 MHz): δ (ppm) 176.3 (*C*O_2_H), 59.5 (C^α^), 45.7 (C^β^), 22.6 (CH_3_). These data are consistent with those reported previously.^22^

##### (*S*)-3-Amino-2-hydroxy-2-methylpropanoic Acid (**8a**)

Yield 96% (44 mg), ee 94%. [α]_D_^25^ = +6.8 (*c* 1.00, H_2_O). HRMS (ESI) *m*/*z* [M + H]^+^ calcd for C_4_H_10_NO_3_: 120.0655. Found: 120.0654. ^1^H NMR (D_2_O, 400 MHz): δ (ppm) 3.36 (d, 1H, *J* = 13.2 Hz, H^β^), 3.26 (d, 1H, *J* = 13.2 Hz, H^β^), 1.54 (s, 3H, CH_3_). ^13^C{^1^H} NMR (D_2_O, 100 MHz): δ (ppm) 176.3 (*C*O_2_H), 59.5 (C^α^), 45.7 (C^β^), 22.6 (CH_3_). These data are consistent with those reported previously.^24^^22^

##### (*R*)-3-Amino-2-ethyl-2-hydroxypropanoic Acid (**7b**)

Yield 94% (46 mg), ee 86%. [α]_D_^25^ = −18.0 (*c* 1.00, H_2_O). HRMS (ESI) *m*/*z* [M + H]^+^ calcd for C_5_H_12_NO_3_: 134.0812. Found: 134.0816. ^1^H NMR (D_2_O, 400 MHz): δ (ppm) 3.30 (d, 1H, *J* = 13.3 Hz, H^β^), 3.05 (d, 1H, *J* = 13.3 Hz, H^β^), 1.79–1.70 (m, 1H, C*H*_2_CH_3_), 1.66–1.57 (m, 1H, C*H*_2_CH_3_), 0.81 (t, 3H, *J* = 7.5 Hz, C*H*_3_CH_2_). ^13^C{^1^H} NMR (D_2_O, 100 MHz): δ (ppm) 176.0 (*C*O_2_H), 75.7 (C^α^), 45.4 (C^β^), 29.8 (*C*H_2_CH_3_), 6.8 (*C*H_3_CH_2_).

##### (*R*)-3-Amino-2-benzyl-2-hydroxypropanoic Acid (**7c**)

Yield 97% (71 mg), ee 96%. [α]_D_^25^ = −24.2 (*c* 1.00, H_2_O). HRMS (ESI) *m*/*z* [M + H]^+^ calcd for C_10_H_14_NO_3_: 196.0968. Found: 196.0970. ^1^H NMR (D_2_O, 400 MHz): δ (ppm) 7.31–7.24 (m, 3H, *Ph*CH_2_), 7.18 (dd, 2H, *J* = 7.8, 1.8 Hz, *Ph*CH_2_), 3.45 (d, 1H, *J* = 13.4 Hz, H^β^), 3.13 (d, 1H, *J* = 13.4 Hz, H^β^), 3.07 (d, 1H, *J* = 13.7 Hz, C*H*_2_Ph), 2.95 (d, 1H, *J* = 13.7 Hz, C*H*_2_Ph). ^13^C{^1^H} NMR (D_2_O, 100 MHz): δ (ppm) 174.7 (*C*O_2_H), 134.1, 130.2, 128.6, 127.6, (Ph), 75.8 (C^α^), 45.2 (C^β^), 42.9 (Ph*C*H_2_).

##### (*S*)-3-Amino-2-benzyl-2-hydroxypropanoic Acid (**8c**)

Yield 90% (50 mg), ee 96%. [α]_D_^25^ = +22.4 (*c* 1.00, H_2_O). HRMS (ESI) *m*/*z* [M + H]^+^ calcd for C_10_H_14_NO_3_: 196.0968. Found: 196.0965. ^1^H NMR (D_2_O, 400 MHz): δ (ppm) 7.31–7.20 (m, 5H, *Ph*CH_2_), 3.25 (d, 1H, *J* = 13.2 Hz, H^β^), 3.01 (d, 1H, *J* = 13.5 Hz, H^β^), 3.01 (d, 1H, *J* = 13.5 Hz, C*H*_2_Ph), 2.94 (d, 1H, *J* = 13.2 Hz, C*H*_2_Ph).

##### Methyl (*R*)-2-Benzyl-3-((*tert*-butoxycarbonyl)amino)-2-hydroxy-propanoate (**9**)

In a round-bottom flask, methanol (2.4 mL) was cooled to 0 °C and acetyl chloride (0.4 mL) was added dropwise. Then, compound **7c** (100 mg, 0.48 mmol) was added, and the reaction was stirred under reflux in an oil bath until the starting materials disappeared (2 h). The solvent was removed, and methyl (*R*)-3-amino-2-benzyl-2-hydroxy-propanoate was obtained as a colorless oil without purification with column chromatography. Yield 98% (105 mg). [α]_D_^25^ = −15.2 (*c* 1.00, H_2_O). HRMS (ESI) *m*/*z* [M + H]^+^ calcd for C_11_H_16_NO_3_: 210.1125. Found: 210.1126. ^1^H NMR (CDCl_3_, 400 MHz): δ (ppm) 7.30–7.23 (m, 3H, *Ph*CH_2_), 7.20–7.17 (m, 2H, *Ph*CH_2_), 3.73 (s, 3H, CO_2_Me), 3.36 (d, 1H, *J* = 13.1 Hz, H^β^), 3.09–3.03 (m, 3H, C*H*_2_Ph, H^β^). ^13^C{^1^H} NMR (CDCl_3_, 100 MHz): δ (ppm) 172.5 (*C*O_2_Me), 134.3, 129.9, 128.0, 127.0 (Ph), 75.4 (C^α^), 52.0 (OMe), 44.8 (C^β^), 43.4 (Ph*C*H_2_). Methyl (*R*)-3-amino-2-benzyl-2-hydroxy-propanoate (100 mg, 0.48 mmol) was dissolved in THF (32 mL), and N_2_CO_3_·10H_2_O (279 mg, 1.05 mmol) and Boc_2_O (136 mg, 0.62 mmol) were added to the solution. Then, water (8 mL) was added, and the mixture was stirred overnight. After this time, the solvent was removed and the aqueous phase was extracted with ethyl acetate (3 × 10 mL). The organic phases were combined and dried over anhydrous Na_2_SO_4_, and the solvent was evaporated. The crude product was purified by column chromatography (hexane/EtOAc 9:1), giving the final product **9** as a colorless oil. Yield 84% (124 mg). [α]_D_^25^ = −57.7 (*c* 1.00, CHCl_3_). HRMS (ESI) *m*/*z* [M + Na]^+^ calcd for C_16_H_23_NO_5_Na: 332.1468. Found: 332.1471. ^1^H NMR (CDCl_3_, 400 MHz): δ (ppm) 7.19–7.15 (m, 3H, *Ph*CH_2_), 7.11–7.08 (m, 2H, *J* = 7.9, 1.8 Hz, *Ph*CH_2_), 4.70 (br s, 1H, NH), 3.66 (s, 3H, CO_2_Me), 3.73–3.65 (m, 1H, H^β^), 3.38 (br s, 1H, OH), 3.20 (dd, 1H, *J* = 13.8, 4.5 Hz, H^β^), 2.98 (d, 1H, *J* = 13.6 Hz, C*H*_2_Ph), 2.84 (d, 1H, *J* = 13.6 Hz, C*H*_2_Ph), 1.35 (s, 9H, Boc). ^13^C{^1^H} NMR (CDCl_3_, 100 MHz): δ (ppm) 174.7 (*C*O_2_Me), 156.0 (*C*O_2_C(CH_3_)_3_), 135.1, 130.0, 128.3, 127.1 (Ph), 79.7 (C^α^), 78.3 (CO_2_*C*(*C*H_3_)_3_), 52.8 (OMe), 47.7 (C^β^), 42.5 (Ph*C*H_2_), 28.3 (C(*C*H_3_)_3_).

##### 3-(*tert*-Butyl) 5-Methyl (*R*)-5-Benzyl-1,2,3-oxathiazolidine-3,5-dicarboxylate 2,2-dioxide (**10**)

To a solution of thionyl chloride (30.0 μL, 0.42 mmol) in dry acetonitrile (5 mL) was added another solution of compound **9** (100 mg, 0.32 mmol) in dry acetonitrile (2 mL) dropwise at −40 °C. The reaction was stirred for 45 min, and then pyridine (130 μL, 1.62 mmol) was added. The mixture was stirred until the starting materials disappeared (2 h). At that time, the reaction was quenched with water, warmed to room temperature, and extracted with ethyl acetate (3 × 10 mL). The organic phases were combined and dried over anhydrous Na_2_SO_4_. The solvent was removed under vacuum; the product was dissolved in acetonitrile (5 mL) and cooled to 0 °C. Ruthenium(III) chloride hydrate (2 mg, 0.005 mmol), sodium periodate (104 mg, 0.49 mmol), and water (5 mL) were added. The mixture was stirred for 2 h at 0 °C, and the aqueous phase was extracted with Et_2_O (3 × 5 mL). The organic phases were combined, washed with a saturated solution of NaHCO_3_, and dried over anhydrous Na_2_SO_4_. The solvent was evaporated, and the crude product was purified by column chromatography (hexane/EtOAc, 8:2), giving the final product **10** as a colorless oil. Yield 71% (85 mg). [α]_D_^25^ = −19.8 (*c* 1.00, CHCl_3_). HRMS (ESI) *m*/*z* [M + Na]^+^ calcd for C_16_H_21_NO_7_SNa: 394.0931. Found: 394.0930. ^1^H NMR (CDCl_3_, 400 MHz): δ (ppm) 7.28–7.22 (m, 3H, *Ph*CH_2_), 7.12 (dd, 2H, *J* = 7.3, 2.3 Hz, *Ph*CH_2_), 4.33 (d, 1H, *J* = 10.5 Hz, H^β^), 3.91 (d, 1H, *J* = 10.5 Hz, H^β^), 3.69 (s, 3H, CO_2_Me), 3.30 (m, 2H, C*H*_2_Ph), 1.46 (s, 9H, Boc). ^13^C{^1^H} NMR (CDCl_3_, 100 MHz): δ (ppm) 168.0 (*C*O_2_Me), 148.2 (*C*O_2_(CH_3_)_3_), 131.7, 130.1, 128.9, 128.2 (Ph), 86.1 (C^α^), 85.3 (CO_2_*C*(*C*H_3_)_3_), 53.6 (OMe), 51.2 (C^β^), 42.2 (Ph*C*H_2_), 27.9 (C(*C*H_3_)_3_).

##### Methyl (*S*)-2-Azido-2-benzyl-3-((*tert*-butoxycarbonyl)amino)propanoate (**11**)

Cyclic sulfamidate **10** (40 mg, 0.11 mmol) and sodium azide (32 mg, 0.49 mmol) were dissolved in DMF (4 mL) and stirred at 25 °C for 1 h until the starting material disappeared. After that time, the solvent was evaporated, and the residue was dissolved in a mixture of 20% aq. H_2_SO_4_ and CH_2_Cl_2_ (1:1, 5 mL). This mixture was stirred for 2 h at room temperature, and the aqueous phase was extracted with CH_2_Cl_2_ (3 × 5 mL). The organic phases were combined and dried over anhydrous Na_2_SO_4_. The solvent was evaporated, and the crude product was purified by column chromatography (hexane/EtOAc, 7:3) to obtain the final product **11** as a colorless oil (30 mg, 83%). [α]_D_^25^ = +9.9 (*c* 1.00, CHCl_3_) HRMS (ESI) *m*/*z* [M + Na]^+^ calcd for C_16_H_22_N_4_O_4_Na: 357.1533. Found: 357.1532. ^1^H NMR (CDCl_3_, 400 MHz): δ (ppm) 7.29–7.17 (m, 3H, *Ph*CH_2_), 7.12 (dd, 2H, *J* = 7.2, 1.9, *Ph*CH_2_), 4.75 (br s, 1H, NH), 3.71 (s, 3H, CO_2_Me), 3.55 (dd, 1H, *J* = 14.0, 6.7 Hz, H^β^), 3.29 (dd, 1H, *J* = 14.0, 6.4 Hz, H^β^), 3.12 (d, 1H, *J* = 13.7 Hz, C*H*_2_Ph), 2.94 (d, 1H, *J* = 13.8 Hz, C*H*_2_Ph), 1.37 (s, 9H, Boc). ^13^C{^1^H} NMR (CDCl_3_, 100 MHz): δ (ppm) 170.9 (*C*O_2_Me), 155.6 (*C*O_2_C(CH_3_)_3_), 134.0, 130.1, 128.6, 127.6 (Ph), 80.0 (C^α^), 70.8 (CO_2_*C*(*C*H_3_)_3_), 52.9 (OMe), 46.2 (C^β^), 41.1 (Ph*C*H_2_), 28.3 (C(*C*H_3_)_3_).

##### Methyl (*S*)-2-Amino-2-benzyl-3-((*tert*-butoxycarbonyl)amino)propanoate (**12**)

Into a Schlenk reactor, palladium on carbon (3 mg, 10% mass) was suspended in methanol (4 mL) and prehydrogenated for 10 min. Then, compound **11** was dissolved in methanol (4 mL) and added to the catalyst in one portion (30 mg, 0.10 mmol). The reaction was stirred at room temperature for 2 h until the starting product disappeared. The mixture was filtered through diatomaceous earth and concentrated in vacuo, and the crude product was purified by column chromatography (hexane/EtOAc, 3:7) to obtain the final product **12** as a colorless oil (23 mg, 83%). [α]_D_^25^ = +9.3 (*c* 1.00, CHCl_3_). HRMS (ESI) *m*/*z* [M + H]^+^ calcd for C_16_H_25_N_2_O_4_: 309.1809. Found: 309.1818. ^1^H NMR (CDCl_3_, 400 MHz): δ (ppm) 7.25–7.16 (m, 3H, *Ph*CH_2_), 7.10–7.02 (m, 2H, *Ph*CH_2_), 4.85 (br s, 1H, N*H*Boc), 3.65 (s, 3H, CO_2_Me), 3.46 (dd, 1H, *J* = 13.6, 6.1 Hz, H^β^), 3.26 (dd, 1H, *J* = 13.6, 6.5 Hz, H^β^), 3.08 (d, 1H, *J* = 13.4 Hz, C*H*_2_Ph), 2.71 (d, 1H, *J* = 13.4 Hz, C*H*_2_Ph), 1.60 (br s, 2H, NH_2_), 1.37 (s, 9H, Boc). ^13^C{^1^H} NMR (CDCl_3_, 100 MHz): δ (ppm) 175.4 (*C*O_2_Me), 156.0 (*C*(CH_3_)_3_), 79.6 (C^α^), 135.5, 129.9, 128.5, 127.2 (Ph), 62.5 (CO_2_*C*(*C*H_3_)_3_), 52.3 (OMe), 48.4 (C^β^), 43.2 (Ph*C*H_2_), 28.4 (C(*C*H_3_)_3_).

##### Methyl (*S*)-2-Benzyl-3-((*tert*-butoxycarbonyl)amino)-2-(phenylthio)propanoate (**13**)

Cyclic sulfamidate **10** (38 mg, 0.116 mmol), DBU (18 μL, 0.122 mmol), and thiophenol (13 μL, 0.128 mmol) were dissolved in DMF (4 mL) and stirred at 50 °C in an oil bath until the starting materials disappeared (2 h). Then, the solvent was removed under vacuum; the residue was dissolved in a mixture of CH_2_Cl_2_ and 20% aq. H_2_SO_4_ (1:1, 5 mL) and stirred for 3 h. After that time, the aqueous phase was extracted with CH_2_Cl_2_ (3 × 5 mL). The organic phases were combined and dried over anhydrous Na_2_SO_4_. The solvent was removed, and the crude product was purified by column chromatography (hexane/EtOAc, 9:1) to obtain the final product **13** as a colorless oil (39 mg, 95%). [α]_D_^25^ = −30.1 (*c* 1.00, CHCl_3_). HRMS (ESI) *m*/*z* [M + H]^+^ calcd for C_22_H_28_NO_4_S: 402.1733. Found: 402.1726. ^1^H NMR (CDCl_3_, 400 MHz): δ (ppm) 7.46–7.08 (m, 10H, *Ph*CH_2_, *Ph*S), 5.07 (br s, 1H, NH), 3.55 (s, 3H, CO_2_Me), 3.45–3.39 (m, 1H, H^β^), 3.27–3.21 (m, 2H, C*H*_2_Ph, H^β^), 2.97 (d, 1H, *J* = 13.7 Hz, C*H*_2_Ph), 1.41 (s, 9H, Boc). ^13^C{^1^H} NMR (CDCl_3_, 100 MHz): δ (ppm) 171.8 (*C*O_2_Me), 155.7 (*C*O_2_C(CH_3_)_3_), 137.0, 135.3, 130.2, 129.9, 129.1, 129.0, 128.4, 127.6, 127.3, 127.2, (Ph), 79.5 (C^α^), 60.0 (CO_2_*C*(*C*H_3_)_3_), 52.1 (OMe), 42.6 (C^β^), 40.3 (Ph*C*H_2_), 28.4 (C(*C*H_3_)_3_).

##### Methyl (*S*)-2-Benzyl-3-((*tert*-butoxycarbonyl)amino)-2-(phenylselanyl)propanoate (**14**)

Cyclic sulfamidate **10** (37 mg, 0.10 mmol), triethylamine (35 μL, 0.25 mmol), and freshly distilled benzeneselenol (9 μL, 0.08 mmol) were dissolved in DMF (2 mL) and stirred at 50 °C in an oil bath until the starting materials disappeared by TLC monitoring (30 min). Then, the solvent was removed, and the residue was dissolved in a mixture of CH_2_Cl_2_ and 20% aq. H_2_SO_4_ (1:1, 5 mL) and stirred for 3 h. After that time, the aqueous phase was extracted with CH_2_Cl_2_ (3 × 5 mL). The organic phases were combined and dried over anhydrous Na_2_SO_4_. The solvent was removed, and the crude product was purified by column chromatography (hexane/EtOAc, 9:1) to obtain the final product **14** as a colorless oil (39 mg, 88%). [α]_D_^25^ = −49.7 (*c* 1.00, CHCl_3_). HRMS (ESI) *m*/*z* [M + Na]^+^ calcd for C_22_H_27_NNaO_4_Se: 472.0998. Found: 472.1009. ^1^H NMR (CDCl_3_, 400 MHz): δ (ppm) 7.66 (d, 2H, *J* = 7.5 Hz, SePh), 7.53–7.41 (m, 1H, SePh), 7.37 (dd, 2H, *J* = 8.3. 6.8 Hz, SePh), 7.34–7.12 (m, 5H, *Ph*CH_2_), 5.19 (br s, 1H, NH), 3.66 (s, 3H, CO_2_Me), 3.61–3.47 (m, 2H, H^β^), 3.39 (d, 1H, *J* = 13.8 Hz, C*H*_2_Ph), 3.14 (d, 1H, *J* = 13.4 Hz, C*H*_2_Ph), 1.51 (s, 9H, Boc). ^13^C{^1^H} NMR (CDCl_3_, 100 MHz): δ (ppm) 172.9 (*C*O_2_Me), 155.7 (*C*O_2_C(CH_3_)_3_), 138.3, 136.0, 130.1, 129.7, 129.0, 128.5, 127.2, 126.1 (Ph), 79.5 (C^α^), 55.2 (CO_2_*C*(*C*H_3_)_3_), 52.1 (OMe), 44.1 (C^β^), 40.9 (Ph*C*H_2_), 28.4 (C(*C*H_3_)_3_).

##### Methyl (*S*)-2-Benzyl-3-((*tert*-butoxycarbonyl)amino)-2-fluoropropanoate (**15**)

Cyclic sulfamidate **10** (30 mg, 0.08 mmol) and 1 M solution of tetrabutylammonium fluoride in THF (105 μL, 0.105 mmol) were dissolved in DMF (2 mL) and stirred at 25 °C until the starting materials disappeared by TLC monitoring (5 h). Then, the solvent was removed, and the residue was dissolved in a mixture of CH_2_Cl_2_ and 20% aq. H_2_SO_4_ (1:1, 5 mL) and stirred for 3 h. After that time, the aqueous phase was extracted with CH_2_Cl_2_ (3 × 5 mL). The organic phases were combined and dried over anhydrous Na_2_SO_4_. The solvent was removed to give a mixture of two compounds in a ratio 79/21. The major compound was the desired product **15**, which was accompanied with a side product arising from an elimination reaction [methyl (*E*)-2-(((*tert*-butoxycarbonyl)amino)methyl)-3-phenyl acrylate] (**15b**). The mixture was purified by column chromatography (hexane/EtOAc, 8:2) to obtain compounds **15** (19 mg, 76%) and **15b** (4 mg, 17%), both as colorless oils. Data for compound **15**: [α]_D_^25^ = +15.5 (*c* 1.00, CHCl_3_). HRMS (ESI) *m*/*z* [M + Na]^+^ calcd for C_16_H_22_FNNaO_4_: 334.1425. Found: 334.1428. ^1^H NMR (CDCl_3_, 400 MHz): δ (ppm) 7.33–7.17 (m, 5H, *Ph*CH_2_), 4.88 (br s, 1H, NH), 3.85 (ddd, 1H, *J* = 12.9, 11.8, 7.5 Hz, H^β^), 3.73 (s, 3H, CO_2_Me), 3.52 (‘t’d, 1H, *J* = 14.8, 5.3 Hz, H^β^), 3.39–3.13 (m, 2H, C*H*_2_Ph), 1.47 (s, 9H, Boc). ^13^C{^1^H} NMR (CDCl_3_, 100 MHz): δ (ppm) 169.6 (*C*O_2_Me), 155.6 (*C*O_2_C(CH_3_)_3_), 133.7, 130.1, 128.5, 128.1, 127.4 (Ph), 97.0 (d, *J* = 190.3 Hz, C^α^), 80.0 (CO_2_*C*(*C*H_3_)_3_), 52.6 (OMe), 45.9 (d, *J* = 23.2 Hz, C^β^), 40.6 (d, *J* = 20.9 Hz, Ph*C*H_2_), 28.3 (C(*C*H_3_)_3_). ^19^F{^1^H} NMR (CDCl_3_, 282 MHz): −167.8. Data for compound **15b**: HRMS (ESI) *m*/*z* [M + Na]^+^ calcd for C_16_H_21_NNaO_4_: 314.1363. Found: 314.1369. ^1^H NMR (400 MHz, CDCl_3_) δ (ppm): 7.79 (s, 1H, CH=C), 7.54–7.32 (m, 5H, *Ph*CH_2_), 5.06 (br s, 1H, NH), 4.22 (d, 2H, *J* = 5.9 Hz, H^β^), 3.85 (s, 3H, CO_2_Me), 1.44 (s, 9H, Boc). Physical data agree to those previously reported.^44^

#### General Procedure for Hydrolysis of Ring-Opening Products

**Method A:** The corresponding ring-opening compound (0.04 mmol of **13** or **15**) was charged in a round-bottom flask with an aqueous 6 M solution of HCl (1 mL). The mixture was stirred for 14 h under reflux in an oil bath. The solvent was evaporated; the residue was dissolved in water (3 mL) and washed with EtOAc (3 mL). The aqueous phase was evaporated, and the corresponding amino acid hydrochloride salt (**17** or **19**) was obtained as a white solid. **Method B:** The ring-opening compound **12** (0.04 mmol) was dissolved in MeOH (2 mL), and LiOH·H_2_O (0.4 mmol) was added. The reaction mixture was stirred at room temperature until the starting materials disappeared by TLC monitoring (3 h). Then, an aqueous 2 M solution of HCl was added to give pH 2. The aqueous phase was evaporated, and the amino acid hydrochloride salt **16** was obtained as a white solid. **Method C:** The ring-opening compound **14** (0.04 mmol) was dissolved in MeOH (2 mL), and LiOH·H_2_O (0.4 mmol) was added. The reaction mixture was stirred at room temperature until the starting materials disappeared by TLC monitoring (3 h). An aqueous 2 M solution of HCl was added to adjust the pH to 6, and then CH_2_Cl_2_ (1 mL) and TFA (1 mL) were added. The mixture was stirred at room temperature for 1 h, and the solution was concentrated in vacuo to afford amino acid hydrochloride salt **18**.

##### (*S*)-2,3-Diamino-2-benzylpropanoic Acid Hydrochloride (**16**)

Yield 96% (6 mg). [α]_D_^25^ = +11.8 (*c* 1.00, H_2_O). HRMS (ESI) *m*/*z* [M]^+^ calcd for C_10_H_15_N_2_O_2_: 195.1128. Found: 195.1135. ^1^H NMR (D_2_O, 400 MHz): δ (ppm) 7.60–7.39 (m, 3H, *Ph*CH_2_), 7.33 (dd, 2H, *J* = 7.1, 2.4 Hz, *Ph*CH_2_), 3.67–3–55 (m, 2H, H^β^), 3.52 (d, 1H, *J* = 14.2, C*H*_2_Ph), 3.20 (d, 1H, *J* = 14.2, C*H*_2_Ph). ^13^C{^1^H} NMR (D_2_O, 100 MHz) δ (ppm): 171.2 (*C*O_2_H), 131.8, 130.3, 129.5, 128.7 (Ph), 61.5 (C^α^), 42.9 (C^β^), 39.5 (Ph*C*H_2_).

##### (*S*)-3-Amino-2-benzyl-2-(phenylthio)propanoic Acid Hydrochloride (**17**)

Yield 89% (7 mg). [α]_D_^25^ = +20.0 (*c* 1.00, H_2_O). HRMS (ESI) *m*/*z* [M]^+^ calcd for C_16_H_18_NO_2_S: 288.1053. Found: 288.1046. ^1^H NMR (D_2_O, 400 MHz): δ (ppm) 7.53 (d, 2H, *J* = 6.8 Hz, *Ph*S), 7.46–7.40 (m, 3H, *Ph*S), 7.31–7.16 (m, 5H, *Ph*CH_2_), 3.21 (d, 1H, *J* = 14.0 Hz, C*H*_2_Ph), 2.89–2.75 (m, 2H, C*H*_2_Ph, H^β^), 2.62 (d, 1H, *J* = 14.1 Hz, H^β^). ^13^C{^1^H} NMR (D_2_O, 100 MHz): δ (ppm) 176.4 (*C*O_2_H), 136.5, 136.4, 130.0, 129.8, 129.7, 129.3, 128.5, 127.1 (Ph), 63.3 (C^α^), 42.8 (C^β^), 41.0 (Ph*C*H_2_).

##### (*S*)-3-Amino-2-benzyl-2-(phenylselanyl)propanoic Acid Hydrochloride (**18**)

Yield 92% (10 mg). [α]_D_^25^ = +18.2 (*c* 1.00, H_2_O). HRMS (ESI) *m*/*z* [M]^+^ calcd for C_16_H_18_NO_2_Se: 336.0497. Found: 336.0497. ^1^H NMR (D_2_O, 400 MHz): δ (ppm) 7.62 (d, 2H, *J* = 7.4 Hz, *Ph*Se), 7.43 (t, 1H, *J* = 7.4 Hz, *Ph*Se), 7.34 (t, 2H, *J* = 7.4 Hz, *Ph*Se), 7.27–7.22 (m, 3H, *Ph*CH_2_), 7.14 (d, 2H, *J* = 6.9 Hz, *Ph*CH_2_), 3.48 (d, 1H, *J* = 12.0 Hz, C*H*_2_Ph), 3.11 (d, 1H, *J* = 12.0 Hz, H^β^), 2.96 (d, 1H, *J* = 16.0 Hz, C*H*_2_Ph), 2.90 (d, 1H, *J* = 16.0 Hz, H^β^). ^13^C{^1^H} NMR (D_2_O, 100 MHz): δ (ppm) 174.8 (*C*O_2_H), 137.8, 135.4, 130.4, 129.9, 129.7, 128.8, 127.7, 124.8 (Ph), 54.5 (C^α^), 42.7 (C^β^), 41.3 (Ph*C*H_2_).

##### (*S*)-3-Amino-2-benzyl-2-fluoropropanoic Acid Hydrochloride (**19**)

Yield 92% (7 mg). [α]_D_^25^ = +13.5 (*c* 1.00, H_2_O). HRMS (ESI) *m*/*z* [M]^+^ calcd for C_10_H_13_FNO_2_: 198.0925. Found: 198.0931. ^1^H NMR (D_2_O, 400 MHz): δ (ppm) 7.27–7.23 (m, 3H, *Ph*CH_2_), 7.17 (d, 2H, *J* = 7.1 Hz, *Ph*CH_2_), 3.58–3.46 (m, 1H, H^β^), 3.34 (‘t’, 1H, *J* = 13.0 Hz, H^β^), 3.17–3.11 (m, 2H, C*H*_2_Ph). ^13^C{^1^H} NMR (D_2_O, 100 MHz): δ (ppm) 172.4 (d, *J* = 24.6 Hz, *C*O_2_H), 133.4, 130.2, 128.6, 127.7 (Ph), 95.3 (d, *J* = 189.9 Hz, C^α^), 44.1 (d, *J* = 22.5 Hz, C^β^), 40.5 (d, *J* = 21.1 Hz, Ph*C*H_2_). ^19^F{^1^H} NMR (D_2_O, 282 MHz): −165.3.

##### Methyl (*S*)-2-Benzyl-3-((*tert*-butoxycarbonyl)amino)-2-(((*R*)-2-((*tert*-butoxycarbonyl)amino)-3-methoxy-3-oxopropyl)thio)propanoate (**20**)

Sulfamidate **10** (20 mg, 0.05 mmol), DBU (9 μL, 0.06 mmol), and *N*-Boc-l-Cys-OMe (13 mg, 0.05 mmol) were dissolved in DMF (2 mL) and stirred at room temperature until the starting materials disappeared (2 h). Then, the solvent was eliminated, and the residue was dissolved in a mixture of CH_2_Cl_2_ and H_2_SO_4_ 20% aq. (1:1, 4 mL) and stirred for 3 h. After that time, the aqueous phase was extracted with CH_2_Cl_2_ (3 × 4 mL). The organic phases were combined and dried over anhydrous Na_2_SO_4_. The solvent was removed, and the crude product was purified by column chromatography (hexane/EtOAc, 7:3) to obtain the final product **20** as a colorless oil. Yield 88% (25 mg). [α]_D_^25^ = −18.0 (*c* 1.00, H_2_O). HRMS (ESI) *m*/*z* [M + Na]^+^ calcd for C_25_H_38_N_2_NaO_8_S: 549.2241. Found: 549.2244. ^1^H NMR (CDCl_3_, 400 MHz): δ (ppm) 7.37–6.93 (m, 5H, *Ph*CH_2_), 4.45 (s, 1H, H^α^_cys_), 3.71 (s, 6H, 2 CO_2_Me), 3.56–3.29 (m, 2H, C*H*_2_Ph), 3.22 (dd, 1H, *J* = 13.6, 7.2, Hz, H^β^), 3.06–2.86 (m, 3H, 2H^β^_cys_, H^β^), 1.39 (s, 9H, Boc), 1.38 (s, 9H, Boc). ^13^C{^1^H} NMR (CDCl_3_, 100 MHz) δ (ppm): 171.9 (*C*O_2_Me), 171.1 (*C*O_2_Me), 155.8 (*C*O_2_*C*(CH_3_)_3_), 155.2 (*C*O_2_*C*(CH_3_)_3_), 135.0, 130.0, 128.5, 127.4 (Ph), 80.3 (CO_2_*C*(CH_3_)_3_), 79.7 (CO_2_*C*(CH_3_)_3_), 77.2 (C^α^), 52.7 (OMe), 52.6 (OMe), 54.5 (C^α^_cys_), 42.9 (C^β^), 42.7 (C^β^), 31.6 (Ph*C*H_2_), 28.4 (C(*C*H_3_)_3_), 28.3 (C(*C*H_3_)_3_).

##### Methyl (2*S*,5*S*,11*R*,14*S*)-11-Acetamido-1-amino-14-benzyl-14-(((*tert*-butoxycarbonyl)amino)methyl)-5-isopropyl-2-methyl-1,4,7,10-tetraoxo-13-thia-3,6,9-triazapentadecan-15-oate (**21**)

Sulfamidate **10** (19 mg, 0.05 mmol), Et_3_N (21 μL, 0.15 mmol), and Ac-CGVA-NH_2_ (24 mg, 0.06 mmol) were dissolved in DMF (2 mL). The reaction was stirred at room temperature followed by analytical RP-HPLC. After semipreparative RP-HPLC purification, peptide **21** was obtained as a white solid using the following conditions: a Phenomenex Luna C18(2) column (10 μ, 250 mm × 21.2 mm) and a dual-absorbance detector with a flow rate of 20 mL/min. Retention time (*R*_t_) = 34.02 min (gradient: acetonitrile/water + 0.1% TFA (22.5:77.5) → (77.5:22.5), 37 min, λ = 212 nm). Yield 49% (17 mg). UPLC-MS: *R*_t_ = 4.92 min (Acquity UPLC BEH 1.7 μm C18, 2.1 × 100 mm (gradient: acetonitrile/water + 0.1% formic acid (5:95) → (100:0), 10 min, 0.45 mL/min, λ = 212 nm, 254 nm)). HRMS (ESI) *m*/*z* [M + Na]^+^ calcd for C_31_H_48_N_6_NaO_9_S: 703.3096. Found: 703.3084. ^1^H NMR (DMF-*d*_7_, 400 MHz): δ (ppm) 8.67 (t, 1H, *J* = 5.8 Hz, NH), 8.58 (d, 1H, *J* = 7.4 Hz, NH), 8.14 (t, 1H, *J* = 7.5 Hz, NH), 7.99 (t, 1H, *J* = 8.2 Hz, NH), 7.44–7.15 (m, 7H, Ph, NH_2_), 7.17 (t, 1H, *J* = 5.8 Hz, NH), 4.64 (t, 1H, *J* = 9.7 Hz, H^α^_Lan_), 4.57–4.51 (m, 1H, H^α^_Ala_), 4.48–4.33 (m, 1H, H^α^_Val_), 4.16 (dd, 1H, *J* = 16.9, 6.3 Hz, H^β^_Lan_), 4.04–4.00 (m, 1H, H^β^_Lan_), 3.64 (s, 2H, H^α^_Gly_), 3.46 (s, 3H, OMe), 3.35 (d, 2H, *J* = 5.0 Hz, C*H*_2_Ph), 3.31 (dd, 1H, *J* = 8.2, 4.6 Hz, H^β^_Lan_), 3.18–3.12 (m, 1H, H^β^_Cys_), 2.38–2.28 (m, 1H, H^β^_Val_), 2.15 (s, 3H, NHCOC*H*_3_), 1.60 (s, 9H, NH*Boc*), 1.50 (d, 3H, *J* = 7.0 Hz, CH_3Ala_), 1.09 (dd, 6H, *J* = 12.8, 6.7 Hz, 2CH_3Val_). ^13^C{^1^H} NMR (DMF-*d*_7_, 100 MHz): δ (ppm) 174.8 (*C*O_2_Me), 172.0, 171.4, 171.0, 170.8, 169.7 (CON), 136.1 (*C*(CH_3_)_3_), 130.8, 130.3, 128.7, 128.4, 127.2 (Ph), 117.9 (CO_2_*C*(CH_3_)_3_), 77.2 (C^α^), 58.9 (C^α^_Val_), 53.7 (C^α^_cys_), 49.5 (C^α^_Ala_), 43.1 (C^β^), 42.8 (C^α^_Gly_), 39.2 (Ph*C*H_2_), 30.8 (C^β^_Val_), 30.7 (C^β^_Lan_), 28.1 (CO_2_C(*C*H_3_)_3_), 22.3 (NHCO*C*H_3_), 19.1 (CH_3Val_), 18.0 (CH_3Val_), 17.7 (CH_3Ala_).

### Quantum Mechanical Calculations

Full geometry optimizations were carried out with Gaussian 16^45^ using the M06-2X hybrid functional^46^ and 6-31+G(d,p) basis set in combination with ultrafine integration grids. Bulk solvent effects in toluene and tetrahydrofuran were considered implicitly through the IEF-PCM polarizable continuum model.^47^ The possibility of different conformations was taken into account. Frequency analyses were carried out at the same level used in the geometry optimizations, and the nature of the stationary points was determined in each case according to the appropriate number of negative eigenvalues of the Hessian matrix. The quasiharmonic approximation reported by Truhlar et al. was used to replace the harmonic oscillator approximation for calculation of the vibrational contribution to enthalpy and entropy.^48^ Scaled frequencies were not considered. Mass-weighted intrinsic reaction coordinate (IRC) calculations were carried out using the Gonzalez and Schlegel scheme^49,50^ in order to ensure that the TSs indeed connected the appropriate reactants and products. The complex nature of the enolate inversion of **2′** to **2′_epi**, which is also coupled with a conformational change at the oxazolidinone ring, caused the IRC calculations to fail in both the forward and the reverse directions. Gibbs free energies (Δ*G*) were used for the discussion on the relative stabilities of the considered structures. The lowest energy conformer for each calculated stationary point was considered in the discussion; all computed structures can be obtained from the authors upon request. Cartesian coordinates, electronic energies, entropies, enthalpies, Gibbs free energies, and lowest frequencies of the calculated structures are available in the Supporting Information.

### X-ray Diffraction Analysis

CCDC 2122265–2122266 contain the supplementary crystallographic data for this paper. The SHELXL97 program^51^ was used for refinement of the ecrystal structures, and hydrogen atoms were fitted at theoretical positions.

### Determination of the Enantiomeric Purity of β-Amino Acids **7c** and **8c**

Following a recent but slightly modified procedure,^52,53^ the corresponding amino acid **7c**, **8c**, or a mixture of both was dissolved in D_2_O to prepare a 0.05 M solution. The pH of these three solutions was adjusted to 10 with a 1 M KOH solution in D_2_O. Then, a solution of 8 mg/mL of samarium(III) complex with (*S*,*S*)-ethylenediamine-*N*,*N*′-disuccinate in D_2_O was prepared, and 0.2 mL of this solution was added to each of the corresponding NMR tubes containing 0.5 mL of a solution of amino acids **7c**, **8c**, or a mixture of both. The ^1^H NMR experiments were registered in a 400 MHz spectrometer at 298 K. Under these conditions, the doublet corresponding to the C*H*_*a*_H_b_Ph signal appears separated by 0.02 ppm for both enantiomers, allowing their integration. Thus, in the case of β-amino acid **7c**, this signal appears at 2.63 ppm (d, 1H, *J* = 13.7 Hz), while the same signal in the case of β-amino acid **8c** appears at 2.65 ppm (d, 1H, *J* = 13.6 Hz). Taking into account that in the spectrum of each amino acid no signals of the other enantiomer were observed, we conclude that the enantiomeric purity for each of them is >95:5 (Supporting Information).

## Supporting Information Available

Additional experimental details, computational data and copies of ^1^H and ^13^C NMR spectra as well as 2D NMR spectra (COSY, edited-HSQC and NOESY) for all new compounds (PDF)
FAIR data, including the primary NMR FID files, for compounds **2** and **3** (ZIP)

## Accession Codes

CCDC 2122265–2122266 contain the supplementary crystallographic data for this paper. These data can be obtained free of charge via www.ccdc.cam.ac.uk/data_request/cif, or by emailing data_request@ccdc.cam.ac.uk, or by contacting The Cambridge Crystallographic Data Centre, 12 Union Road, Cambridge CB2 1EZ, UK; fax: +44 1223 336033.
