# Supplementary material for: Synthesis of β2,2-Amino Acids by Stereoselective Alkylation of Isoserine Derivatives Followed by Nucleophilic Ring Opening of Quaternary Sulfamidates
Source: J Org Chem. 2022 Jun 22;87(13):8730–43. doi: 10.1021/acs.joc.2c01034 (PMC9490828; doi:10.1021/acs.joc.2c01034)
Supplement: Supplementary file 1 — jo2c01034_si_001.pdf [file jo2c01034_si_001.pdf]

Supporting Information

Synthesis of $\beta^{2,2}$-Amino Acids by Stereoselective Alkylation of Isoserine Derivatives Followed by Nucleophilic Ring-Opening of Quaternary Sulfamidates

Pablo Tovillas,[†,a] Claudio D. Navo,[†,b] Paula Oroz,[a] Alberto Avenoza,[a] Francisco Corzana,[a] María M. Zurbano,[a] Gonzalo Jiménez-Osés,[b,c] Jesús H. Busto,[a,*] and Jesús M. Peregrina[a,*]

[a] Departamento de Química, Centro de Investigación en Síntesis Química, Universidad de La Rioja, 26006 Logroño, La Rioja, Spain.

[b] Center for Cooperative Research in Biosciences (CIC bioGUNE), Basque Research and Technology Alliance (BRTA), Bizkaia Technology Park, Building 800, 48160 Derio, Spain.

[c] Ikerbasque, Basque Foundation for Science, 48013 Bilbao, Spain.

* Email: jesusmanuel.peregrina@unirioja.es hector.busto@unirioja.es

[†] *P.T. and C.D.N. contributed equally to this work.*

Contents

1. Additional tables to follow the text of the manuscript S02-S03
2. Diastereomeric purity determination S04-S08
3. NMR spectra S09-S62
4. Chromatogram for peptide **21** S63
5. Computational details........ S64-S88
6. X-Ray diffraction analysis S89-S90
7. Enantiomeric purity determination of **7c** and **8c** by NMR chiral shift reagents S91-S93

1. Additional tables to follow the text of the manuscript

Table S1. Formation of chiral *N,O*-acetals **2**, **3** and **4** from Boc-L-*iso*Ser-OMe (**1**) and TMB.

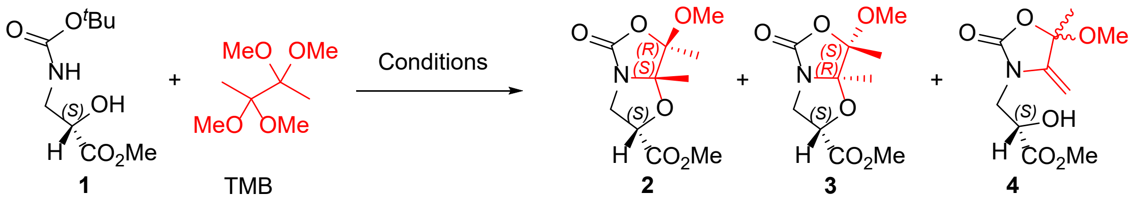

TMB = 2,2,3,3-tetramethoxybutane

Entry	Solvent	Temperature	Time	Acid (equiv)	mmol 1	Yield 2+3 (%)	Ratio 2/3	Yield 4 (%)
**1**	toluene	reflux	4 h	TsOH·H_2O (0.1)	0.91	51	0.69/0.31	34
**2**	toluene	reflux	24 h	TsOH·H_2O (0.1)	0.46	64	0.64/0.36	27
**3**	toluene	reflux	1 h	TsOH·H_2O (0.5)	0.46	87	0.60/0.40	13
**4**	toluene	reflux	1 h	CSA·H_2O (0.1)	0.91	57	0.72/0.28	29
**5**	toluene	reflux	1 h	CSA·H_2O (0.2)	0.1	88	0.63/0.37	11
**6**	toluene	80 °C	1 h	TsOH·H_2O (0.2)	0.46	86	0.62/0.38	14
**7**	toluene	80 °C	1 h	CSA·H_2O (0.2)	0.91	87	0.63/0.37	13
**8**	toluene	80 °C	1 h	CSA·H_2O (0.1)	0.91	57	0.74/0.26	29
**9**	toluene	80 °C	1 h	BF_3·Et_2O (0.2)	0.46	62	0.52/0.48	38
**10**	toluene	50 °C	1 h	TsOH·H_2O (0.2)	0.46	58	0.79/0.21	25
**11**	toluene	reflux	30 min	H_2SO_4 (2)	0.46	85	0.60/0.40	15
**12**	xylene	reflux	15 min	H_2SO_4 (2)	0.46	68	0.59/0.41	3
**13**	xylene	reflux	5 min	H_2SO_4 (1)	0.46	91	0.66/0.34	9
**14**	xylene	125 °C	30 min	TsOH·H_2O (1)	0.46	62	0.79/0.21	38
**15**	toluene	reflux	1 h	CSA·H_2O (0.2)	13.7	85	0.63/0.37	13

Only the conditions of entry 15 gave good results when we tried to scale up the reaction. For this reason, other entries such as 5, 7 and 13 were not considered.

Table S2. Diastereoselective alkylation of chiral bicyclic *N,O*-acetal **2**.

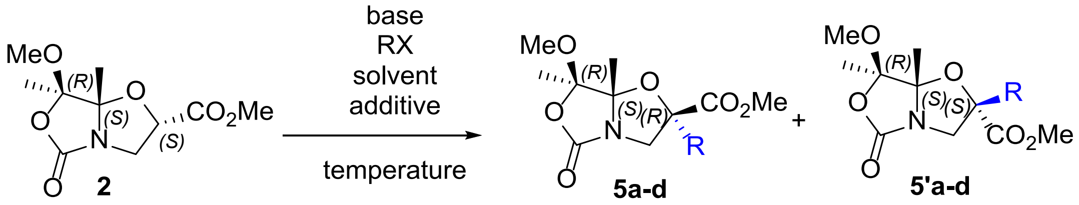

Entry	RX	Base (equiv)	Solvent	Additive	Temperature	Time (min)	Ratio 5a-d/5'a-d	Yield 5a-d+5'a-d (%)
**1**	MeI	LHMDS (2.0)	THF	HMPA	-78 °C	5	**5a/5'a** 83/17	95
**2**	MeI	KHDMS (2.0)	THF	-	-78 °C	5	**5a/5'a** 46/14	55
**3**	MeI	LHDMS (2.0)	THF	-	-78 °C	60	- -/-	-
**4**	MeI	LHDMS (2.0)	THF	-	-50 °C	60	- -/-	-
**5**	MeI	LHDMS (2.0)	THF	HMPA	-90 °C	15	**5a/5'a** 88/12	95
**6**	MeI	LHDMS (2.0)	THF	HMPA	-90 °C	5	**5a/5'a** 88/12	95
**7**	MeI	LHDMS (2.0)	Et_2O	HMPA	-78 °C	5	**5a/5'a** 62/17	84
**8**	MeI	LHMDS (1.2)	THF	HMPA	-78 °C	60	**5a/5'a** 32/3	30
**9**	AllylI	LHDMS (2.0)	THF	HMPA	-78 °C	10	**5d/5'd** 87/13	93
**10**	BnBr	LHDMS (2.0)	THF	HMPA	-78 °C	10	**5c/5'c** 73/18	84
**11**	BnI	LHDMS (2.0)	THF	HMPA	-78 °C	10	**5c/5'c** 80/20	91
**12**	EtI	LHDMS (2.0)	THF	HMPA	-78 °C	60	**5b/5'b** 14/-	12
**13**	EtOTf	LHDMS (2.0)	THF	HMPA	-78 °C	10	**5b/5'b** 85/15	92

2. Diastereomeric purity determination

Purity of building blocks 2 and 3.

After an easy purification by column chromatography, compound **2** was used as starting material with a 98:2 diastereomeric ratio with respect to diastereomer **3**. In the case of compound **3** the diastereomeric ratio after column chromatography was 99:1 with respect to diastereomer **2**.

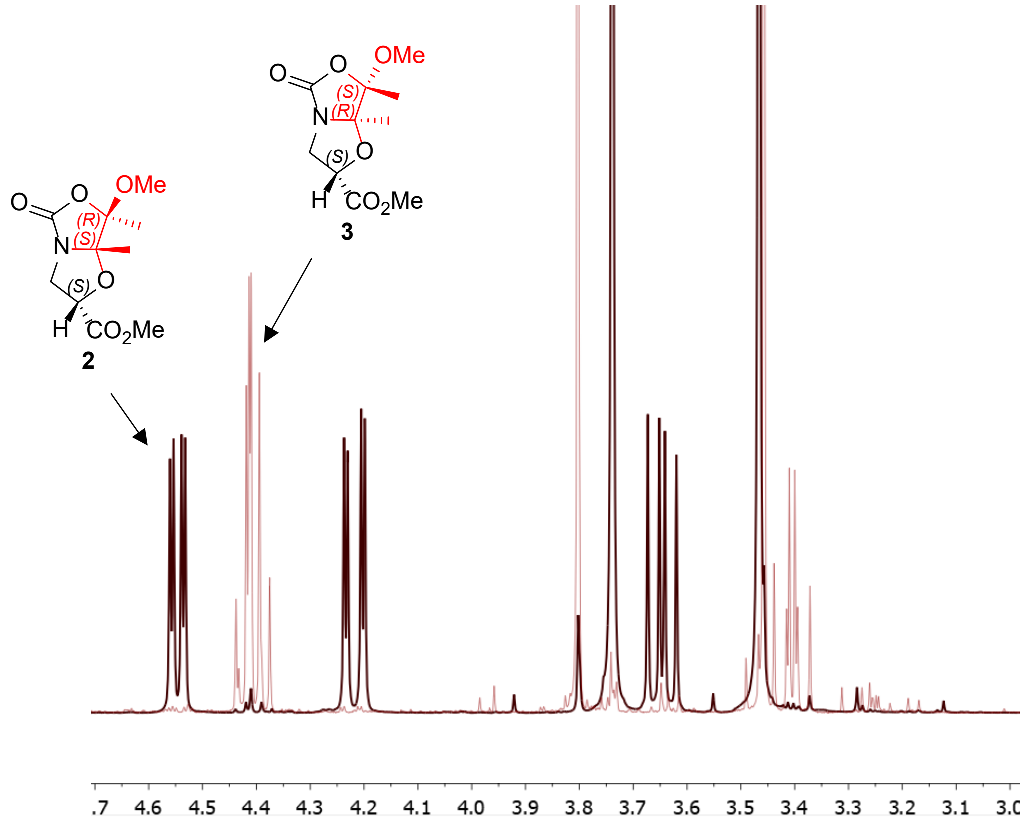

Figure S1. *Overlapping of ^{1}H NMR spectra corresponding to compounds **2** and **3**.*

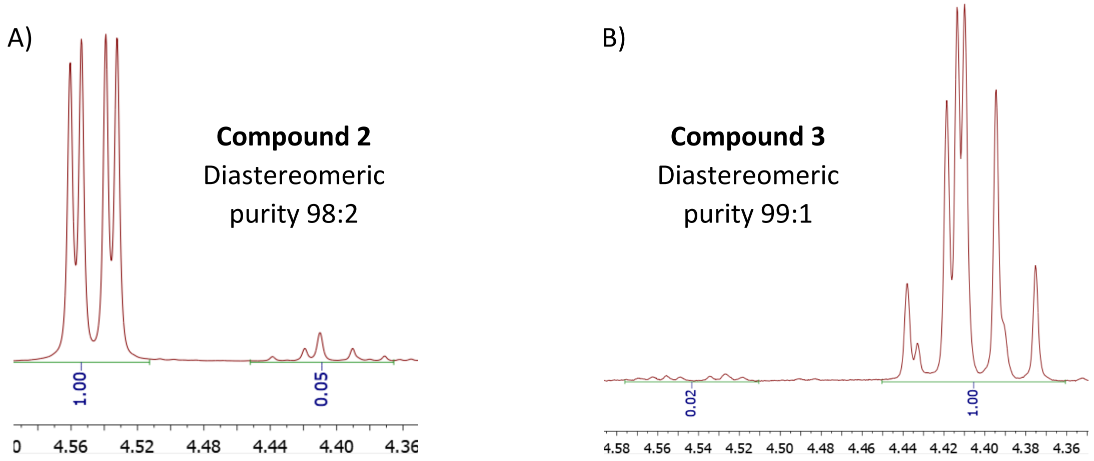

Figure S2. *^{1}H NMR zoom corresponding to spectra of starting materials after purification by column chromatography: compound **2** (A) and compound **3** (B).*

Purity of alkylated compounds and derivatives.

1) The ratio of diastereoisomers for alkylation of compound **2** with methyl iodide to give **5a** as a major compound is 83/17. In the same way, the ratio of diastereoisomers for alkylation of compound **3** with methyl iodide to give **6a** (enantiomer of **5a**) as the major compound is 82/18. In both cases, these ratios were measured by ^{1}H NMR of the crude reaction mixture.

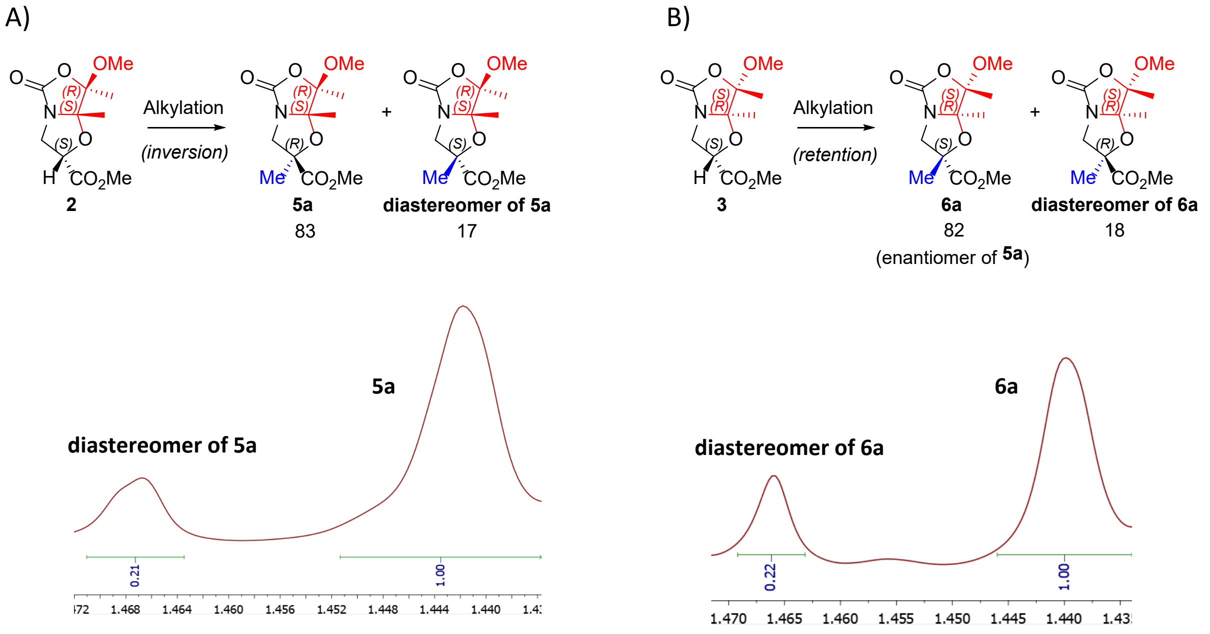

Figure S3. *Ratio of diastereomers extracted from the* ^{1}H *NMR spectra of the crude alkylation reaction mixtures of* ***2*** *(A) and* ***3*** *(B) with MeI.*

After purification by column chromatography, in the ^{1}H NMR spectrum of compound **5a**, the minor diastereomer was not observed. Therefore, its diasteromeric purity is >99:1. However, after purification, compound **6a** showed some quantity of its diastereomer, which was integrated (98:2).

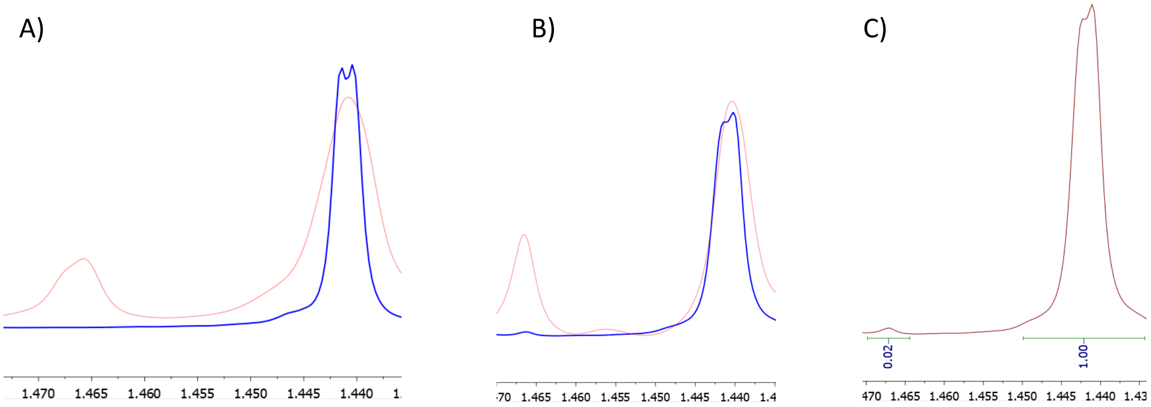

Figure S4. *Peaks extracted from the* ^{1}H *NMR spectra showing in red the mixture of diastereomers from the crude alkylation reactions and in blue the pure compounds* ***5a*** *(A) and* ***6a*** *(B) after purification by column chromatography. (C) Integration of compound* ***6a****.*

Taking into account the starting purity of compound **2** (98% of **2** and 2% of **3**), and that alkylation of the minor isomer (**3**) gives the enantiomer of the alkylated compound (**6a**), subsequent hydrolysis to obtain the amino acid gives compound **7a** with an ee = 96%. Similar features occurred for amino acid **8a**, which was obtained with an ee = 94%.

2) The diastereomeric ratio for alkylation reaction of compound **2** with ethyl triflate to give **5b** is 85/15 measured by ^{1}H NMR of the crude reaction mixture.

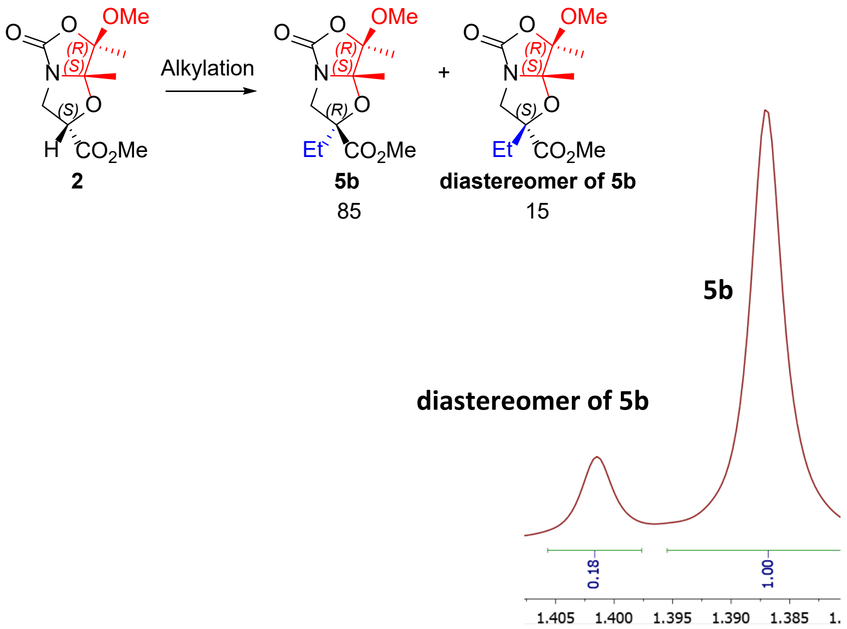

Figure S5. *Ratio of diastereomers extracted from the ^{1}H NMR spectrum of the crude alkylation reaction mixture of **2** with EtOTf.*

After purification by column chromatography, the corresponding compound **5b** has a diastereomeric purity of 95:5 with respect to the minor diastereomer.

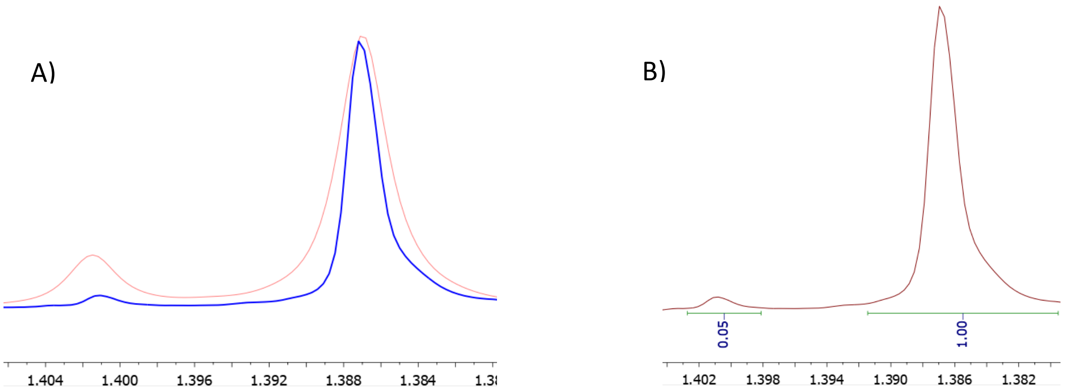

Figure S6. *A) In red the mixture 85:15 of diastereomers and in blue the purified compound **5b** in 95:5 ratio. B) Integration of compound **5b** after purification by a column chromatography.*

Taking into account the starting purity of compound **2** (98% of **2** and 2% of **3**), and that alkylation of the minor isomer (**3**) gives the enantiomer of the alkylated compound, subsequent hydrolysis to obtain the amino acid gives compound **7b** with an ee = 86%.

3) The diastereomeric ratio for alkylation reaction of compound **2** with benzyl iodide to give **5c** is 80/20. In the same way, the ratio of diastereoisomers for alkylation of compound **3** with benzyl iodide to give **6c** (enantiomer of **5c**) as the major compound is 80/20. In both cases, these ratios were measured by ^{1}H NMR of the crude reaction mixtures.

A) B)

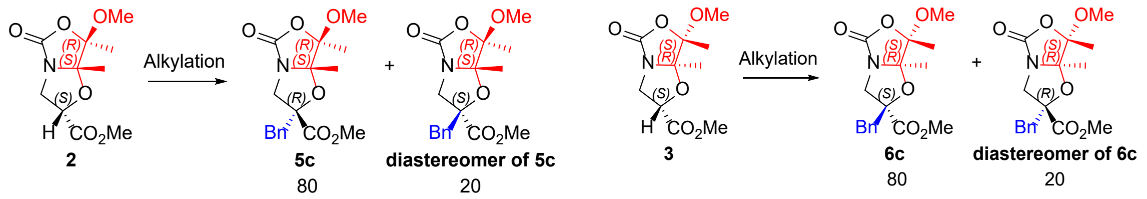

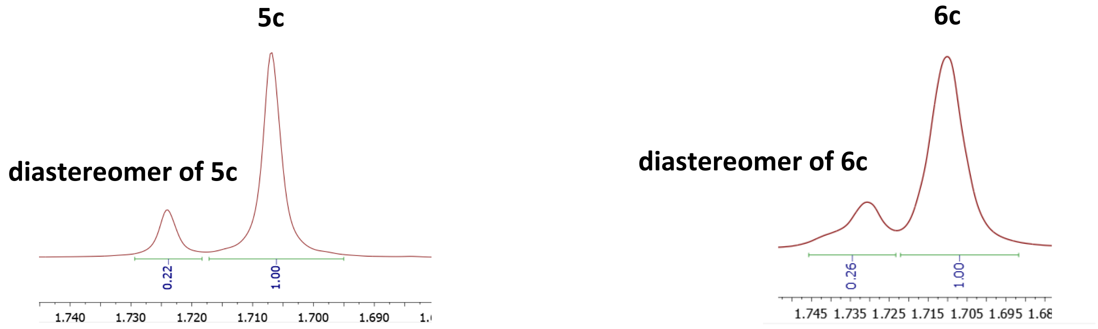

Figure S7. *Ratio of diastereomers extracted from the ^{1}H NMR spectra of the crude alkylation reaction mixture of **2** (A) and **3** (B) with BnI.*

After purification by column chromatography, in the ^{1}H NMR spectrum of the corresponding compound **5c**, used as starting material for sulfamidate chemistry, the minor diastereomer was not observed. In the same way, after purification by column chromatography the corresponding compound **6c** was obtained in a 98:2 ratio respect to its diastereomer.

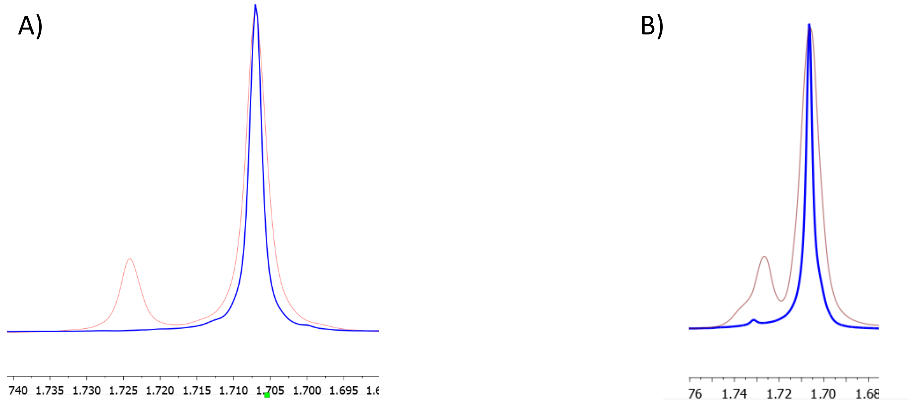

Figure S8. *In red the mixture 80:20 of diastereoisomers. In blue the purified compounds **5c** (A) and **6c** (B). Compound **5b** appears as a pure compound and **6c** in a 98:2 ratio.*

Taking into account the starting purity of compound **2** (98% of **2** and 2% of **3**), and that alkylation of the minority isomer gives the enantiomer of the alkylated compound, enantiomeric excess of **5c** is ee = 96%. Subsequent hydrolysis to obtain the amino acid gives compound **7c** with an ee = 96%. The same enantiomeric excess was obtained for the sulfamidate **10** and the amino acid derivatives, which undergo reactions without loss of enantiomeric purity (ring-opening and hydrolysis reactions).

4) The diastereomeric ratio for alkylation reaction of compound **2** with allyl iodide to give **5c** is 87/13 measured by ^{1}H NMR of the crude reaction mixture.

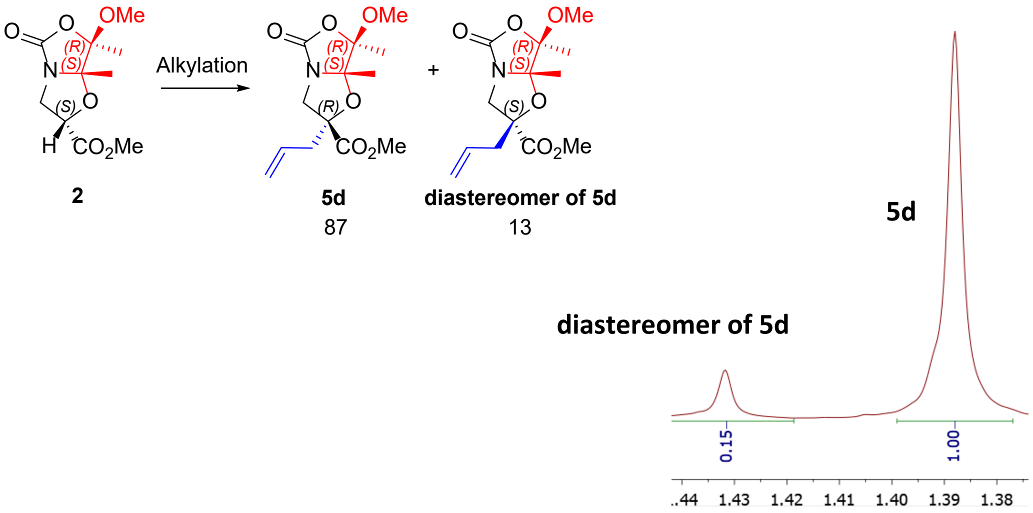

Figure S9. *Ratio of diastereoisomers extracted from the ^{1}H NMR spectrum of the crude alkylation reaction mixture of **2** with allyl iodide.*

After purification by column chromatography, in the ^{1}H NMR spectrum of the corresponding compound **5d**, the minor diastereomer was not observed.

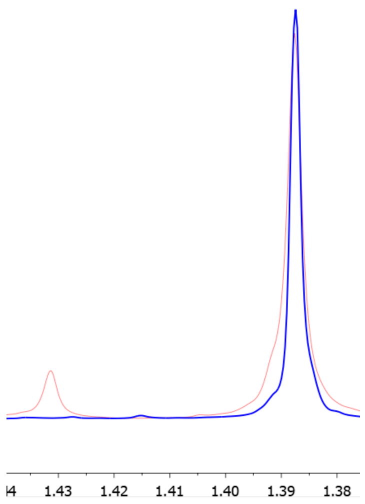

Figure S10. *In red de mixture 87:13 of diastereoisomers. In blue the pure compound **5d**.*

4. NMR spectra

^{1}H NMR 400 MHz in $CDCl_3$

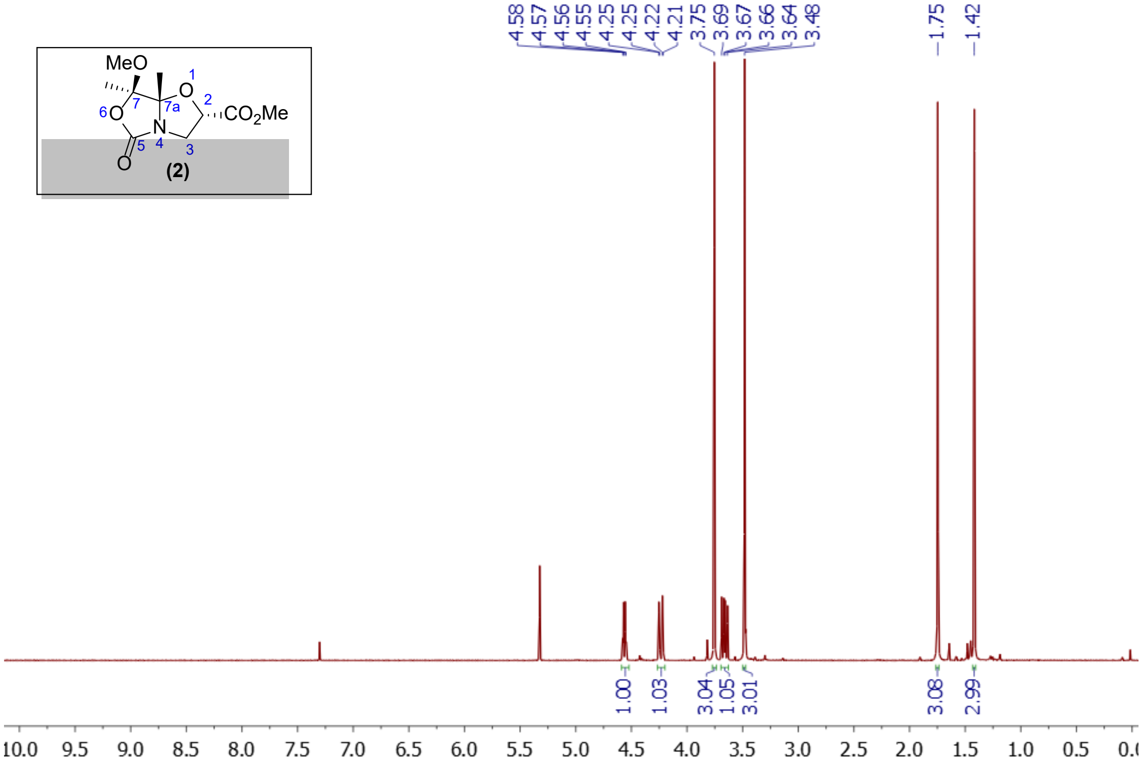

$^{13}C\{^1H\}$ NMR 100 MHz in $CDCl_3$

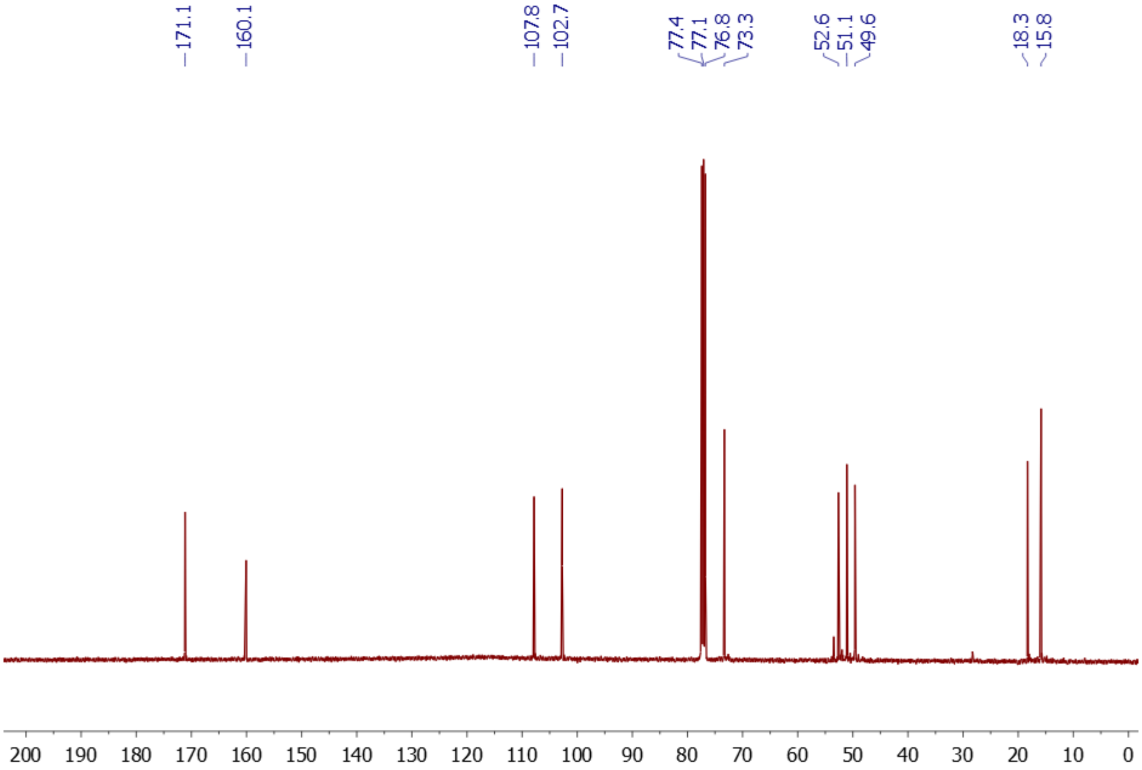

COSY in $CDCl_3$

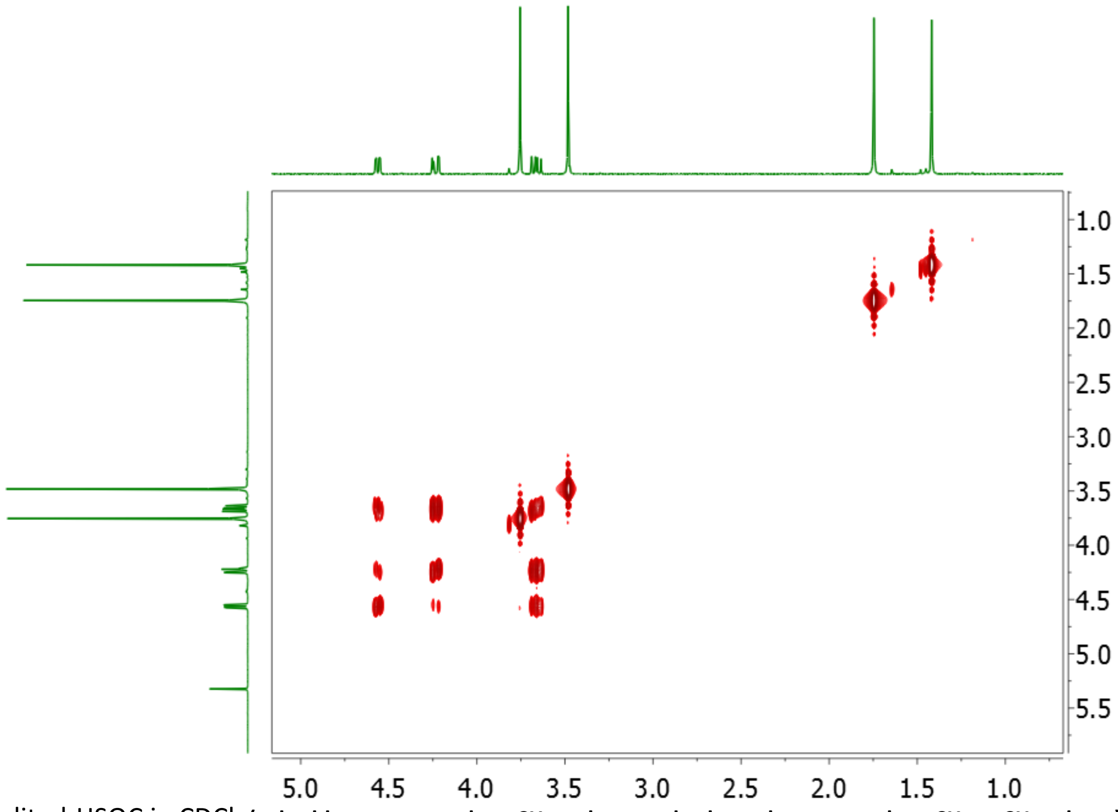

edited-HSQC in $CDCl_3$ **(color blue corresponds to CH_2 carbons and color red corresponds to CH_3 or CH carbons)**

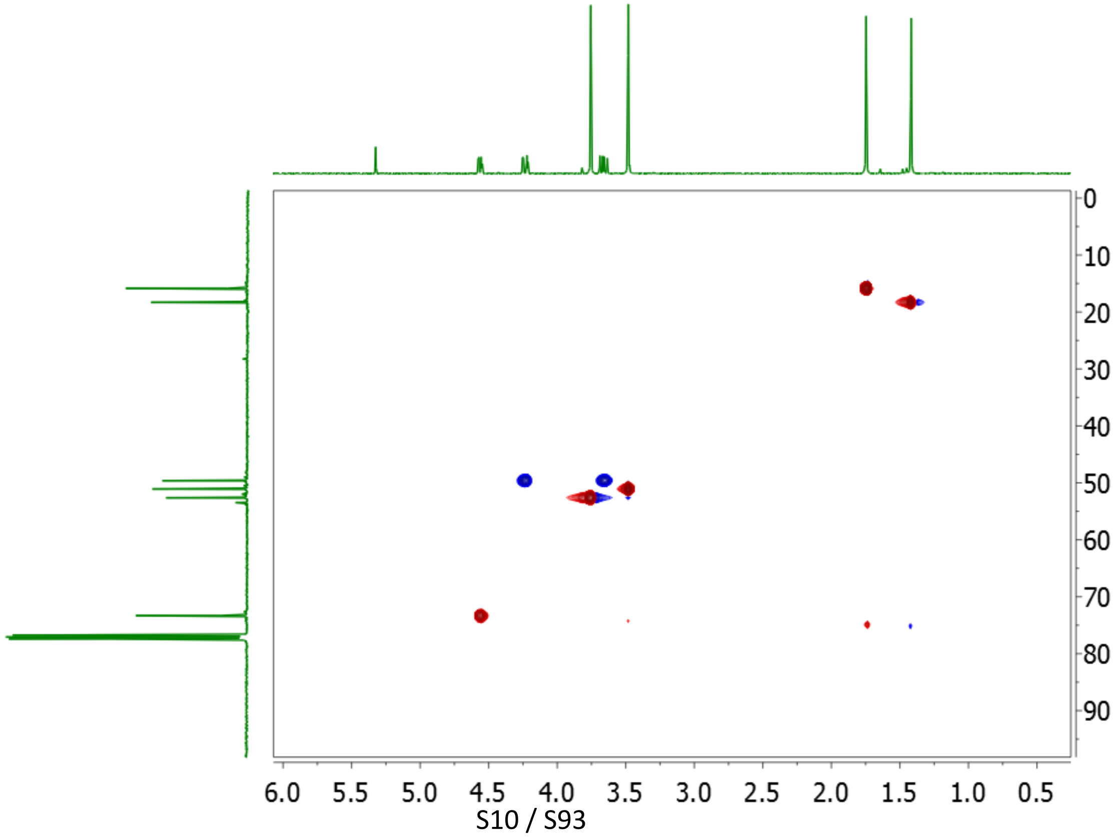

HMBC in $CDCl_3$

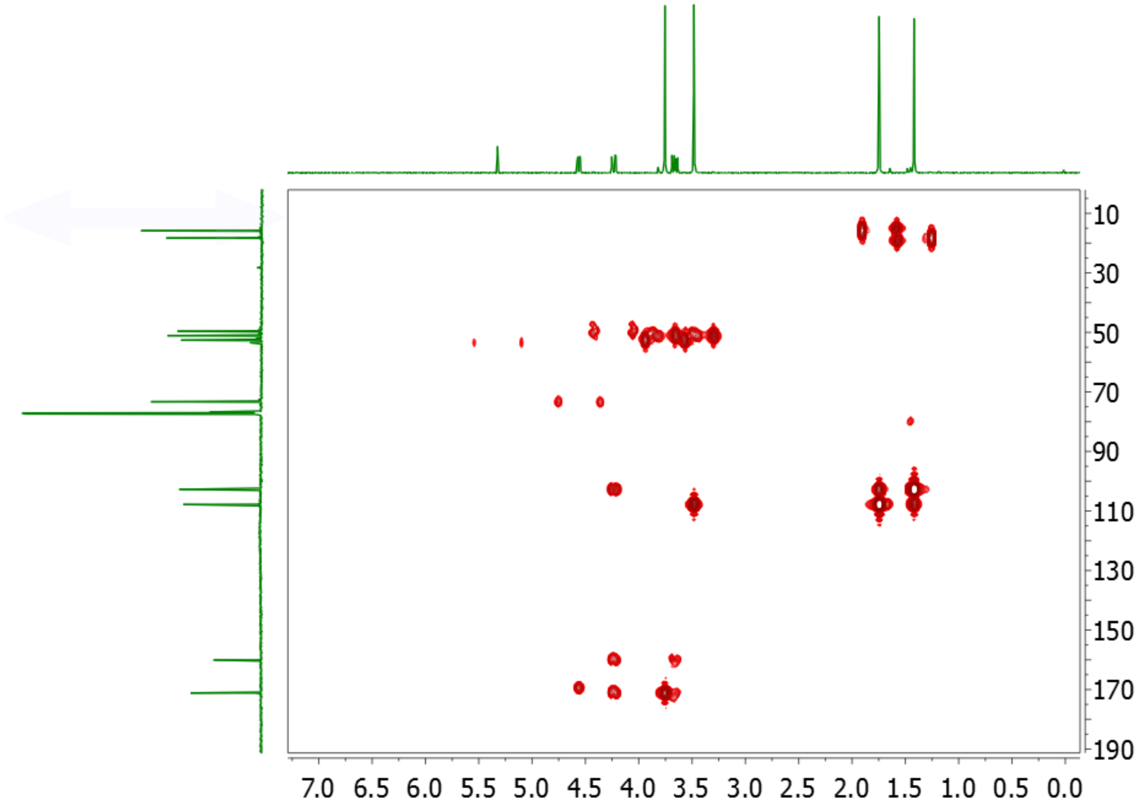

NOESY in $CDCl_3$

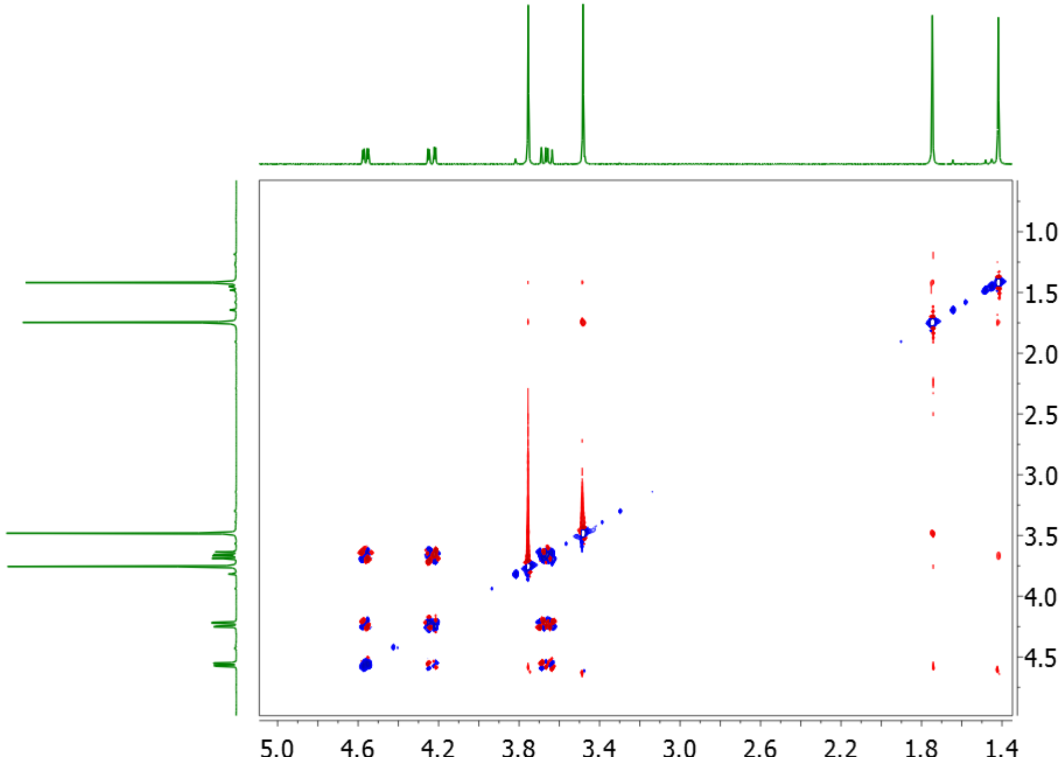

^1H NMR 400 MHz in $CDCl_3$

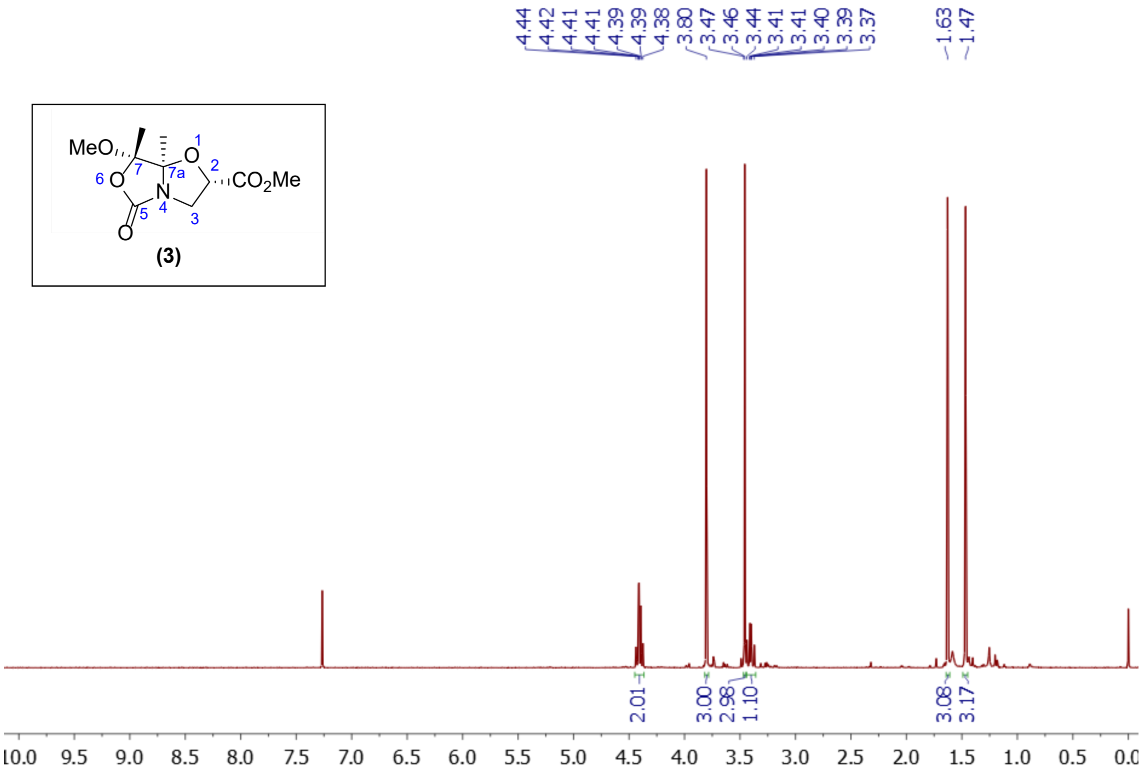

(3)

$^{13}C\{^1H\}$ NMR 100 MHz in $CDCl_3$

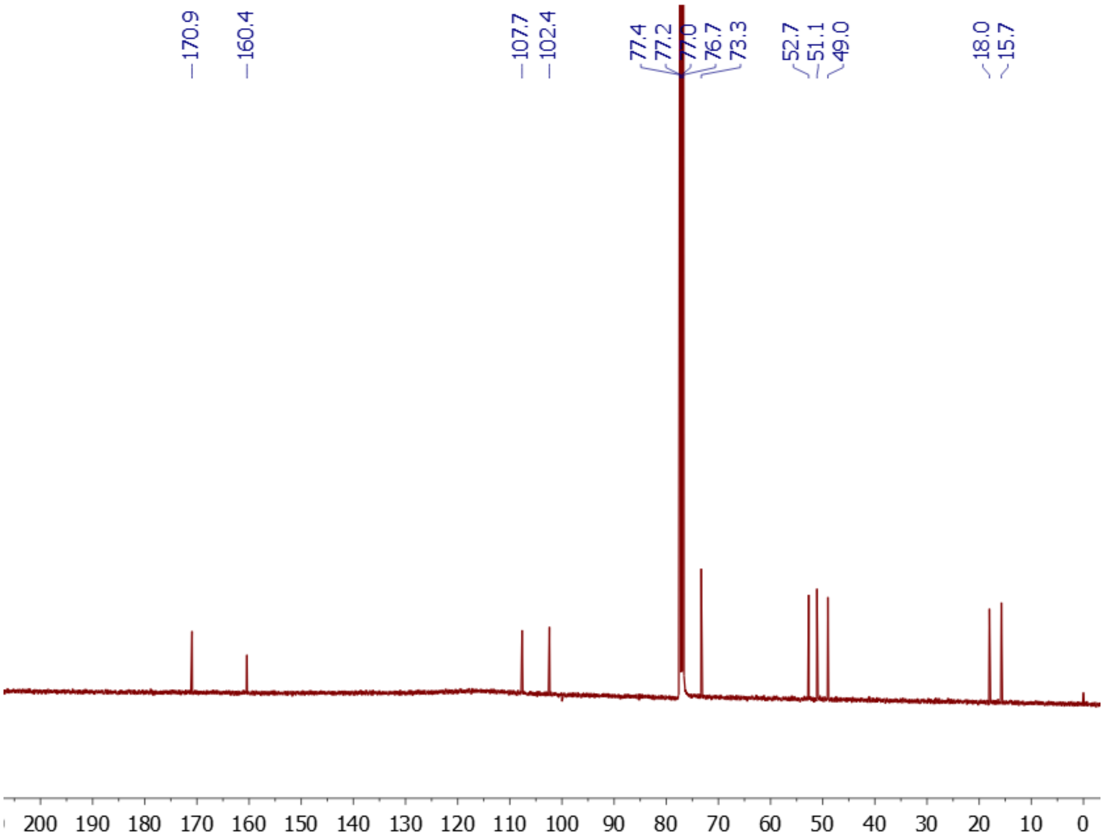

COSY in $CDCl_3$

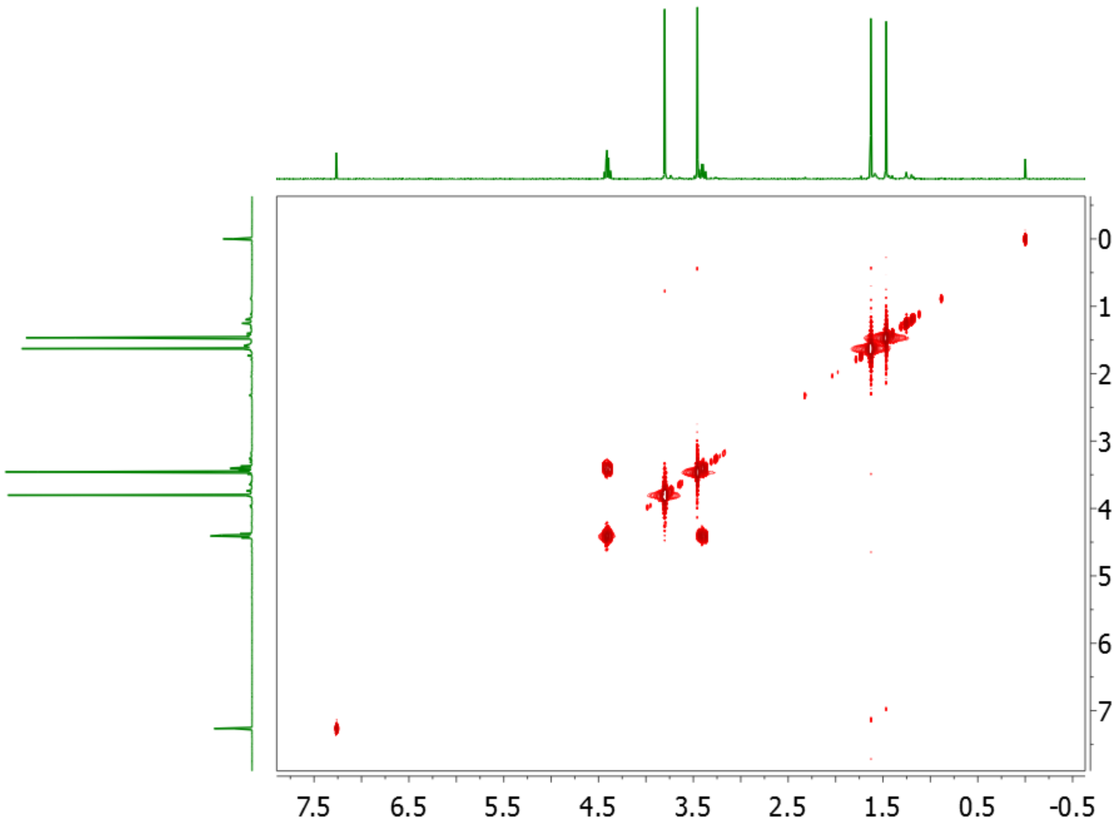

edited-HSQC in $CDCl_3$ **(color blue corresponds to CH_2 carbons and color red corresponds to CH_3 or CH carbons)**

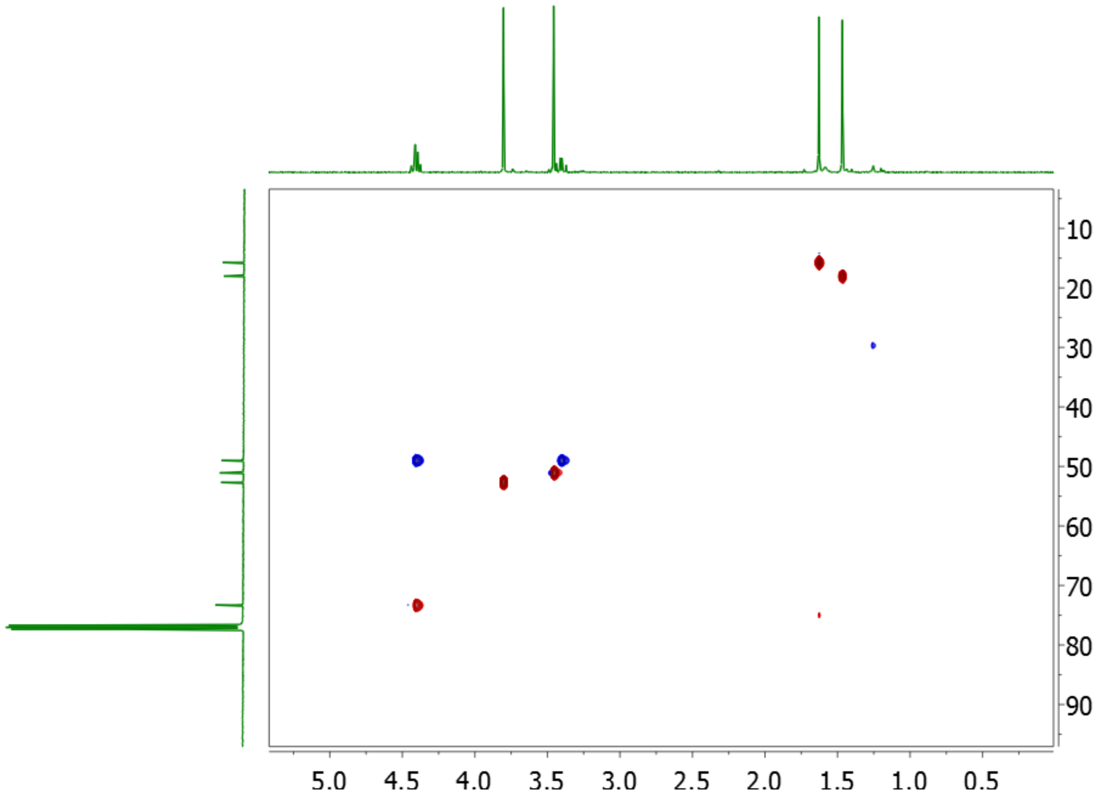

HMBC in $CDCl_3$

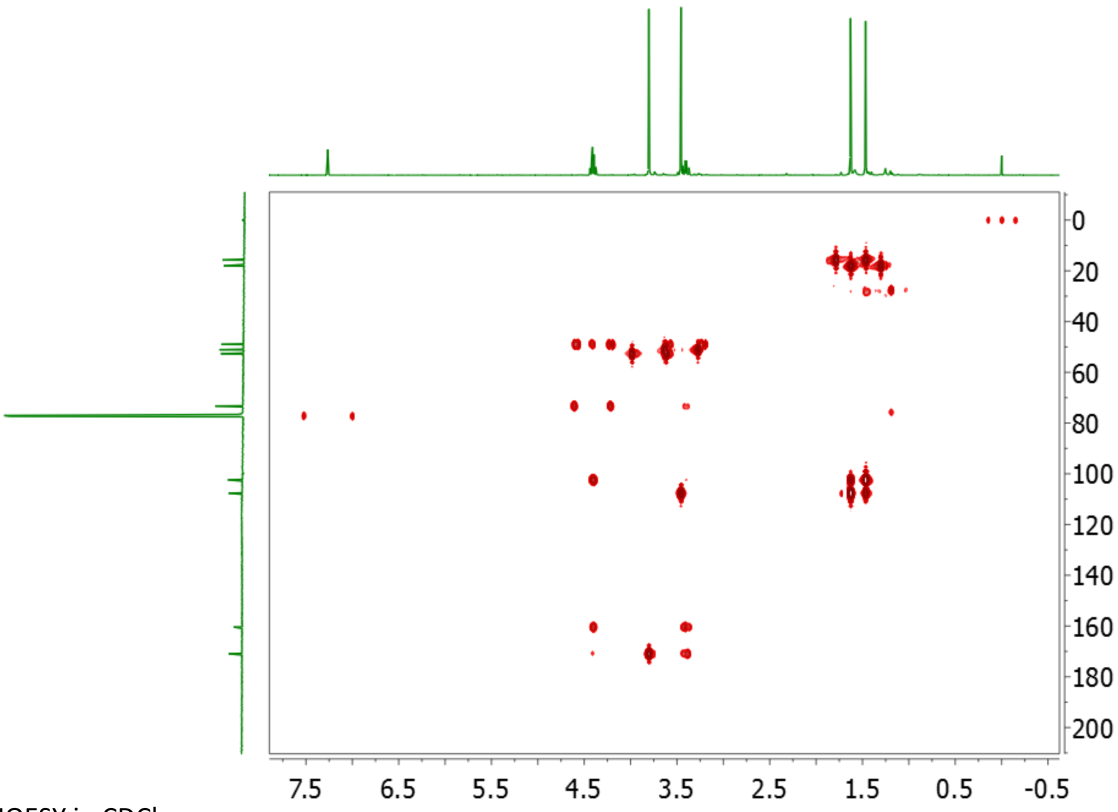

NOESY in $CDCl_3$

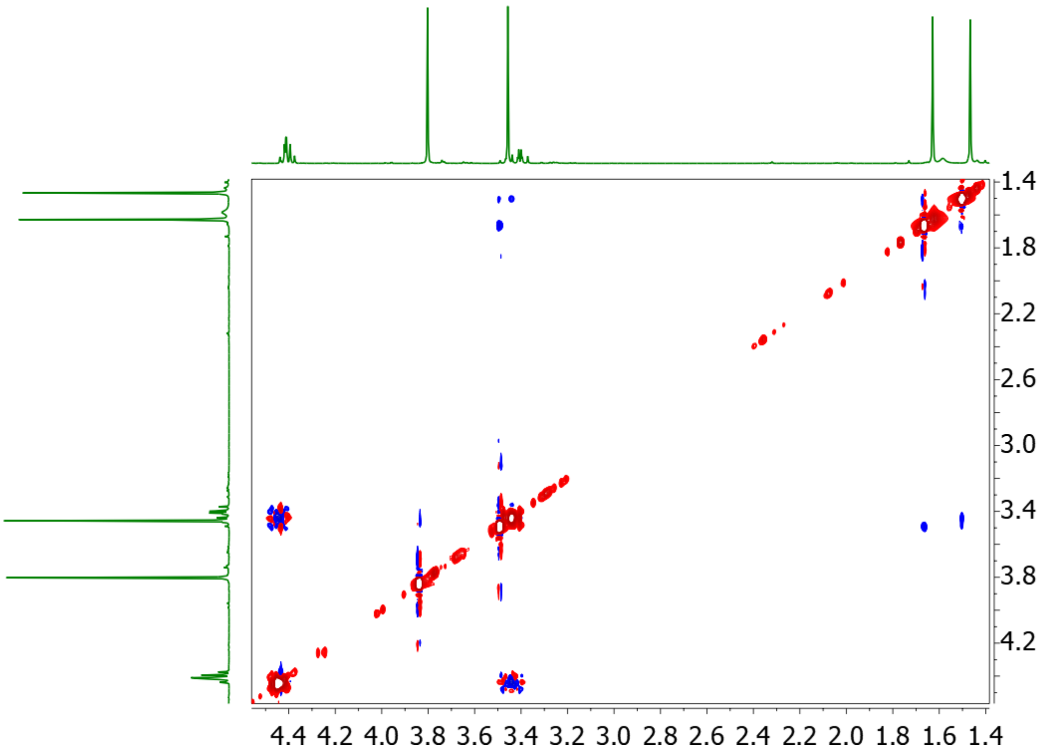

^{1}H NMR 400 MHz in $CDCl_3$

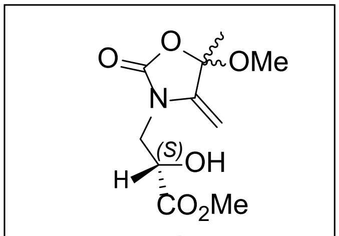

4

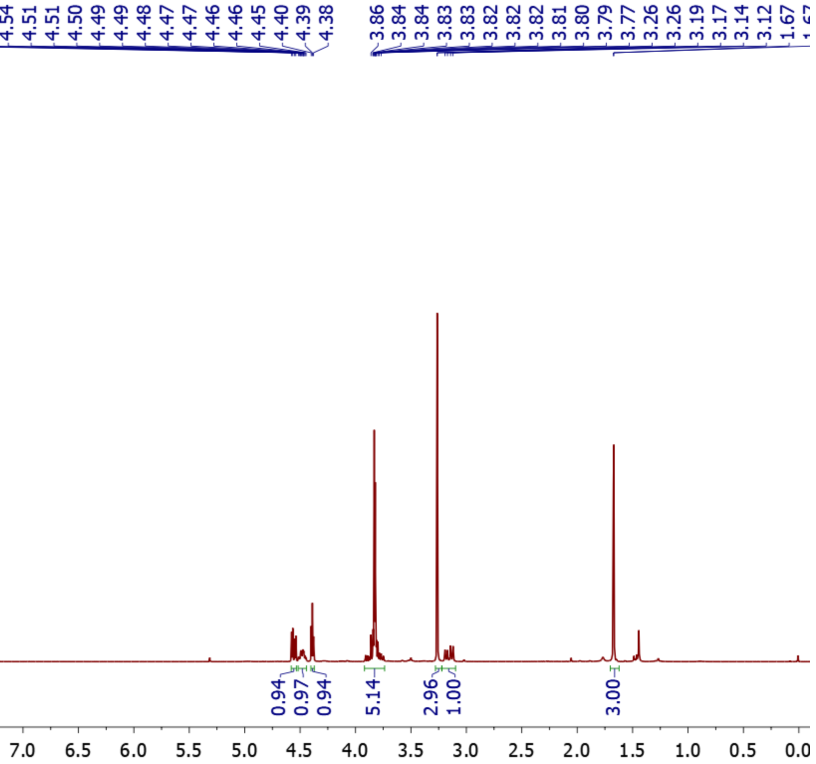

$^{13}C\{^{1}H\}$ NMR 100 MHz in $CDCl_3$

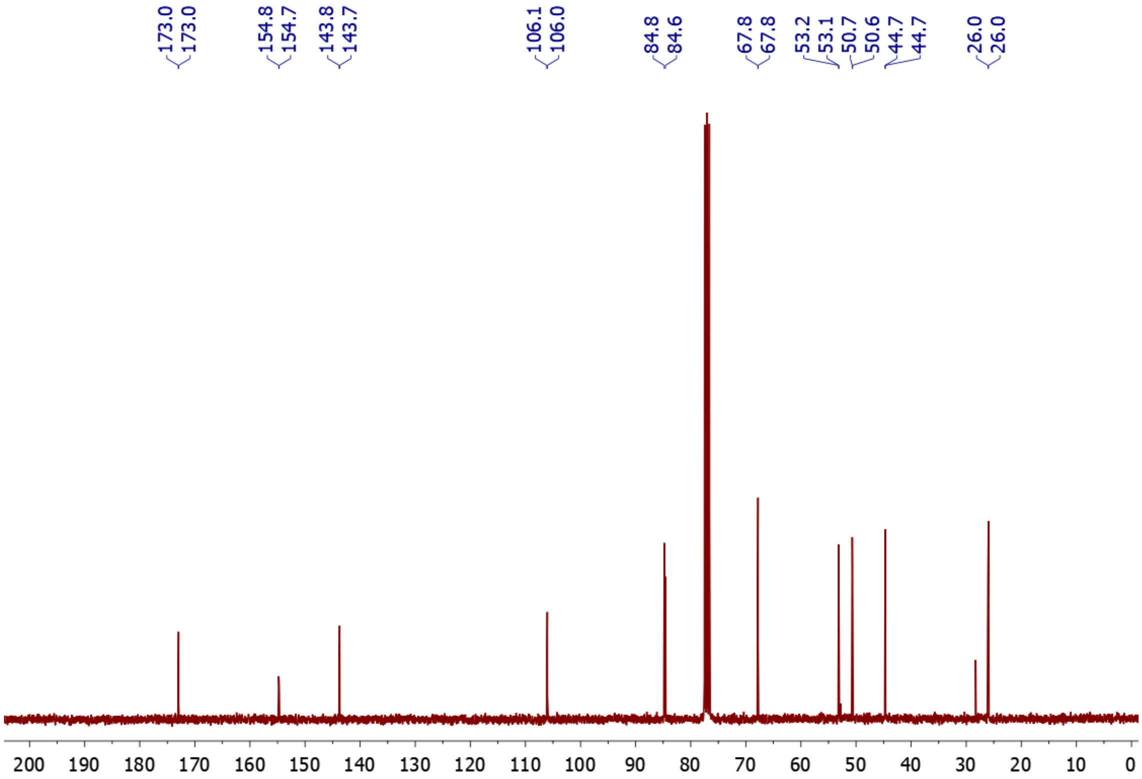

COSY in $CDCl_3$

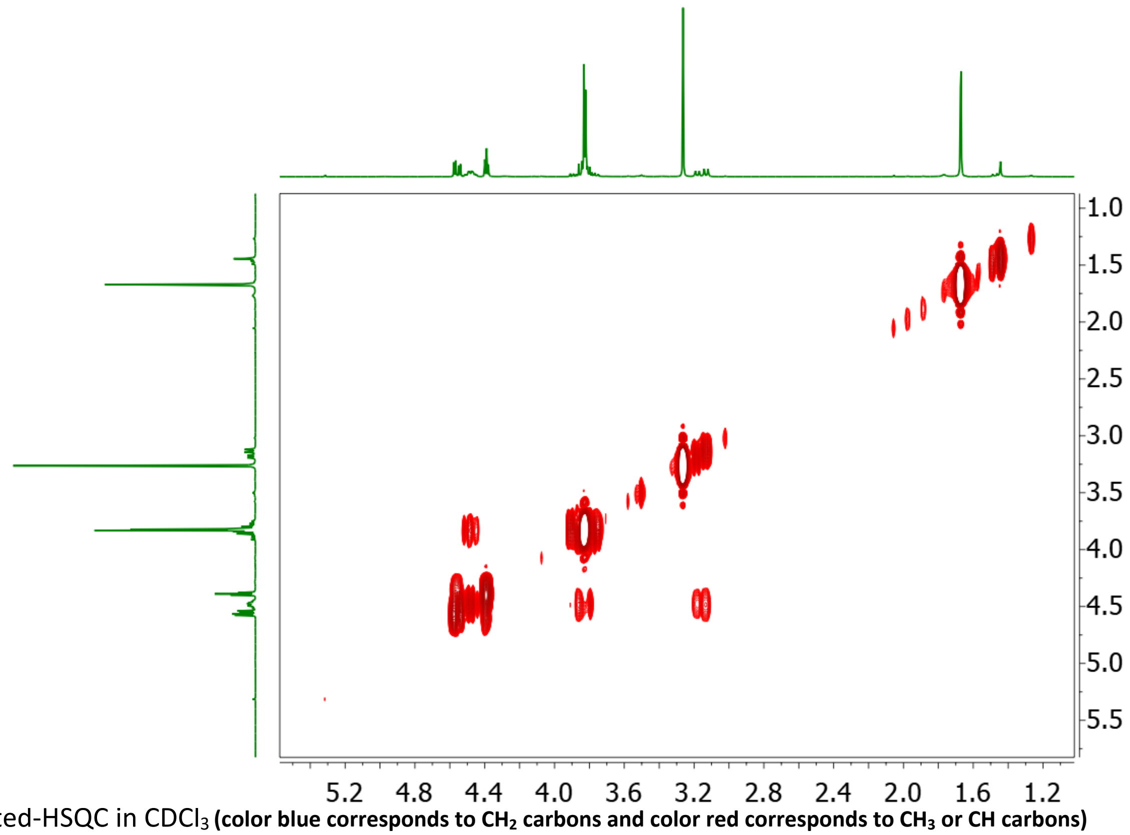

edited-HSQC in $CDCl_3$ **(color blue corresponds to CH_2 carbons and color red corresponds to CH_3 or CH carbons)**

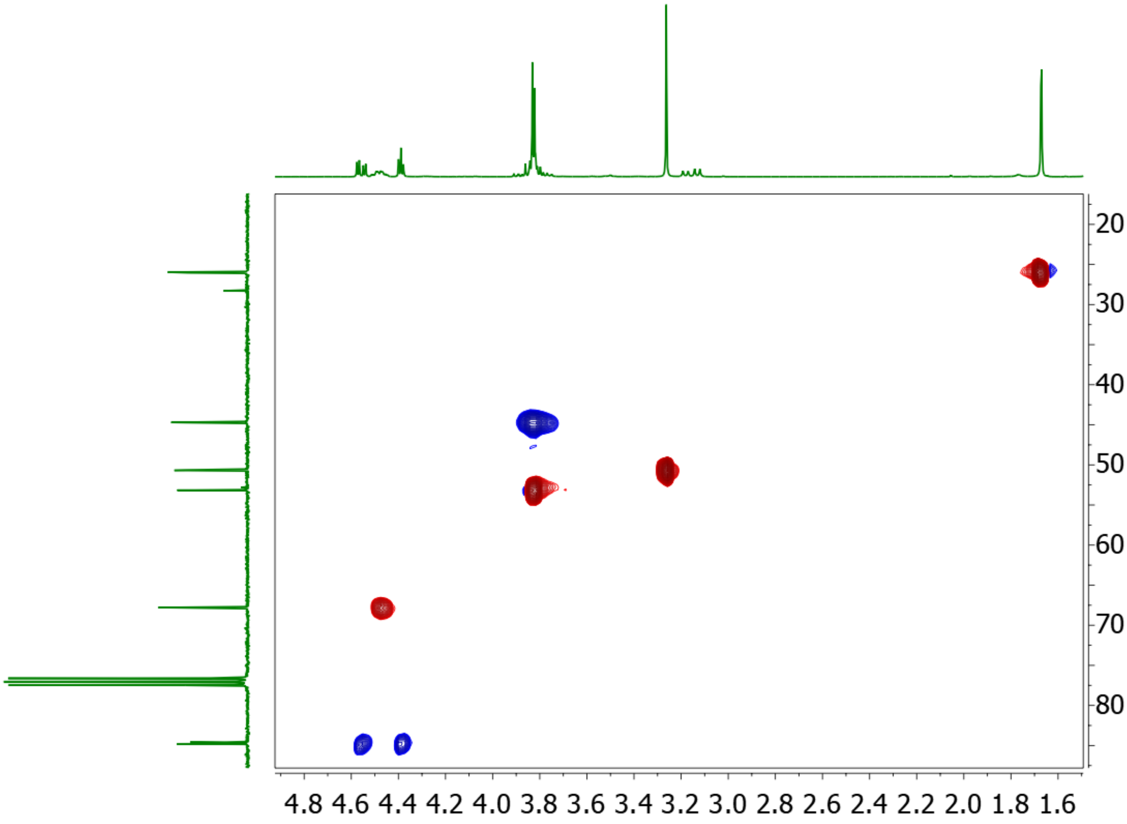

^{1}H NMR 400 MHz in $CDCl_3$

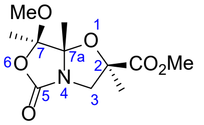

(5a)

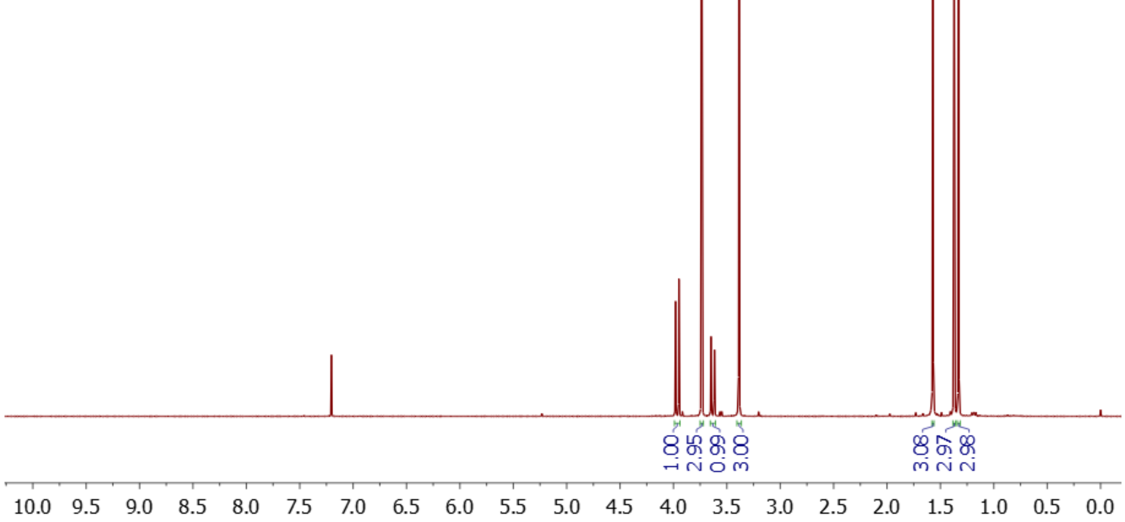

$^{13}C\{^{1}H\}$ NMR 100 MHz in $CDCl_3$

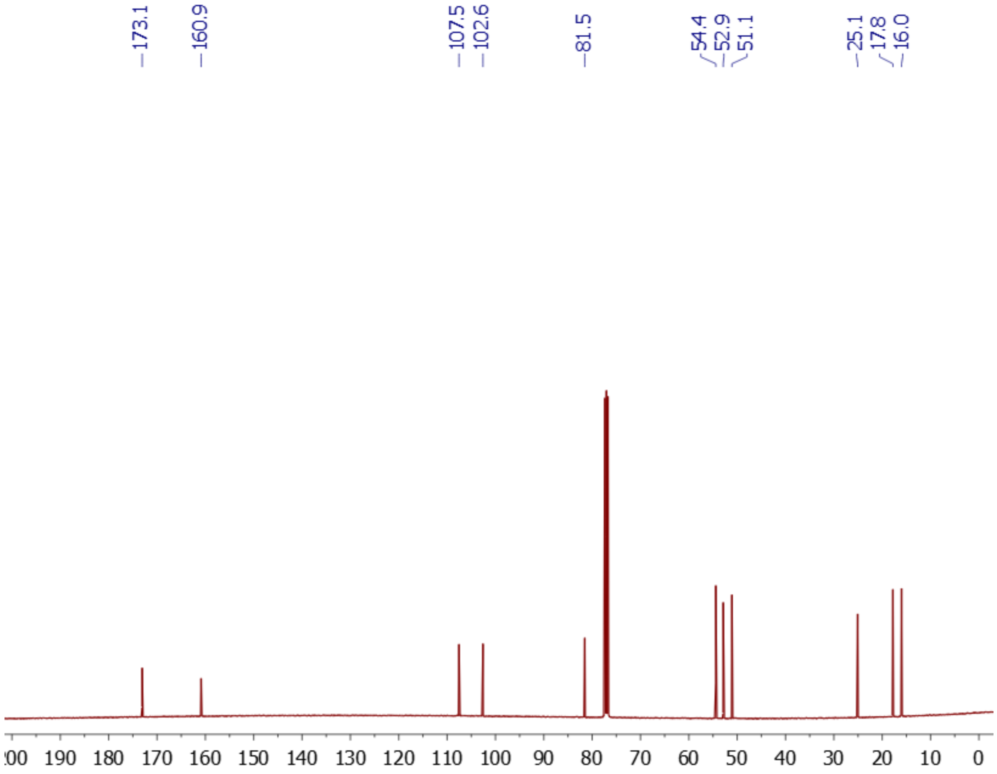

COSY in $CDCl_3$

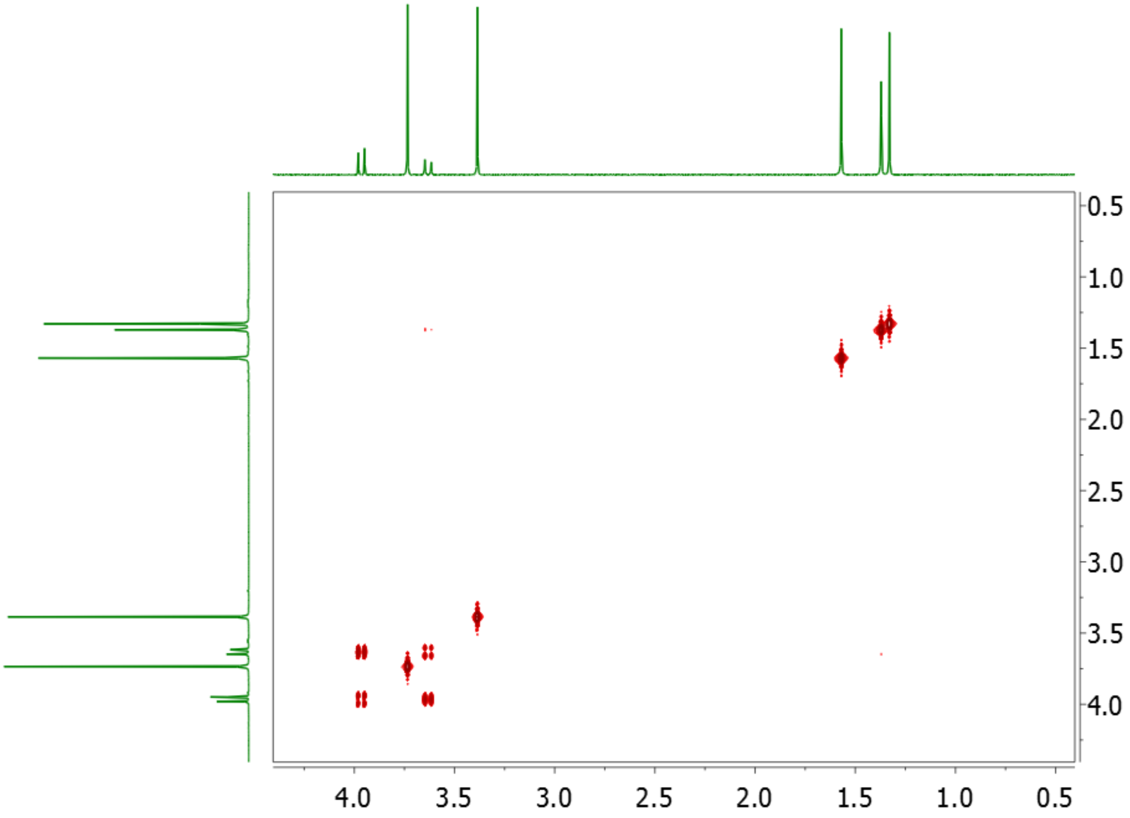

edited-HSQC in $CDCl_3$ **(color blue corresponds to CH_2 carbons and color red corresponds to CH_3 or CH carbons)**

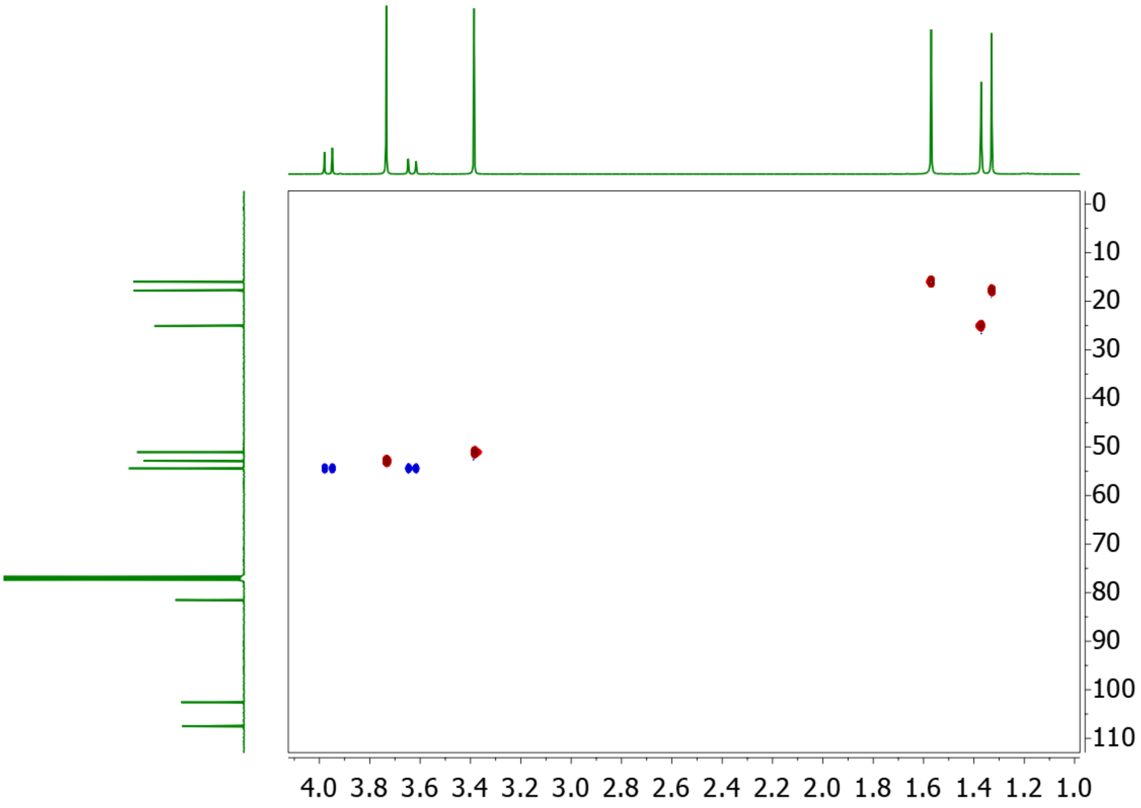

NOESY in $CDCl_3$

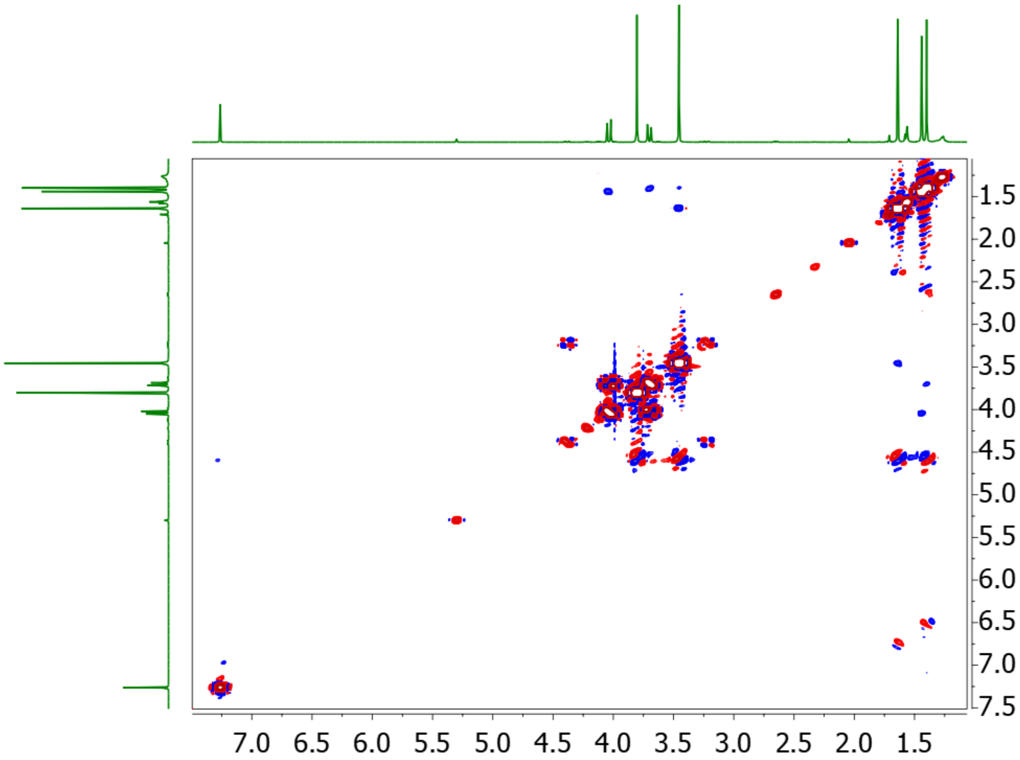

^{1}H NMR 400 MHz in $CDCl_3$

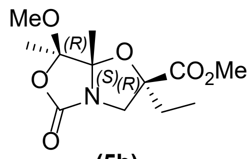

(5b)

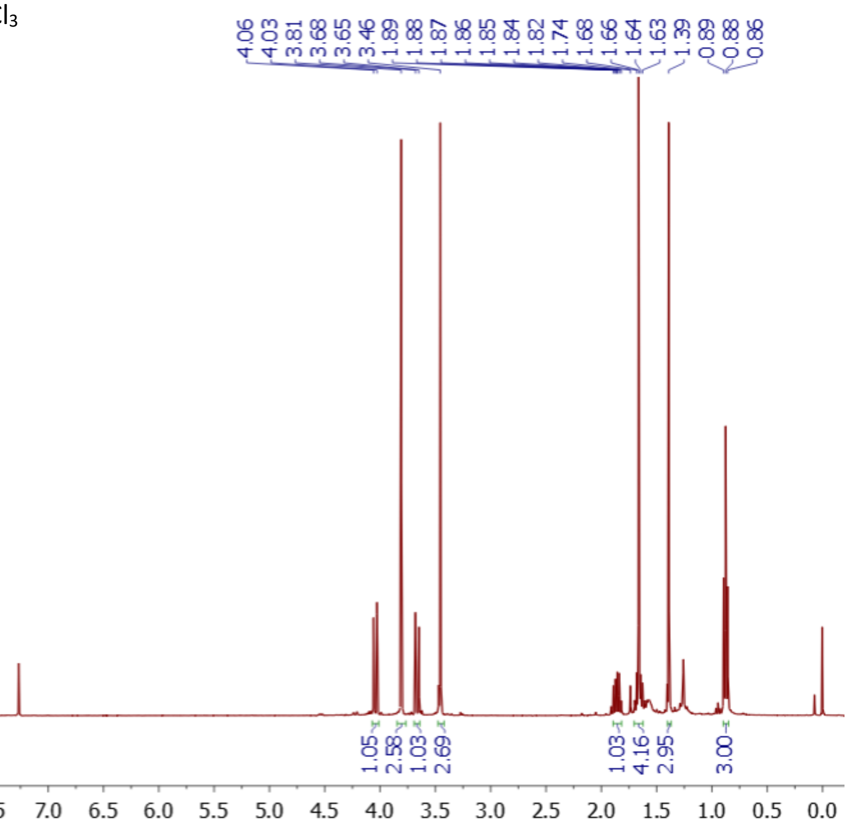

^{13}C{^{1}H} NMR 100 MHz in $CDCl_3$

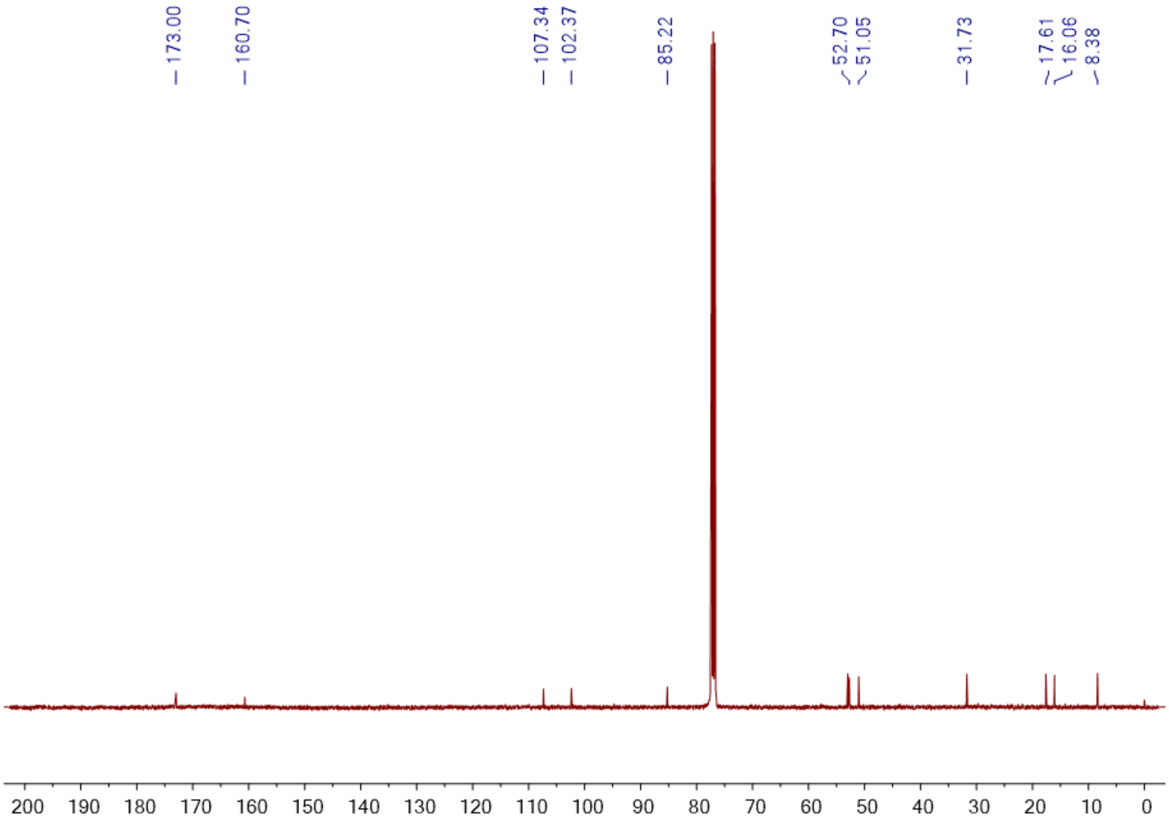

COSY in $CDCl_3$

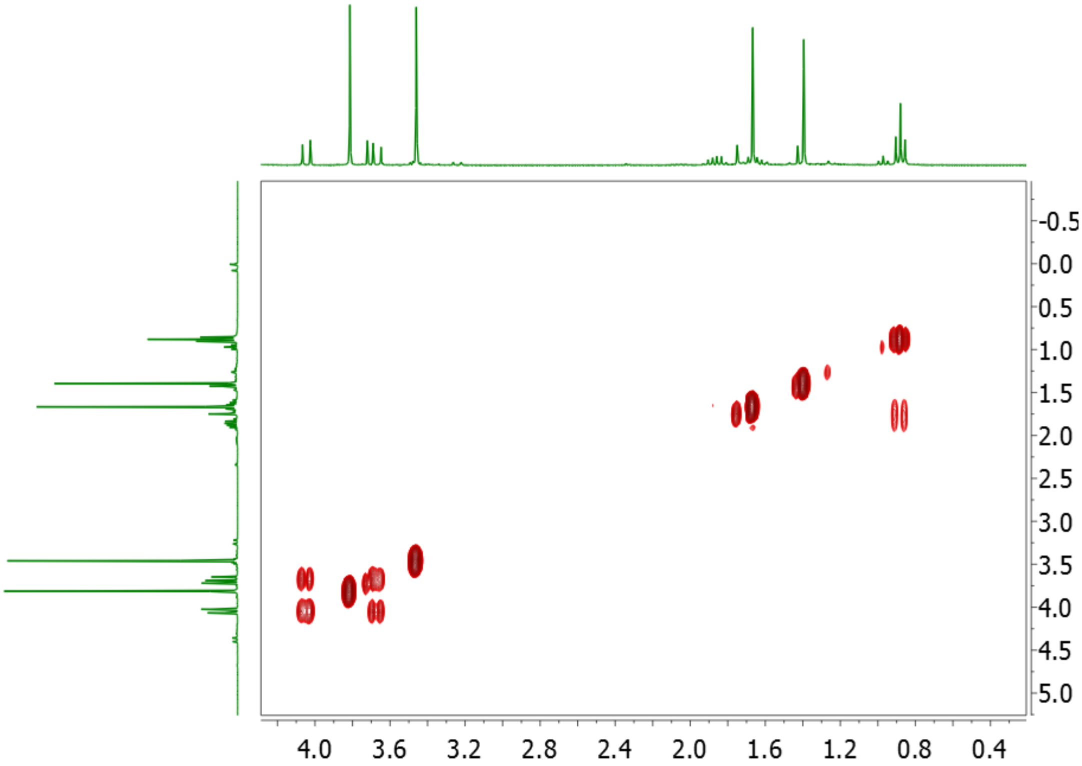

edited-HSQC in $CDCl_3$ **(color blue corresponds to CH_2 carbons and color red corresponds to CH_3 or CH carbons)**

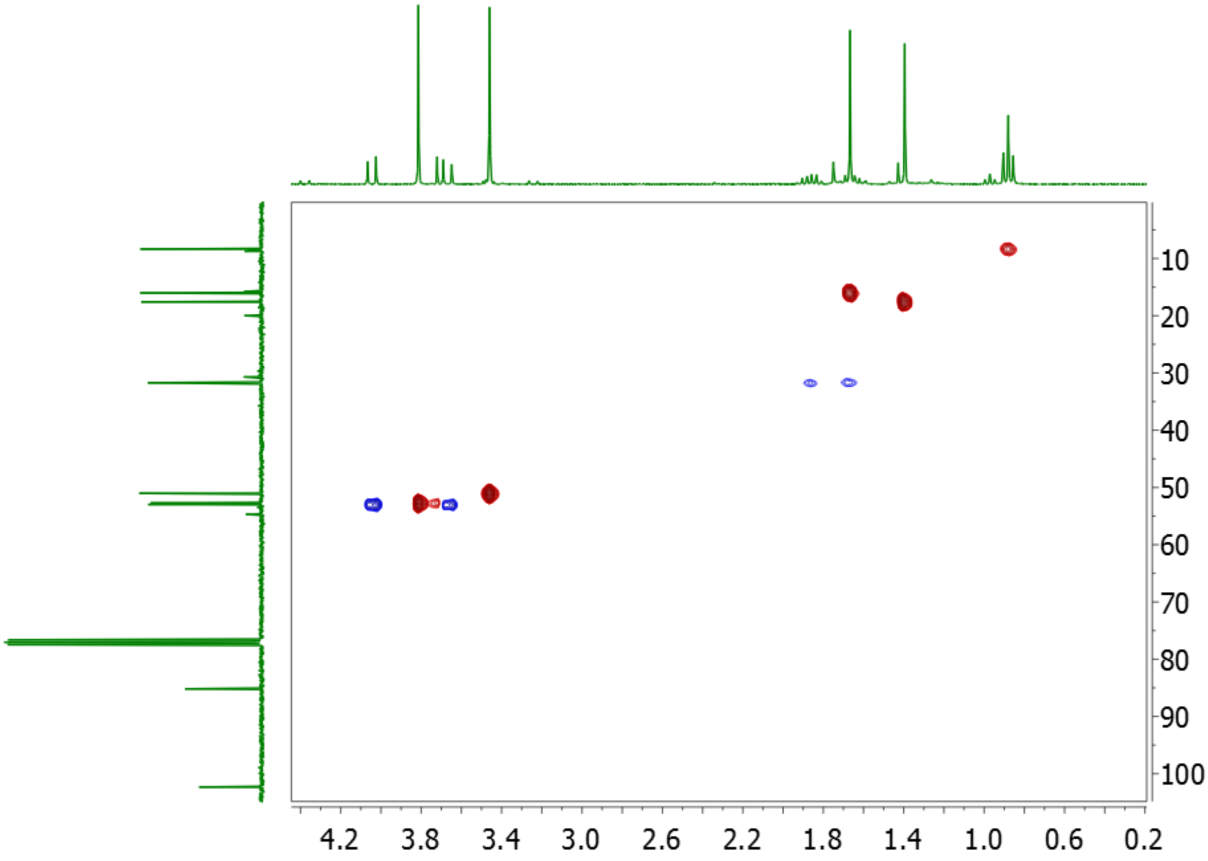

^{1}H NMR 400 MHz in $CDCl_3$

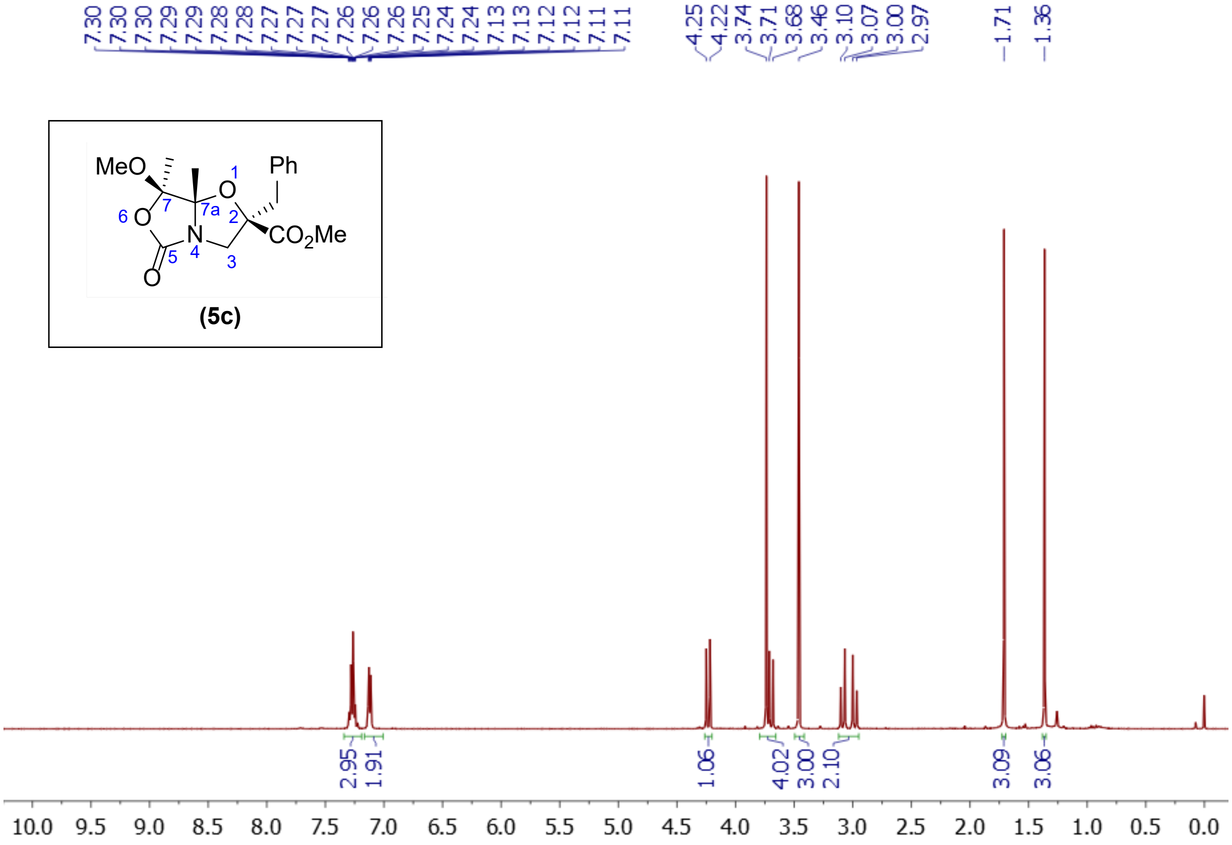

(5c)

$^{13}C\{^{1}H\}$ NMR 100 MHz in $CDCl_3$

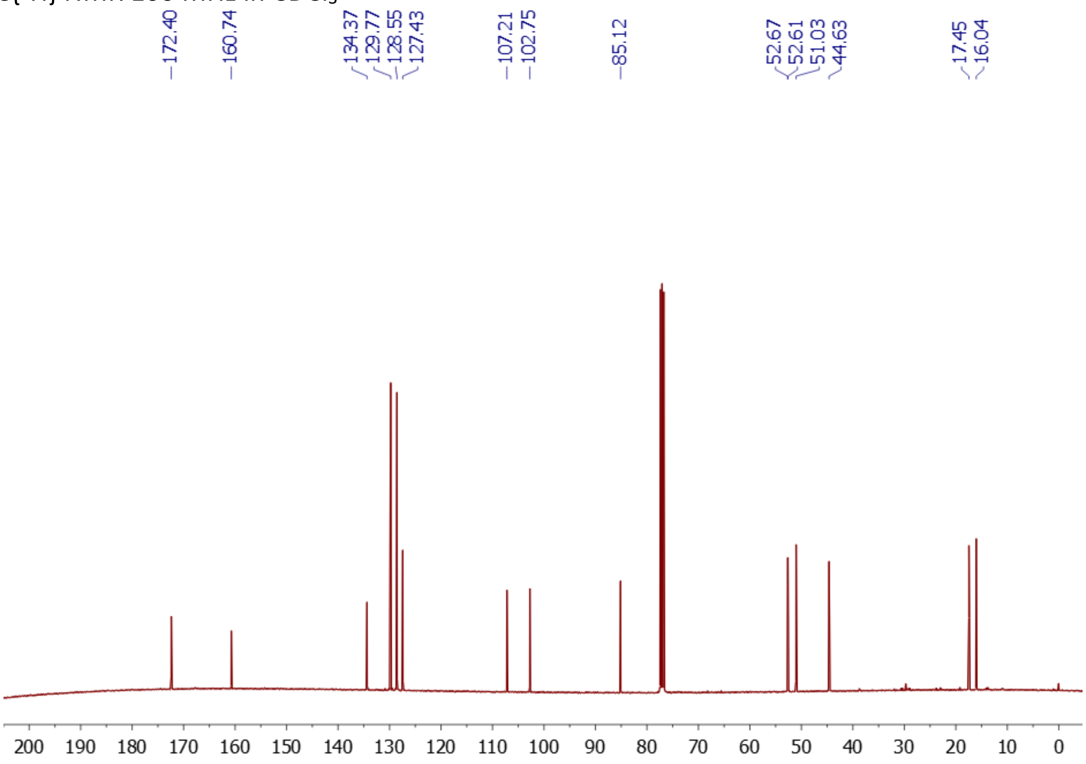

COSY in $CDCl_3$

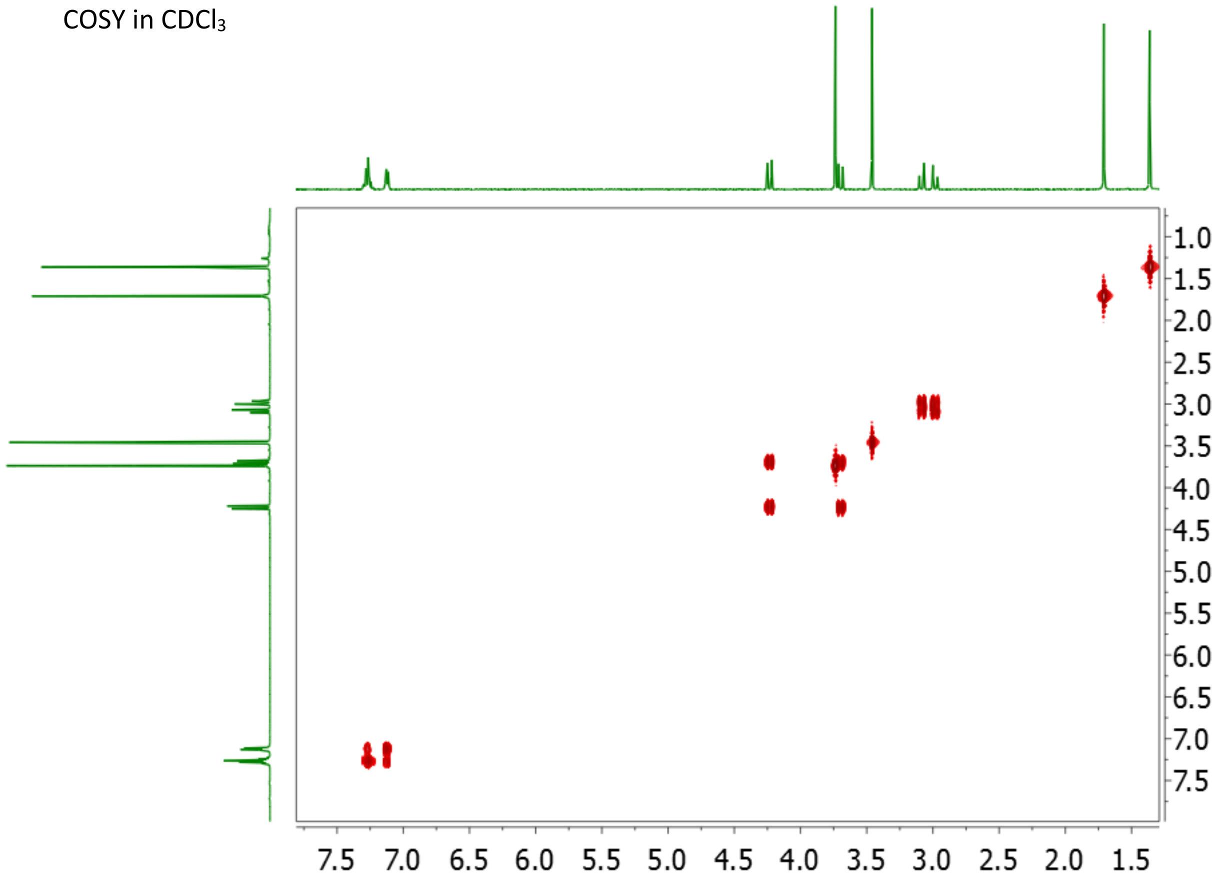

edited-HSQC in $CDCl_3$ **(color blue corresponds to CH_2 carbons and color red corresponds to CH_3 or CH carbons)**

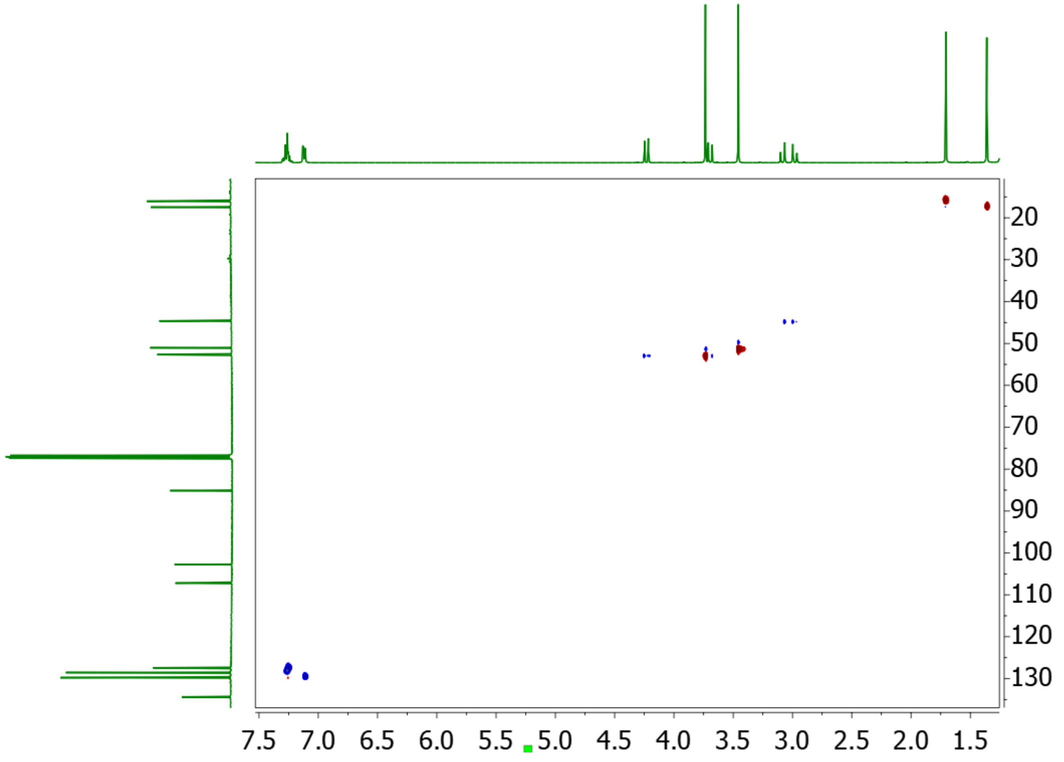

^{1}H NMR 400 MHz in $CDCl_3$ (enantiomer of **5c**)

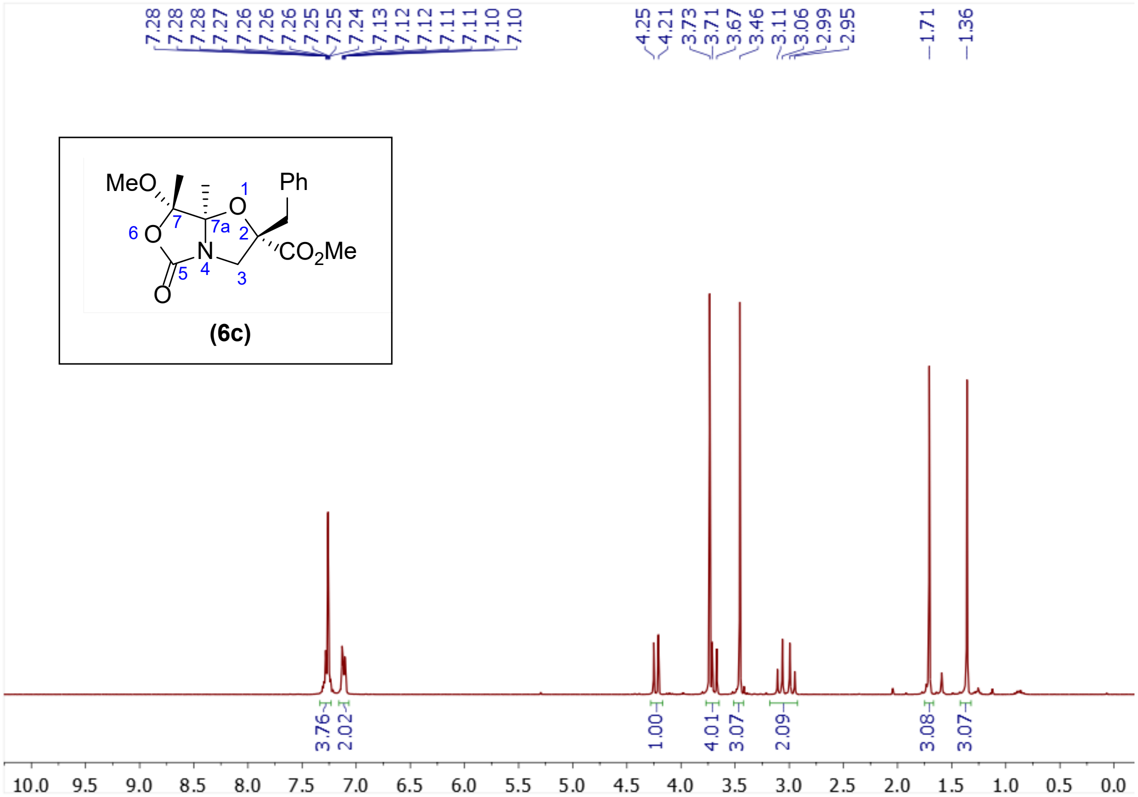

^1H NMR 400 MHz in $CDCl_3$

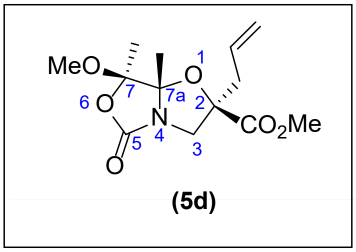

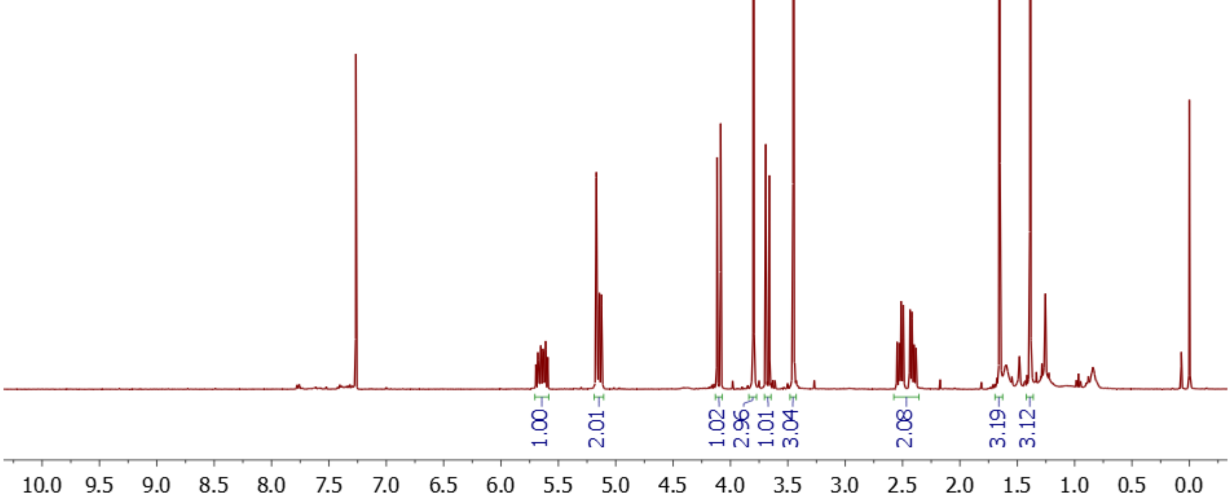

$^{13}C\{^1H\}$ NMR 100 MHz in $CDCl_3$

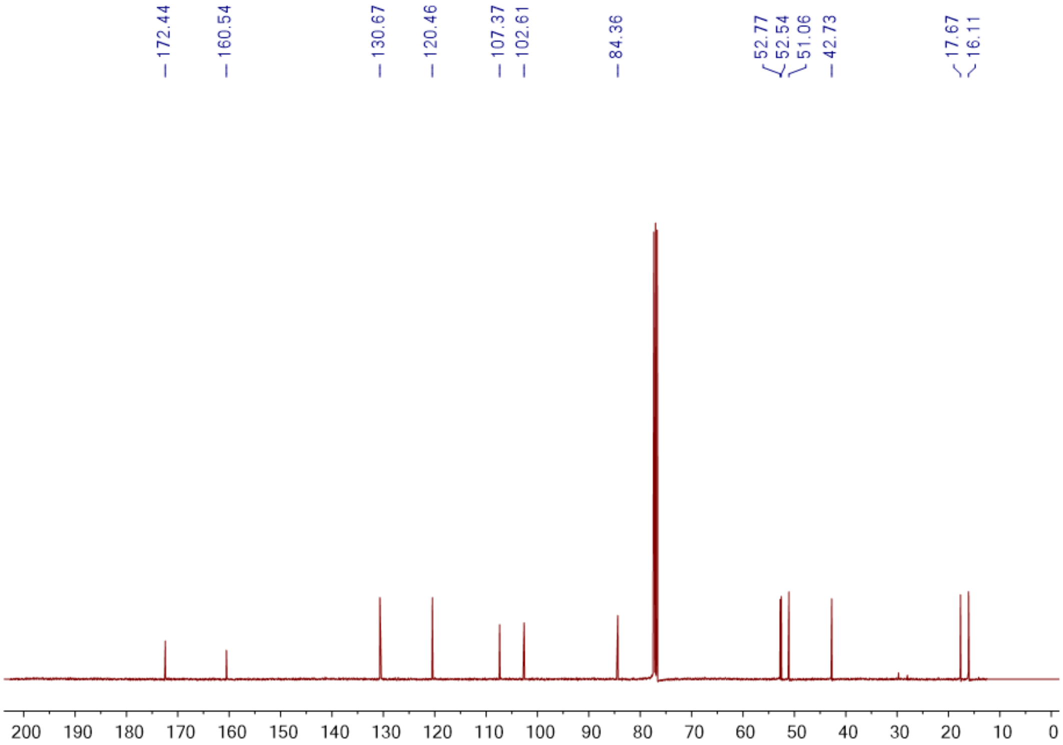

COSY in $CDCl_3$

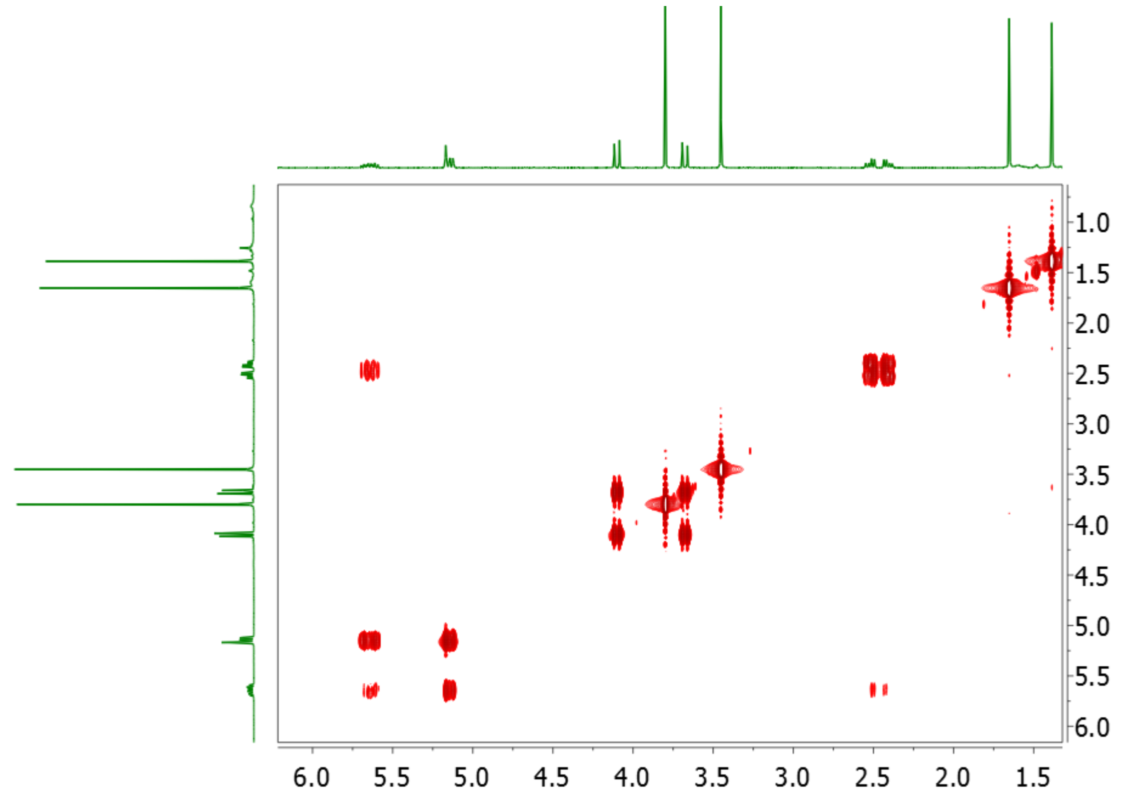

edited-HSQC in $CDCl_3$ **(color blue corresponds to CH_2 carbons and color red corresponds to CH_3 or CH carbons)**

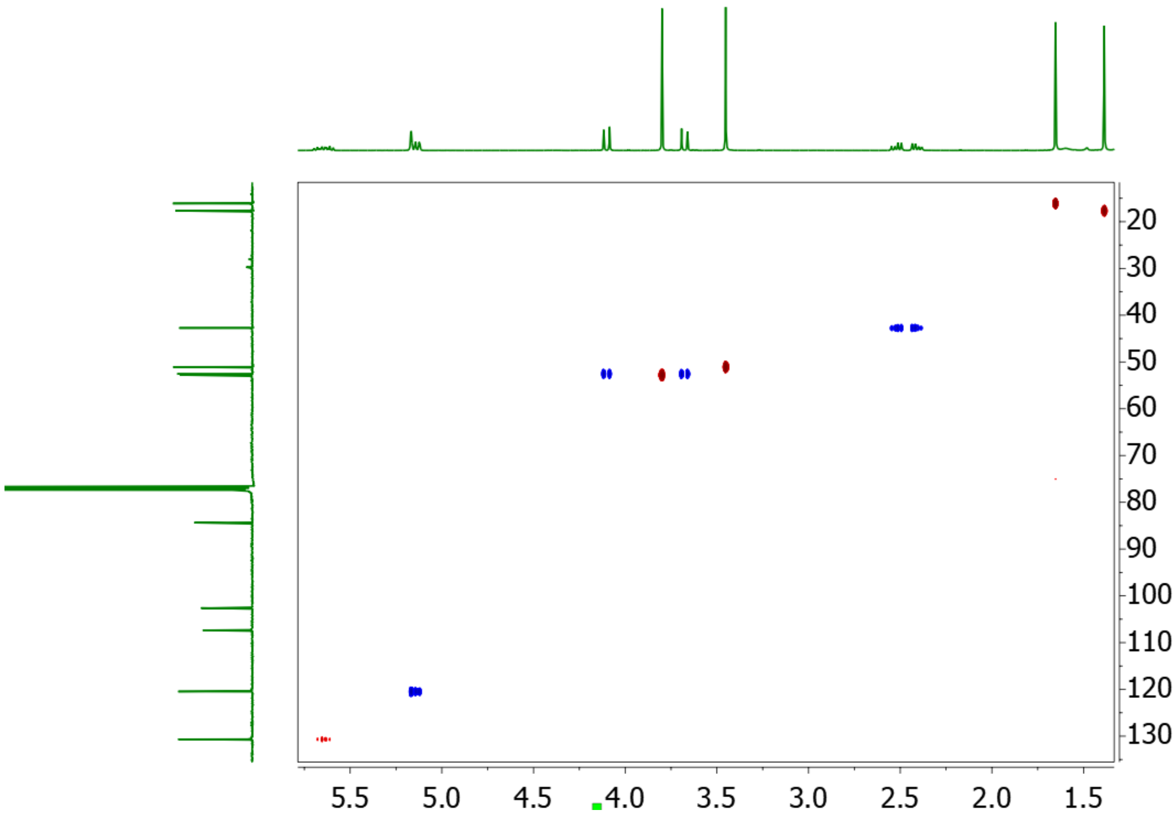

^{1}H NMR 400 MHz in $CDCl_3$

Compound **6a** is the enantiomer of **5a** and its spectral data are in good agreement

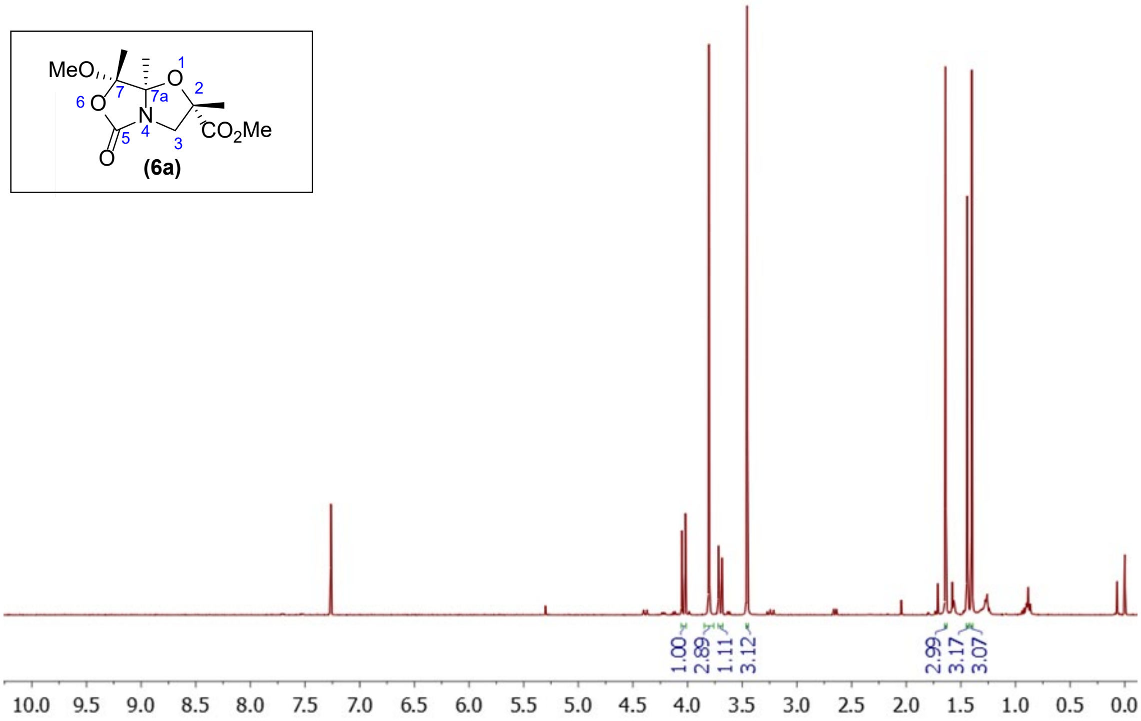

^{1}H NMR 400 MHz in $CDCl_3$

Compound **7a** (α–Me*iso*Ser) is the enantiomer of **8a** and their spectral data are in good agreement and match with previously published data (reference 24 in the manuscript).

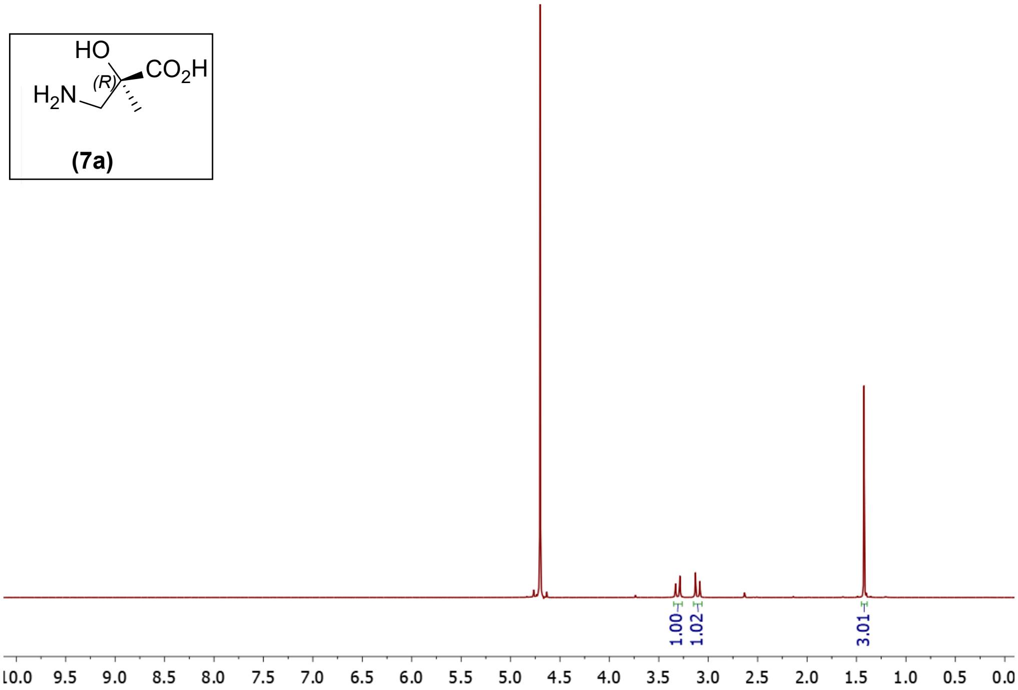

^{1}H NMR 400 MHz in D_2O

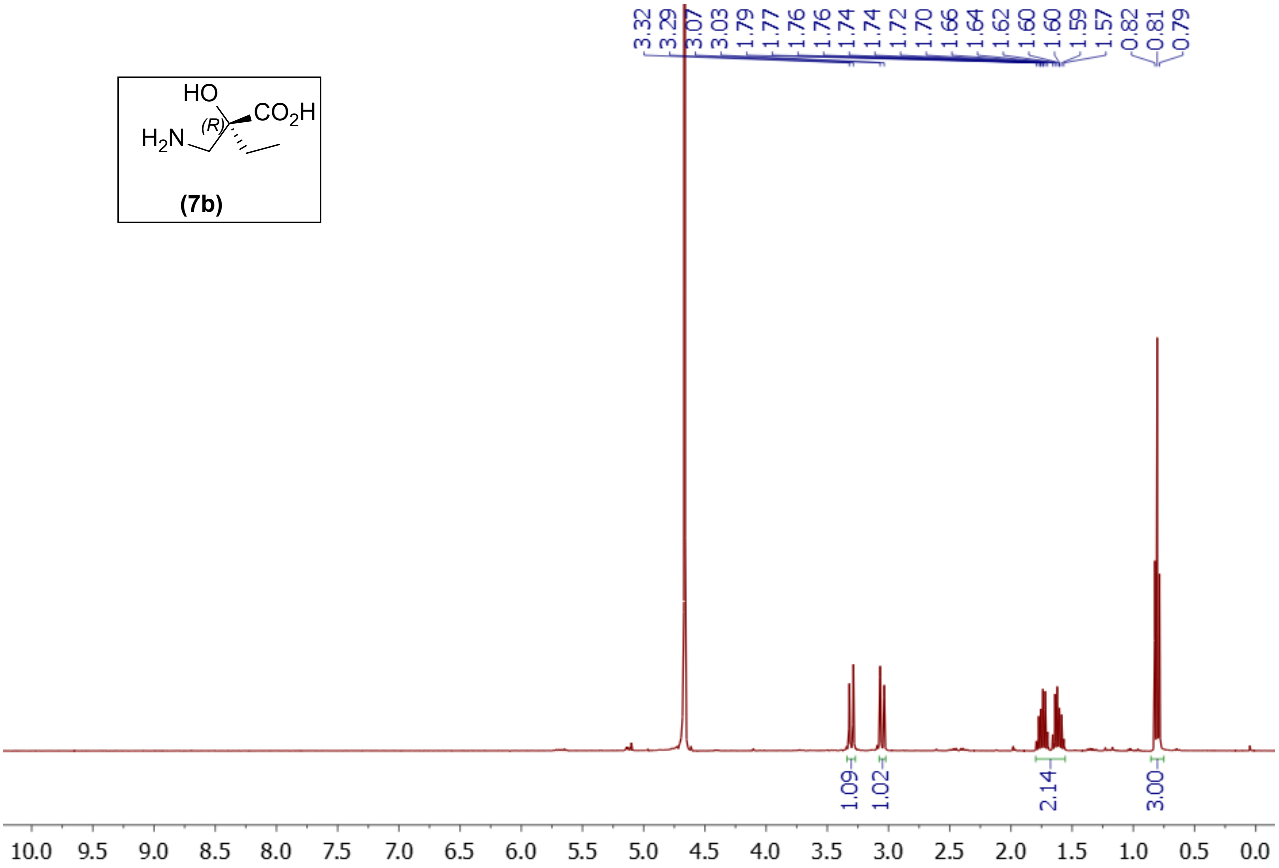

$^{13}C\{^{1}H\}$ NMR 100 MHz in D_2O

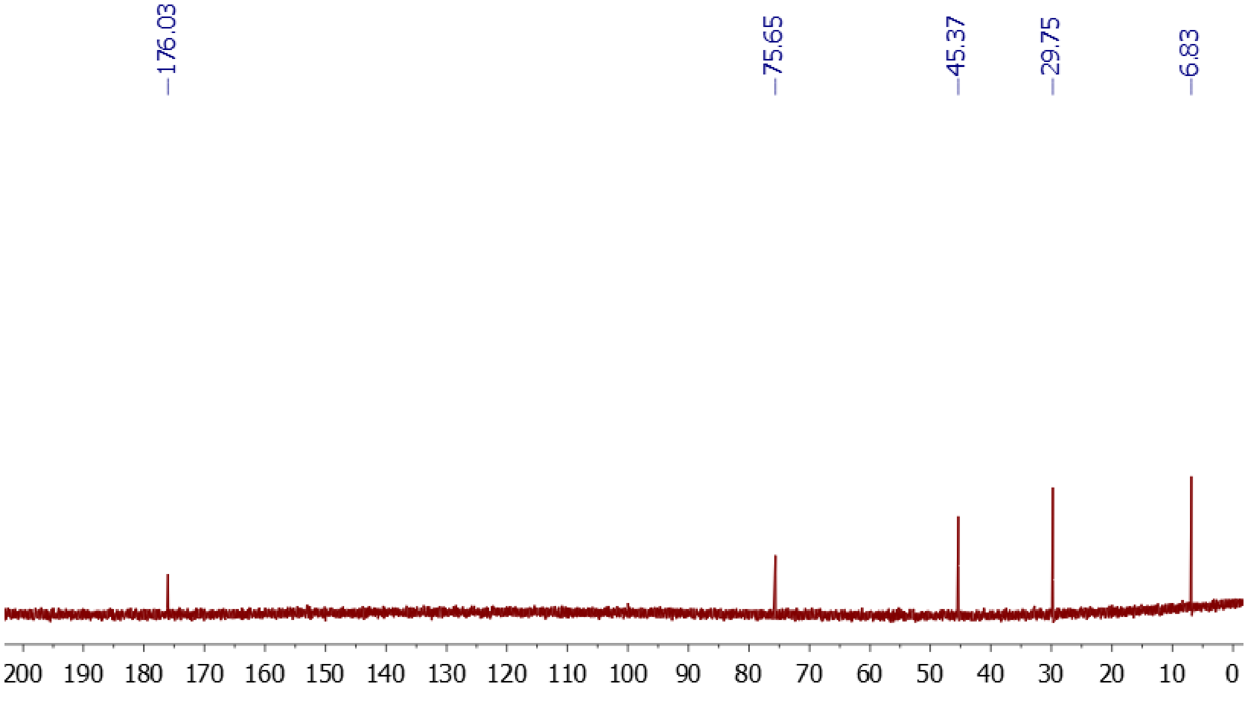

COSY in D_2O

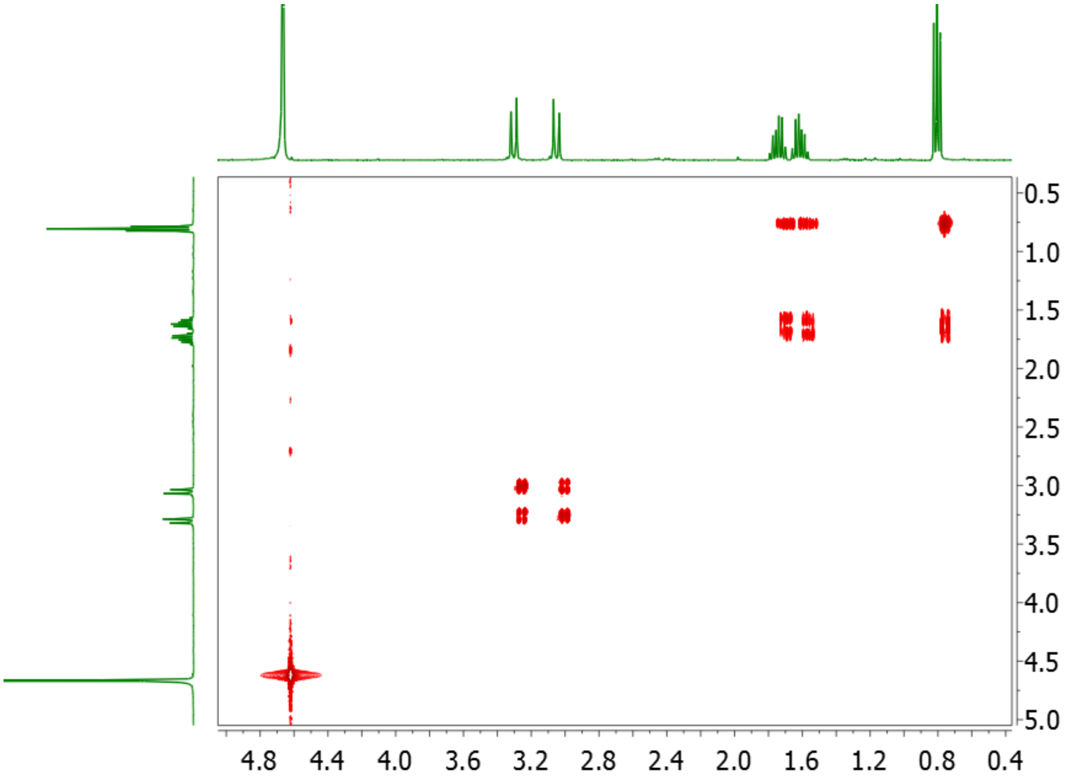

edited-HSQC in D_2O **(color blue corresponds to CH_2 carbons and color red corresponds to CH_3 or CH carbons)**

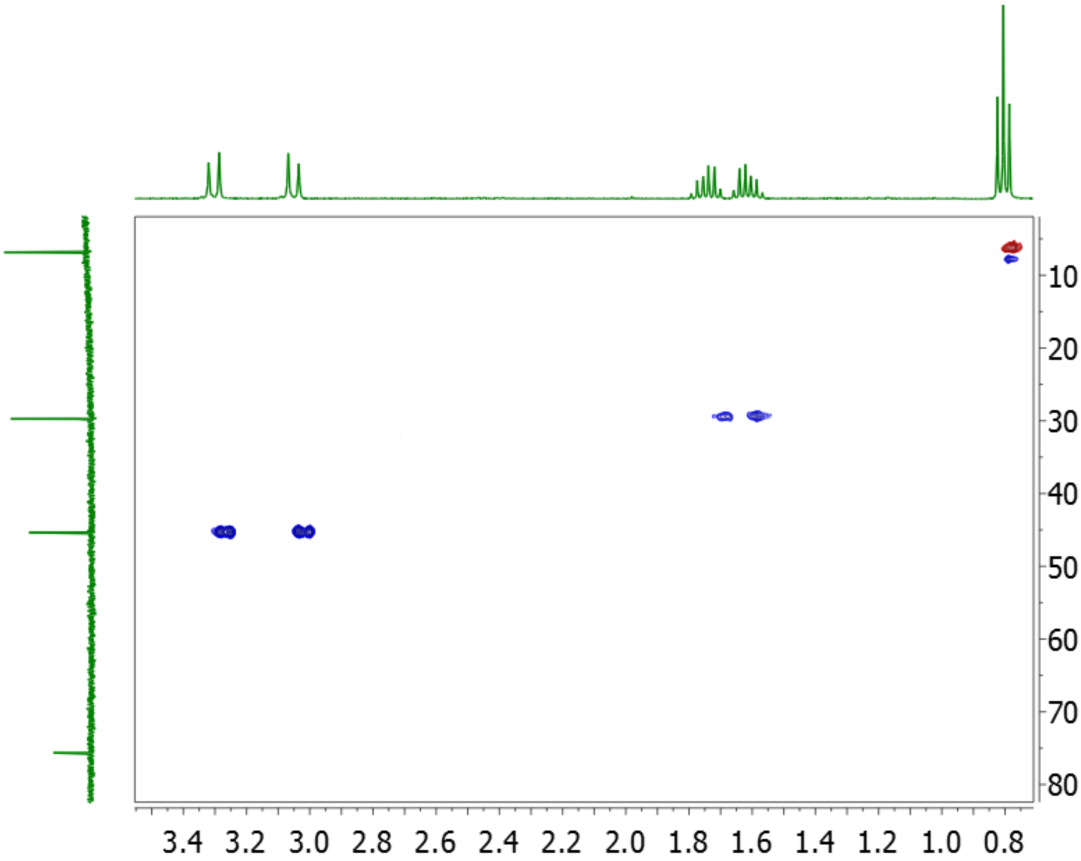

^{1}H NMR 400 MHz in D_2O

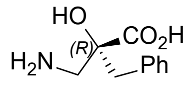

(7c)

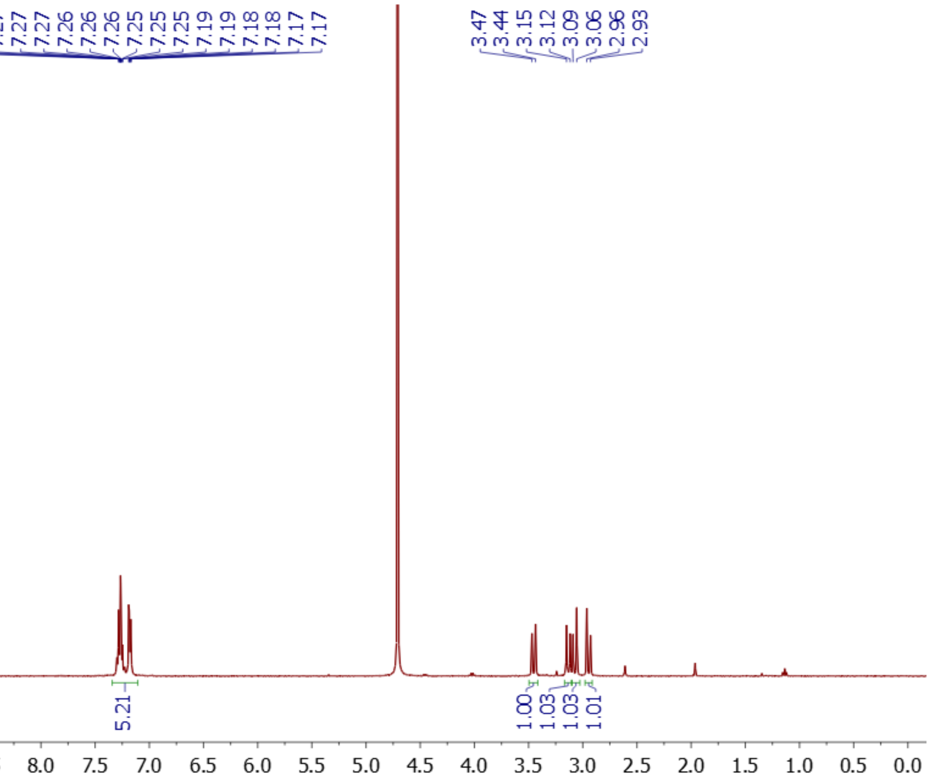

$^{13}C\{^{1}H\}$ NMR 100 MHz in D_2O

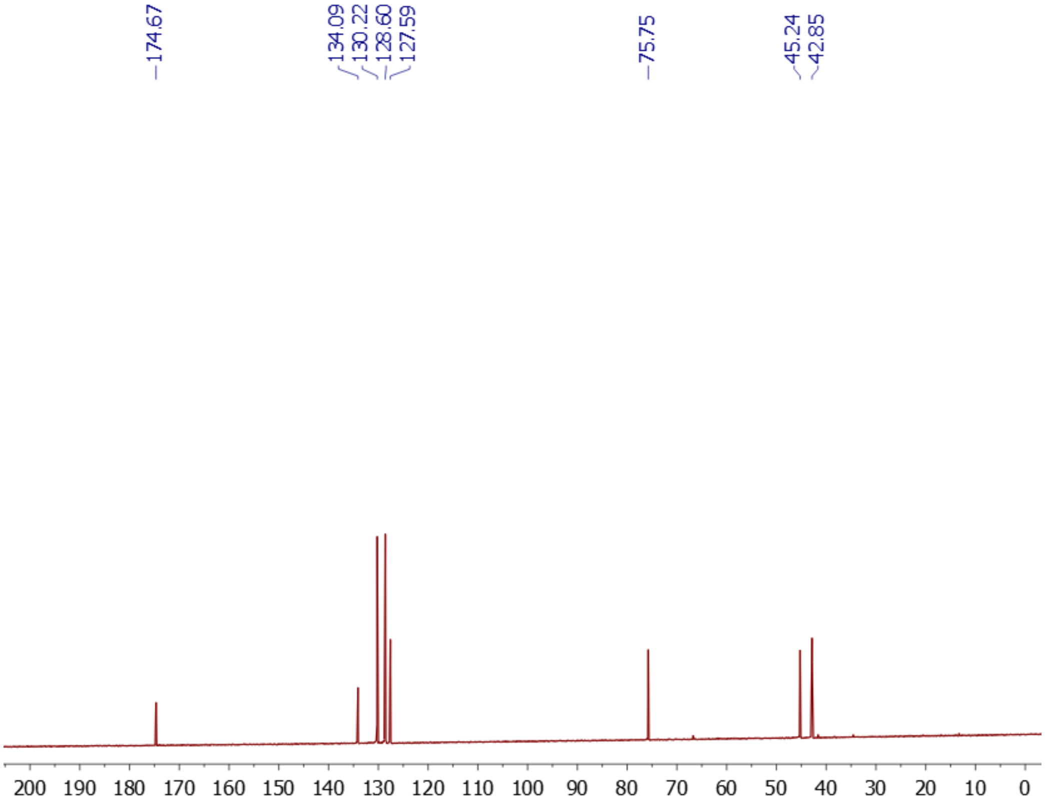

COSY in D_2O

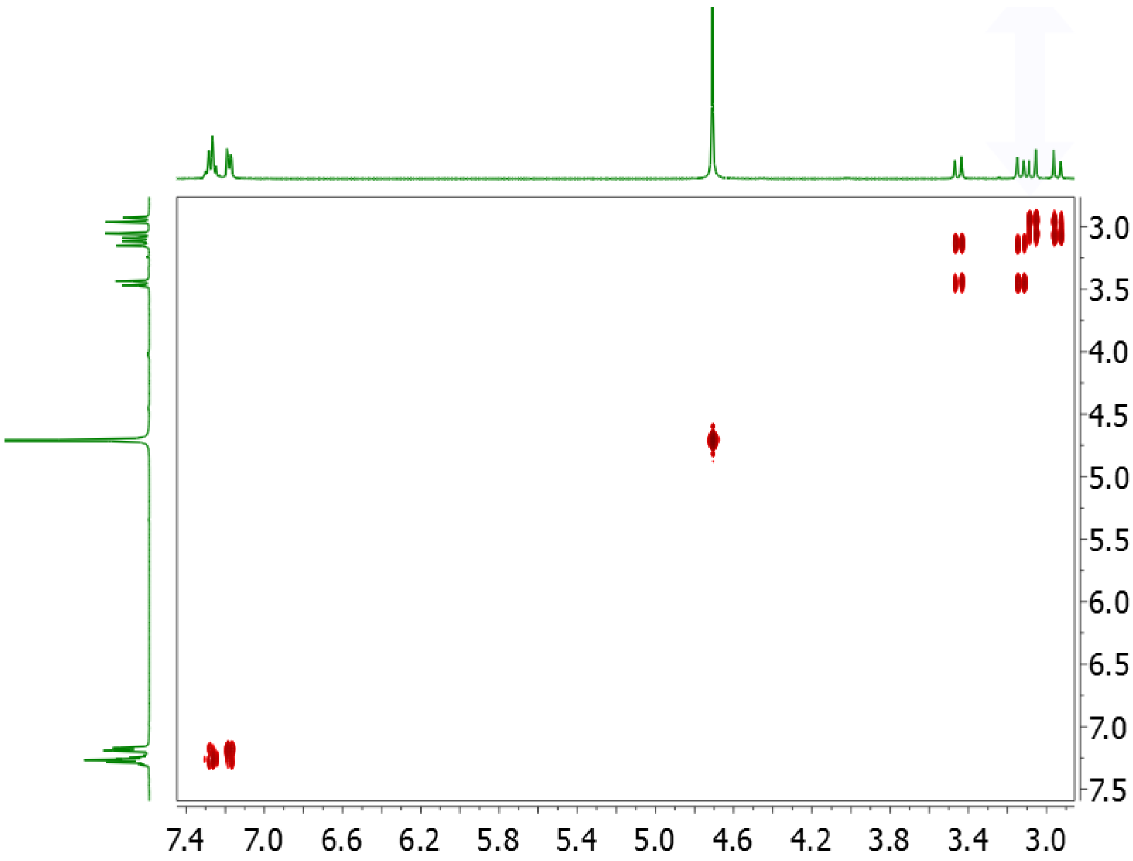

edited-HSQC in D_2O **(color blue corresponds to CH_2 carbons and color red corresponds to CH_3 or CH carbons)**

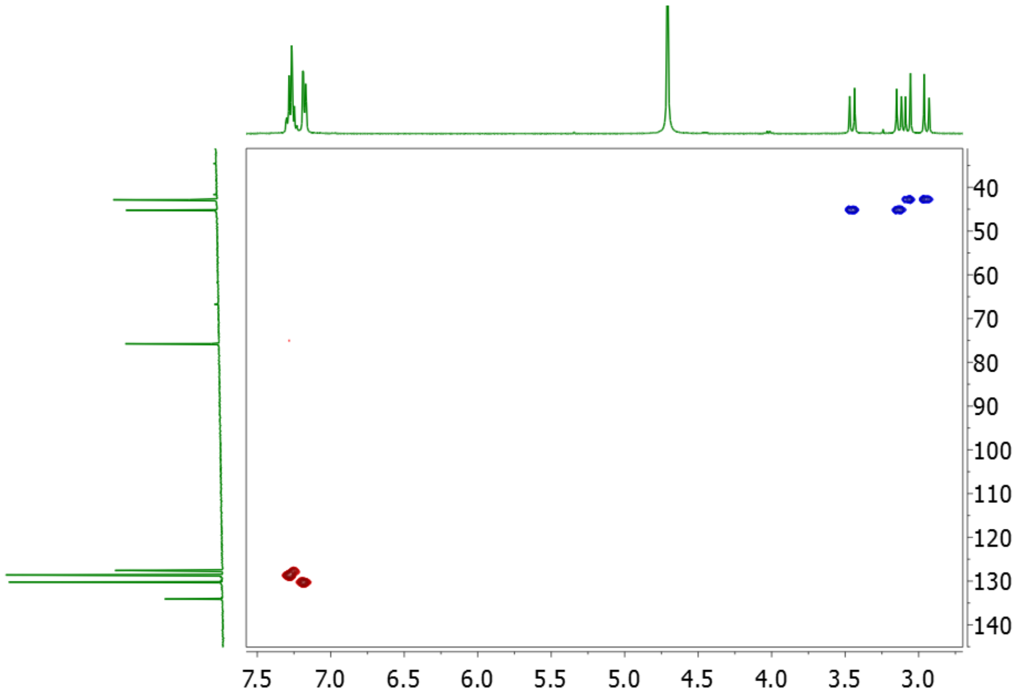

^{1}H NMR 400 MHz in D_2O (enantiomer of **7c**)

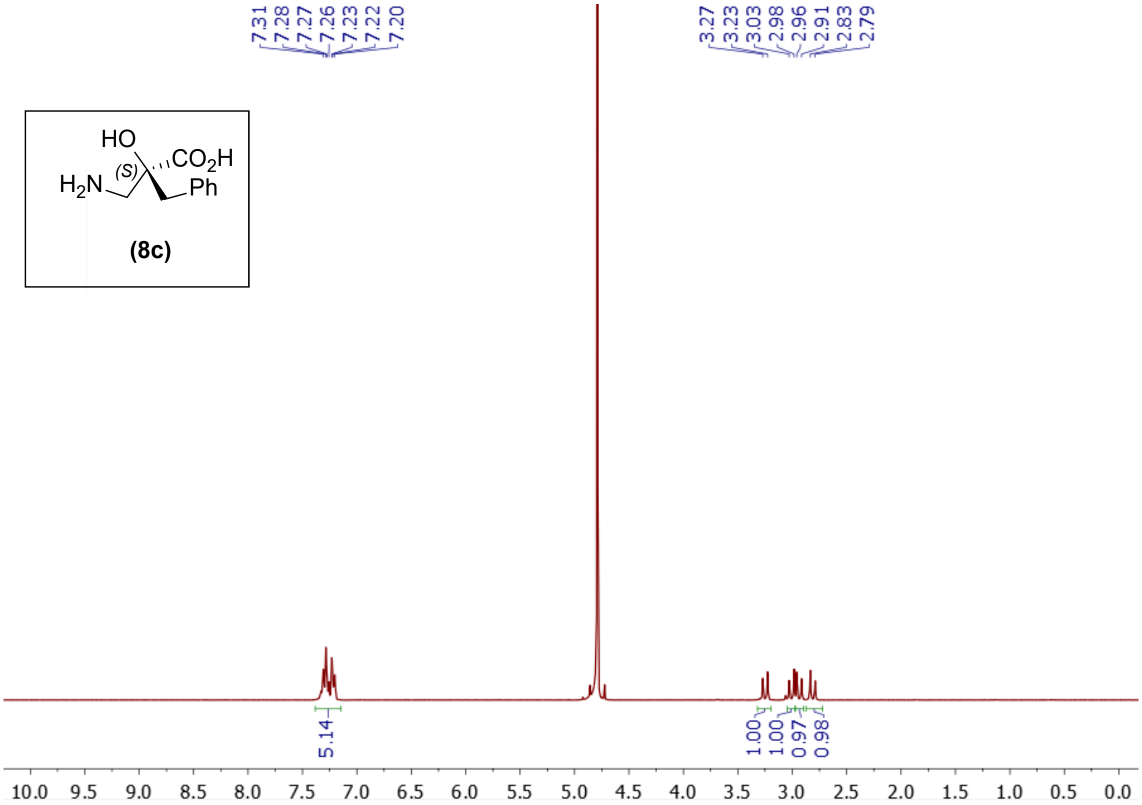

^{1}H NMR 400 MHz in $CDCl_3$

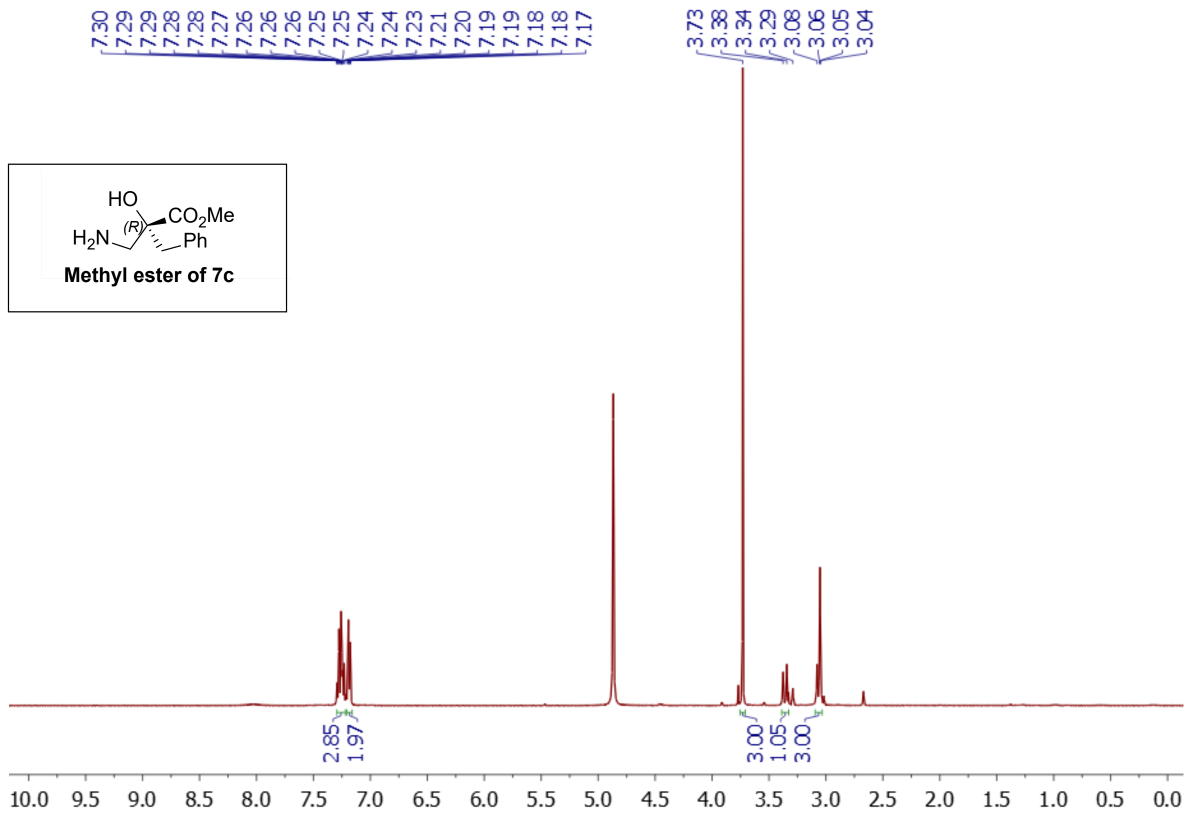

$^{13}C\{^{1}H\}$ NMR 100 MHz in CD_3Cl

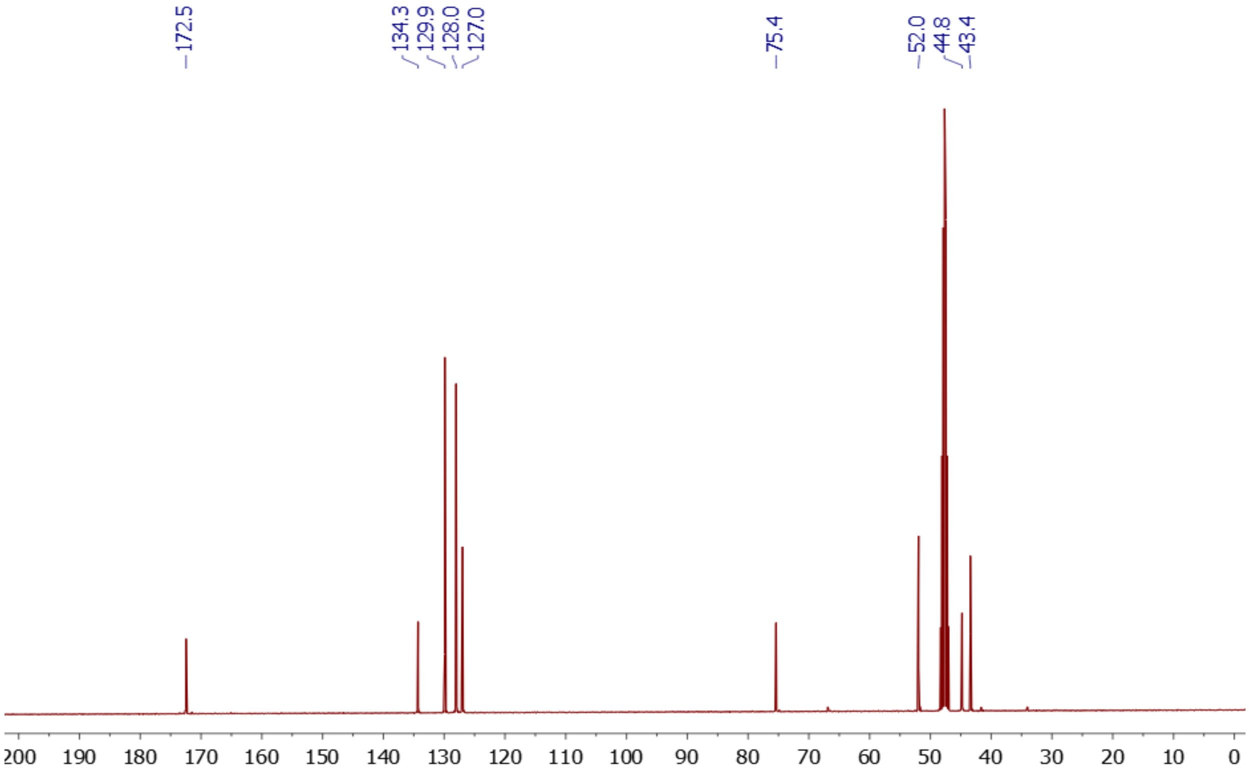

COSY in CD_3Cl

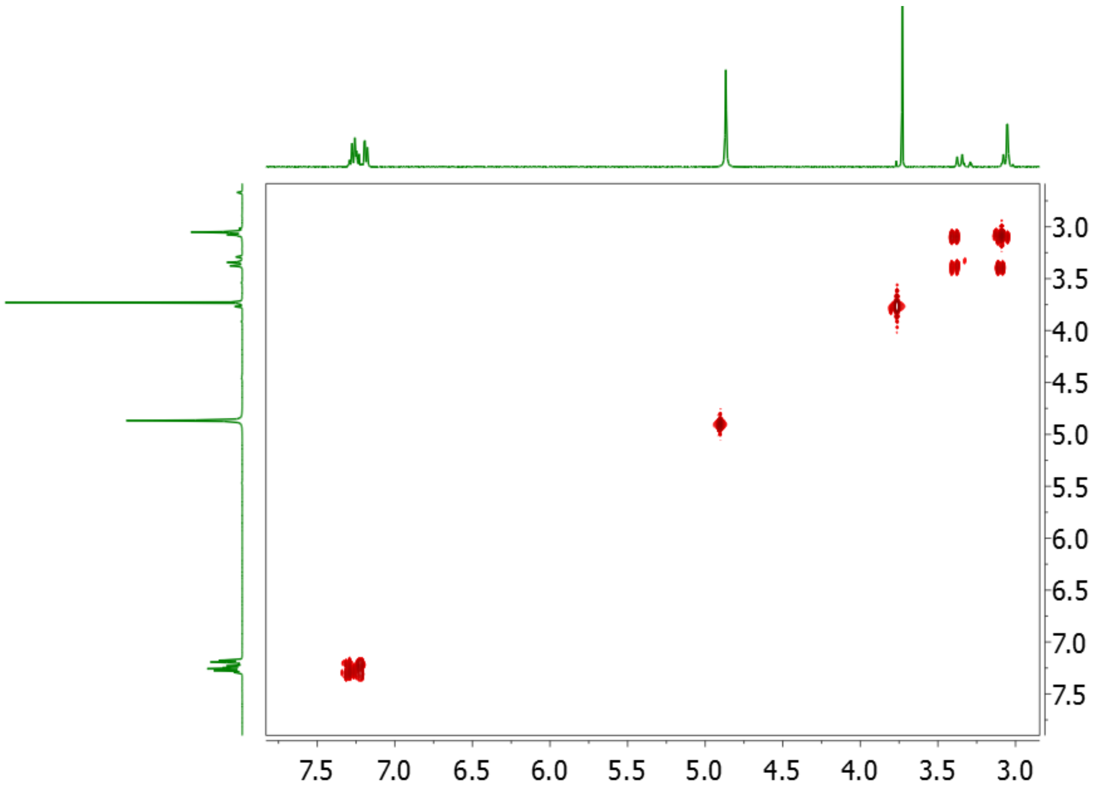

edited-HSQC in $CDCl_3$ **(color blue corresponds to CH_2 carbons and color red corresponds to CH_3 or CH carbons)**

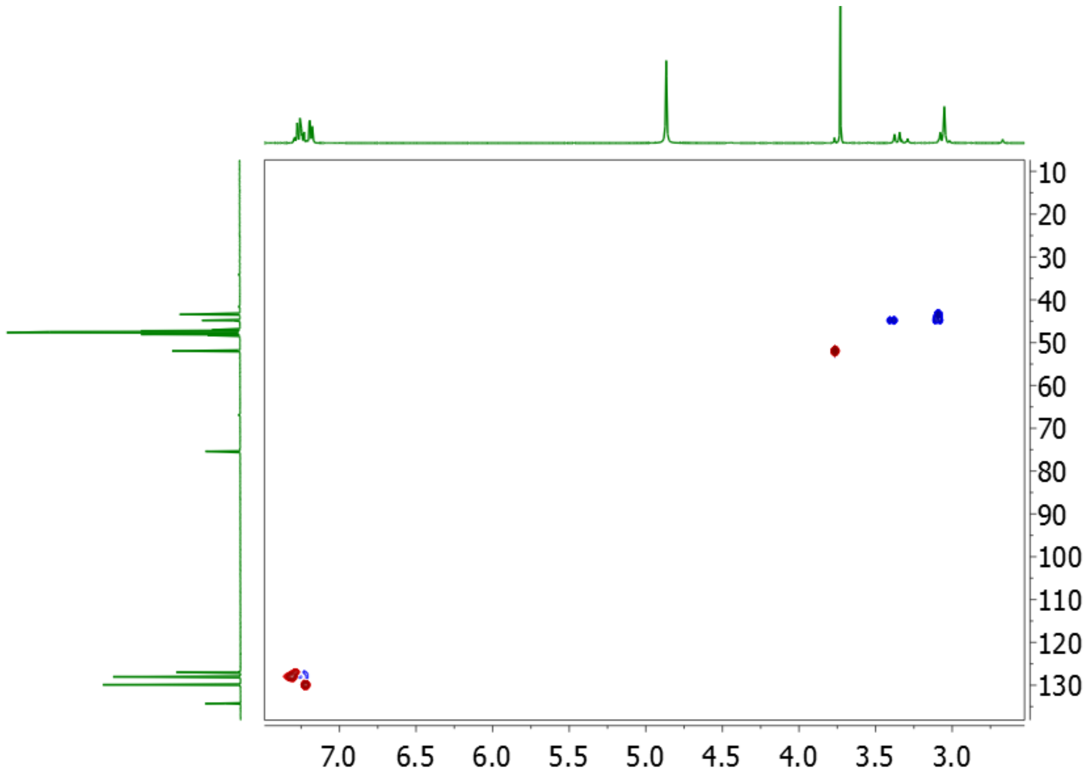

^1H NMR 400 MHz in CD_3Cl

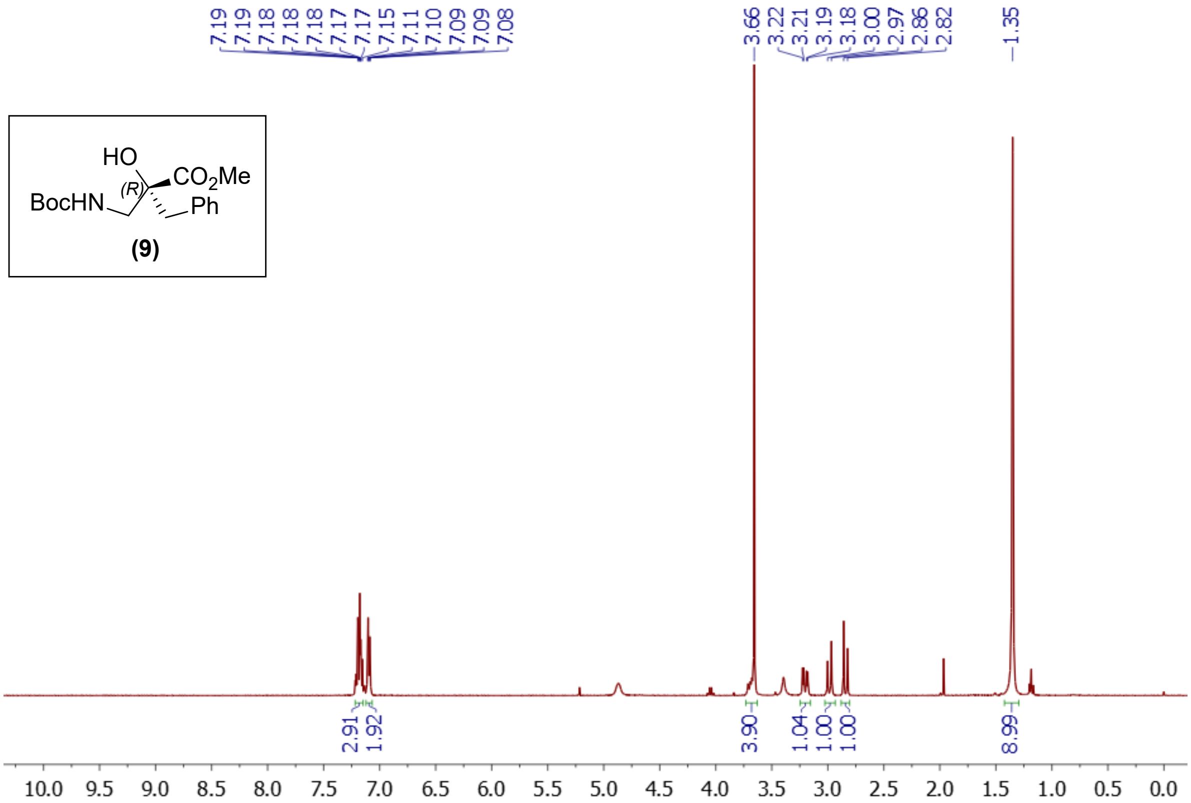

$^{13}C\{^1H\}$ NMR 100 MHz in $CDCl_3$

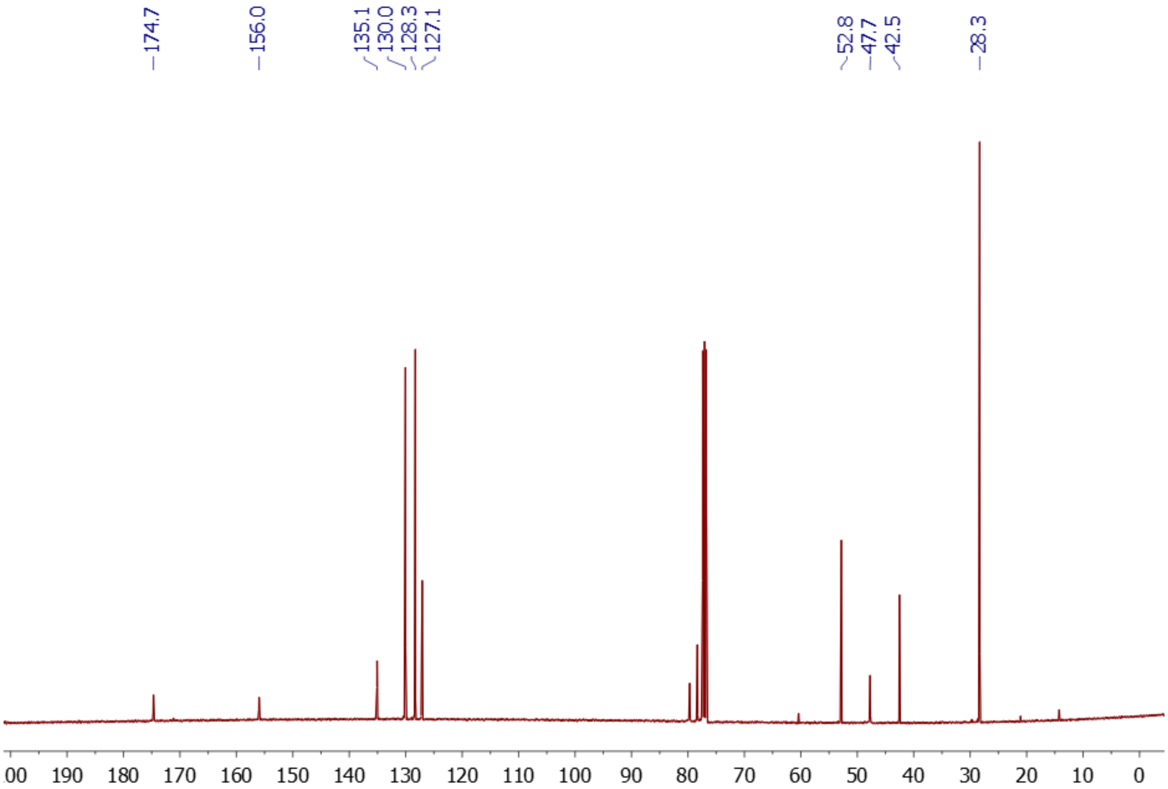

COSY in $CDCl_3$

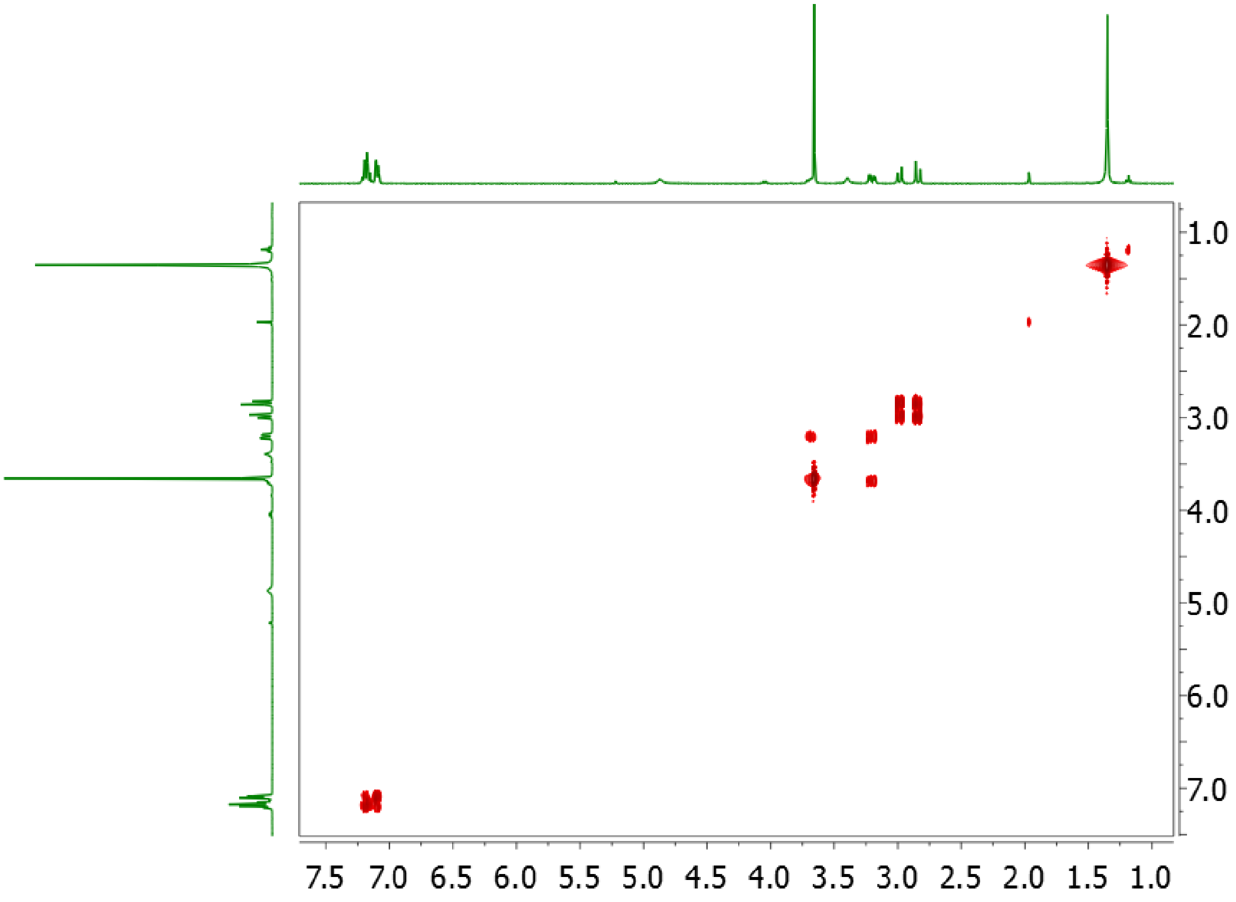

edited-HSQC in $CDCl_3$ **(color blue corresponds to CH_2 carbons and color red corresponds to CH_3 or CH carbons)**

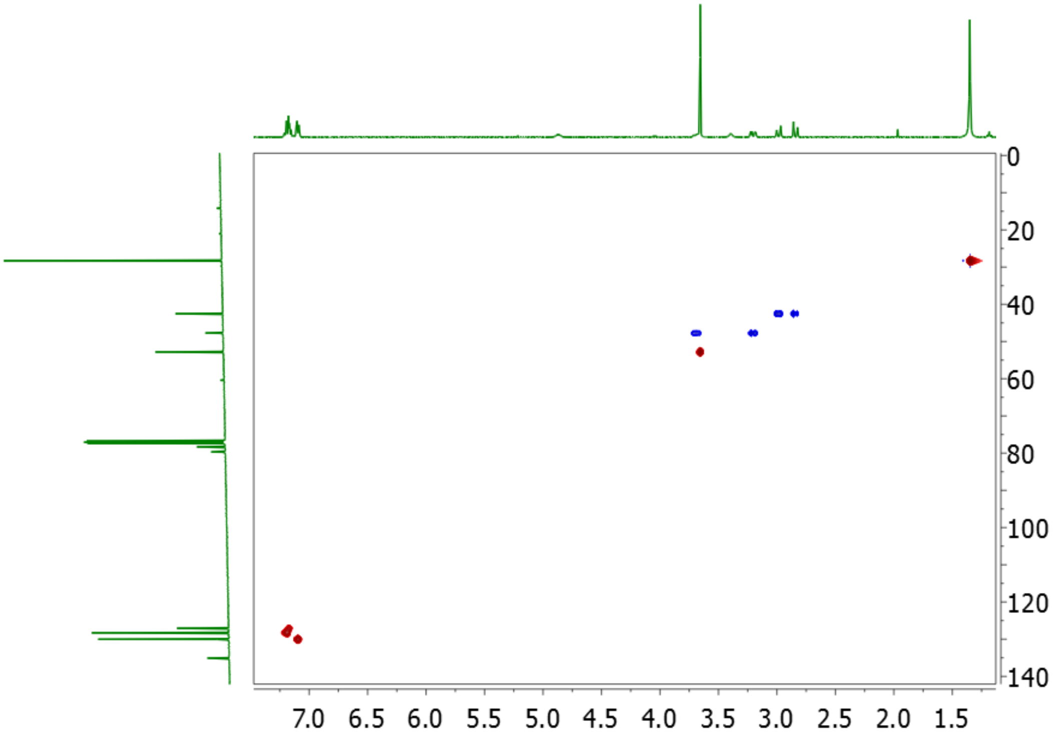

^1H NMR 400 MHz in $CDCl_3$

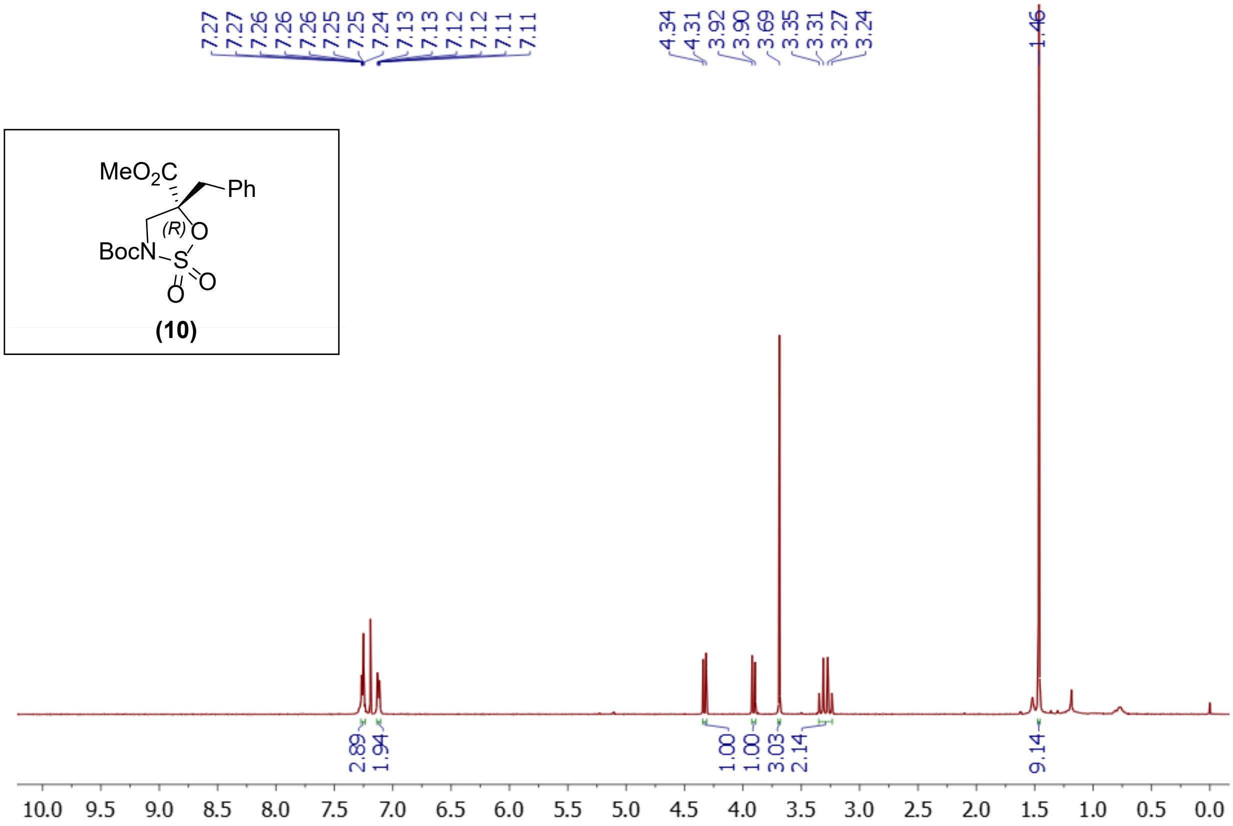

(10)

$^{13}C\{^1H\}$ NMR 100 MHz in $CDCl_3$

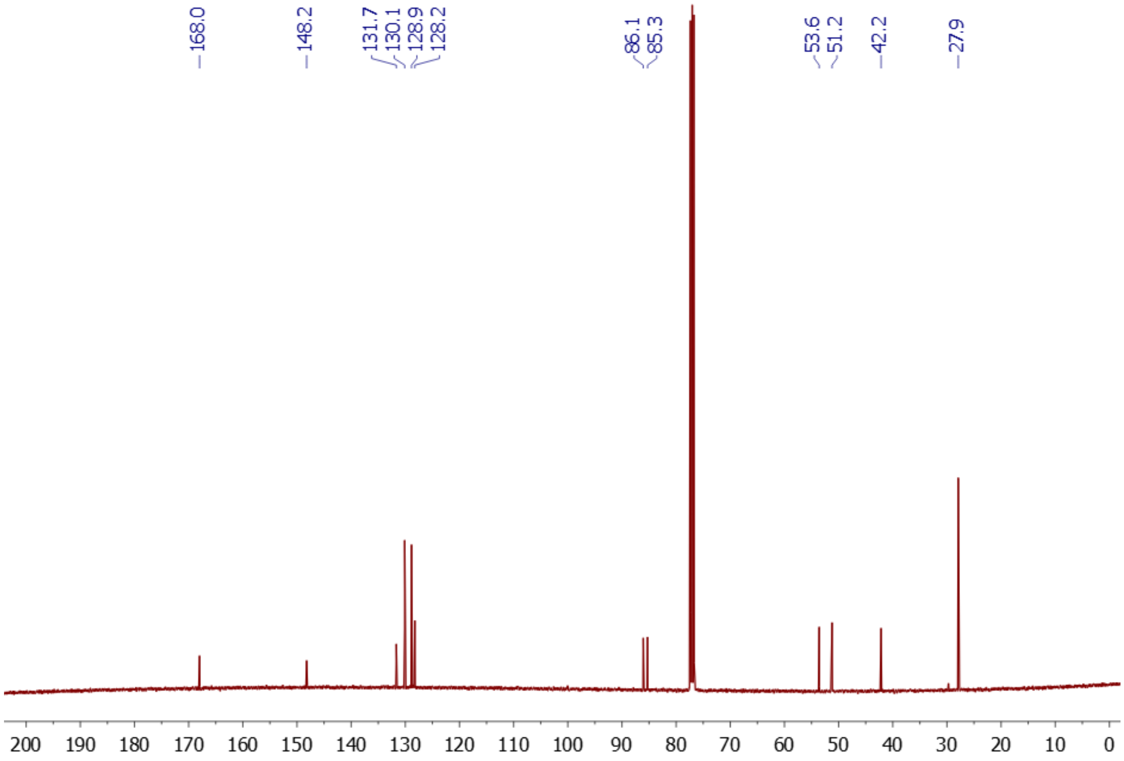

COSY in $CDCl_3$

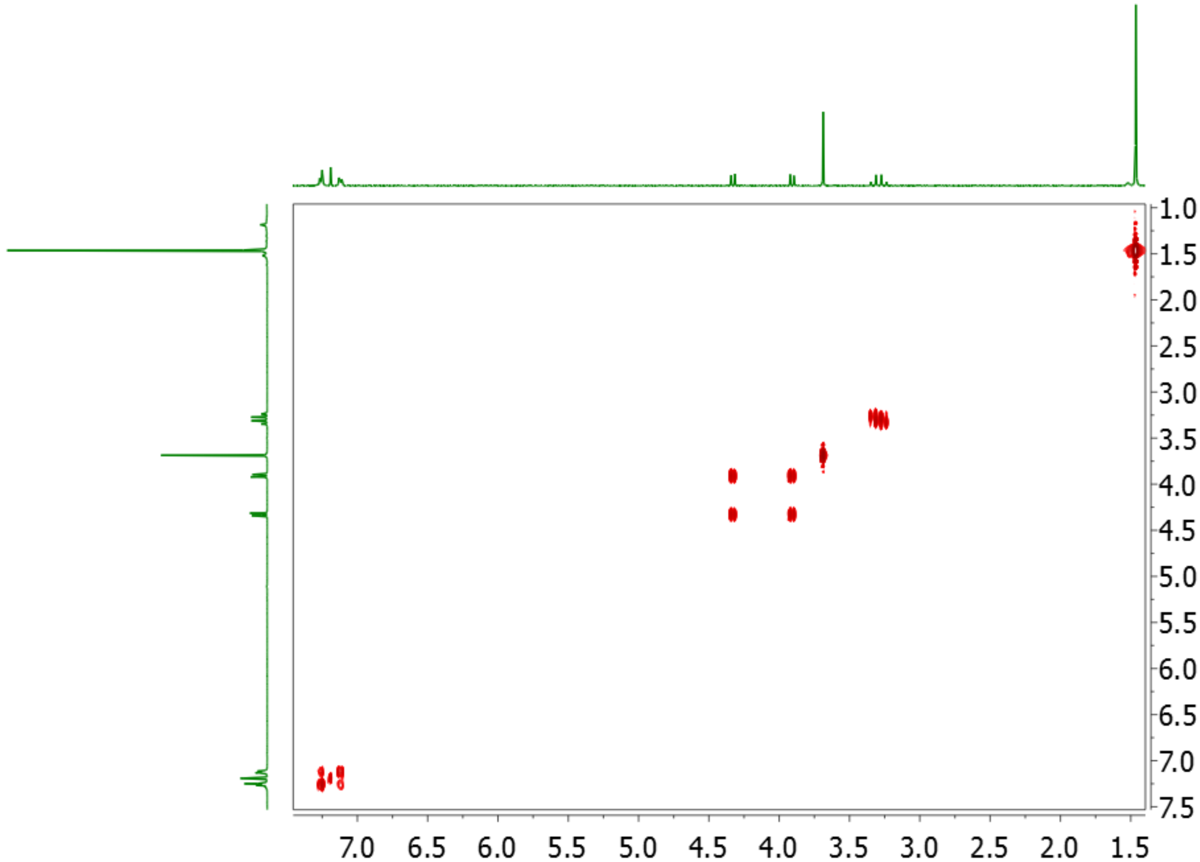

edited-HSQC in $CDCl_3$ **(color blue corresponds to CH_2 carbons and color red corresponds to CH_3 or CH carbons)**

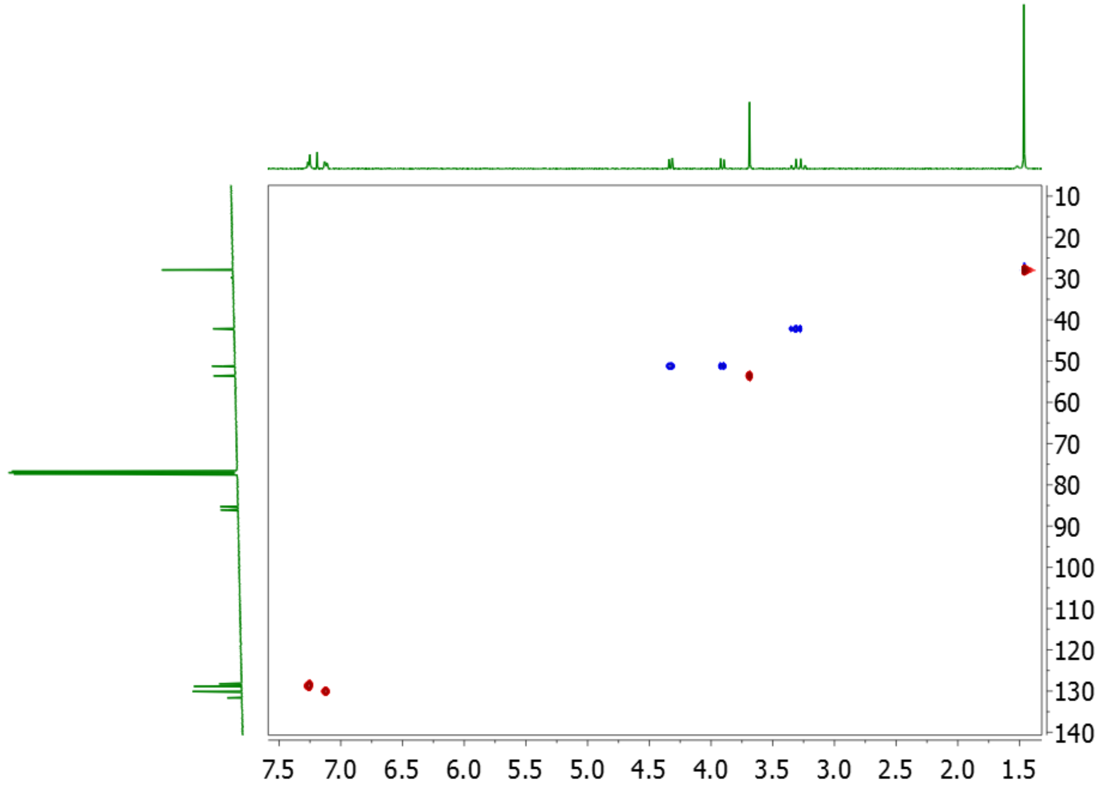

^{1}H NMR 400 MHz in $CDCl_3$

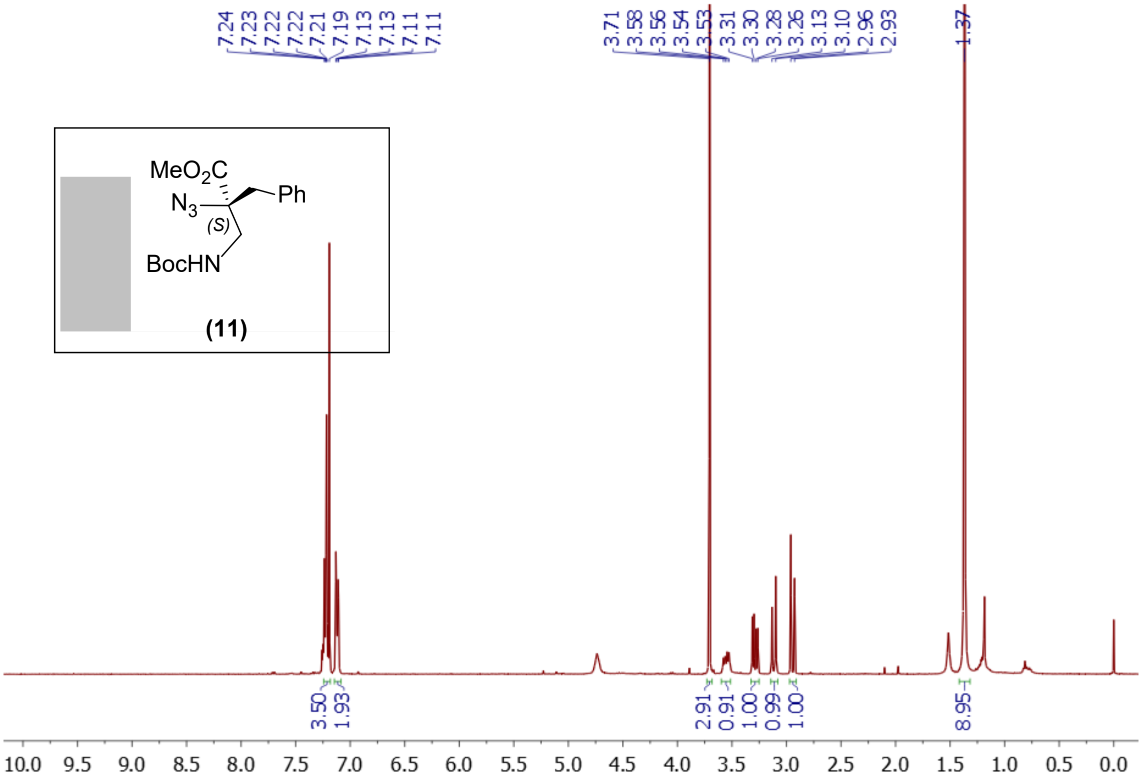

$^{13}C\{^{1}H\}$ NMR 100 MHz in $CDCl_3$

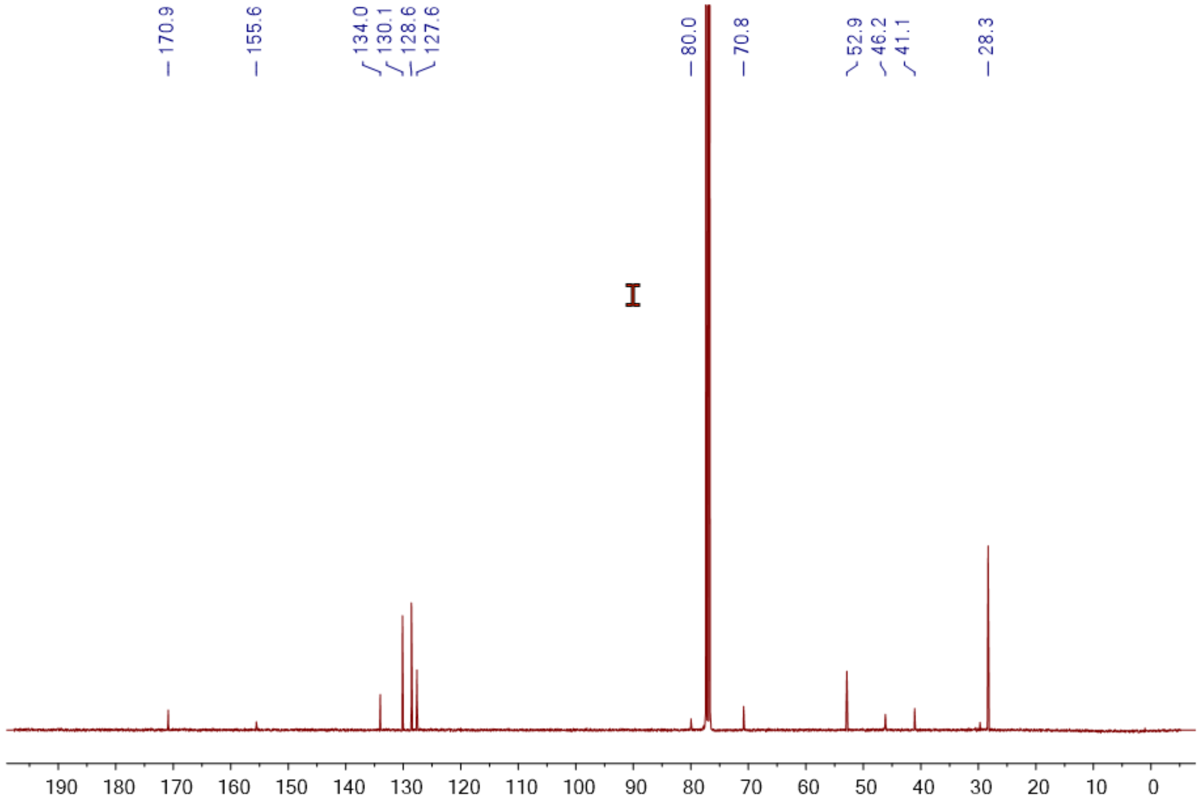

COSY in $CDCl_3$

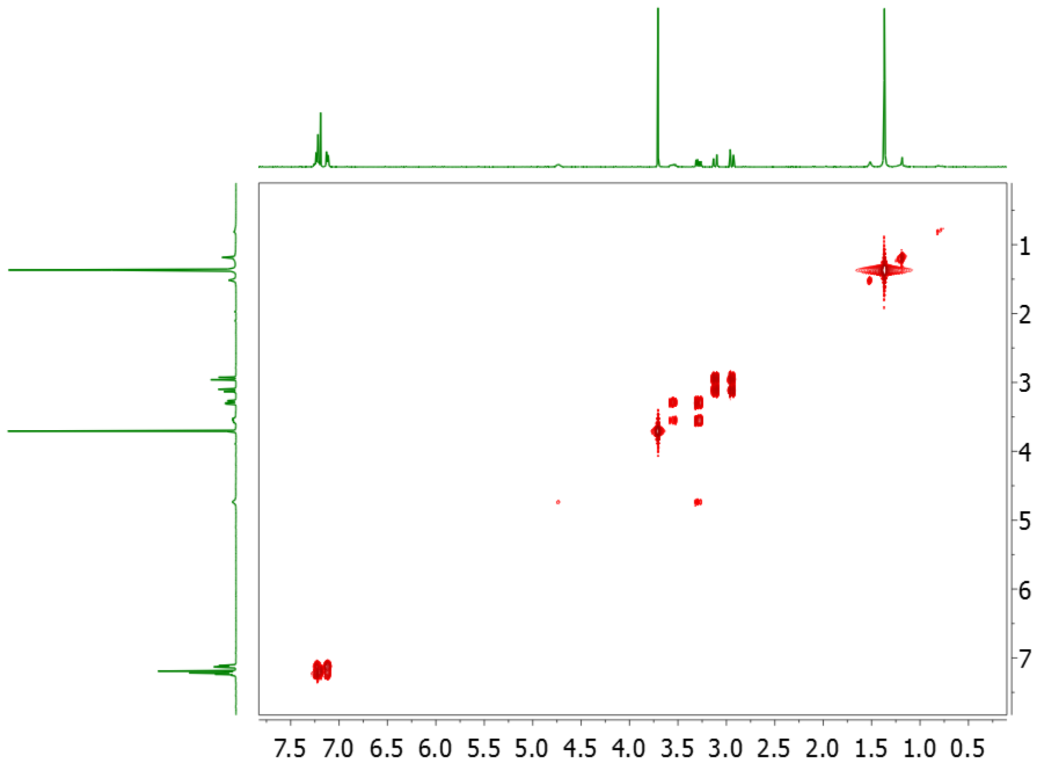

edited-HSQC in $CDCl_3$ **(color blue corresponds to CH_2 carbons and color red corresponds to CH_3 or CH carbons)**

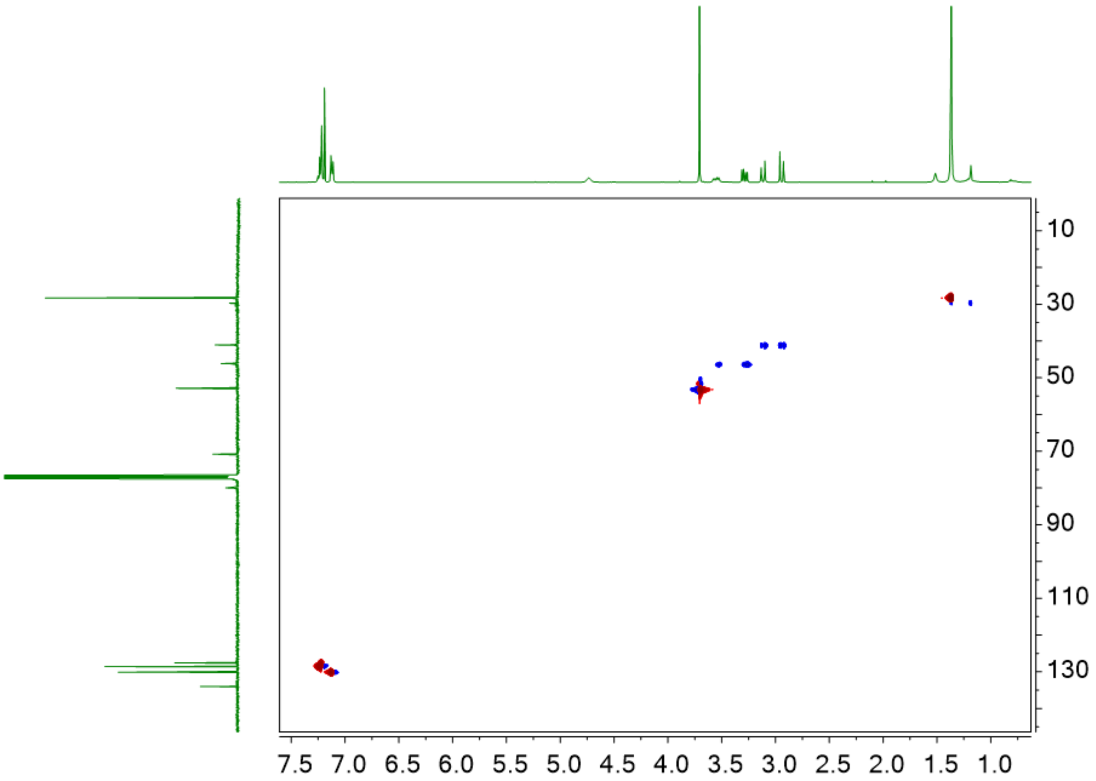

^{1}H NMR 400 MHz in $CDCl_3$

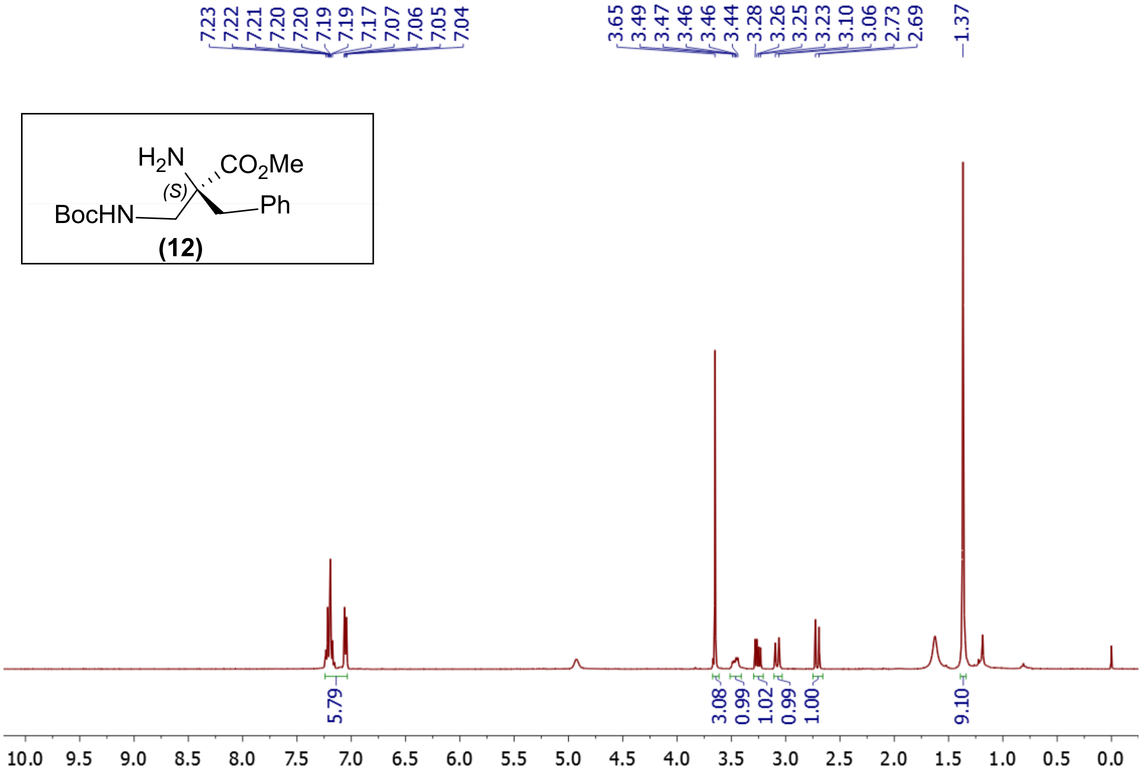

$^{13}C\{^{1}H\}$ NMR 100 MHz in $CDCl_3$

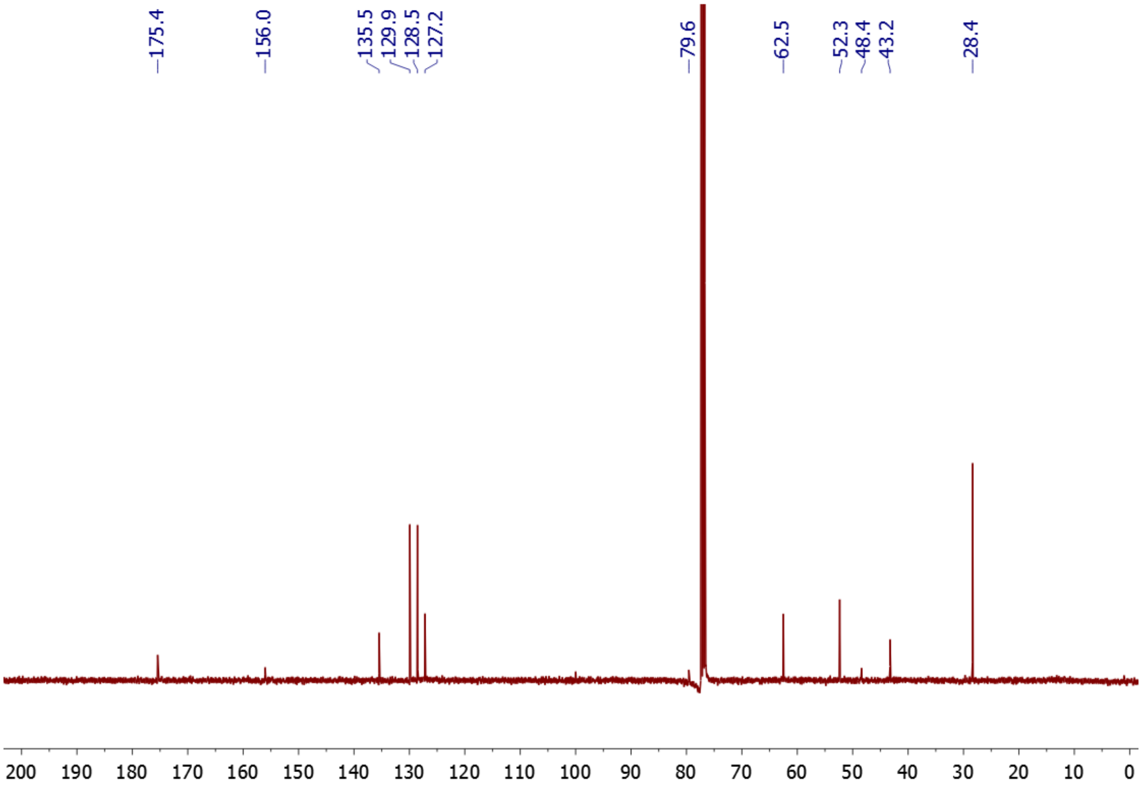

COSY in $CDCl_3$

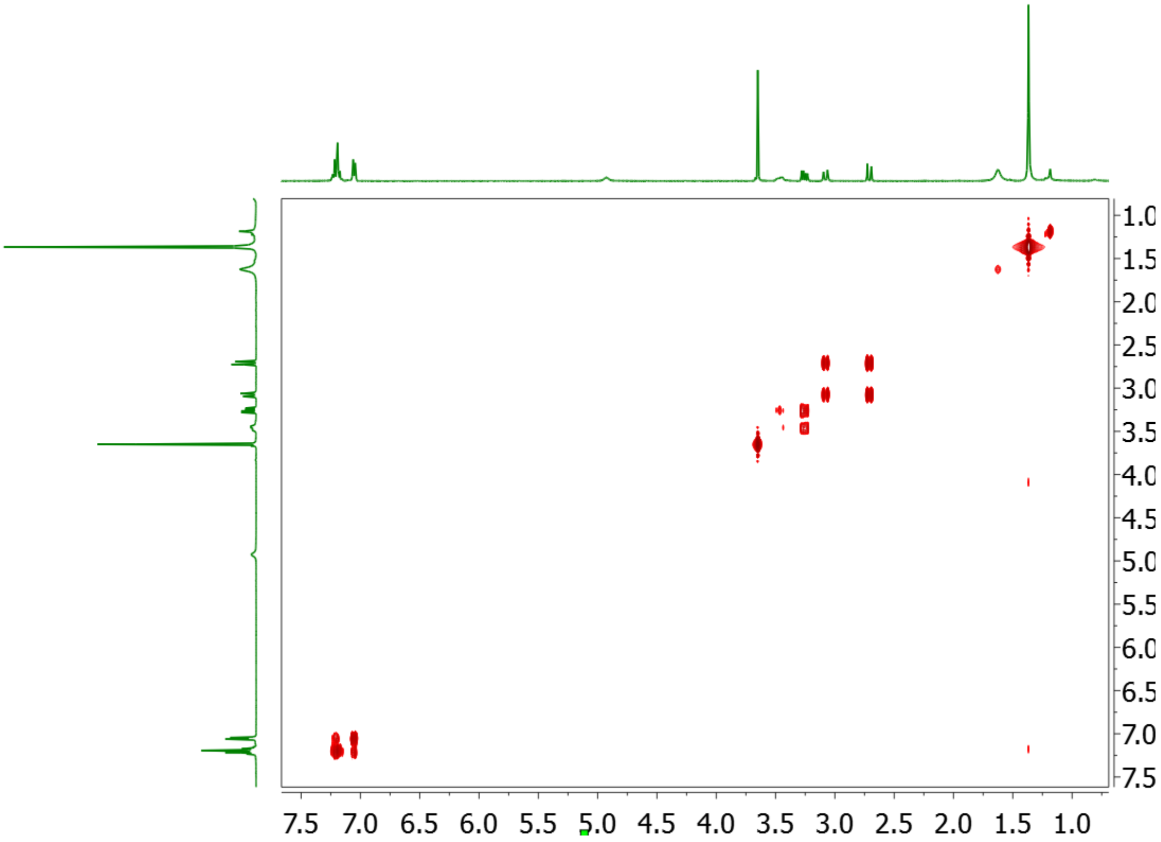

edited-HSQC in $CDCl_3$ **(color blue corresponds to CH_2 carbons and color red corresponds to CH_3 or CH carbons)**

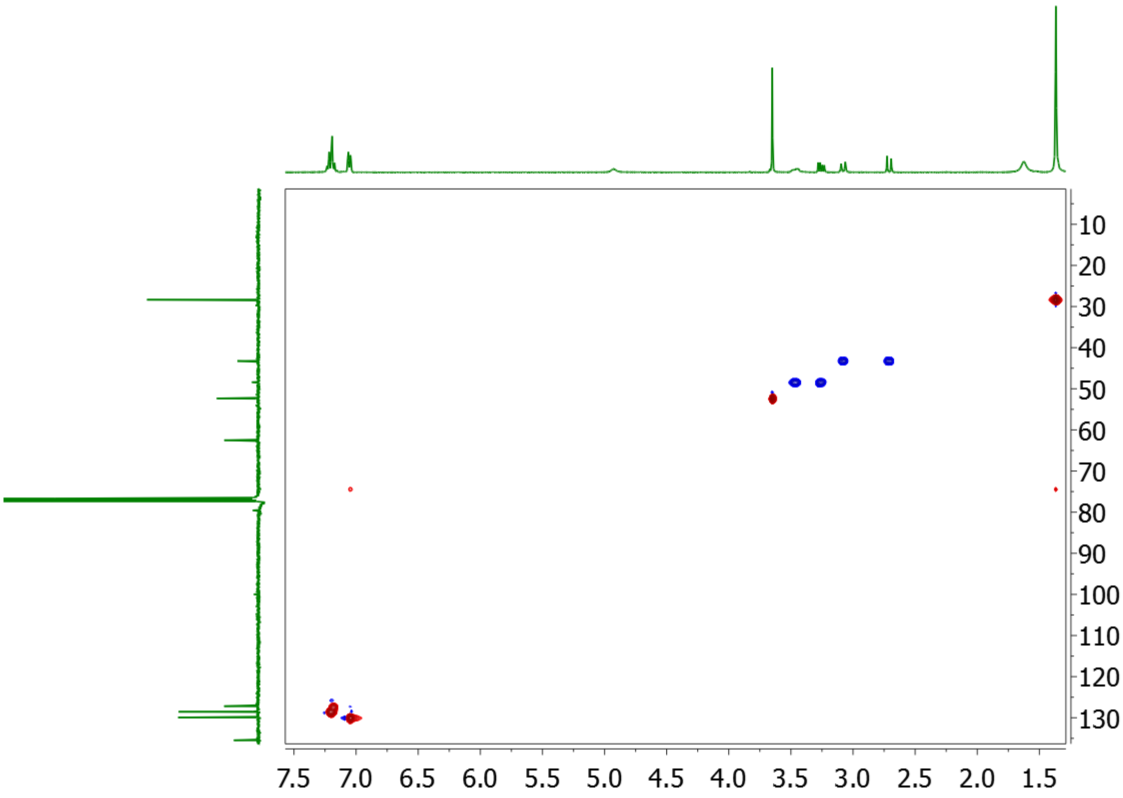

^1H NMR 400 MHz in $CDCl_3$

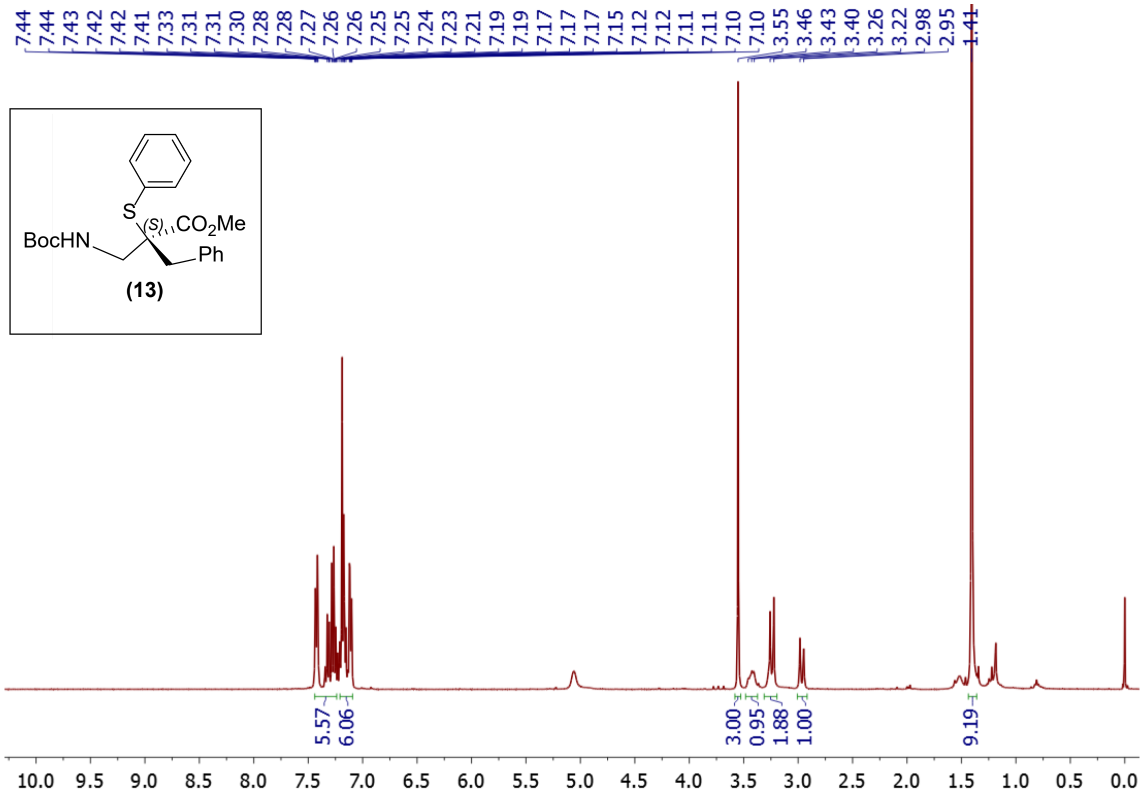

$^{13}C\{^1H\}$ NMR 100 MHz in $CDCl_3$

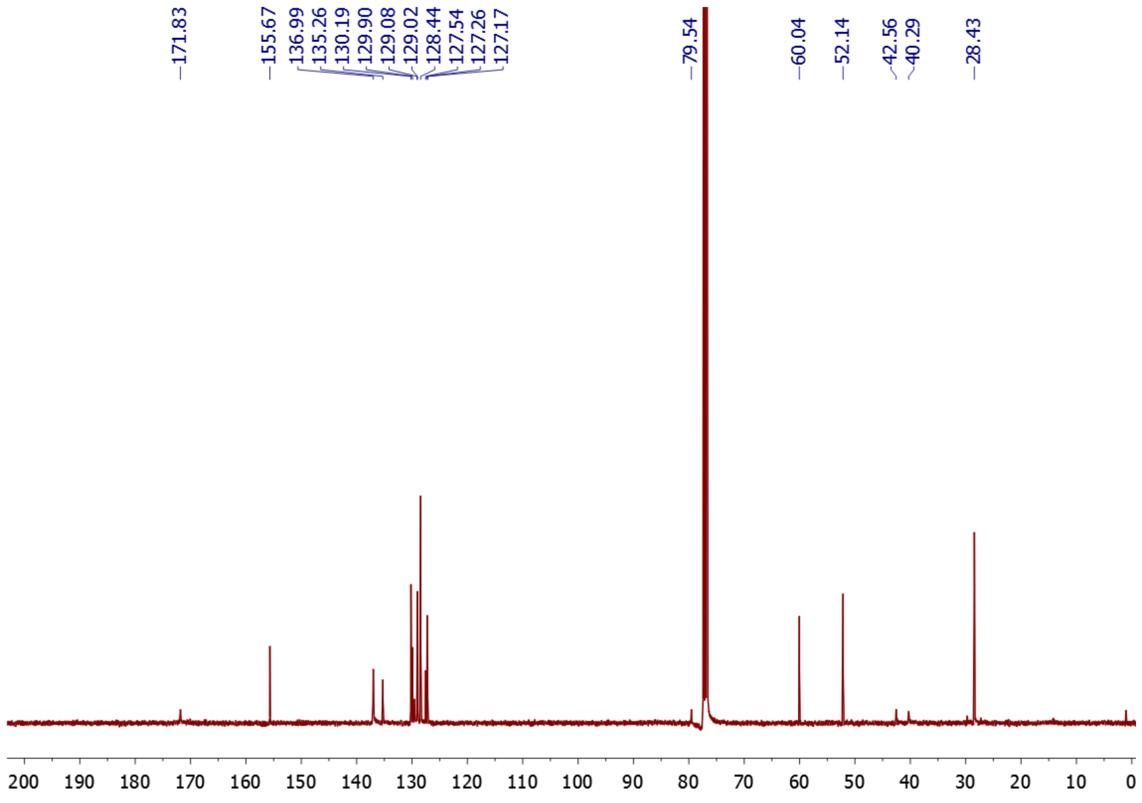

COSY in CD_3Cl

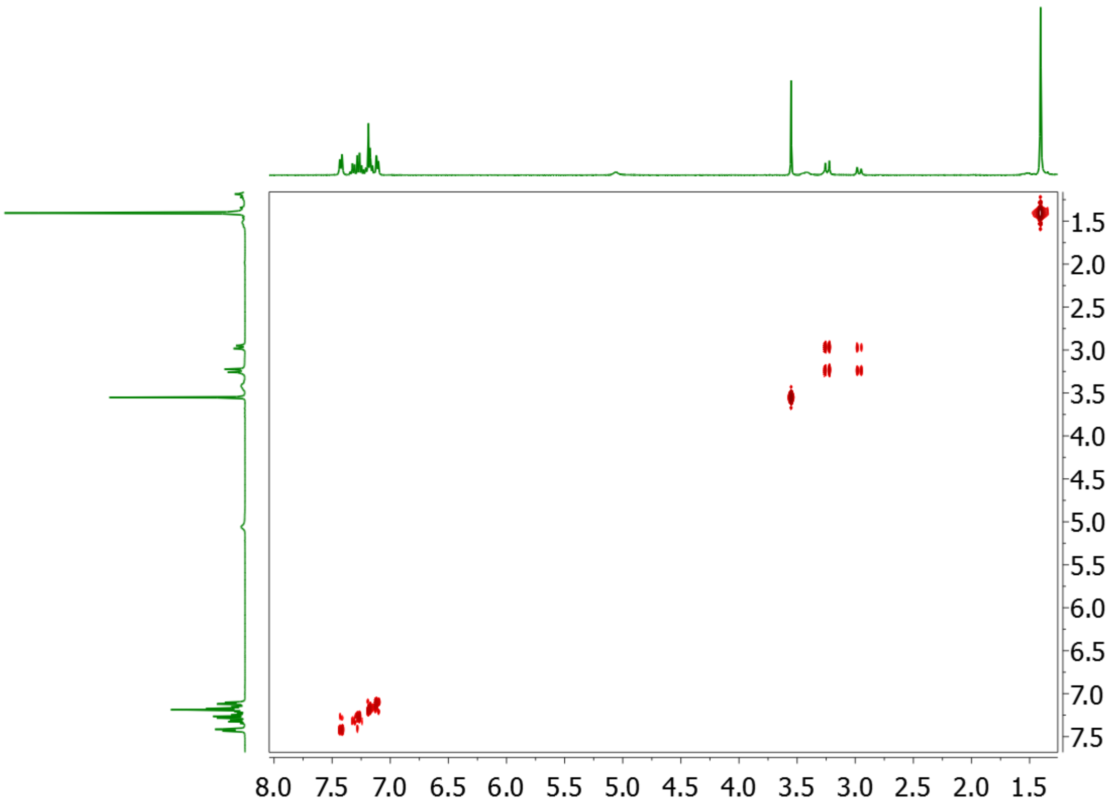

edited-HSQC in $CDCl_3$ **(color blue corresponds to CH_2 carbons and color red corresponds to CH_3 or CH carbons)**

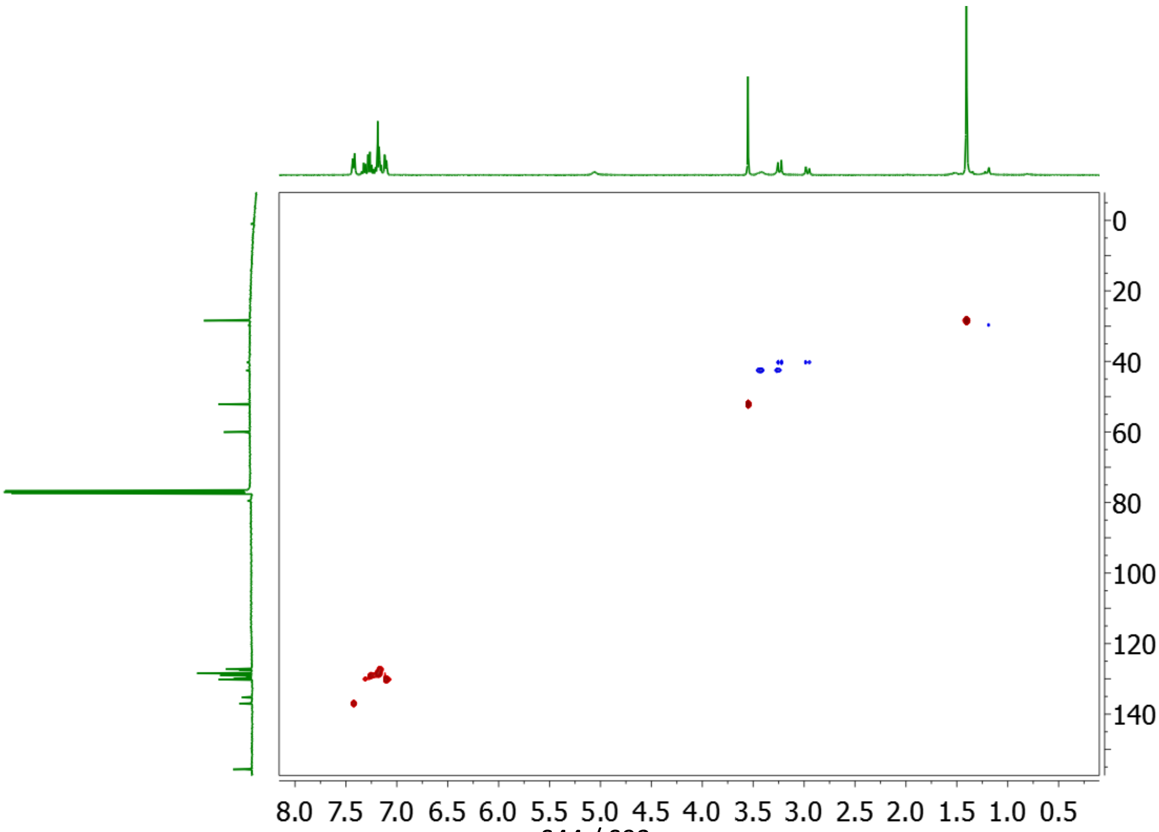

^{1}H NMR 400 MHz in $CDCl_3$

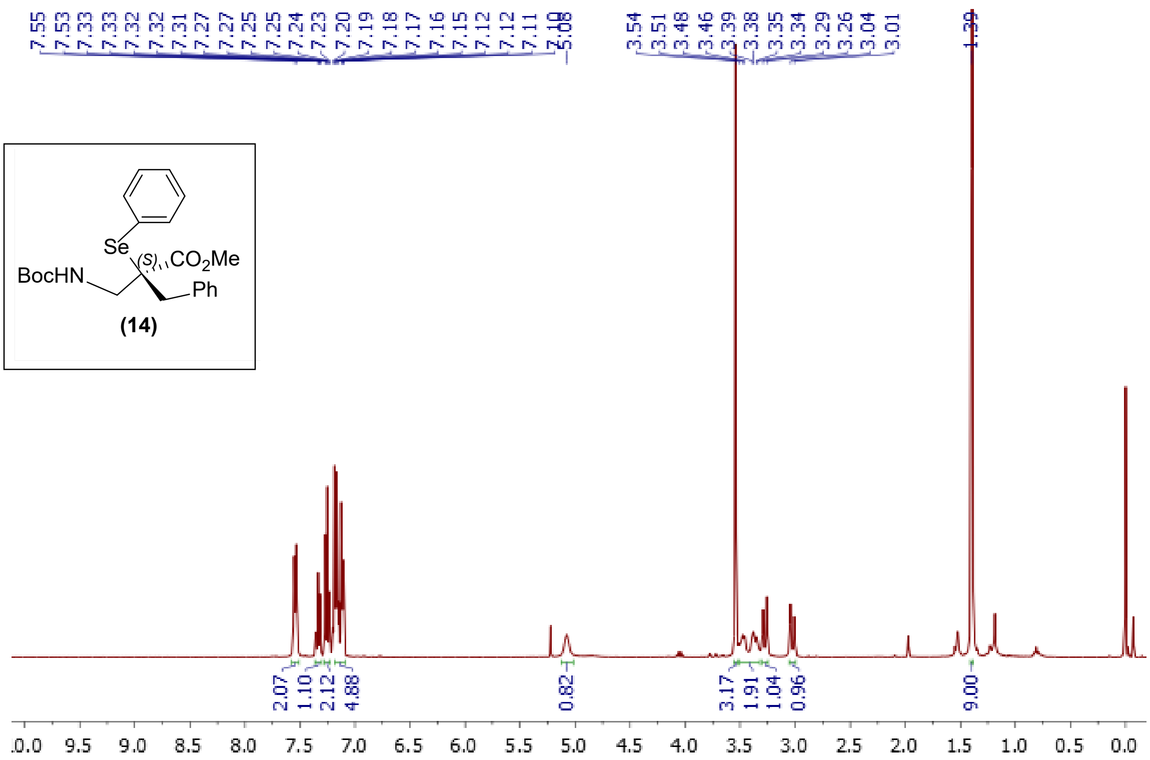

^{13}C{^{1}H} NMR 100 MHz in $CDCl_3$

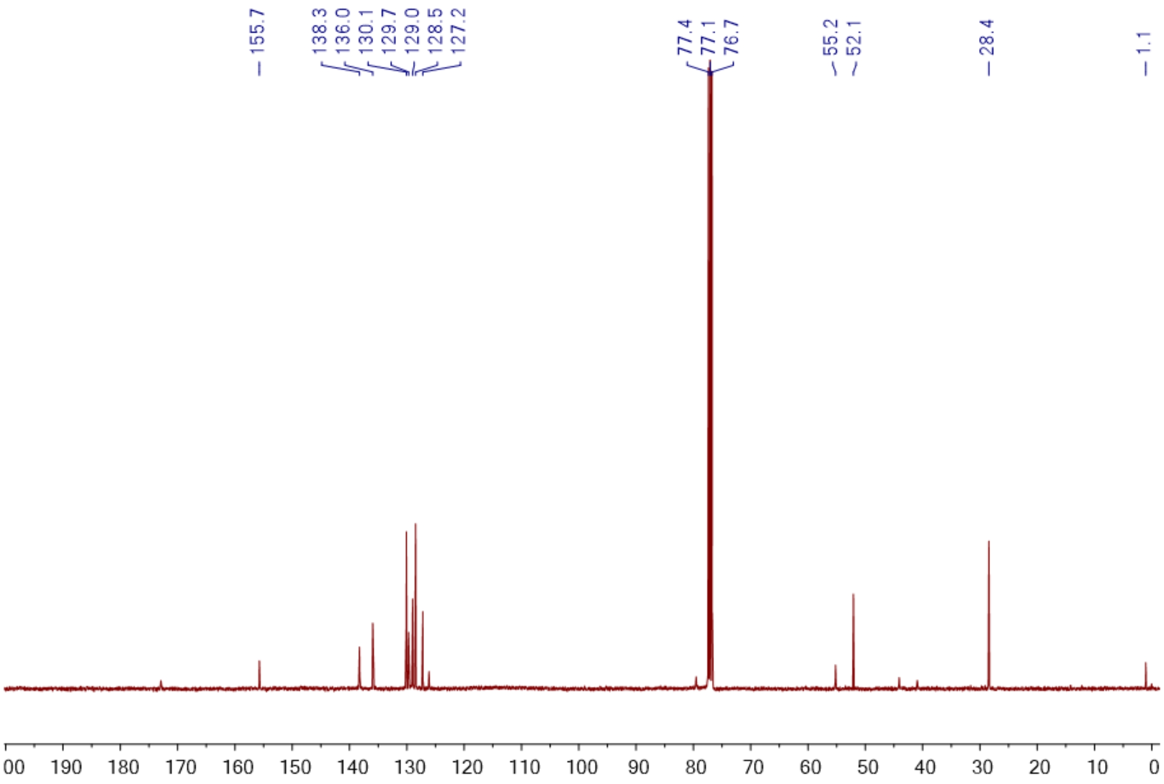

COSY in $CDCl_3$

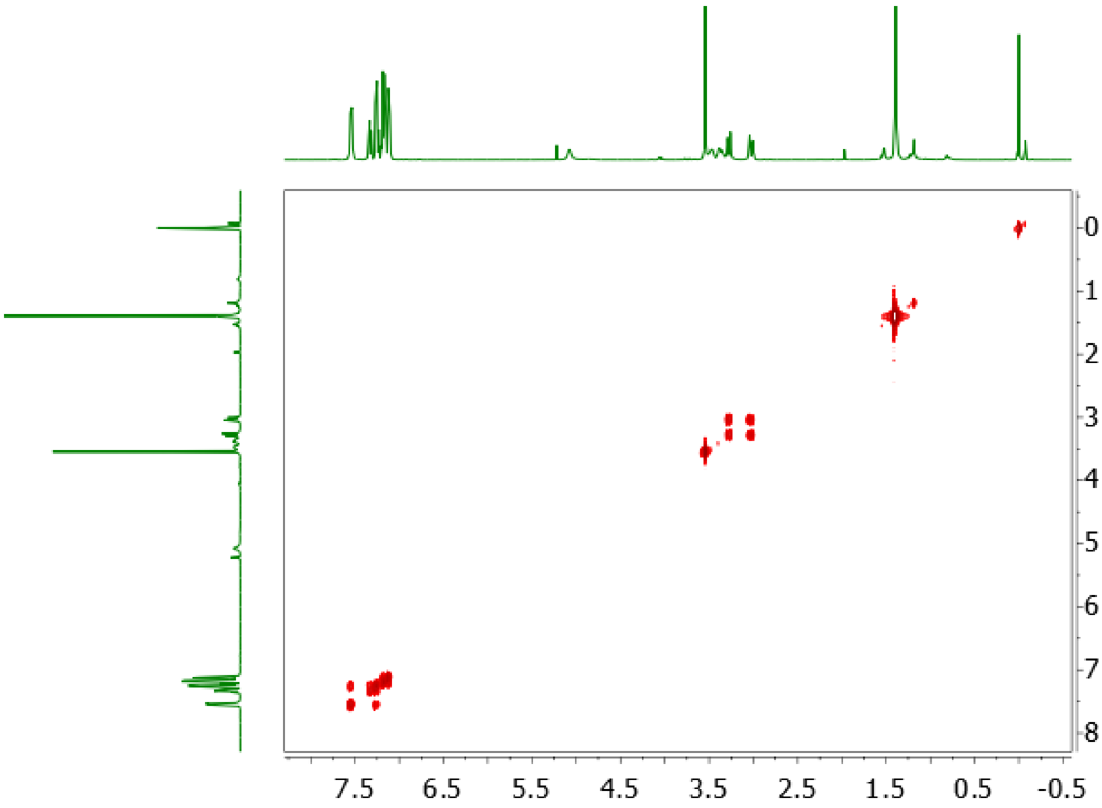

edited-HSQC in $CDCl_3$ **(color blue corresponds to CH_2 carbons and color red corresponds to CH_3 or CH carbons)**

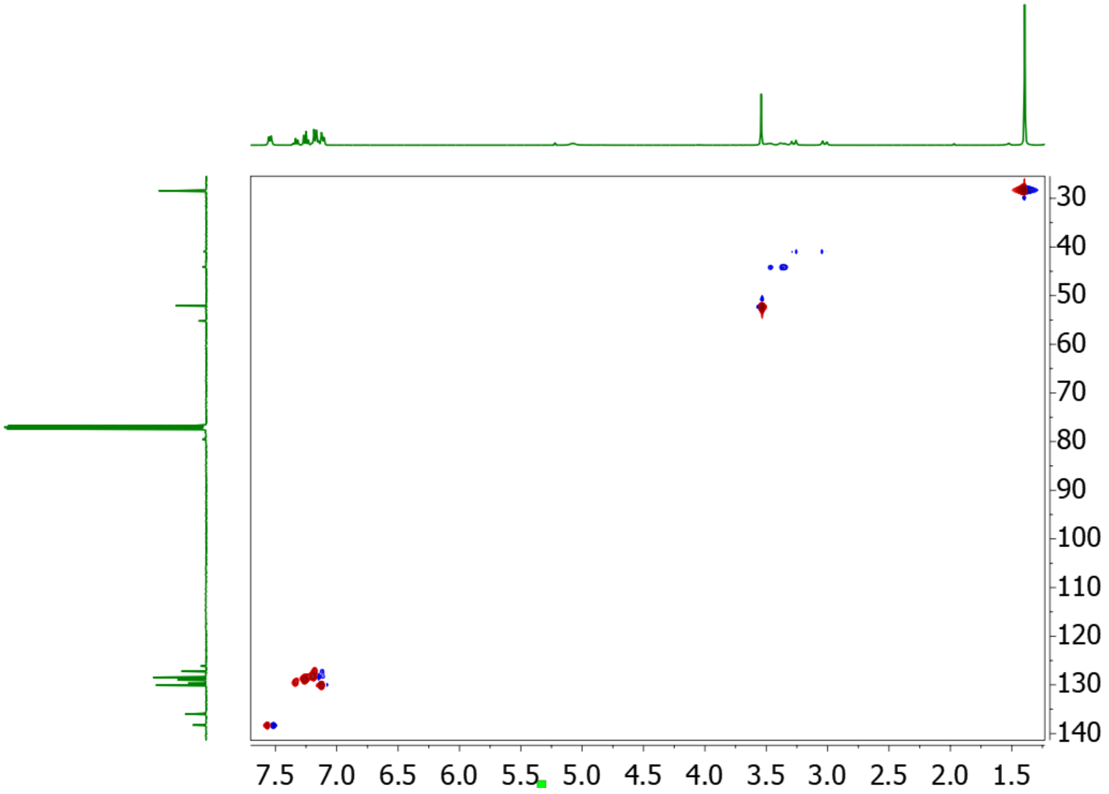

^1H NMR 400 MHz in $CDCl_3$

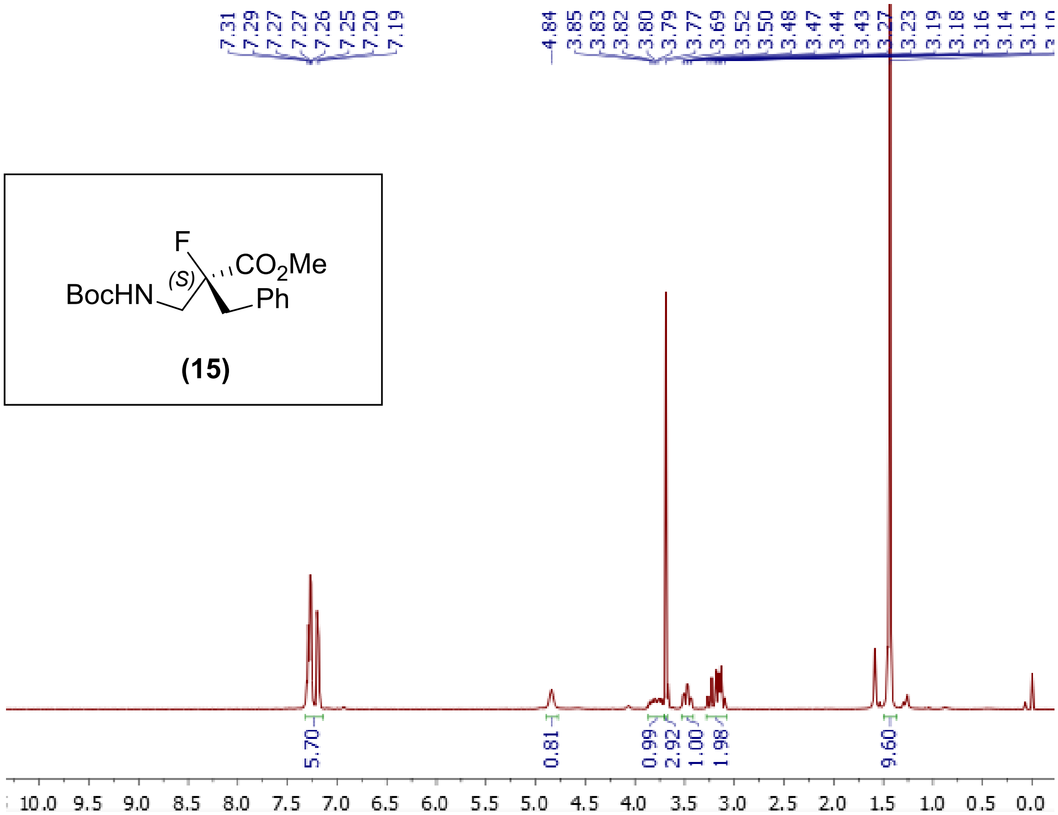

(15)

$^{13}C\{^1H\}$ NMR 100 MHz in $CDCl_3$

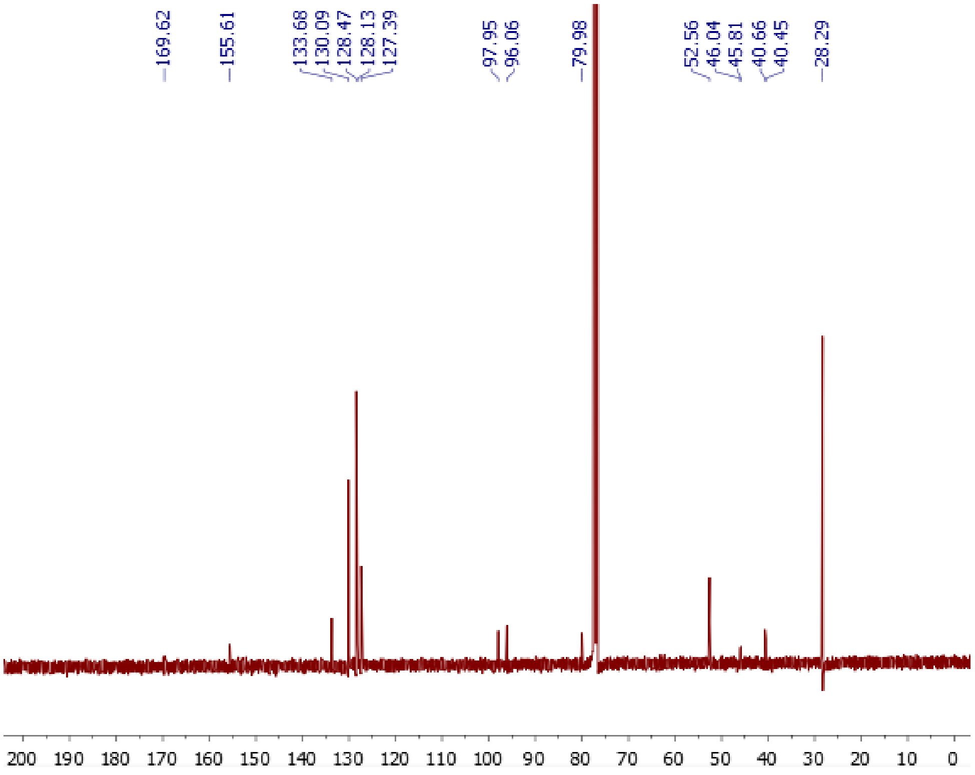

COSY in $CDCl_3$

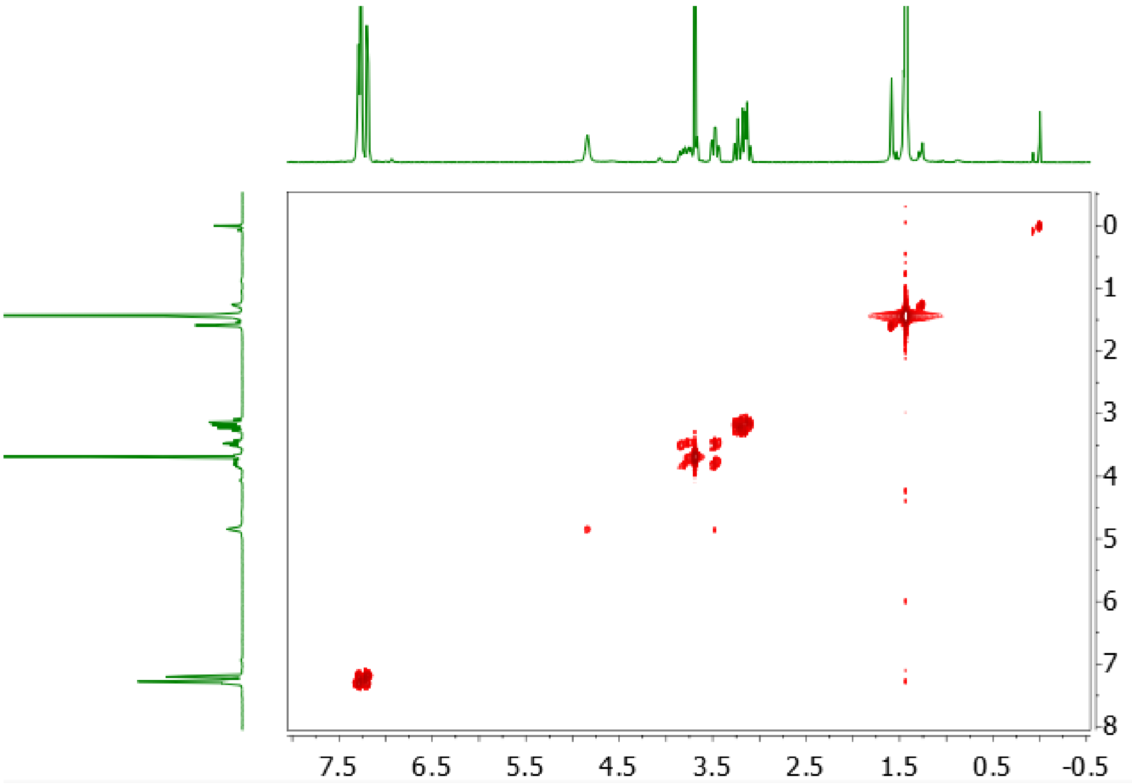

edited-HSQC in $CDCl_3$ **(color blue corresponds to CH_2 carbons and color red corresponds to CH_3 or CH carbons)**

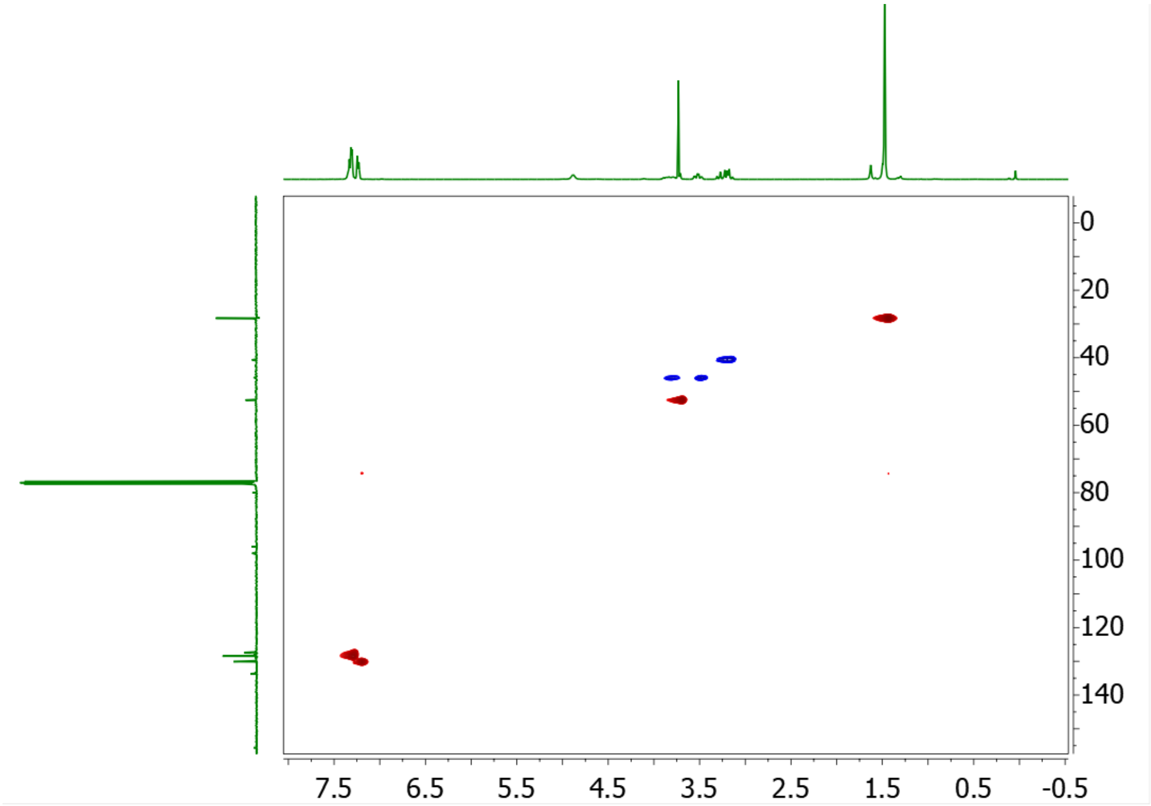

$^{19}F\{^{1}H\}$ NMR 281.25 MHz in $CDCl_3$

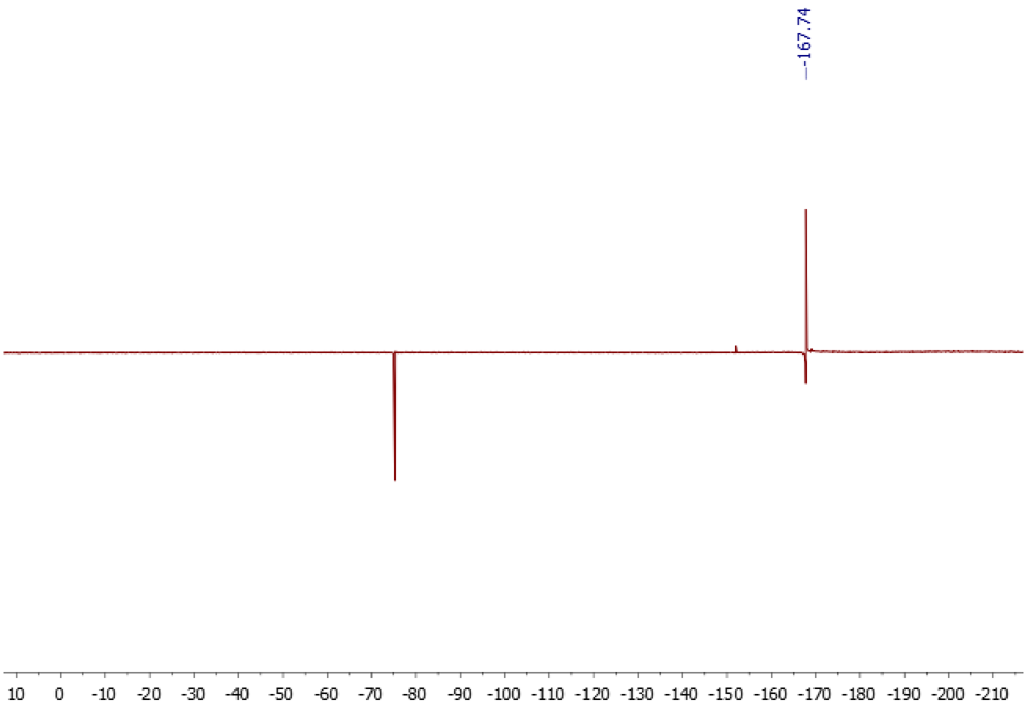

^{1}H NMR 400 MHz in $CDCl_3$

Spectral data match with previously published data (reference 57 in the manuscript).

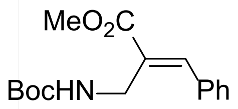

(15b)

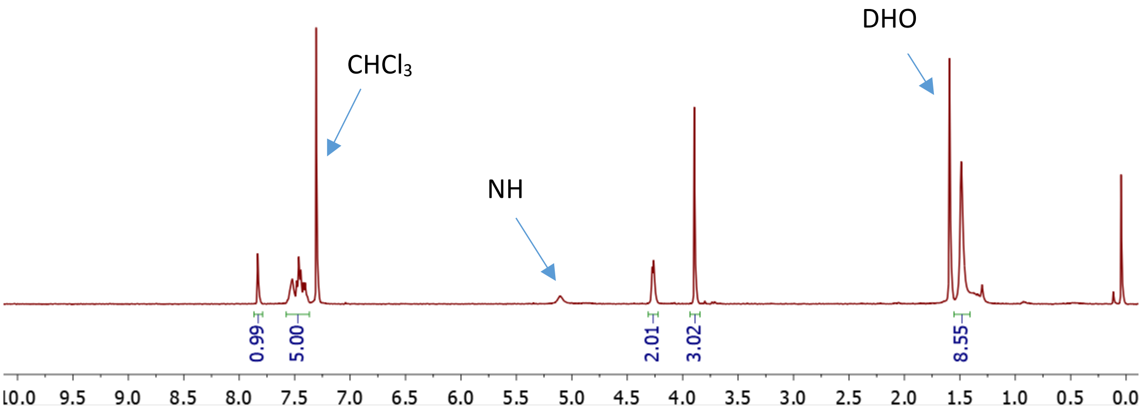

^{1}H NMR 400 MHz in D_2O

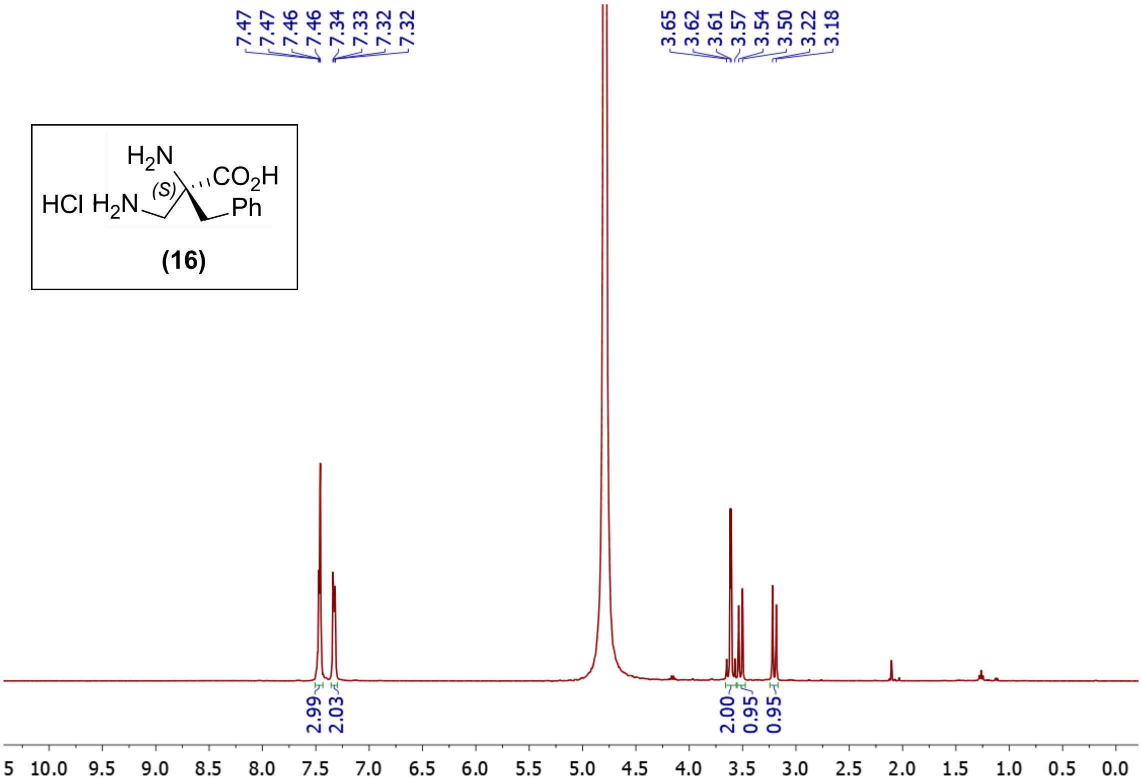

$^{13}C\{^{1}H\}$ NMR 100 MHz in D_2O

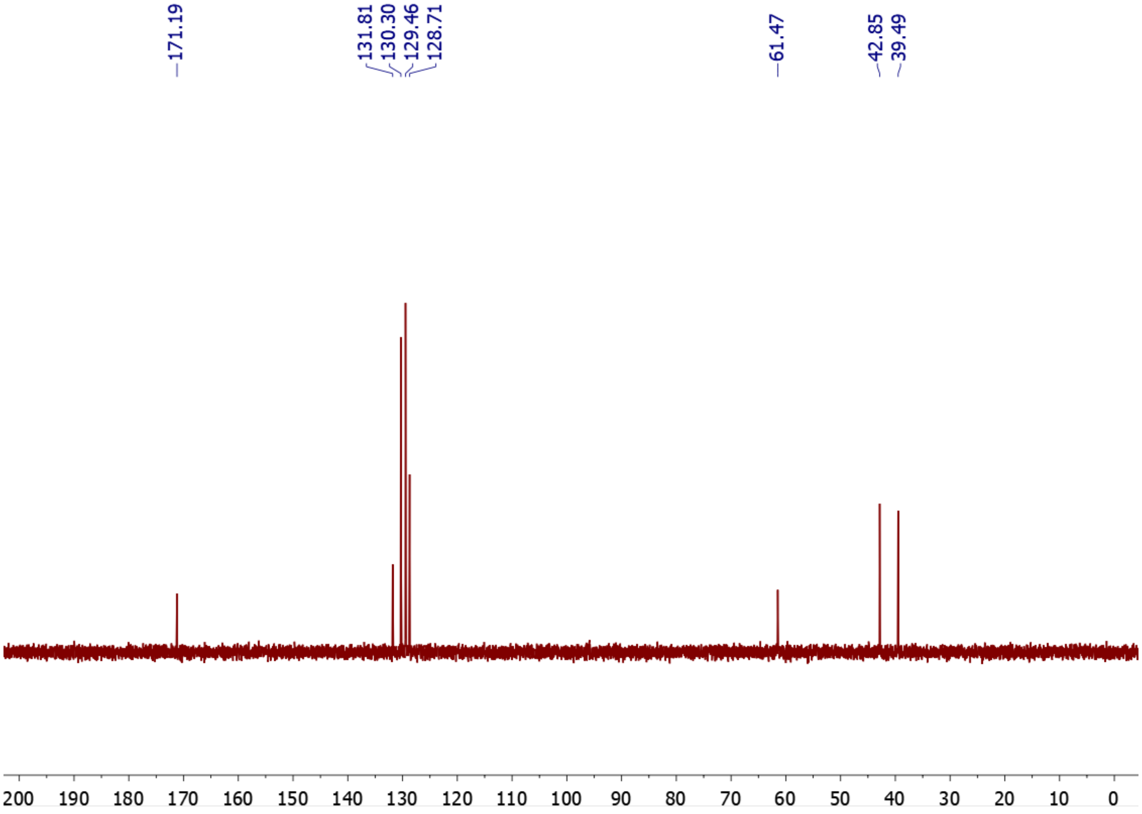

COSY in D_2O

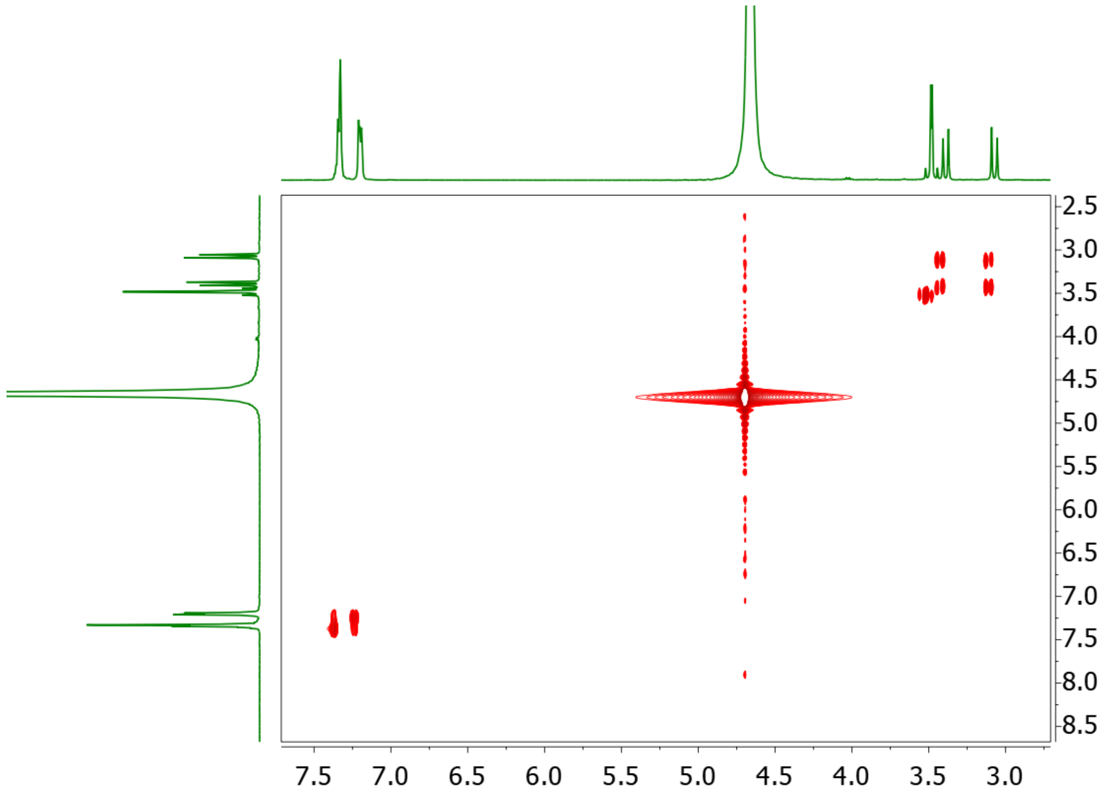

edited-HSQC in D_2O **(color blue corresponds to CH_2 carbons and color red corresponds to CH_3 or CH carbons)**

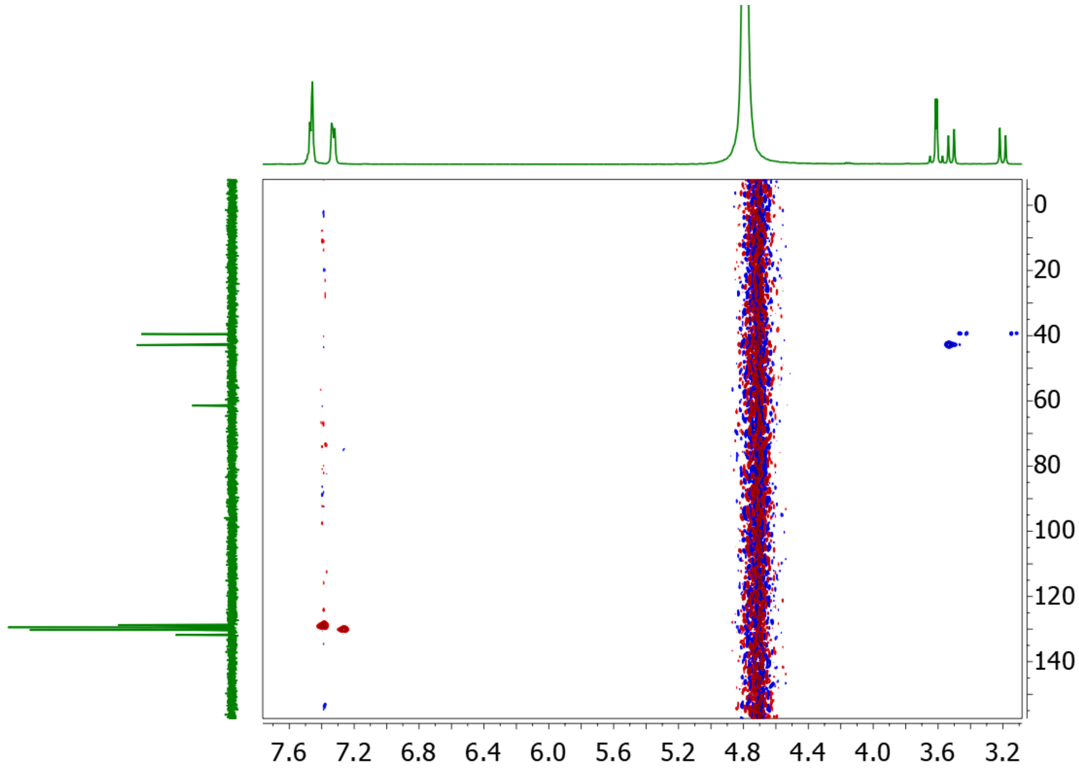

^{1}H NMR 400 MHz in D_2O

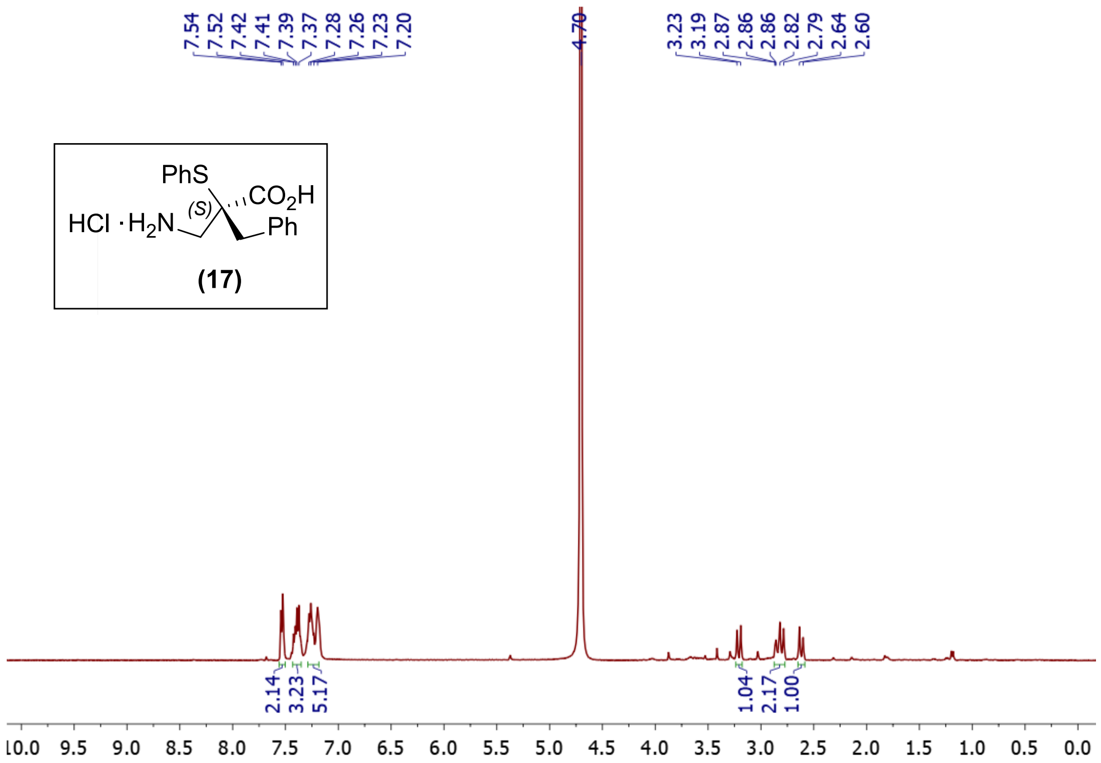

$^{13}C\{^{1}H\}$ NMR 100 MHz in D_2O

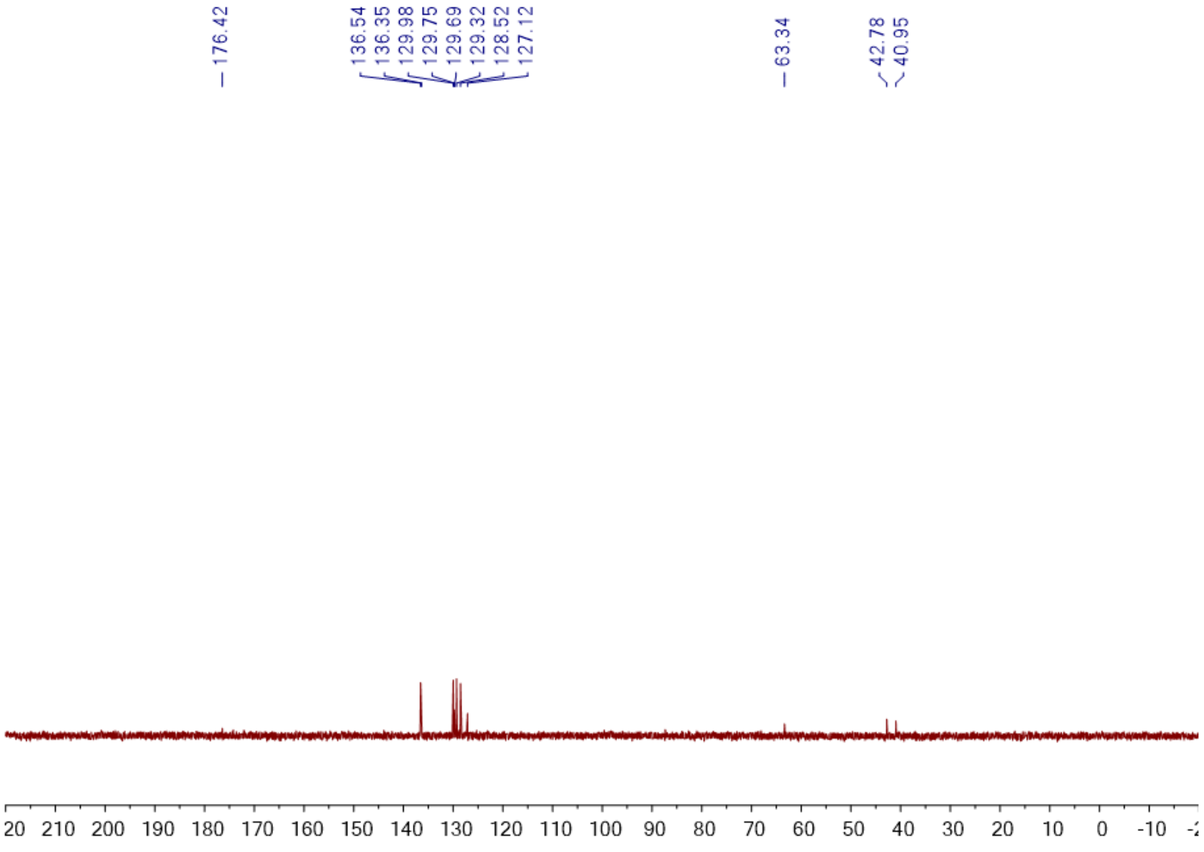

COSY in D_2O

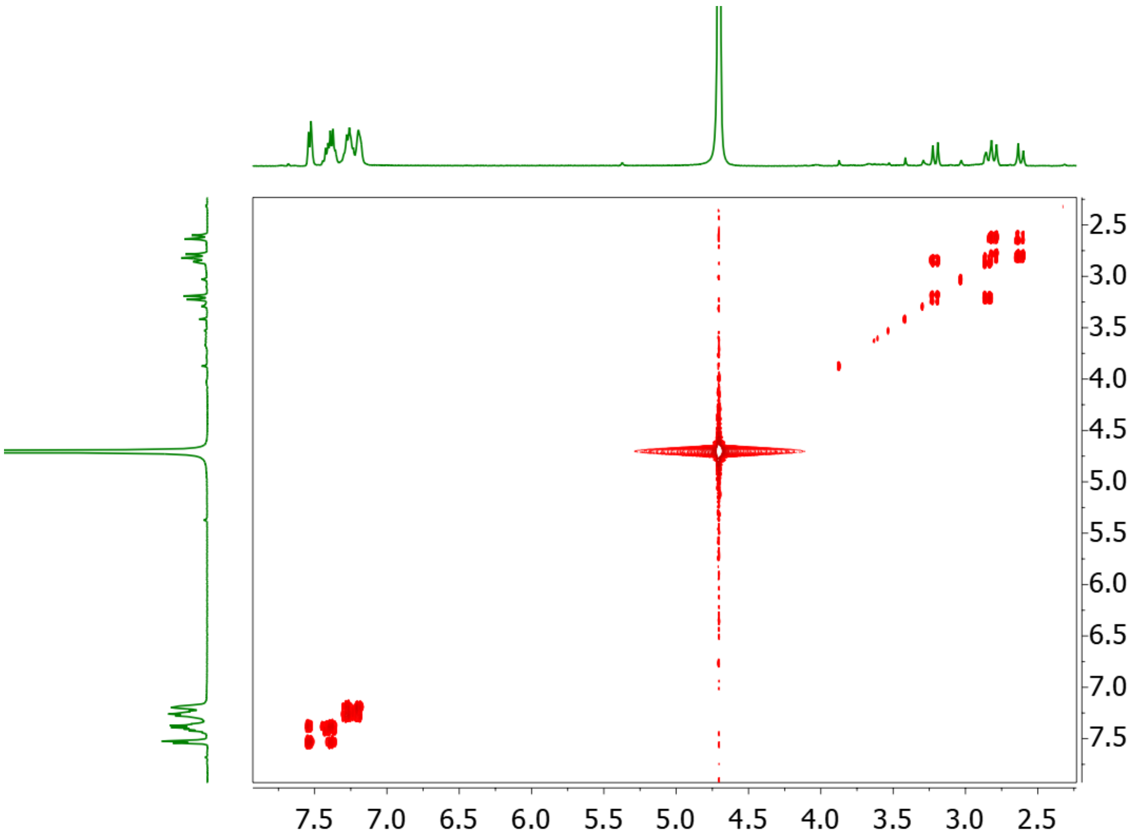

edited-HSQC in D_2O **(color blue corresponds to CH_2 carbons and color red corresponds to CH_3 or CH carbons)**

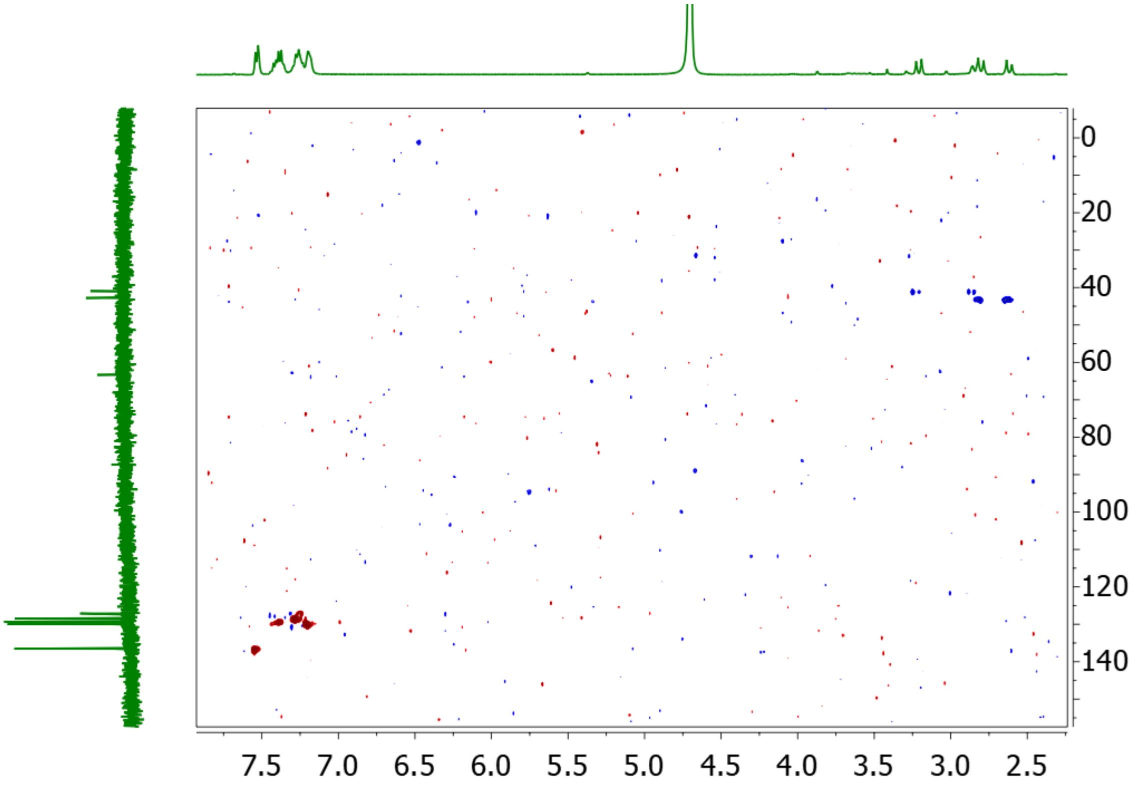

^1H NMR 400 MHz in D_2O

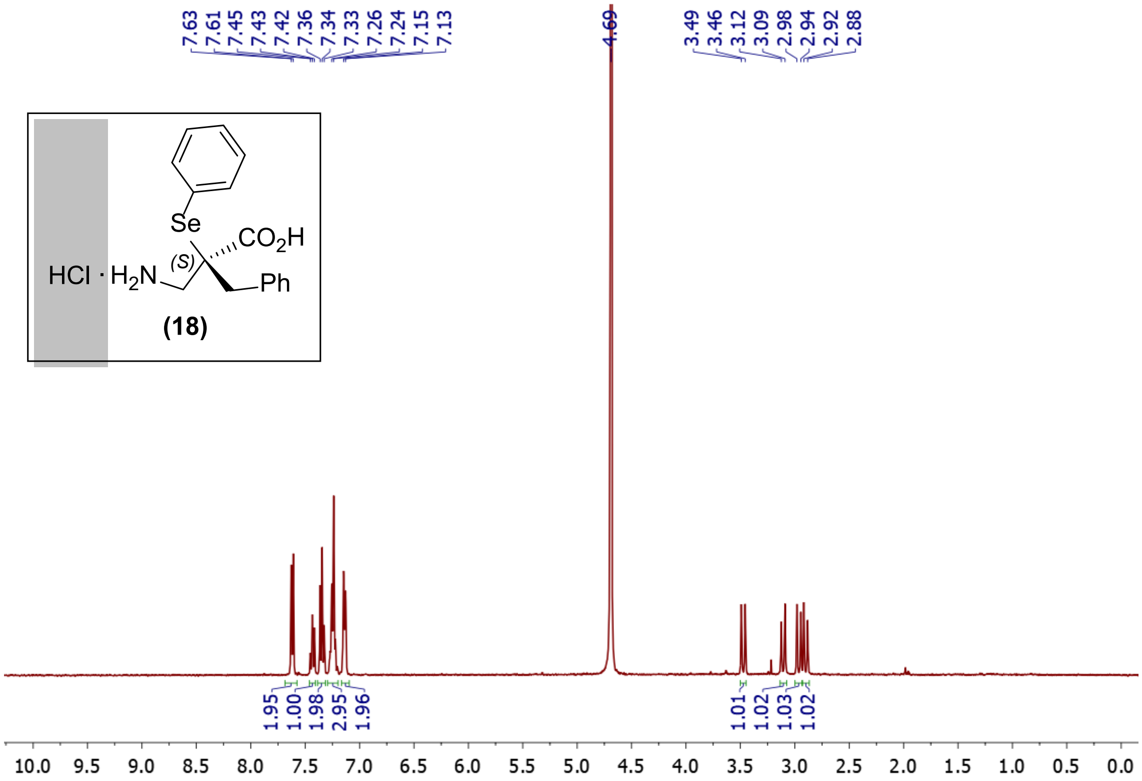

$^{13}C\{^1H\}$ NMR 75 MHz in D_2O

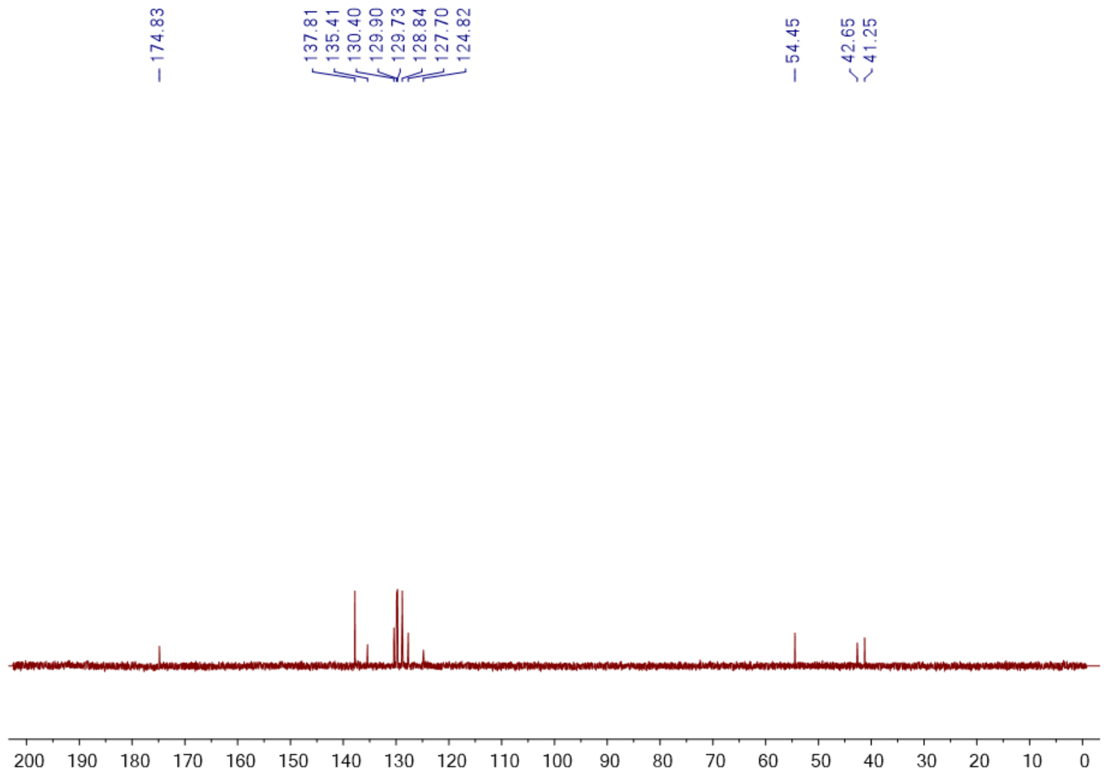

COSY in D_2O

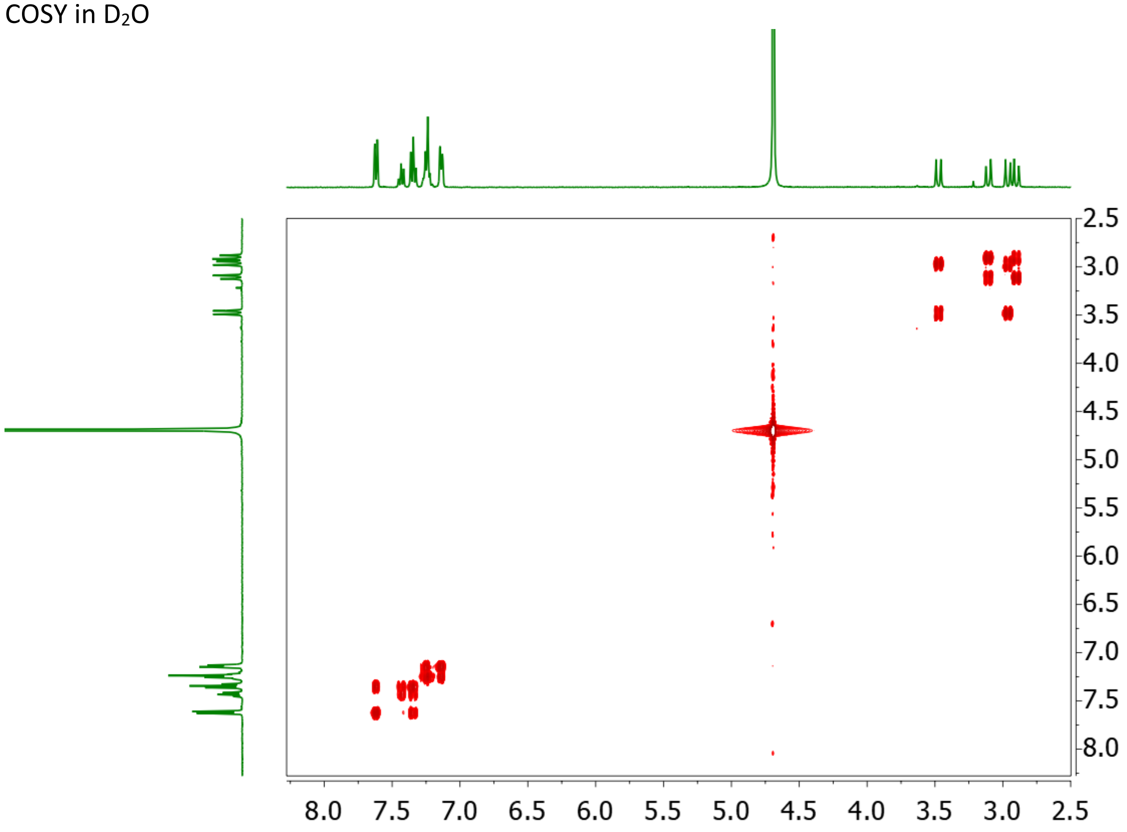

edited-HSQC in D_2O **(color blue corresponds to CH_2 carbons and color red corresponds to CH_3 or CH carbons)**

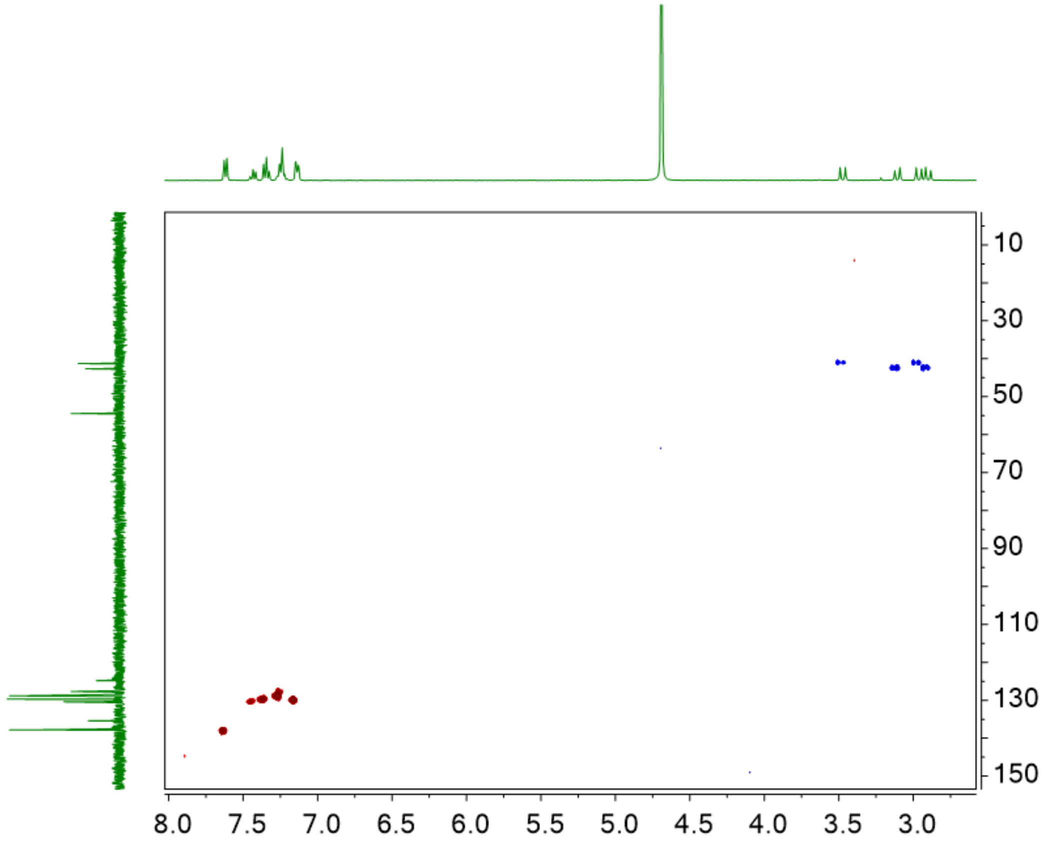

^{1}H NMR 400 MHz in D_2O

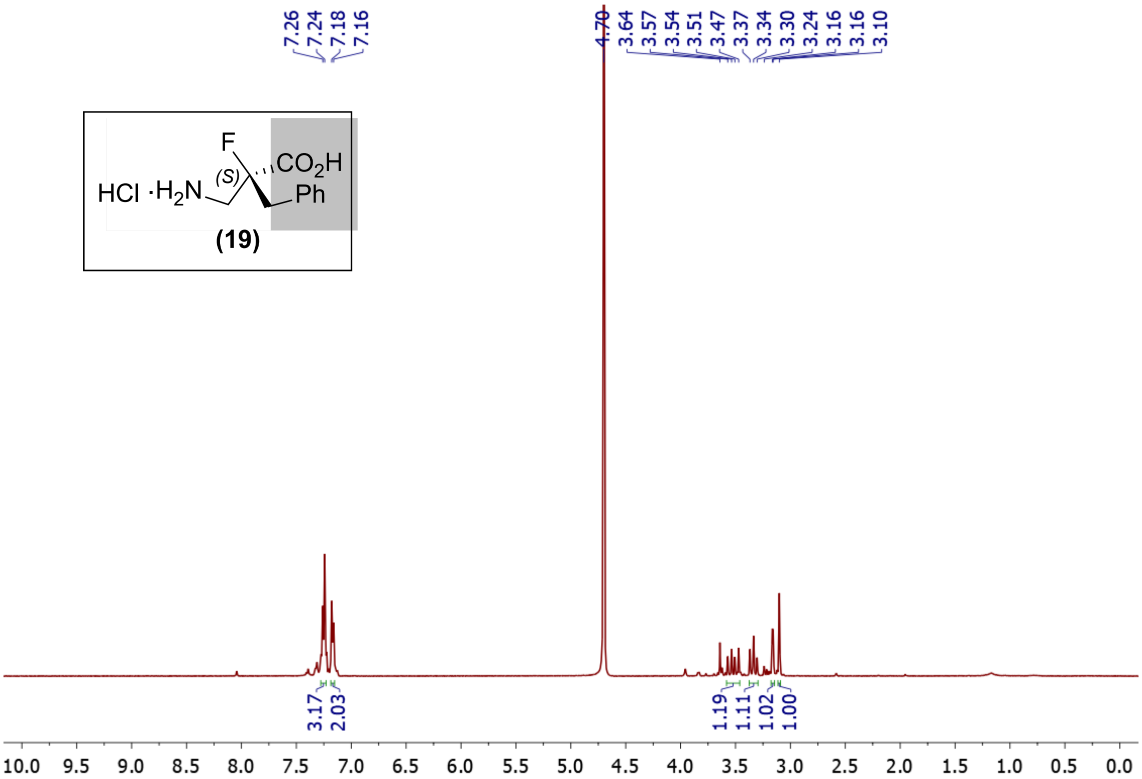

^{13}C{^{1}H} NMR 100 MHz in D_2O

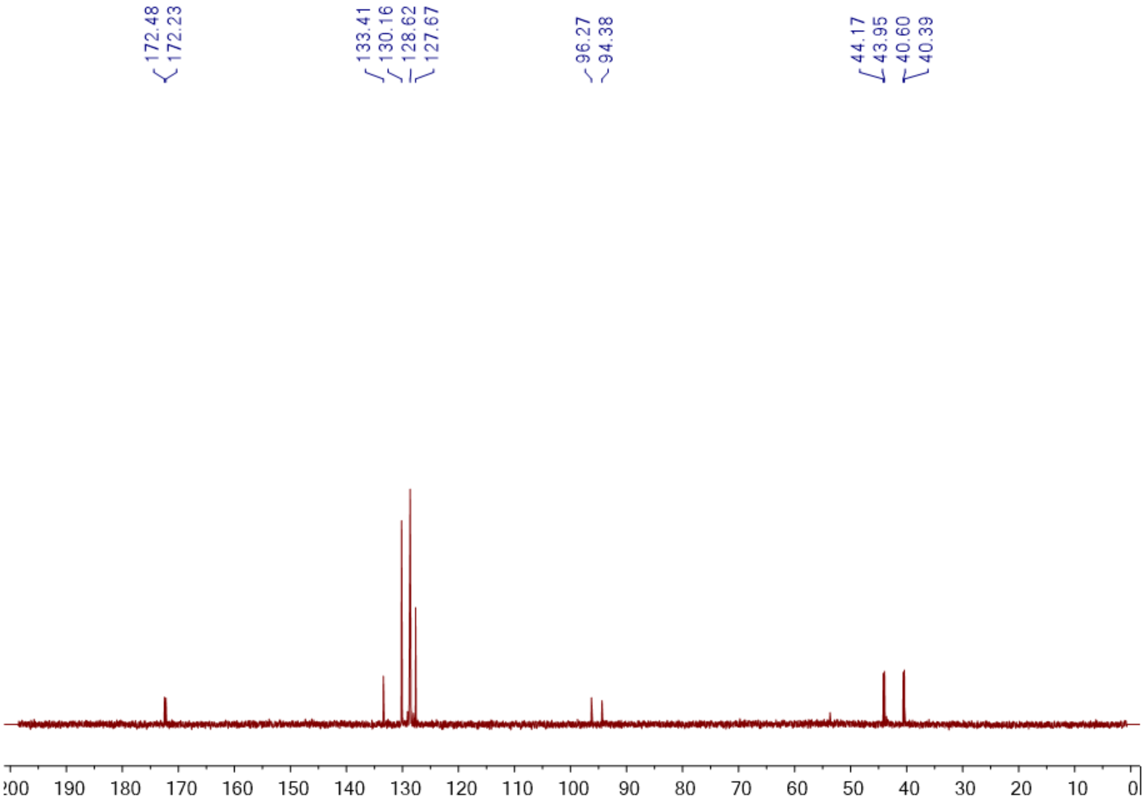

COSY in D_2O

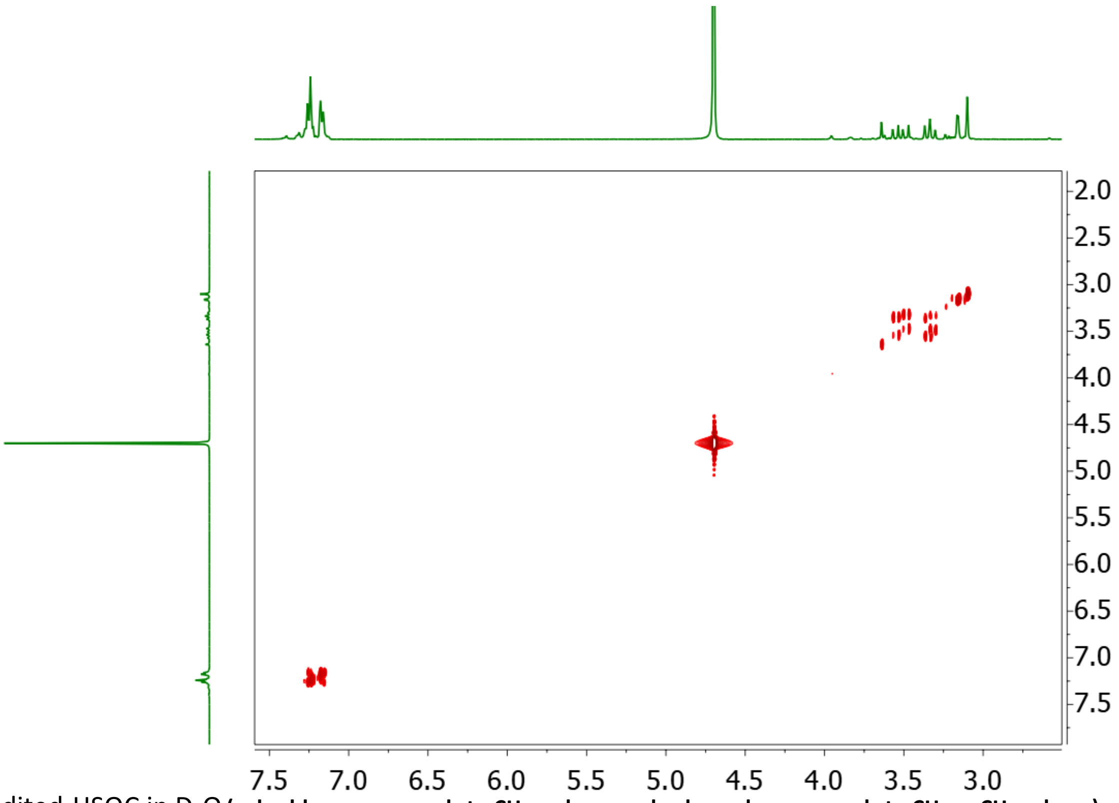

edited-HSQC in D_2O **(color blue corresponds to CH_2 carbons and color red corresponds to CH_3 or CH carbons)**

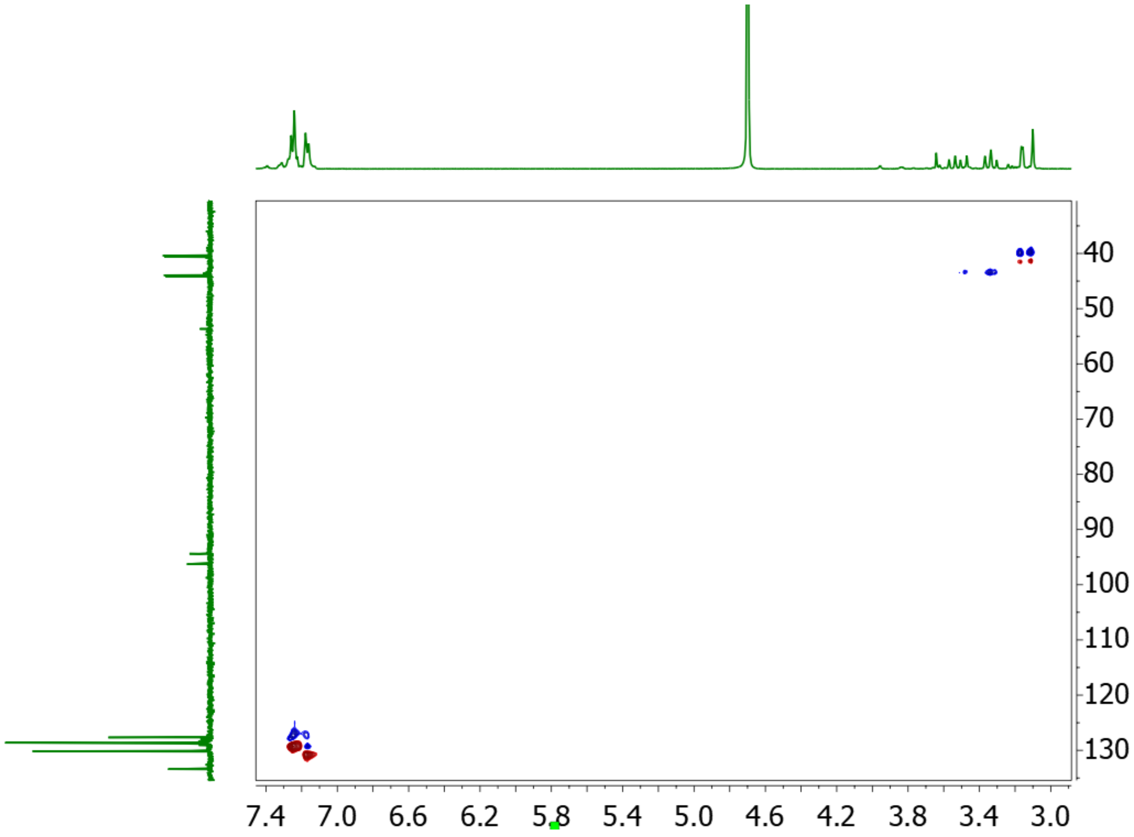

$^{19}F\{^1H\}$ NMR 282 MHz in D_2O

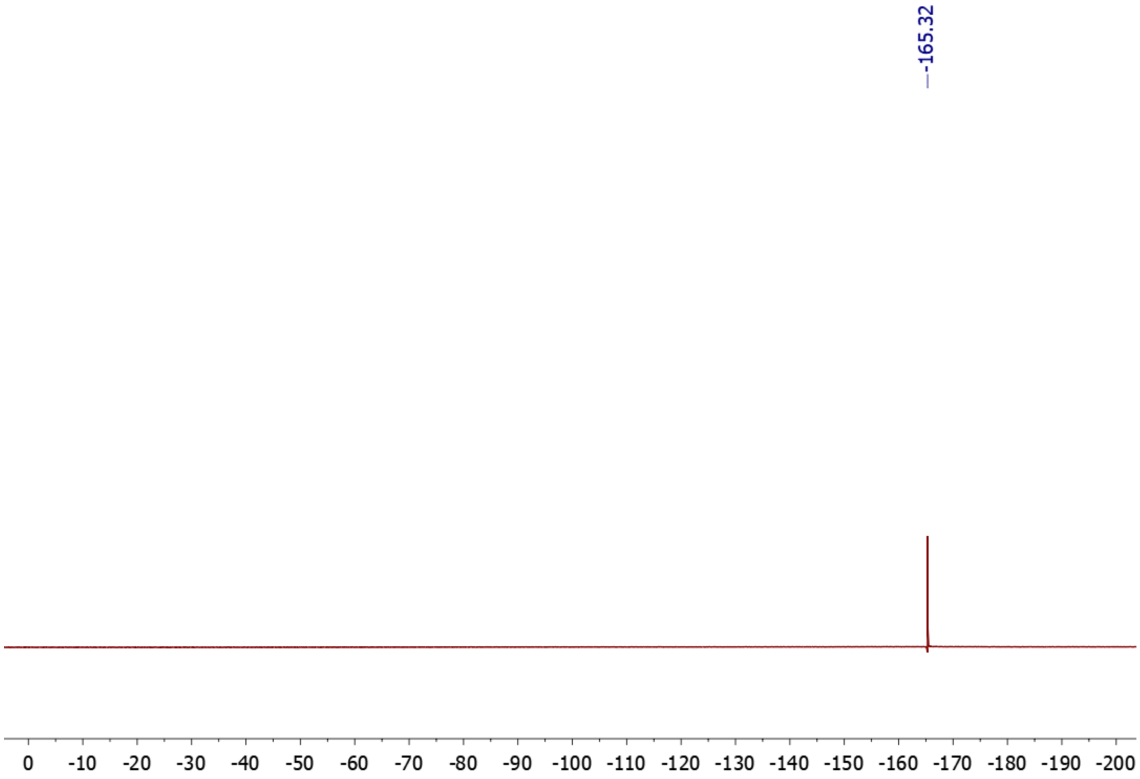

^{1}H NMR 400 MHz in $CDCl_3$

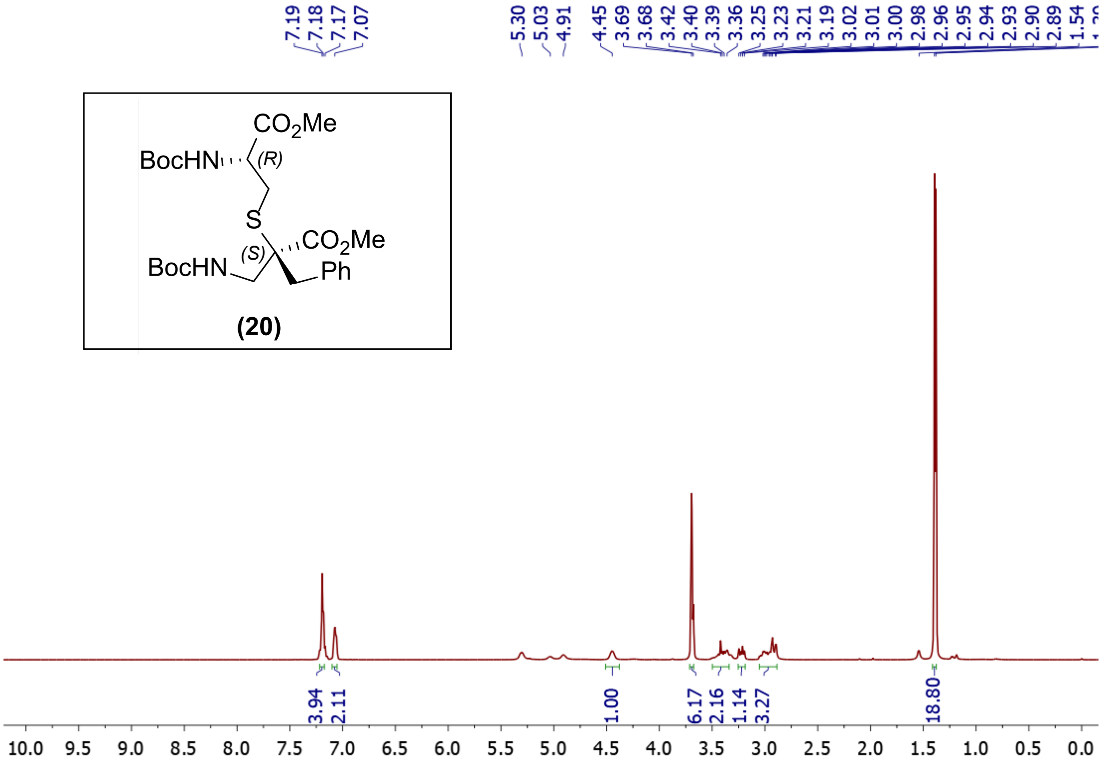

$^{13}C\{^{1}H\}$ NMR 100 MHz in $CDCl_3$

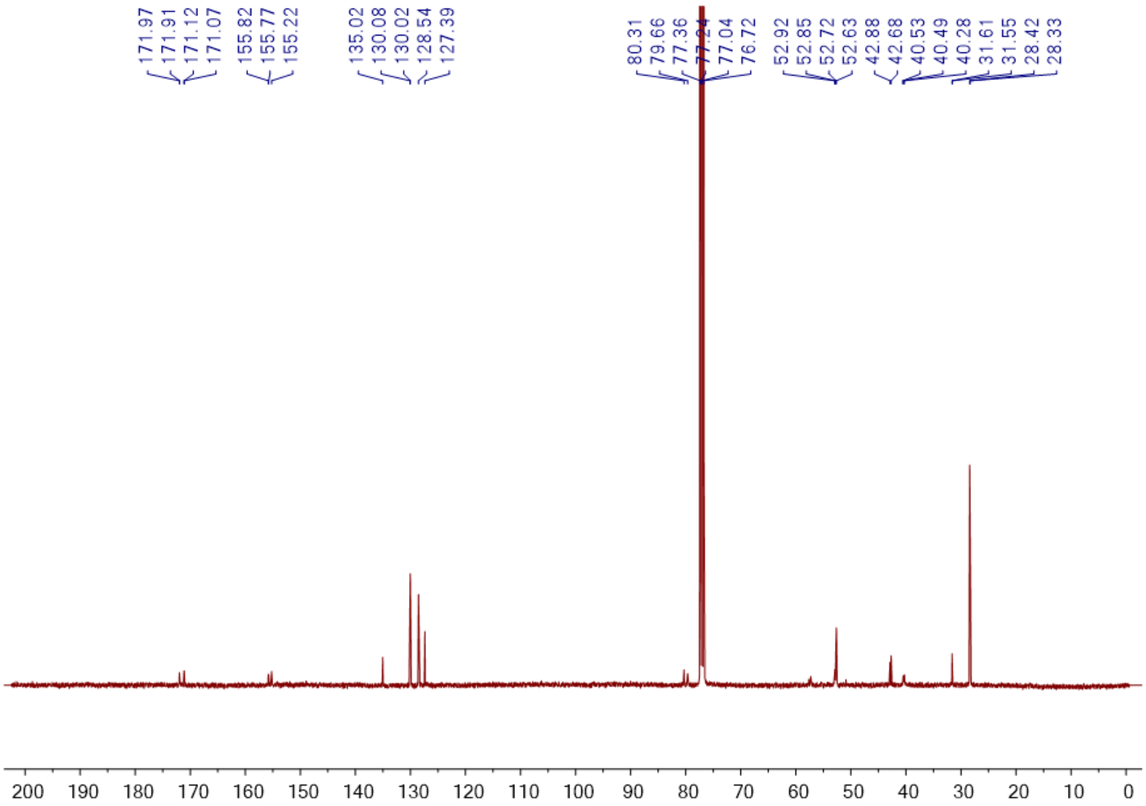

COSY in $CDCl_3$

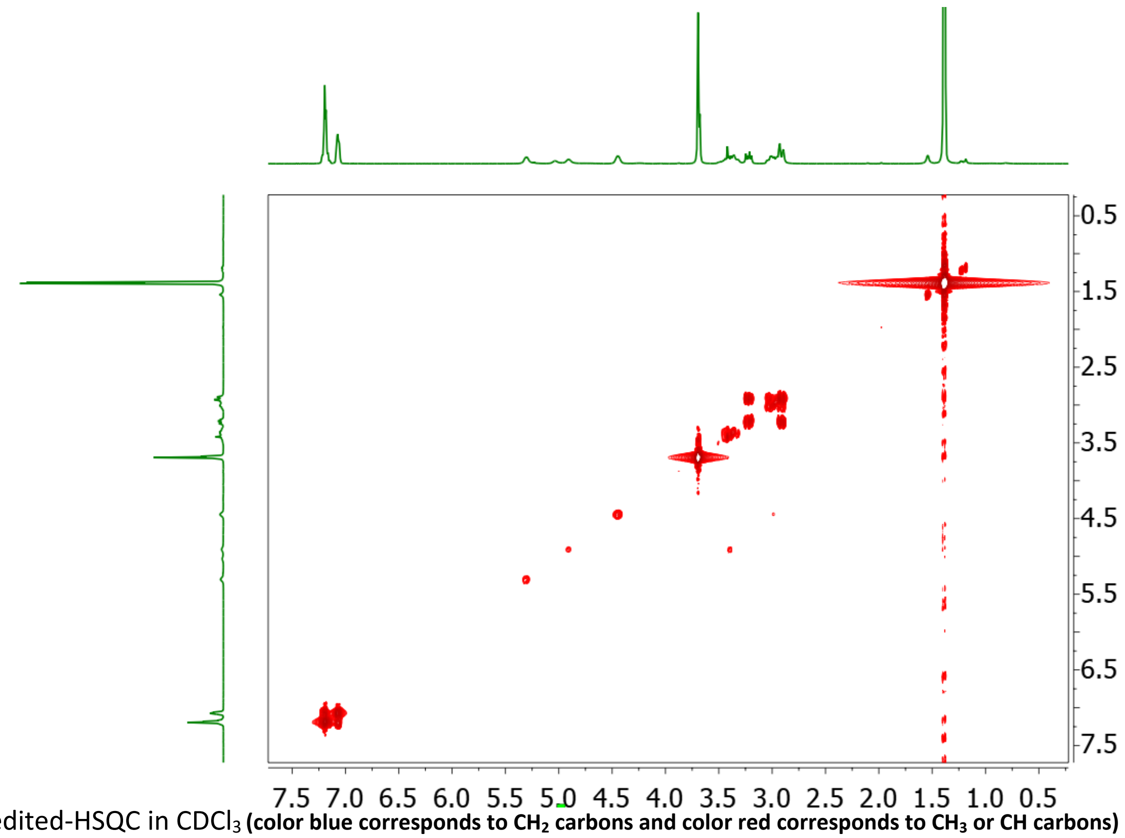

edited-HSQC in $CDCl_3$ **(color blue corresponds to CH_2 carbons and color red corresponds to CH_3 or CH carbons)**

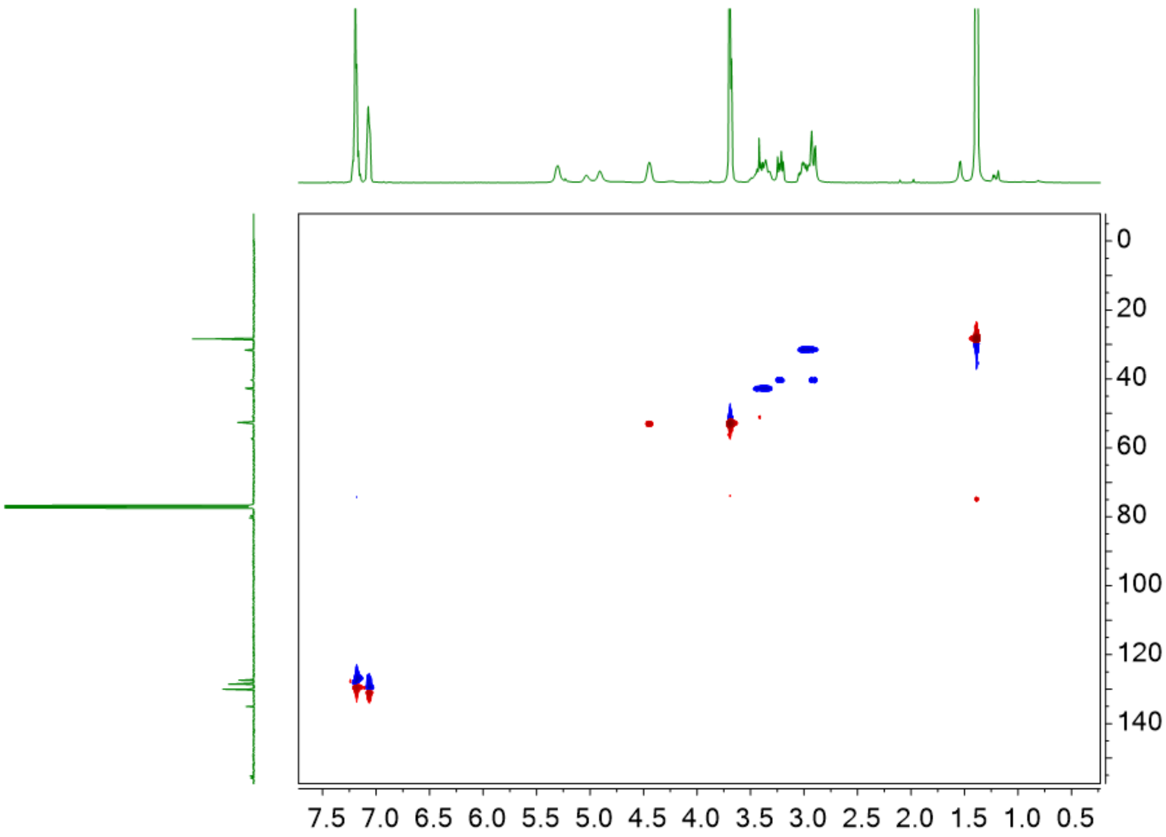

^{1}H NMR 400 MHz in DMF-d7

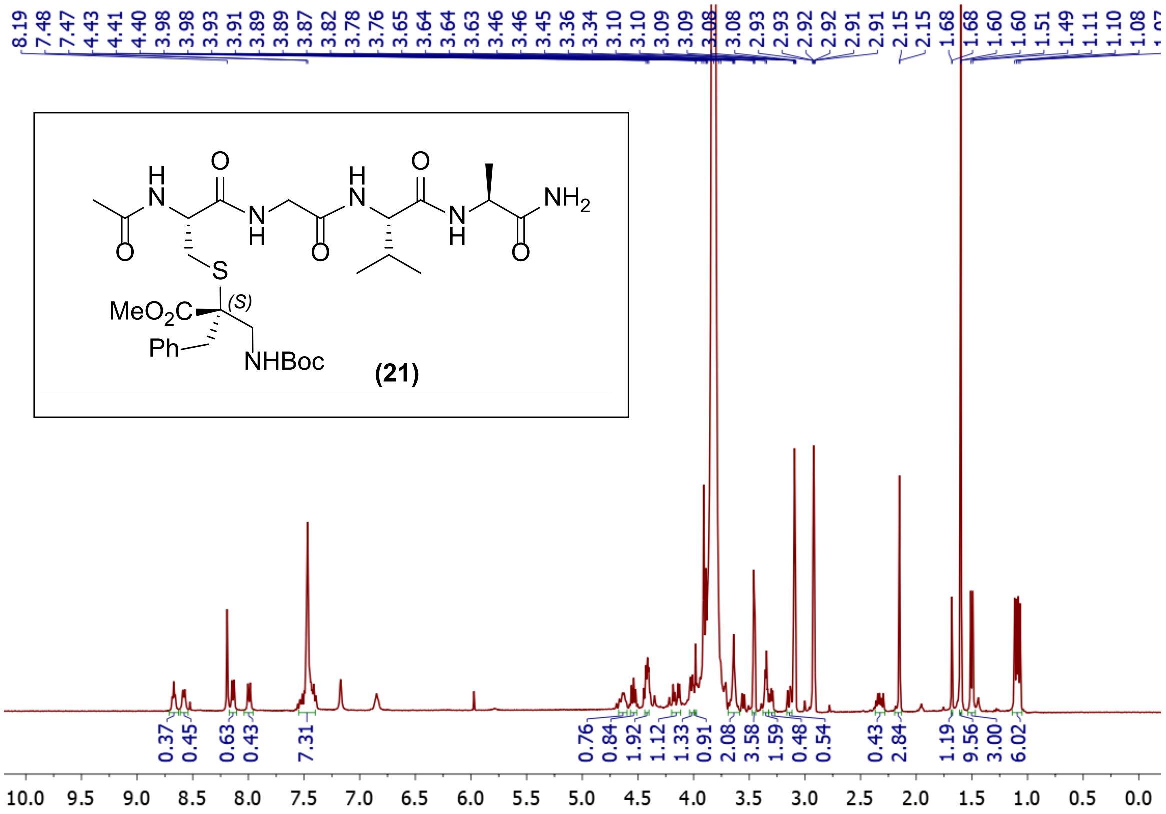

^{13}C{^{1}H} NMR 100 MHz in DMF-d7

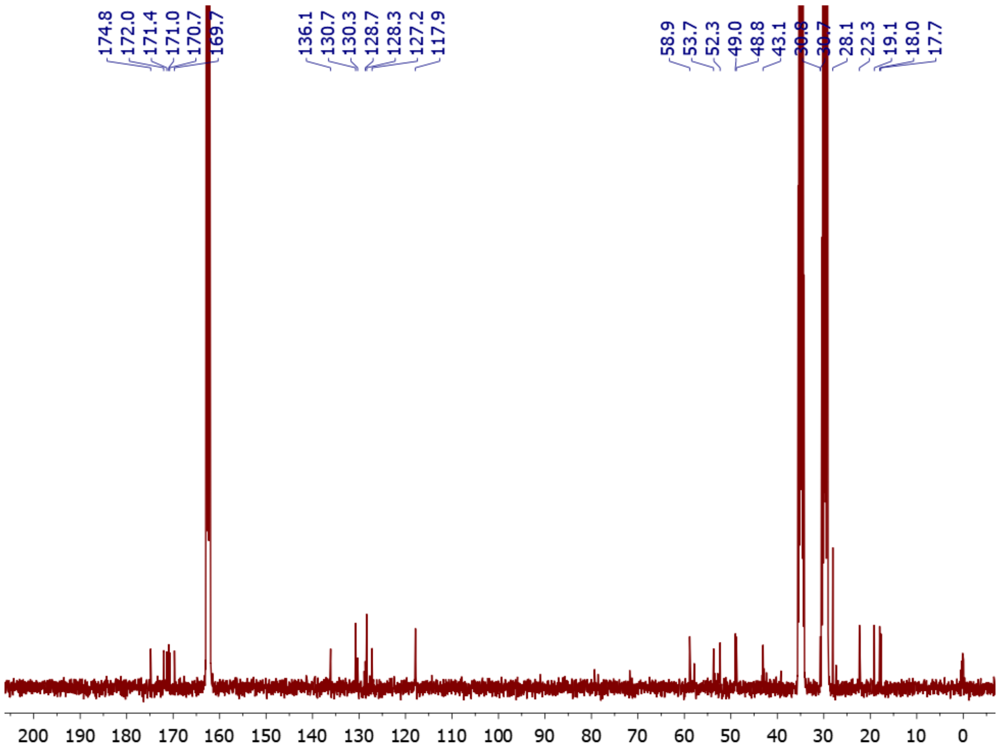

COSY in DMF-d7

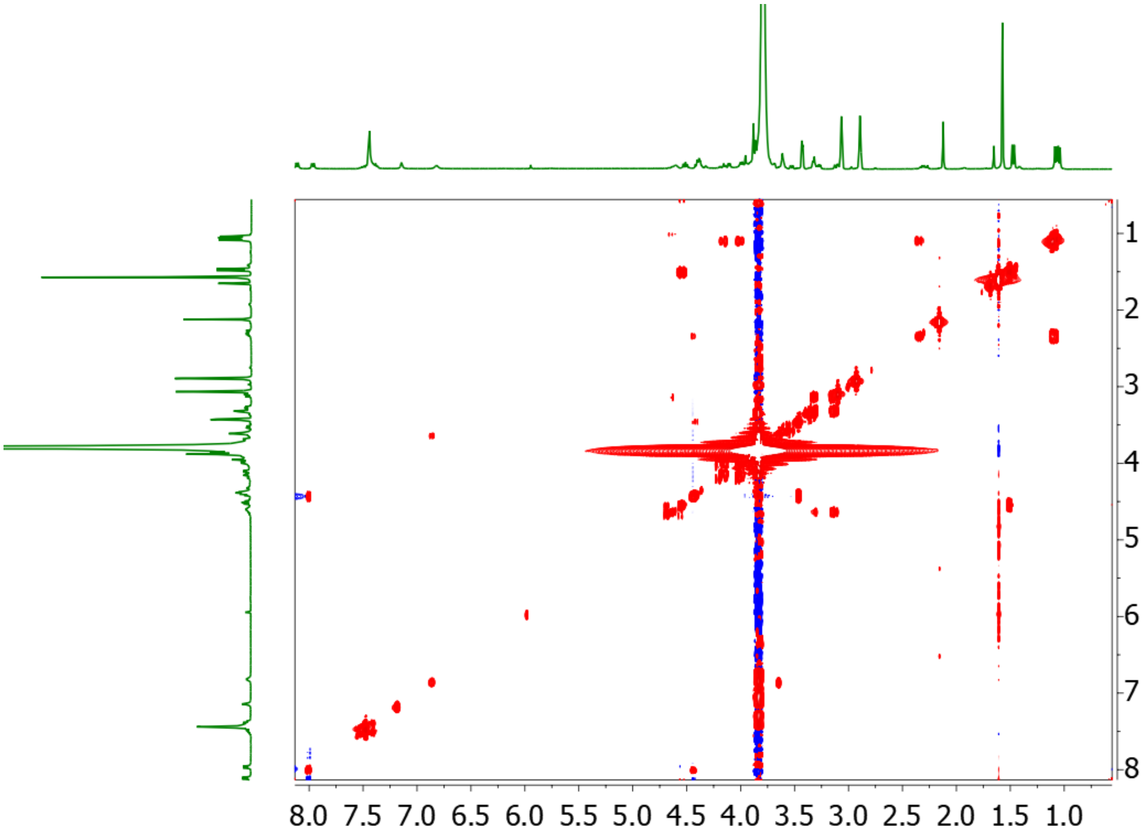

edited-HSQC in DMF-d7 **(color blue corresponds to CH_2 carbons and color red corresponds to CH_3 or CH carbons)**

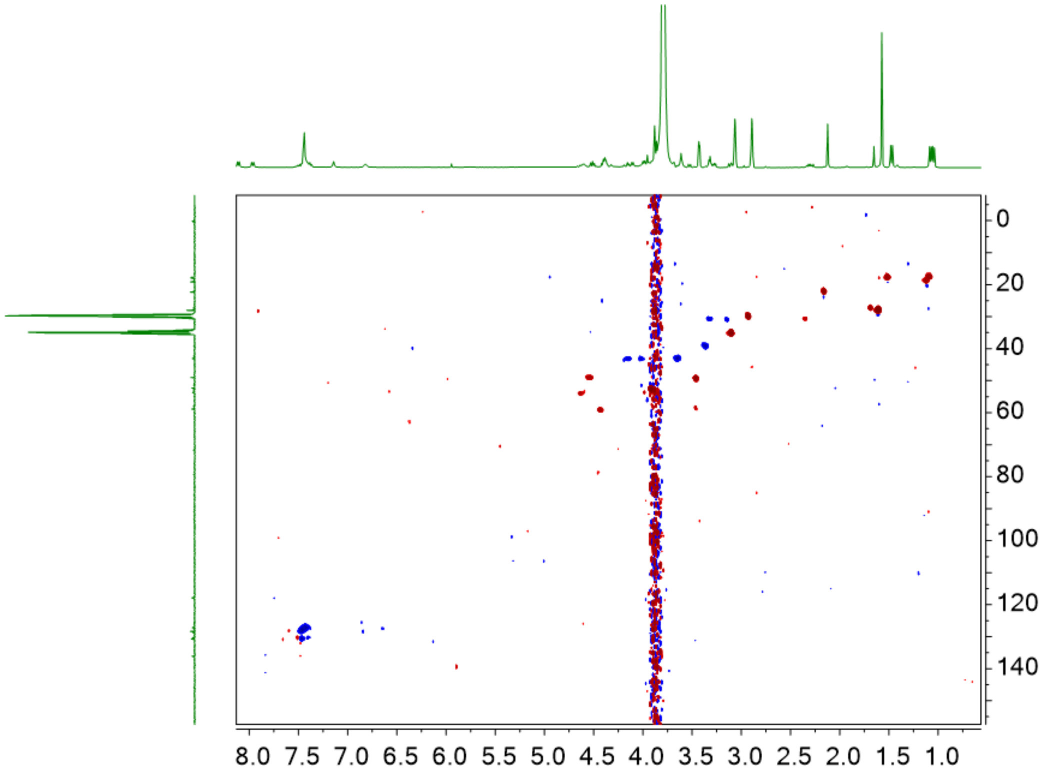

4. Chromatogram for peptide 21

Semi-preparative RP-HPLC purification of peptide **21** using the following conditions:

Phenomenex Luna C18(2) column (10 µm, 250 mm × 21.2 mm) and a dual absorbance detector, with a flow rate of 20 mL/min. Retention time (Rt) = 34.02 min, using a gradient: acetonitrile/water+0.1% TFA (22.5:77.5) → (77.5:22.5), 37 min (λ = 212 nm).

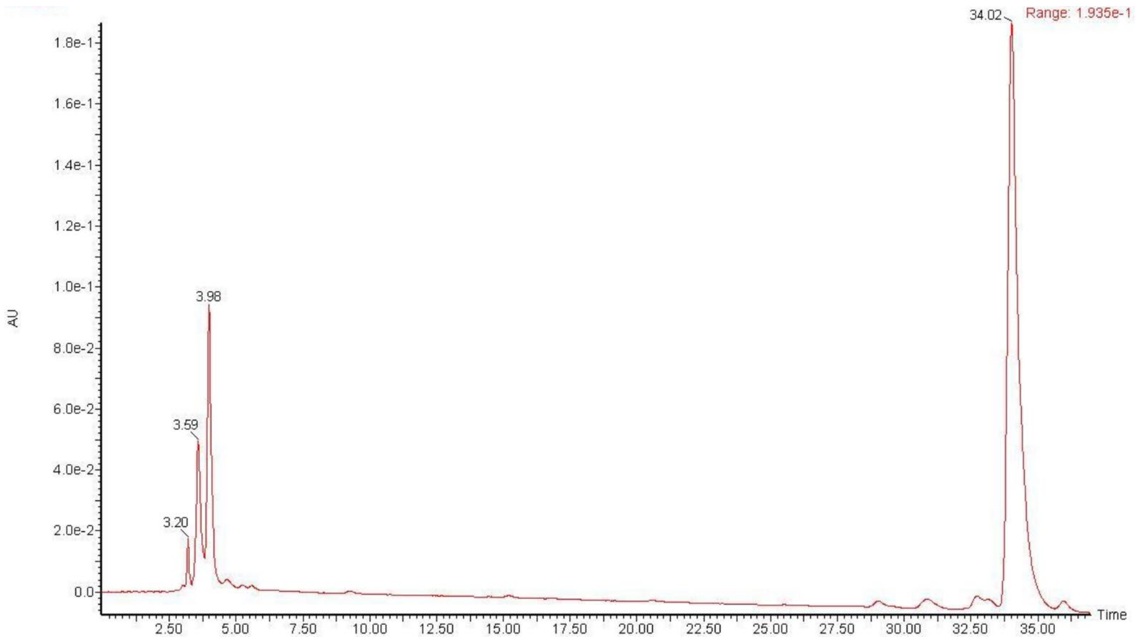

***Figure S11**. Chromatogram of peptide **21**.*

5. Computational details

All possible conformers and ring isomers were investigated. Some structures converged to the same stationary point upon optimization; redundant isomers were discarded and only the unique structures were included in the Boltzmann distribution of Gibbs free energies, summarized in the following table. Bold entries are the minimum energy structures for each diastereomer. The index in last position of each structure names corresponds to the approximate value of dihedral angle formed between Me_7-C_7-O_7-OMe_7.

Table S3. Energies, enthalpies, free energies and entropies of all the conformers of diastereomers I-IV calculated with PCM(toluene)/M062x/6-31+G(d,p).

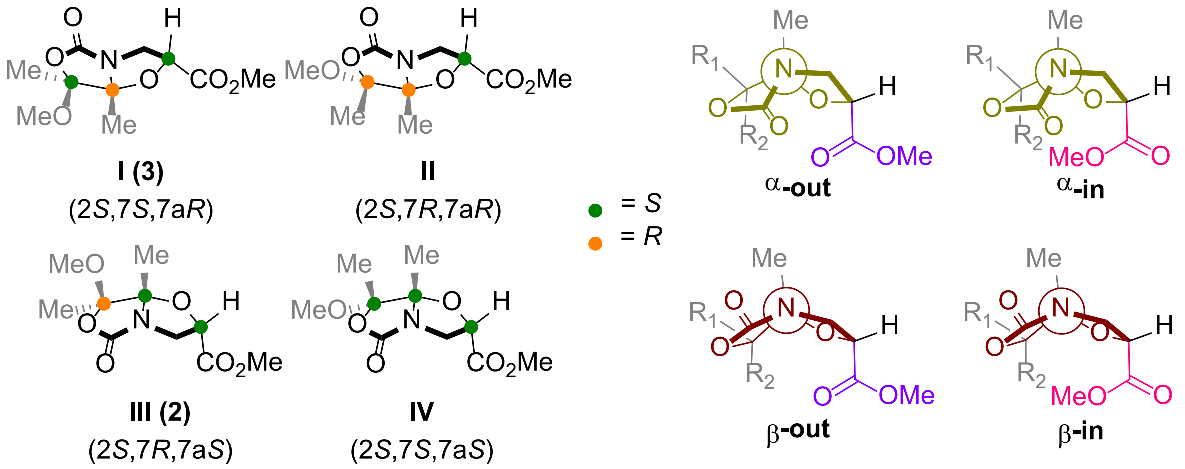

Structure	Eelec (Hartree)[a]	Eelec + ZPE (Hartree)[a]	H (Hartree)[a]	S (cal mol^{-1} K^{-1})[b]	Lowest freq. (cm^{-1})	G (Hartree)[a,b]	G (kcal mol^{-1})[a,b]	*p* (%)	cumulative *p* (%)
**I-α-in-60**	-895.896008	-895.634610	-895.606992	148.3	28.7	-895.695952	5.5	0.0	25.5
**I-α-in-180**	-895.900938	-895.639530	-895.611854	148.4	24.2	-895.700896	2.4	1.6	
**I-α-in-300**	-895.901896	-895.640657	-895.612855	149.6	23.9	-895.702378	1.5	5.3	
**I-α-out-60**	-895.897292	-895.635821	-895.608233	148.5	22.9	-895.697173	4.7	0.1	
**I-α-out-180**	-895.902043	-895.640776	-895.613019	148.7	26.5	-895.702384	1.5	5.3	
**I-α-out-300**	**-895.903045**	**-895.641726**	**-895.613981**	**148.5**	**33.0**	**-895.703487**	**0.8**	**13.1**	
**II-α-in-60**	-895.896364	-895.634918	-895.607300	148.7	25.3	-895.696357	5.3	0.0	0.7
**II-α-in-180**	-895.896428	-895.635212	-895.607408	150.3	23.9	-895.696817	5.0	0.1	
**II-α-in-300**	-895.893544	-895.632030	-895.604432	149.2	21.8	-895.693094	7.3	0.0	
**II-α-out-60**	-895.897451	-895.636006	-895.608387	148.5	27.3	-895.697534	4.5	0.1	
**II-α-out-180**	-895.897857	-895.636362	-895.608733	148.6	25.4	-895.697664	4.4	0.1	
**II-α-out-300**	-895.894893	-895.633425	-895.605824	148.6	25.1	-895.694680	6.3	0.0	
**II-β-in-60**	-895.897568	-895.636318	-895.608583	149.3	23.9	-895.697823	4.3	0.1	
**II-β-in-300**	-895.894820	-895.633599	-895.605920	148.8	29.2	-895.694787	6.2	0.0	
**II-β-out-60**	**-895.898160**	**-895.637036**	**-895.609264**	**149.3**	**28.6**	**-895.698623**	**3.8**	**0.3**	
**II-β-out-300**	-895.895603	-895.634384	-895.606717	149.1	21.1	-895.695565	5.8	0.0	

**III-α-in-60**	**-895.905267**	**-895.643851**	**-895.616333**	**146.5**	**42.1**	**-895.704741**	**0.0**	**36.4**	72.9
**III-α-in-180**	-895.904377	-895.642780	-895.615392	145.3	41.7	-895.703225	1.0	10.6	
**III-α-in-300**	-895.899429	-895.637991	-895.610562	146.1	38.6	-895.698714	3.8	0.3	
**III-α-out-60**	-895.904562	-895.642967	-895.615520	146.4	32.7	-895.703877	0.5	18.0	
**III-α-out-180**	-895.903363	-895.641982	-895.614492	146.2	33.2	-895.702807	1.2	7.5	
**III-α-out-360**	-895.898699	-895.637295	-895.609857	146.4	33.2	-895.698107	4.2	0.2	
**IV-α-in-60**	-895.895947	-895.634462	-895.607036	146.7	31.9	-895.694992	6.1	0.0	0.8
**IV-α-in-180**	**-895.899342**	**-895.637800**	**-895.610273**	**147.3**	**34.3**	**-895.698383**	**4.0**	**0.2**	
**IV-α-in-300**	-895.898115	-895.636612	-895.609161	147.2	30.7	-895.697279	4.7	0.1	
**IV-α-out-60**	-895.892875	-895.631549	-895.604019	148.0	24.2	-895.692288	7.8	0.0	
**IV-α-out-180**	-895.898651	-895.637154	-895.609618	147.4	29.4	-895.697974	4.2	0.1	
**IV-α-out-300**	-895.896251	-895.634948	-895.607360	149.2	24.0	-895.695874	5.6	0.0	
**IV-β-in-60**	-895.899139	-895.637205	-895.610169	142.3	50.9	-895.697136	4.8	0.1	
**IV-β-in-300**	-895.898320	-895.636565	-895.609328	144.1	47.7	-895.697050	4.8	0.1	
**IV-β-out-60**	-895.897193	-895.635188	-895.608222	142.5	46.0	-895.694961	6.1	0.0	
**IV-β-out-300**	-895.898649	-895.637399	-895.609794	148.2	16.5	-895.698367	4.0	0.2	

[a]Energy values calculated with PCM(toluene)/M06-2X/6-31+G(d,p). 1 Hartree = 627.51 kcal mol^{-1}.
[b]Thermal corrections at 388.15 K.

Acid-catalyzed elimination to form enecarbamates 4

Protonation at O1 in all the conformers calculated for **I** (compound **3**) and **III** (compound **2**) led to the spontaneous cleavage of the O1-C7a bond and formation of enammonium cations (**4**$^{\mathbf{H}}$) upon optimization of the corresponding structures. Only one structure of each diastereoisomers (**2**$^{\mathbf{H}}$ and **3**$^{\mathbf{H}}$) kept these atoms bonded to each other. Potential energy scan along the O1-C7a bond revealed a barrierless ($\Delta E^{\ddagger} < 0.2$ kcal mol^{-1}) and highly exergonic process ($\Delta E \approx -15.0$ kcal mol^{-1}) for both diastereomers.

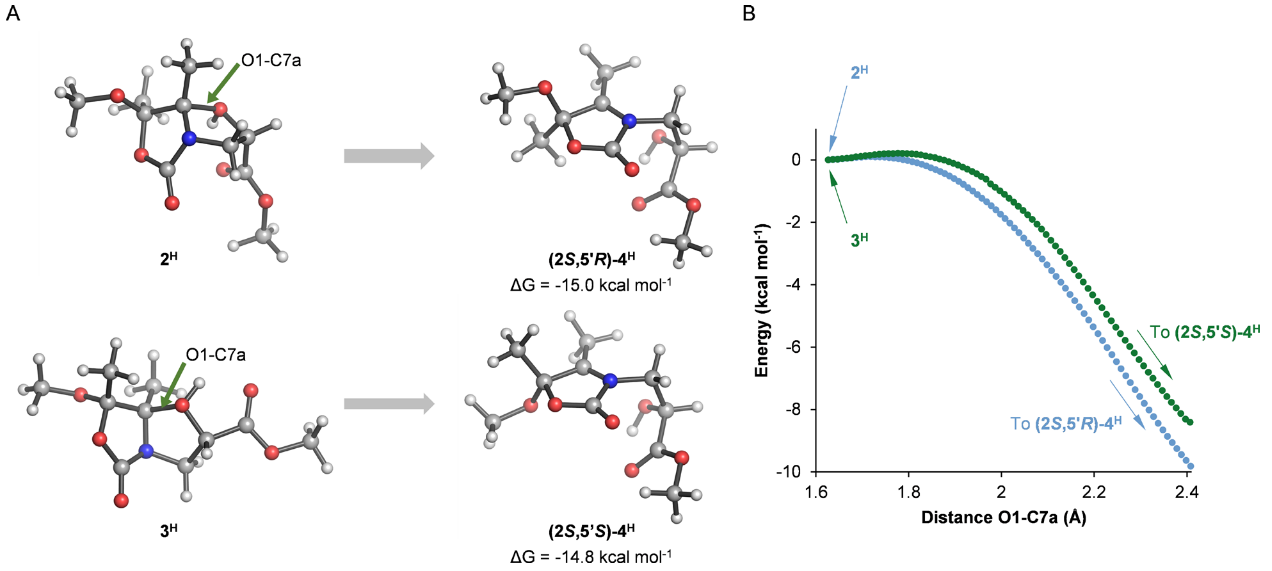

***Figure S12**. A) Lowest-energy structures for the protonated bicyclic scaffolds (**2**$^{\mathbf{H}}$ and **3**$^{\mathbf{H}}$) and the corresponding enammonium cations (**4**$^{\mathbf{H}}$) formed upon cleavage of the O1-C7a bond. B) Potential energy scan along the O1-C7a bond calculated with PCM(toluene)/M06-2X/6-31+G(d,p).*

Table S4. Energies, enthalpies, free energies and entropies of the lowest-energy structures for the protonated compounds 2^{H}-4^{H} calculated with PCM(toluene)/M062x/6-31+G(d,p).

Structure	E_{elec} (Hartree)[a]	E_{elec} + ZPE (Hartree)[a]	H (Hartree)[a]	S (cal mol^{-1} K^{-1})[b]	G (Hartree)[a,b]	Lowest freq. (cm^{-1})	# of imaginary frequencies
**2^{H}**	-896.263391	-895.989469	-895.961270	148.9	-896.051515	43.3	0
**3^{H}**	-896.263619	-895.990495	-895.962321	147.9	-896.052205	45.7	0
**(2*S*,5'*R*)-4^{H}**	-896.286615	-896.012925	-895.983912	152.1	-896.076140	40.5	0
**(2*S*,5'*S*)-4^{H}**	-896.285306	-896.012075	-895.982940	153.1	-896.075134	37.3	0

[a]Energy values calculated with PCM(toluene)/M06-2X/6-31+G(d,p). 1 Hartree = 627.51 kcal mol^{-1}.
[b]Thermal corrections at 388.15 K.

Table S5. Energies, enthalpies, free energies and entropies of the lowest-energy structures for the alkylation reaction of enolates 2' and 3' with bromomethane calculated with PCM(THF)/M062x/6-31+G(d,p).

Structure	E_{elec} (Hartree)[a]	E_{elec} + ZPE (Hartree)[a]	H (Hartree)[a]	S (cal mol^{-1} K^{-1})[b]	G (Hartree)[a,b]	Lowest freq. (cm^{-1})	# of imag freq
**2'**	-895.385429	-895.139171	-895.130778	105.8	-895.162455	32.6	0
**2'_TS_{inv}**	-895.383295	-895.137343	-895.129284	102.7	-895.160568	-49.7	1
**2'_epi**	-895.386465	-895.140164	-895.131624	106.5	-895.163771	44.4	0
**MeBr**	-2611.678064	-2611.640379	-2611.637871	54.8	-2611.654906	619.7	0
**2'_$preTS_{MeBr}$**	-3507.079438	-3506.794090	-3506.783029	131.1	-3506.819923	14.6	0
**2'_TS_{MeBr}**	-3507.072730	-3506.787764	-3506.777132	125.9	-3506.813518	-533.6	1
**5a_epi**	-935.213258	-934.924431	-934.915493	108.1	-934.948056	39.5	0
**2'_epi_$preTS_{MeBr}$**	-3507.082843	-3506.797538	-3506.786279	129.4	-3506.823998	26.7	0
**2'_epi_TS_{MeBr}**	-3507.074239	-3506.789171	-3506.778460	126.9	-3506.814996	-546.4	1
**5a**	-935.210622	-934.922044	-934.912860	110.9	-934.945962	31.1	0
**3'**	-895.386465	-895.140164	-895.131624	106.5	-895.163771	44.4	0
**3'_TS_{inv}**	-895.383295	-895.137343	-895.129284	102.7	-895.160568	-49.7	1
**3'_epi**	-895.385429	-895.139171	-895.130778	105.8	-895.162455	32.6	0
**3'_$preTS_{MeBr}$**	-3507.082843	-3506.797538	-3506.786279	129.4	-3506.823998	26.7	0
**3'_TS_{MeBr}**	-3507.074239	-3506.789171	-3506.778460	126.9	-3506.814996	-546.4	1
**6a**	-935.210622	-934.922044	-934.912860	110.9	-934.945962	31.1	0
**3'_epi_$preTS_{MeBr}$**	-3507.079438	-3506.794090	-3506.783029	131.1	-3506.819923	14.6	0
**3'_epi_TS_{MeBr}**	-3507.072730	-3506.787764	-3506.777132	125.9	-3506.813518	-533.6	1
**6a_epi**	-935.213258	-934.924431	-934.915493	108.1	-934.948056	39.5	0
**Br^-**	-2571.959141	-2571.959141	-2571.957596	36.9	-2571.969074		0

[a]Energy values calculated with PCM(THF)/M06-2X/6-31+G(d,p). 1 Hartree = 627.51 kcal mol^{-1}. [b]Thermal corrections at 195.15 K.

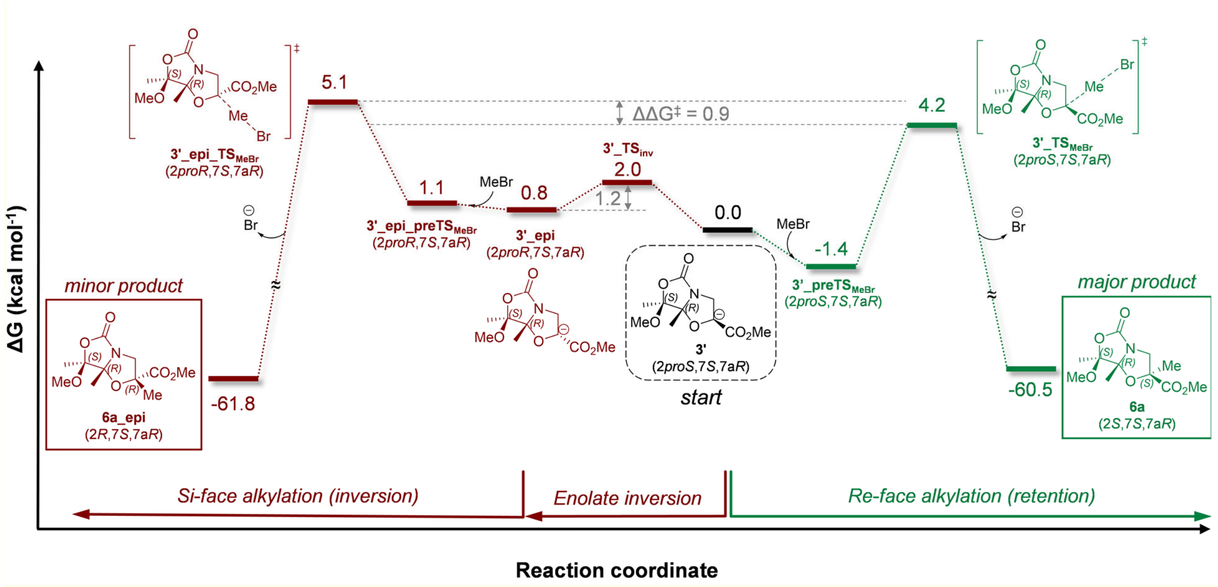

Figure S13. *Minimum-energy pathways for the alkylation reaction of enolate **3'** with bromomethane calculated with PCM(THF)/M06-2X/6-31+G(d,p). Free Gibbs energies (ΔG) calculated at 195 K are given in kcal mol*$^{-1}$.

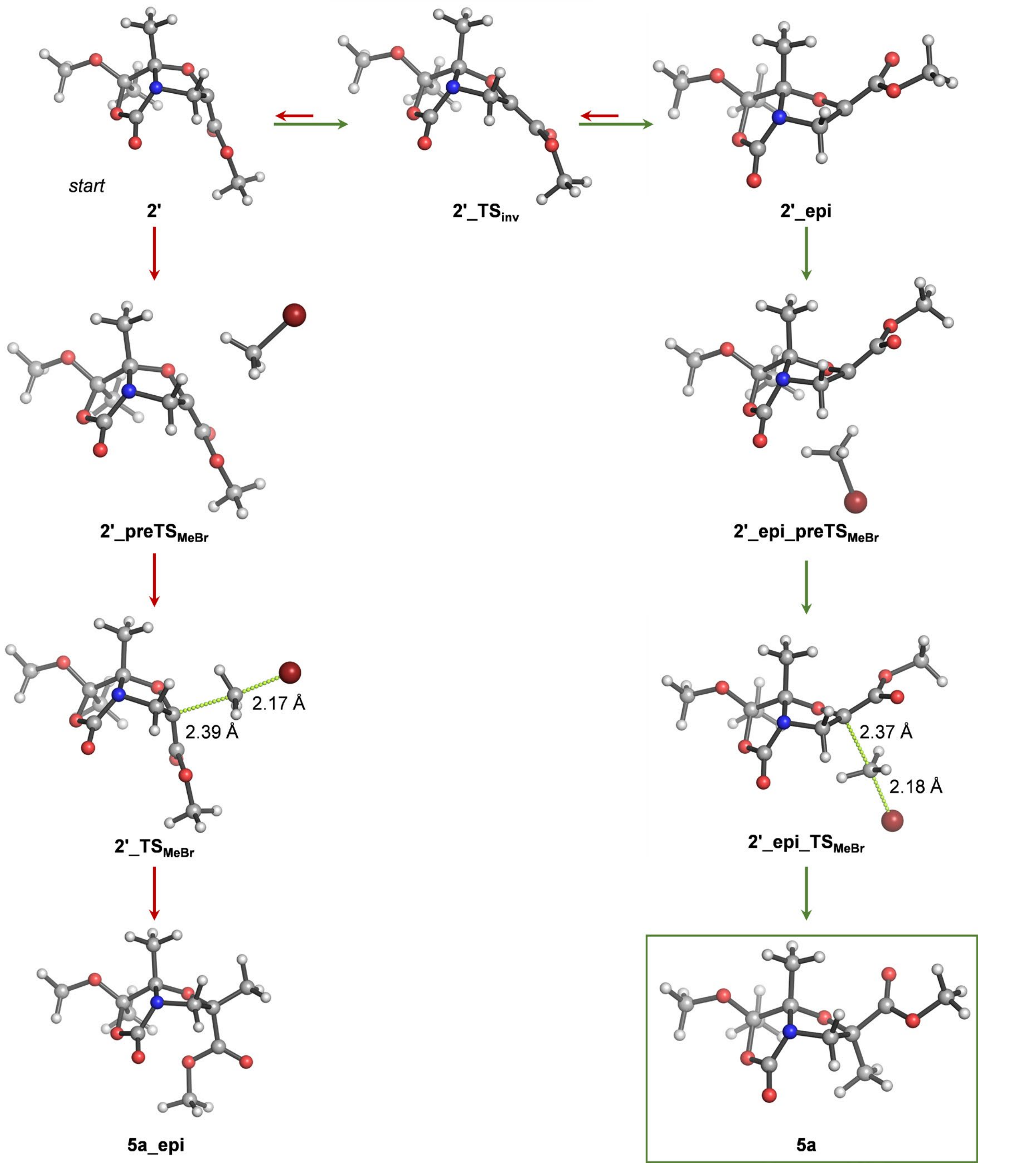

***Figure S14**. Lowest-energy structures for the alkylation reaction of enolates **2'** with bromomethane calculated with PCM(THF)/M06-2X/6-31+G(d,p).*

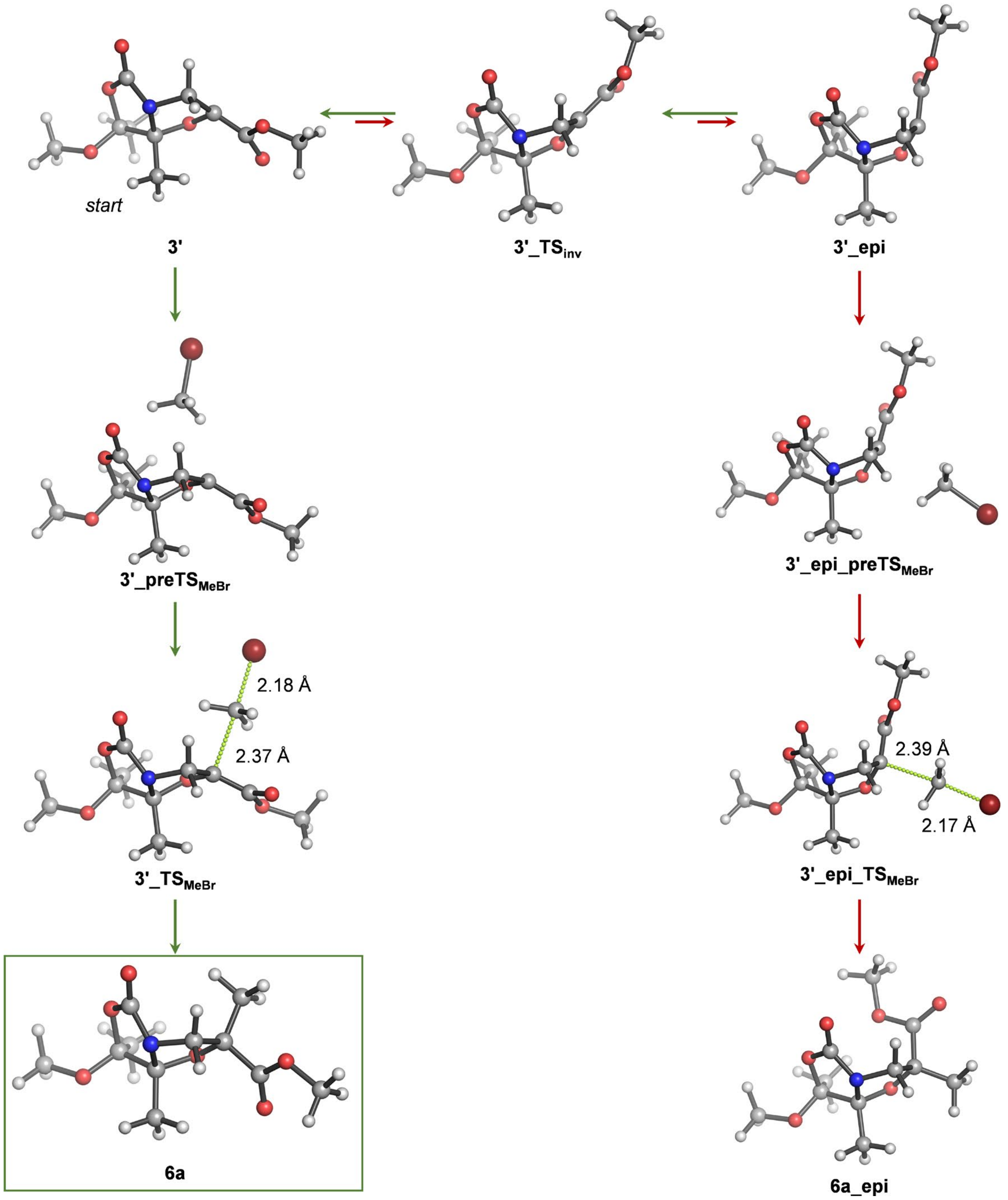

Figure S15. *Lowest-energy structures for the alkylation reaction of enolates **3'** with bromomethane calculated with PCM(THF)/M06-2X/6-31+G(d,p).*

Cartesian coordinates of the lowest-energy calculated structures.

Structure I-α-in-180

O	-0.529474	-0.279341	0.885654
O	2.318519	0.771788	0.662455
O	1.681909	2.747980	-0.210286
N	0.595225	0.773956	-0.786735
O	2.805406	-1.352839	-0.123915
C	1.888629	-0.599742	0.605913
C	0.136469	-1.682750	-0.959260
H	0.882208	-1.856844	-1.734451
H	0.066797	-2.571996	-0.327874
H	-0.841405	-1.514402	-1.414612
C	-0.753147	1.322335	-0.849796
C	1.545280	1.556906	-0.119768
C	0.500586	-0.495890	-0.092594
C	3.324987	-0.784327	-1.322682
H	3.937116	-1.563165	-1.776076
H	2.532110	-0.493477	-2.020194
H	3.947909	0.085497	-1.098811
C	1.845168	-1.173759	2.001740
H	1.495180	-2.207677	1.956863
H	2.856429	-1.157181	2.411263
H	1.172092	-0.592368	2.629830
C	-1.362480	0.796564	0.484504
H	-0.720970	2.409458	-0.907437
H	-1.347138	1.571084	1.256499
H	-1.275380	0.921318	-1.721448
C	-2.811429	0.386263	0.289932
O	-3.733630	1.144373	0.483288
O	-2.942246	-0.853415	-0.178121
C	-4.288447	-1.276987	-0.436900
H	-4.874600	-1.236164	0.482439
H	-4.208720	-2.299585	-0.798685
H	-4.745774	-0.632230	-1.189304

Structure I-α-in-300

O	-0.518740	-0.317141	0.806685
O	2.236931	0.901179	0.502887
O	1.474622	2.882001	-0.250103
N	0.449508	0.887715	-0.862885
O	2.691644	-1.058795	-0.618837
C	1.883241	-0.488920	0.358774
C	0.098926	-1.561434	-1.175435
H	0.803268	-1.606625	-2.004720
H	0.128384	-2.503196	-0.621257
H	-0.918200	-1.419418	-1.546068
C	-0.925029	1.365867	-0.816147
C	1.397328	1.682241	-0.211760
C	0.454792	-0.423831	-0.242506
C	4.080026	-1.132985	-0.312495
H	4.580889	-1.375439	-1.249070
H	4.450318	-0.173241	0.059771
H	4.281069	-1.922726	0.418124
C	1.986211	-1.150770	1.715549
H	1.781044	-2.219680	1.614335
H	2.989103	-1.012976	2.123636
H	1.261617	-0.707676	2.397287
C	-1.425934	0.735463	0.518839
H	-0.950812	2.454723	-0.811283
H	-1.399535	1.465302	1.332944
H	-1.480153	0.987352	-1.677545
C	-2.863065	0.264850	0.385981
O	-3.805418	0.972050	0.659792
O	-2.965821	-0.960452	-0.124328
C	-4.304505	-1.433533	-0.330099
H	-4.840739	-1.458465	0.619834
H	-4.200558	-2.435187	-0.741003
H	-4.829944	-0.779181	-1.027763

Structure I-α-in-60

O	-0.448595	-0.213399	0.835601
O	2.238117	1.135833	0.463572
O	1.356433	3.034956	-0.362851
N	0.454365	0.958495	-0.893741
O	2.891293	-0.755999	-0.538579
C	1.969957	-0.268022	0.395568
C	0.241057	-1.512675	-1.085430
H	0.987533	-1.595843	-1.875979
H	0.234289	-2.420094	-0.475557
H	-0.753098	-1.406577	-1.524110
C	-0.944989	1.360872	-0.869201
C	1.352890	1.835581	-0.277838
C	0.533871	-0.315022	-0.206092
C	3.391722	-2.075104	-0.379428
H	4.029123	-2.251586	-1.245539
H	3.991314	-2.171786	0.530236
H	2.590049	-2.821810	-0.370564
C	2.093832	-0.845889	1.788331
H	1.834438	-1.907315	1.798085
H	3.118147	-0.716449	2.144054
H	1.409558	-0.316800	2.449914
C	-1.415837	0.767500	0.493653
H	-1.030199	2.445717	-0.915703
H	-1.439832	1.535493	1.271795
H	-1.476554	0.913303	-1.712173
C	-2.819311	0.200630	0.378095
O	-3.807414	0.849442	0.633619
O	-2.839218	-1.043284	-0.097762
C	-4.143157	-1.611404	-0.285375
H	-4.675217	-1.644949	0.666634
H	-3.972488	-2.615304	-0.667619
H	-4.712763	-1.014653	-0.999796

Structure I-α-out-180

O	-0.387924	-0.541535	0.883383
O	2.295330	0.843835	0.751452

O	1.396932	2.827671	0.177618
N	0.542301	0.833436	-0.660382
O	3.003093	-1.078867	-0.332594
C	2.029555	-0.549666	0.511081
C	0.356236	-1.617344	-1.149639
H	1.080389	-1.591998	-1.963680
H	0.420834	-2.583848	-0.644321
H	-0.654879	-1.518886	-1.550152
C	-0.863058	1.217997	-0.646100
C	1.408259	1.627553	0.099204
C	0.617504	-0.519785	-0.140347
C	3.405751	-0.297602	-1.453944
H	4.099856	-0.923460	-2.013695
H	2.559970	-0.029012	-2.095770
H	3.916608	0.611968	-1.127429
C	2.101215	-1.310633	1.813225
H	1.874497	-2.363298	1.629321
H	3.116676	-1.227273	2.203732
H	1.385657	-0.907185	2.527876
C	-1.408228	0.379714	0.556218
H	-0.958886	2.292848	-0.497212
H	-1.599493	1.015959	1.425210
H	-1.338653	0.937802	-1.588685
C	-2.698494	-0.327899	0.175309
O	-2.806431	-1.493564	-0.113580
O	-3.704411	0.552587	0.158204
C	-4.980638	0.033738	-0.242550
H	-4.920504	-0.365834	-1.256341
H	-5.666449	0.876608	-0.199321
H	-5.293050	-0.755896	0.442527

Structure I-α-out-300

O	-0.384437	-0.519897	0.817044
O	2.208810	0.987913	0.573651
O	1.217789	2.930987	0.013275
N	0.406233	0.897583	-0.767571
O	2.841687	-0.809646	-0.721686
C	2.002080	-0.415472	0.314145
C	0.297690	-1.542292	-1.273660
H	0.974773	-1.441819	-2.120732
H	0.442839	-2.517353	-0.802230
H	-0.740300	-1.486156	-1.608631
C	-1.011651	1.218299	-0.683112
C	1.272700	1.730962	-0.057129
C	0.562349	-0.452854	-0.257097
C	4.236790	-0.766902	-0.441963
H	4.738517	-0.873702	-1.403034
H	4.516325	0.189632	0.009525
H	4.533378	-1.592581	0.212445
C	2.206653	-1.175478	1.606430
H	2.114986	-2.247743	1.415117
H	3.197861	-0.964279	2.011767
H	1.454252	-0.873732	2.333740
C	-1.458770	0.357217	0.543995
H	-1.148558	2.287692	-0.527203
H	-1.631579	0.985112	1.422976
H	-1.522111	0.917042	-1.600619
C	-2.736425	-0.404298	0.231351
O	-2.815271	-1.578139	-0.032515
O	-3.773950	0.439417	0.245989
C	-5.046916	-0.131231	-0.088491
H	-5.017281	-0.547267	-1.096992
H	-5.760026	0.687694	-0.029176
H	-5.300732	-0.918409	0.623173

Structure I-α-out-60

O	-0.315052	-0.384112	0.867430
O	2.189556	1.249500	0.517436
O	1.079100	3.085490	-0.162941
N	0.402962	0.958351	-0.814865
O	3.014922	-0.496049	-0.616586
C	2.074190	-0.169101	0.366930
C	0.441963	-1.508439	-1.142464
H	1.164172	-1.465045	-1.958436
H	0.552084	-2.443078	-0.586732
H	-0.572437	-1.492885	-1.546973
C	-1.032090	1.199748	-0.757760
C	1.211410	1.889941	-0.158205
C	0.636636	-0.336418	-0.203844
C	3.659138	-1.759896	-0.551541
H	4.281582	-1.815938	-1.444272
H	4.296525	-1.842075	0.333573
H	2.943180	-2.588891	-0.565242
C	2.299056	-0.813772	1.717020
H	2.153788	-1.895502	1.664311
H	3.313633	-0.596668	2.057562
H	1.581777	-0.402628	2.425775
C	-1.439621	0.400819	0.524465
H	-1.231728	2.267609	-0.677172
H	-1.663643	1.075959	1.355342
H	-1.517420	0.807450	-1.654248
C	-2.660315	-0.463061	0.253633
O	-2.654682	-1.652756	0.056246
O	-3.754104	0.304857	0.216220
C	-4.981798	-0.372104	-0.088230
H	-4.916349	-0.842283	-1.070899
H	-5.750820	0.396750	-0.078099
H	-5.184415	-1.132962	0.667107

Structure II-α-in-180

O	-0.347525	-0.442164	0.590352
O	2.488256	0.668637	0.127715
O	1.749066	2.753821	-0.287399
N	0.567769	0.888319	-1.016916
O	2.151210	-1.416150	1.098170
C	2.022716	-0.683733	-0.068898
C	0.030939	-1.494650	-1.533000
H	0.623558	-1.489390	-2.448477

H	0.063338	-2.488693	-1.079159
H	-1.010413	-1.269101	-1.771053
C	-0.767108	1.430863	-0.801364
C	1.614146	1.561746	-0.377391
C	0.545776	-0.479187	-0.532627
C	1.987694	-0.743302	2.348198
H	1.992350	-1.532900	3.099073
H	2.823438	-0.062110	2.523441
H	1.042431	-0.201462	2.395972
C	2.908805	-1.334203	-1.112975
H	3.937725	-1.286225	-0.752976
H	2.626088	-2.379549	-1.248739
H	2.834719	-0.806686	-2.066826
C	-1.183012	0.701964	0.509620
H	-0.727757	2.514144	-0.696821
H	-1.013287	1.338819	1.383962
H	-1.415899	1.162175	-1.638055
C	-2.659842	0.348750	0.491951
O	-3.505566	1.048379	0.999312
O	-2.915955	-0.761716	-0.197011
C	-4.301410	-1.120580	-0.298143
H	-4.719138	-1.275867	0.697752
H	-4.323214	-2.043090	-0.873820
H	-4.856424	-0.330769	-0.807204

Structure II-α-in-300

O	0.333394	-0.429111	-0.593197
O	-2.487282	0.880256	-0.345902
O	-1.663941	2.968666	-0.187821
N	-0.562513	1.161004	0.769548
O	-2.156478	-1.131187	-1.233762
C	-2.070812	-0.451592	-0.025256
C	-0.174594	-1.121863	1.658725
H	-0.820913	-0.953177	2.521286
H	-0.208709	-2.178244	1.380259
H	0.856606	-0.881678	1.926101
C	0.798013	1.614132	0.516767
C	-1.576476	1.791959	0.044785
C	-0.595890	-0.260169	0.481783
C	-1.735433	-2.487350	-1.219924
H	-1.868495	-2.849166	-2.238872
H	-0.678504	-2.570721	-0.948038
H	-2.349829	-3.094738	-0.546097
C	-3.030045	-0.984797	1.026271
H	-4.030868	-0.999405	0.590053
H	-2.761298	-1.992021	1.349693
H	-3.030719	-0.327435	1.898918
C	1.223931	0.673605	-0.648696
H	0.802275	2.668000	0.241449
H	1.122743	1.172124	-1.616742
H	1.413589	1.462961	1.406709
C	2.677548	0.261660	-0.500147
O	3.583883	0.864088	-1.026971
O	2.841383	-0.773296	0.323205
C	4.201266	-1.168887	0.552645
H	4.670073	-1.454808	-0.390197
H	4.148750	-2.017928	1.230479
H	4.760165	-0.346303	1.002041

Structure II-α-in-60

O	0.389541	-0.438454	-0.626541
O	-2.332556	0.871413	-0.258336
O	-1.452191	2.930951	-0.035159
N	-0.406551	1.067016	0.883614
O	-2.023788	-1.294215	-1.012022
C	-1.958838	-0.486644	0.099264
C	0.002815	-1.295405	1.588865
H	-0.570163	-1.177741	2.509397
H	-0.098163	-2.319240	1.219256
H	1.059888	-1.116898	1.793581
C	0.959036	1.500175	0.616085
C	-1.403555	1.745727	0.167489
C	-0.468850	-0.336840	0.513185
C	-3.325848	-1.539506	-1.534014
H	-3.172431	-2.003672	-2.507278
H	-3.889483	-2.224765	-0.892986
H	-3.877653	-0.603175	-1.661071
C	-2.877583	-0.906624	1.236067
H	-3.917453	-0.766244	0.936166
H	-2.712101	-1.955450	1.492846
H	-2.693045	-0.284369	2.114579
C	1.312833	0.637616	-0.631974
H	0.988318	2.570964	0.420378
H	1.190735	1.212812	-1.554303
H	1.597325	1.262834	1.470334
C	2.759916	0.181716	-0.577073
O	3.660000	0.809498	-1.085723
O	2.931171	-0.925182	0.144010
C	4.287802	-1.371821	0.276103
H	4.707105	-1.585009	-0.708406
H	4.240711	-2.275916	0.878996
H	4.888481	-0.605695	0.769390

Structure II-α-out-180

O	-0.188408	-0.652381	0.488287
O	2.416840	0.871983	0.399798
O	1.379108	2.869334	0.370506
N	0.537383	1.055382	-0.818100
O	2.458793	-1.408406	0.826004
C	2.182835	-0.456276	-0.131168
C	0.342455	-1.240933	-1.788091
H	0.918307	-0.962705	-2.671660
H	0.536845	-2.286306	-1.534680
H	-0.724069	-1.147321	-2.002810
C	-0.871352	1.351889	-0.593508
C	1.439019	1.717694	0.025127
C	0.701221	-0.371900	-0.600152
C	1.929555	-1.277414	2.149224

H	2.687449	-1.698492	2.810964
H	1.768110	-0.228956	2.407024
H	0.994756	-1.830088	2.242701
C	3.163915	-0.678948	-1.266125
H	4.170809	-0.552458	-0.865630
H	3.059798	-1.690719	-1.661458
H	2.998811	0.046822	-2.065764
C	-1.246245	0.286282	0.485917
H	-0.992655	2.376122	-0.243960
H	-1.317199	0.741252	1.478721
H	-1.434937	1.211836	-1.518748
C	-2.573657	-0.371712	0.148412
O	-2.713038	-1.438660	-0.396077
O	-3.578601	0.438602	0.494561
C	-4.896101	-0.027963	0.170071
H	-4.987707	-0.169433	-0.908157
H	-5.575188	0.747114	0.517441
H	-5.092233	-0.972434	0.679852

Structure II-α-out-300

O	0.186070	-0.574400	-0.587728
O	-2.403786	1.083984	-0.460196
O	-1.299003	3.044370	-0.434437
N	-0.479918	1.181282	0.687228
O	-2.372542	-1.025313	-1.168573
C	-2.184844	-0.262610	-0.023009
C	-0.392011	-1.078762	1.707337
H	-1.000406	-0.767611	2.557812
H	-0.574282	-2.134739	1.494051
H	0.664716	-0.975522	1.963079
C	0.936029	1.430143	0.452556
C	-1.381289	1.888291	-0.112010
C	-0.701326	-0.238901	0.481417
C	-2.111052	-2.416564	-1.045877
H	-2.337590	-2.846904	-2.020738
H	-1.058589	-2.601087	-0.809729
H	-2.756629	-2.885214	-0.294726
C	-3.207923	-0.560393	1.060923
H	-4.201346	-0.454924	0.620541
H	-3.094548	-1.569967	1.460148
H	-3.103418	0.152556	1.882295
C	1.289252	0.308047	-0.576792
H	1.079996	2.434869	0.056858
H	1.419217	0.720055	-1.581593
H	1.498599	1.323034	1.382897
C	2.566519	-0.408698	-0.170622
O	2.630387	-1.489545	0.361093
O	3.622338	0.363055	-0.445517
C	4.899525	-0.165350	-0.060894
H	4.927165	-0.329539	1.017703
H	5.628354	0.585808	-0.356224
H	5.083197	-1.108218	-0.578272

Structure II-α-out-60

O	0.259273	-0.616917	-0.583148
O	-2.276859	1.015324	-0.366962
O	-1.150212	2.961988	-0.279837
N	-0.363928	1.063271	0.810521
O	-2.226974	-1.223755	-0.948892
C	-2.078422	-0.347110	0.099925
C	-0.245847	-1.278771	1.680007
H	-0.800295	-1.022128	2.583682
H	-0.476342	-2.304677	1.381805
H	0.825396	-1.229597	1.884559
C	1.051183	1.310124	0.565956
C	-1.253319	1.799516	0.015443
C	-0.589002	-0.347138	0.534720
C	-3.542833	-1.352752	-1.477280
H	-3.435554	-1.909104	-2.407459
H	-4.192609	-1.911615	-0.796341
H	-3.975749	-0.370368	-1.689127
C	-3.055061	-0.563740	1.245214
H	-4.066165	-0.320058	0.914647
H	-3.022993	-1.602436	1.582073
H	-2.804142	0.095912	2.078890
C	1.360807	0.267395	-0.556314
H	1.205708	2.340289	0.248161
H	1.445483	0.754280	-1.532305
H	1.627581	1.117903	1.473826
C	2.659068	-0.464248	-0.259004
O	2.756070	-1.562317	0.230167
O	3.697794	0.318410	-0.570554
C	4.994507	-0.221996	-0.281256
H	5.085124	-0.427009	0.786871
H	5.705652	0.540687	-0.590068
H	5.147184	-1.144529	-0.843521

Structure II-β-in-300

O	0.484747	-0.722602	0.296784
O	-2.679623	0.688075	-0.084341
O	-1.651522	-0.894585	-1.274285
O	-1.971474	2.765482	-0.646389
N	-0.613050	1.274758	0.497285
C	0.709889	1.580894	-0.020230
C	-0.694534	-0.146451	0.828714
C	-0.724830	-0.402479	2.321371
H	-1.609215	0.056268	2.769555
H	0.169348	0.037519	2.768446
H	-0.730615	-1.476193	2.524982
C	-1.759761	1.692596	-0.146442
C	-2.920372	-1.549596	0.709086
H	-3.772802	-1.733433	0.051109
H	-3.285641	-1.141860	1.651990
H	-2.409648	-2.494289	0.908355
C	1.119657	0.198752	-0.576709
C	-1.993247	-0.558302	0.042170
C	-1.302744	-2.250174	-1.529921
H	-0.901040	-2.266642	-2.543284

H	-2.184778	-2.897114	-1.486917
H	-0.538503	-2.606248	-0.833213
H	0.765463	0.070264	-1.604588
H	1.377095	1.892155	0.788348
H	0.652197	2.360495	-0.780505
C	2.623914	0.004811	-0.563154
O	3.312244	0.161434	-1.545099
O	3.094277	-0.281483	0.648196
C	4.519147	-0.418628	0.739775
H	4.723314	-0.656912	1.781068
H	4.859549	-1.221973	0.084437
H	5.004712	0.515548	0.452321

Structure II-β-in-60

O	-0.602197	-0.866371	0.016833
O	2.598231	0.507781	-0.394829
O	1.530687	-0.452202	1.405380
O	1.921594	2.656558	-0.653083
N	0.488943	0.867882	-1.002692
C	-0.796522	1.387769	-0.566369
C	0.551707	-0.572240	-0.737868
C	0.520807	-1.400623	-2.006064
H	1.382621	-1.170099	-2.636642
H	-0.394015	-1.158940	-2.551135
H	0.519926	-2.466487	-1.765376
C	1.678803	1.477835	-0.671357
C	2.761435	-1.870825	-0.145290
H	3.711230	-1.747666	0.378373
H	2.976250	-2.003504	-1.205543
H	2.253015	-2.758914	0.238311
C	-1.176818	0.336191	0.501837
C	1.886856	-0.658215	0.075956
C	2.604810	-0.348972	2.335129
H	2.172040	0.054205	3.250097
H	3.377735	0.331982	1.966803
H	3.036941	-1.332020	2.546203
H	-0.760372	0.600278	1.478144
H	-1.512969	1.386853	-1.392769
H	-0.686070	2.396949	-0.168166
C	-2.680216	0.202005	0.645019
O	-3.307259	0.786864	1.498271
O	-3.225848	-0.555669	-0.302695
C	-4.654507	-0.670708	-0.252768
H	-4.923599	-1.326566	-1.077536
H	-4.961007	-1.102331	0.701414
H	-5.114125	0.312093	-0.371700

Structure II-β-out-300

O	0.296520	-0.856657	0.541029
O	-2.579545	0.885747	-0.377656
O	-1.519957	-0.791246	-1.399967
O	-1.569180	2.878269	-0.754547
N	-0.604168	1.248665	0.582643
C	0.814599	1.410877	0.310867
C	-0.892715	-0.159098	0.860325
C	-1.226832	-0.414282	2.315346
H	-2.128698	0.131973	2.601267
H	-0.393135	-0.070396	2.931155
H	-1.377814	-1.482212	2.490698
C	-1.564895	1.787460	-0.247915
C	-3.190821	-1.317964	0.304876
H	-3.922009	-1.410154	-0.501447
H	-3.679589	-0.876377	1.173611
H	-2.824592	-2.310993	0.574570
C	1.165502	-0.009092	-0.185459
C	-2.060321	-0.426995	-0.158865
C	-1.273274	-2.175176	-1.621832
H	-0.705783	-2.229136	-2.551252
H	-2.210701	-2.726349	-1.747339
H	-0.682515	-2.612585	-0.812051
H	0.978744	-0.090512	-1.263525
H	1.358889	1.656488	1.227557
H	0.976052	2.188430	-0.435991
C	2.604045	-0.382267	0.110947
O	2.976830	-1.176979	0.935822
O	3.417103	0.340073	-0.668080
C	4.821131	0.123551	-0.469800
H	5.321730	0.785972	-1.172103
H	5.095806	0.368303	0.557619
H	5.070105	-0.918840	-0.675014

Structure II-β-out-60

O	-0.419536	-1.093594	-0.212594
O	2.551134	0.772475	-0.169022
O	1.417483	-0.391623	1.462145
O	1.586293	2.800608	-0.482579
N	0.507532	0.827605	-1.046527
C	-0.888378	1.127579	-0.771277
C	0.761937	-0.595779	-0.796080
C	1.035676	-1.370529	-2.068750
H	1.928610	-0.985806	-2.567007
H	0.178221	-1.252589	-2.734188
H	1.169444	-2.432099	-1.847651
C	1.534752	1.599845	-0.551767
C	3.046440	-1.560774	0.079361
H	3.887837	-1.311036	0.728295
H	3.417888	-1.627630	-0.943051
H	2.634189	-2.526679	0.381736
C	-1.230101	-0.015602	0.211543
C	1.973711	-0.502072	0.190458
C	2.332097	-0.160639	2.529254
H	1.728043	0.149828	3.381243
H	3.038551	0.634955	2.274641
H	2.872128	-1.076534	2.788286
H	-0.980687	0.276459	1.238085
H	-1.486190	1.058349	-1.684886
H	-0.989010	2.122887	-0.337783
C	-2.688820	-0.418819	0.137617

O	-3.122992	-1.438267	-0.334426
O	-3.443393	0.559348	0.653253
C	-4.859431	0.335446	0.617973
H	-5.307024	1.218356	1.068495
H	-5.194152	0.214839	-0.413763
H	-5.109793	-0.560044	1.189033

Structure III-α-in-180

C	-0.900430	0.901283	-0.254399
N	-0.392160	0.658493	1.079719
C	-0.195307	-0.717975	1.240606
O	0.454159	-1.250231	2.102110
O	-0.868161	-1.391243	0.284976
C	-1.482025	-0.492994	-0.654912
C	-1.883744	2.048857	-0.373989
H	-1.394723	2.974842	-0.061374
H	-2.181055	2.153117	-1.420219
H	-2.775130	1.887888	0.231066
C	-1.142616	-0.921864	-2.061777
H	-1.567741	-1.912749	-2.230649
H	-1.582865	-0.217598	-2.771860
H	-0.061426	-0.940373	-2.191195
O	-2.867797	-0.553006	-0.516932
C	-3.388140	-0.629967	0.807231
H	-4.461957	-0.469318	0.714440
H	-3.198843	-1.616099	1.237999
H	-2.963426	0.137215	1.464565
C	0.776036	1.514248	1.251756
H	0.464420	2.517925	1.544716
H	1.444707	1.102715	2.008659
C	1.364626	1.507874	-0.185623
O	0.262251	1.237290	-1.033456
H	1.821578	2.462351	-0.454308
C	2.432750	0.426560	-0.279982
O	3.596281	0.622383	-0.016882
O	1.912246	-0.758226	-0.590713
C	2.799654	-1.881355	-0.495868
H	3.177199	-1.963800	0.524795
H	2.196771	-2.749334	-0.753788
H	3.630954	-1.759961	-1.191957

Structure III-α-in-300

C	-0.886285	0.867153	-0.112493
N	-0.149459	0.861155	1.133170
C	0.080407	-0.462204	1.517267
O	0.875731	-0.841382	2.336929
O	-0.736322	-1.283929	0.830359
C	-1.512584	-0.567363	-0.134805
C	-1.883850	2.003031	-0.229145
H	-1.362271	2.952713	-0.085577
H	-2.317396	2.010795	-1.232539
H	-2.671903	1.903704	0.517399
C	-1.392613	-1.278895	-1.465076
H	-1.816352	-2.281755	-1.377045
H	-1.910989	-0.728899	-2.254374
H	-0.336167	-1.345361	-1.722518
O	-2.800110	-0.540910	0.414924
C	-3.914719	-0.535436	-0.463888
H	-4.791680	-0.446991	0.177063
H	-3.892482	0.315302	-1.153667
H	-3.982667	-1.465860	-1.034952
C	1.021200	1.706203	0.938411
H	0.761394	2.753813	1.097950
H	1.817288	1.418823	1.626304
C	1.345813	1.431918	-0.556300
O	0.113037	1.035602	-1.133075
H	1.735800	2.313227	-1.069268
C	2.393144	0.330312	-0.640729
O	3.582556	0.546150	-0.640630
O	1.840293	-0.880375	-0.616191
C	2.743522	-1.984686	-0.466247
H	3.308514	-1.871688	0.460573
H	2.113269	-2.870498	-0.425710
H	3.426176	-2.026852	-1.316317

Structure III-α-in-60

C	-0.783428	1.054694	-0.178965
N	-0.159616	0.877241	1.116288
C	-0.117514	-0.483611	1.424884
O	0.549967	-1.002290	2.282055
O	-0.954553	-1.161408	0.614523
C	-1.558049	-0.285156	-0.358932
C	-1.636298	2.302377	-0.293006
H	-1.004929	3.179909	-0.131870
H	-2.059166	2.362302	-1.298811
H	-2.444382	2.285398	0.436313
C	-1.416386	-0.925293	-1.722966
H	-1.880445	-1.913436	-1.721394
H	-1.903098	-0.297069	-2.473786
H	-0.358840	-1.026191	-1.963389
O	-2.871384	-0.034444	0.024278
C	-3.748302	-1.155378	0.012951
H	-4.642215	-0.842008	0.551213
H	-4.022777	-1.431463	-1.009997
H	-3.297099	-2.010426	0.524931
C	1.109457	1.591000	1.076793
H	0.953697	2.648798	1.293955
H	1.808148	1.168223	1.799791
C	1.526660	1.380029	-0.405231
O	0.311823	1.165380	-1.103123
H	2.052214	2.242609	-0.819420
C	2.450431	0.173225	-0.491858
O	3.652807	0.250486	-0.393243
O	1.767796	-0.965508	-0.590457
C	2.532171	-2.172274	-0.461556
H	3.033532	-2.186020	0.507715
H	1.808892	-2.981928	-0.531018
H	3.269673	-2.234786	-1.263191

Structure III-α-out-180

O	-0.125305	1.498756	0.559117
O	1.021626	-1.429460	0.284257
O	3.030682	-0.323441	0.574227
O	-0.406790	-2.012095	-1.356680
N	0.430034	0.150148	-1.206010
C	-0.792423	0.844683	-1.598080
C	1.002265	0.895072	-0.101768
C	1.951441	2.009302	-0.495278
H	2.817096	1.634546	-1.039953
H	1.417327	2.733908	-1.114772
H	2.296348	2.516962	0.408766
C	0.275341	-1.185522	-0.809434
C	1.400937	-0.087880	2.248140
H	1.845839	0.845092	2.603014
H	0.328213	-0.079443	2.437316
H	1.875082	-0.927923	2.758503
C	-1.293184	1.339839	-0.219698
C	1.652941	-0.230390	0.767159
C	3.479848	-0.900863	-0.648022
H	4.550578	-0.703343	-0.694781
H	3.303753	-1.979250	-0.651537
H	2.991842	-0.453939	-1.521881
H	-1.828757	2.290180	-0.286210
H	-0.552302	1.681836	-2.255306
H	-1.476789	0.162471	-2.102373
C	-2.199337	0.291588	0.418174
O	-1.860980	-0.467289	1.292819
O	-3.403821	0.300453	-0.156447
C	-4.312475	-0.715611	0.294667
H	-5.222930	-0.571536	-0.282396
H	-3.884899	-1.702243	0.108546
H	-4.506066	-0.595053	1.361641

Structure III-α-out-360

O	0.010238	1.067463	-1.052972
O	-0.902427	-1.400862	0.702224
O	-2.941506	-0.546900	0.379309
O	0.747491	-1.156646	2.214066
N	-0.230691	0.685058	1.193103
C	0.983460	1.479378	1.047524
C	-0.981310	0.833171	-0.037418
C	-1.939833	2.007561	-0.031711
H	-2.723380	1.862102	0.711888
H	-1.384500	2.920901	0.195969
H	-2.383602	2.125981	-1.023492
C	-0.048413	-0.678134	1.448172
C	-1.574484	-1.161649	-1.575199
H	-2.072222	-0.519637	-2.306391
H	-0.521784	-1.251135	-1.841605
H	-2.040598	-2.149444	-1.575304
C	1.274730	1.286292	-0.460887
C	-1.659953	-0.570699	-0.185195
C	-4.060083	-0.428894	-0.486389
H	-4.929265	-0.354634	0.166956
H	-4.006645	0.470526	-1.109801
H	-4.168677	-1.308873	-1.126820
H	1.749579	2.162074	-0.910545
H	0.779013	2.525930	1.278781
H	1.767865	1.102933	1.704101
C	2.163615	0.065549	-0.674949
O	1.776011	-1.006999	-1.068984
O	3.422976	0.336018	-0.326313
C	4.335484	-0.770651	-0.391402
H	5.294147	-0.379510	-0.058734
H	3.992107	-1.571405	0.265579
H	4.401259	-1.139358	-1.416232

Structure III-α-out-60

O	0.158458	1.233184	-1.000621
O	-1.077466	-1.281137	0.472753
O	-3.001195	-0.091320	0.056625
O	0.468969	-1.315261	2.109477
N	-0.257519	0.685518	1.183862
C	1.028377	1.372314	1.180383
C	-0.912877	1.011573	-0.067796
C	-1.763530	2.264528	-0.015438
H	-2.556526	2.161505	0.723238
H	-1.125451	3.115498	0.235992
H	-2.206815	2.444225	-0.997975
C	-0.219675	-0.703929	1.333512
C	-1.584070	-0.779950	-1.812454
H	-2.076566	-0.065610	-2.477930
H	-0.530346	-0.865148	-2.076551
H	-2.058987	-1.757539	-1.914358
C	1.396655	1.284358	-0.321463
C	-1.697463	-0.298230	-0.382863
C	-3.882515	-1.202055	-0.066628
H	-4.761626	-0.954598	0.527602
H	-4.182902	-1.353829	-1.108039
H	-3.421956	-2.112602	0.327743
H	1.972970	2.148063	-0.662280
H	0.901837	2.409307	1.495243
H	1.733115	0.868547	1.842185
C	2.186985	0.009005	-0.593290
O	1.724439	-0.996760	-1.073355
O	3.450368	0.143300	-0.184571
C	4.266964	-1.031883	-0.299816
H	5.242527	-0.749994	0.089454
H	3.832625	-1.842880	0.287108
H	4.339047	-1.334002	-1.345817

Structure IV-α-in-180

O	0.023863	-0.617555	-1.239086
O	1.063122	1.042640	1.089132
O	1.920055	1.433688	-1.037401
O	-0.458414	0.255120	2.552396

N	0.483365	-1.122424	0.930576
C	-0.701728	-1.909390	0.617717
C	1.119495	-0.817544	-0.334421
C	2.004612	-1.922088	-0.879650
H	2.780750	-2.204038	-0.167405
H	1.394214	-2.800389	-1.104452
H	2.466655	-1.583698	-1.810634
C	0.276707	0.084420	1.615549
C	3.266257	0.320391	0.493932
H	3.627287	1.284331	0.855511
H	3.287353	-0.403205	1.312194
H	3.911620	-0.022149	-0.316679
C	-1.117667	-1.317495	-0.766183
C	1.848241	0.518954	-0.010261
C	0.733312	2.079374	-1.511147
H	1.051923	3.070459	-1.836578
H	0.303762	1.525809	-2.346804
H	-0.006554	2.168093	-0.715443
H	-1.417825	-2.093099	-1.474479
H	-0.442706	-2.965520	0.534188
H	-1.455341	-1.782588	1.396179
C	-2.294982	-0.375650	-0.571626
O	-3.445699	-0.703983	-0.744002
O	-1.905465	0.804100	-0.096542
C	-2.949329	1.701829	0.303911
H	-2.442033	2.587774	0.680048
H	-3.581054	1.945313	-0.551769
H	-3.550808	1.240995	1.089190

Structure IV-α-in-300

O	-0.101917	-0.853257	-1.328309
O	0.988211	0.809273	1.144124
O	-0.704443	0.212258	2.502858
N	0.140445	-1.254280	0.910561
O	1.574773	1.095356	-1.078172
C	1.688704	0.250469	0.002424
C	1.735169	-2.245405	-0.728335
H	2.464908	-2.511469	0.036784
H	2.250568	-2.014056	-1.663909
H	1.080337	-3.101801	-0.905703
C	-1.117563	-1.887500	0.533842
C	0.054481	-0.041134	1.602959
C	0.889157	-1.053897	-0.315667
C	2.216647	2.359247	-0.956519
H	1.842517	2.962823	-1.782679
H	3.304034	2.266741	-1.043880
H	1.956024	2.839863	-0.008433
C	3.119970	0.013222	0.459251
H	3.539061	0.945606	0.841588
H	3.733087	-0.346470	-0.370279
H	3.138009	-0.717788	1.270249
C	-1.379964	-1.233034	-0.850516
H	-1.886117	-1.678543	1.278923
H	-1.856025	-1.918546	-1.554452
H	-0.983313	-2.966285	0.440645
C	-2.295995	-0.030820	-0.667972
O	-3.500125	-0.092860	-0.768718
O	-1.618256	1.049092	-0.294926
C	-2.400526	2.182331	0.099648
H	-3.040232	1.912930	0.942143
H	-1.680323	2.943295	0.392537
H	-3.012072	2.523885	-0.737018

Structure IV-α-in-60

O	0.091144	-0.456888	-1.413899
O	0.851708	0.576055	1.555166
O	-0.841688	-0.510229	2.564700
N	0.196147	-1.377380	0.677764
O	1.326772	1.658141	-0.334311
C	1.606412	0.478672	0.344547
C	2.027142	-1.772265	-0.942560
H	2.727802	-2.112100	-0.178938
H	2.580812	-1.302062	-1.759235
H	1.498874	-2.639193	-1.347231
C	-0.963497	-1.985356	0.039231
C	-0.028892	-0.435102	1.680736
C	1.010965	-0.798093	-0.369918
C	2.007095	1.835508	-1.567052
H	1.593813	2.742146	-2.007994
H	1.824144	0.997781	-2.248114
H	3.083766	1.972016	-1.417171
C	3.069723	0.356240	0.741232
H	3.356165	1.276605	1.254923
H	3.718360	0.208872	-0.124328
H	3.201964	-0.485618	1.424209
C	-1.182713	-1.030514	-1.164585
H	-1.801362	-2.023767	0.736960
H	-1.535985	-1.550728	-2.056674
H	-0.724561	-2.995154	-0.298849
C	-2.212206	0.026771	-0.790838
O	-3.378828	-0.034298	-1.105475
O	-1.679969	0.957402	-0.008582
C	-2.581604	1.919144	0.552076
H	-3.333100	1.410377	1.158686
H	-1.963194	2.568770	1.167392
H	-3.068191	2.482209	-0.245985

Structure IV-α-out-180

O	0.069666	-0.042217	-1.307496
O	1.325464	0.566385	1.388282
O	-0.293143	-0.501735	2.536714
N	0.500395	-1.333904	0.515287
O	2.206743	1.585193	-0.506590
C	2.035214	0.382257	0.137102
C	1.889715	-1.587900	-1.545185
H	2.638953	-2.183871	-1.022992
H	2.374219	-0.987678	-2.319660
H	1.178628	-2.259826	-2.032596

C	-0.775787	-1.824277	0.011746
C	0.430876	-0.419070	1.579352
C	1.148477	-0.666465	-0.595791
C	1.095179	2.475854	-0.675546
H	1.509881	3.480718	-0.584209
H	0.334685	2.311058	0.086779
H	0.649726	2.338139	-1.661605
C	3.422395	-0.136297	0.471066
H	3.897562	0.590847	1.131200
H	4.017744	-0.236307	-0.438106
H	3.362388	-1.101145	0.980144
C	-1.141730	-0.717586	-1.022832
H	-1.494237	-1.920640	0.826244
H	-1.571077	-1.140567	-1.935312
H	-0.641840	-2.792570	-0.471706
C	-2.135620	0.256720	-0.406421
O	-1.851023	1.309896	0.106597
O	-3.370918	-0.249321	-0.463366
C	-4.389780	0.530320	0.180505
H	-4.468535	1.507599	-0.298337
H	-5.311231	-0.034521	0.059344
H	-4.147571	0.659158	1.236705

Structure IV-α-out-300

O	-0.006433	-0.582762	-1.347025
O	1.170871	0.794088	1.180222
O	-0.549882	0.177643	2.496595
N	0.255745	-1.227229	0.829576
O	1.880958	1.164518	-0.994909
C	1.877148	0.252476	0.032909
C	1.752319	-2.148017	-0.943738
H	2.470162	-2.540384	-0.222797
H	2.274044	-1.847290	-1.855886
H	1.043409	-2.937622	-1.203917
C	-1.035210	-1.766596	0.417534
C	0.209067	-0.047669	1.589882
C	0.992108	-0.956428	-0.391342
C	2.603241	2.368694	-0.763071
H	2.321335	3.043107	-1.570491
H	3.684462	2.198735	-0.795928
H	2.320006	2.813529	0.195366
C	3.263099	-0.134362	0.527851
H	3.732320	0.728662	1.003480
H	3.885462	-0.472895	-0.303771
H	3.187253	-0.928967	1.273470
C	-1.287282	-0.959239	-0.883720
H	-1.782622	-1.602728	1.193928
H	-1.804233	-1.551377	-1.643489
H	-0.946891	-2.834486	0.212832
C	-2.112449	0.282936	-0.567442
O	-1.667182	1.388580	-0.395957
O	-3.405771	-0.042166	-0.450601
C	-4.274848	1.024450	-0.044760
H	-4.257097	1.823628	-0.787612
H	-5.267248	0.584663	0.024928
H	-3.954222	1.417358	0.921651

Structure IV-α-out-60

O	-0.156350	0.779561	1.162272
O	-1.037251	-0.716404	-1.514700
O	0.714520	-2.130792	-1.481450
N	-0.284155	-1.400767	0.482886
O	-1.647966	1.362049	-1.003494
C	-1.798550	0.051544	-0.576690
C	-2.023293	-0.467421	1.984184
H	-2.719802	-1.284586	1.793120
H	-2.585796	0.444640	2.199356
H	-1.425664	-0.711160	2.865999
C	0.925776	-1.307210	1.291043
C	-0.108939	-1.470959	-0.903143
C	-1.095185	-0.242837	0.802530
C	-2.290301	2.352215	-0.216876
H	-2.024564	3.307387	-0.668580
H	-1.927570	2.337364	0.815904
H	-3.380545	2.241768	-0.236650
C	-3.236625	-0.437063	-0.665160
H	-3.592437	-0.240724	-1.678712
H	-3.885829	0.071848	0.049763
H	-3.281548	-1.511814	-0.474974
C	1.136462	0.226991	1.317611
H	1.740008	-1.860362	0.822434
H	1.576252	0.571554	2.256688
H	0.741645	-1.701101	2.291801
C	2.030629	0.643478	0.154996
O	1.640608	1.084623	-0.895024
O	3.312276	0.393196	0.448759
C	4.245568	0.634048	-0.614212
H	4.222796	1.687042	-0.899341
H	5.220656	0.362422	-0.216104
H	3.991530	0.016514	-1.477502

Structure IV-β-in-300

O	0.363774	-1.019450	-1.510376
O	1.145441	0.811575	1.334981
O	0.200963	1.386702	-0.692344
O	-0.033718	-0.460601	2.785358
N	0.686714	-1.286087	0.740921
C	-0.390006	-2.189220	0.356116
C	1.272368	-0.710648	-0.465487
C	2.610139	-1.333375	-0.816754
H	3.331975	-1.179829	-0.012209
H	2.463949	-2.406564	-0.956804
H	2.999650	-0.906405	-1.743973
C	0.515791	-0.331214	1.722767
C	2.607618	1.557834	-0.397760
H	2.572087	2.556769	0.040348
H	3.461499	1.036398	0.033801
H	2.740087	1.644298	-1.479016

C	-0.842619	-1.545595	-0.962296
C	1.319800	0.818642	-0.097872
C	-0.027205	2.765759	-0.431730
H	-0.997254	2.993718	-0.873448
H	-0.051276	2.962990	0.645035
H	0.730074	3.392398	-0.912364
H	-1.241321	-2.258099	-1.687429
H	0.002103	-3.193065	0.176331
H	-1.152443	-2.216898	1.133484
C	-1.880064	-0.425386	-0.805911
O	-2.447077	0.087317	-1.739668
O	-2.093861	-0.107326	0.472375
C	-3.022055	0.953544	0.715036
H	-3.023828	1.098970	1.793135
H	-2.695150	1.858507	0.200800
H	-4.013930	0.671650	0.356616

Structure IV-β-in-60

O	-0.353158	0.357839	1.670514
O	-1.060123	-0.488288	-1.609463
O	-0.131056	1.364442	-0.772037
O	0.110197	-2.420425	-1.646190
N	-0.638298	-1.484610	0.340391
C	0.439773	-1.795233	1.271511
C	-1.245145	-0.215310	0.722186
C	-2.592252	-0.389915	1.395815
H	-3.306252	-0.856046	0.713655
H	-2.465402	-1.036187	2.267224
H	-2.983227	0.575383	1.726885
C	-0.444748	-1.553067	-1.025650
C	-2.553383	1.268278	-1.021572
H	-2.419722	1.753711	-1.991085
H	-3.369405	0.549666	-1.100000
H	-2.816789	2.021716	-0.276412
C	0.867813	-0.384137	1.693701
C	-1.267095	0.554850	-0.657835
C	-0.216372	2.672655	-0.213002
H	0.803207	3.056857	-0.205138
H	-0.850017	3.318156	-0.829356
H	-0.586671	2.641574	0.815751
H	1.263292	-0.324357	2.709501
H	0.054105	-2.351525	2.129010
H	1.215753	-2.370487	0.767542
C	1.869970	0.296857	0.753908
O	2.356732	1.376790	0.992484
O	2.118508	-0.421502	-0.338368
C	2.880333	0.232249	-1.360521
H	2.963018	-0.491103	-2.168633
H	2.339874	1.121896	-1.690437
H	3.864158	0.508802	-0.977990

Structure IV-β-out-300

O	-0.057839	-0.809381	-1.528908
O	1.745376	0.799041	1.003822
O	-0.131444	1.414873	-0.200458
O	1.333952	-0.723116	2.632140
N	1.127754	-1.242727	0.380300
C	0.041963	-2.210943	0.346794
C	1.146380	-0.477330	-0.863965
C	2.306764	-0.856181	-1.763367
H	3.258336	-0.643442	-1.270899
H	2.248233	-1.926840	-1.970934
H	2.254795	-0.308065	-2.707052
C	1.372853	-0.434115	1.465811
C	2.051045	1.981068	-1.062005
H	2.006479	2.954932	-0.570730
H	3.092870	1.660717	-1.093501
H	1.666606	2.083908	-2.079085
C	-0.909300	-1.542249	-0.651668
C	1.194546	0.987807	-0.308774
C	-0.390530	2.389638	0.807558
H	-1.328019	2.871567	0.530110
H	-0.493897	1.909877	1.784699
H	0.397709	3.146374	0.855021
H	-1.465894	-2.255719	-1.265115
H	0.393874	-3.173251	-0.031517
H	-0.387836	-2.326903	1.342314
C	-1.916494	-0.619067	0.038718
O	-2.001999	-0.460582	1.234645
O	-2.728375	-0.058597	-0.854552
C	-3.718453	0.827017	-0.321664
H	-4.261260	1.214425	-1.180910
H	-4.387990	0.283042	0.347413
H	-3.238296	1.636691	0.229952

Structure IV-β-out-60

O	0.010335	0.158554	1.592622
O	-1.450843	-0.009070	-1.524104
O	0.126045	1.375289	-0.747663
O	-1.068103	-2.205662	-1.883659
N	-1.128429	-1.313844	0.256942
C	-0.102460	-2.082613	0.950949
C	-1.179465	0.027782	0.820006
C	-2.359006	0.228814	1.751699
H	-3.301789	0.116371	1.212750
H	-2.312946	-0.525980	2.540037
H	-2.318975	1.219509	2.211441
C	-1.176424	-1.280661	-1.125474
C	-2.220738	2.017818	-0.560502
H	-2.090201	2.558396	-1.500903
H	-3.217348	1.576011	-0.548540
H	-2.136747	2.718882	0.272094
C	0.859150	-0.960935	1.353007
C	-1.173253	0.923825	-0.483640
C	0.519189	2.567167	-0.076267
H	1.582862	2.689681	-0.276745
H	-0.018931	3.434682	-0.470648
H	0.371804	2.482489	1.004564

H	1.410775	−1.156722	2.276208
H	−0.521211	−2.575698	1.830820
H	0.338419	−2.813830	0.273788
C	1.858719	−0.657132	0.232584
O	1.972372	−1.315571	−0.773859
O	2.610551	0.403089	0.525390
C	3.521033	0.800562	−0.508075
H	4.024177	1.689697	−0.132633
H	4.240927	0.003932	−0.703338
H	2.964857	1.018691	−1.423034

Structure 2H

O	−0.486571	−0.607761	0.697681
O	2.171731	0.985384	0.666744
O	1.109968	2.918654	0.176237
N	0.434606	0.902138	−0.762766
O	2.946101	−0.699185	−0.709700
C	2.048669	−0.401641	0.294240
C	0.444067	−1.510358	−1.440613
H	1.173284	−1.364478	−2.238498
H	0.615603	−2.494238	−0.997001
H	−0.563228	−1.467039	−1.859112
C	−0.988951	1.195023	−0.785680
C	1.249984	1.741125	0.046772
C	0.676470	−0.429780	−0.425713
C	4.333983	−0.673378	−0.349977
H	4.877998	−0.688399	−1.292245
H	4.572613	0.241356	0.198120
H	4.596611	−1.555793	0.238573
C	2.173996	−1.248225	1.542314
H	2.097569	−2.306332	1.279029
H	3.146646	−1.066725	2.001264
H	1.403072	−0.986612	2.266331
C	−1.495254	0.428657	0.468336
H	−1.148965	2.269057	−0.698655
H	−1.516706	1.059700	1.357542
H	−1.446206	0.831402	−1.707054
C	−2.796702	−0.333740	0.275131
O	−2.793150	−1.548808	0.280346
O	−3.814046	0.459295	0.091128
C	−5.102727	−0.174581	−0.118798
H	−5.056165	−0.797303	−1.011865
H	−5.802659	0.645919	−0.245225
H	−5.352090	−0.776972	0.754145
H	−0.973098	−1.477767	0.638446

Structure 3H

O	0.243091	1.295255	−0.944126
O	−0.999913	−1.353704	0.250514
O	−2.979524	−0.160319	0.238437
O	0.656983	−1.505571	1.780638
N	−0.258216	0.548889	1.194841
C	0.968150	1.317471	1.330856
C	−0.983137	0.973372	0.077466
C	−1.729081	2.271266	0.126218
H	−2.544138	2.161492	0.841996
H	−1.073405	3.087198	0.435180
H	−2.142371	2.498818	−0.857876
C	−0.129808	−0.864792	1.150637
C	−1.816868	−0.567929	−1.864818
H	−2.314292	0.260717	−2.373460
H	−0.827030	−0.743718	−2.292437
H	−2.389450	−1.481022	−2.034024
C	1.459734	1.391592	−0.135825
C	−1.754043	−0.295403	−0.375167
C	−3.892830	−1.256727	0.088570
H	−4.658145	−1.101283	0.845977
H	−4.351420	−1.242435	−0.902785
H	−3.385814	−2.208127	0.267466
H	1.973710	2.317659	−0.393997
H	0.760146	2.308369	1.732645
H	1.675017	0.795977	1.976686
C	2.250625	0.156259	−0.568942
O	1.771670	−0.565318	−1.428140
O	3.373944	0.001128	0.059218
C	4.146919	−1.183264	−0.276070
H	5.024269	−1.135839	0.361544
H	3.547404	−2.067895	−0.063151
H	4.418875	−1.144571	−1.330675
H	0.446183	0.509601	−1.546852

Structure (2S,5'R)-4H

O	−1.208722	2.231831	0.662519
O	1.245461	−1.410057	−0.082413
O	3.211394	−0.232430	−0.434218
O	−0.745262	−1.754087	−1.114333
N	0.147924	0.393201	−0.864140
C	−1.030449	1.075119	−1.408007
C	1.204841	0.900015	−0.325692
C	1.633462	2.303439	−0.271920
H	2.684748	2.344802	−0.573908
H	1.015263	2.958274	−0.881451
H	1.568945	2.639303	0.770138
C	0.139886	−1.072410	−0.716097
C	2.125774	−0.163136	1.776282
H	2.788343	0.656458	2.062576
H	1.132629	−0.004865	2.206190
H	2.519687	−1.104530	2.163308
C	−1.947133	1.440320	−0.224734
C	2.036155	−0.234742	0.261749
C	4.205339	−1.181762	−0.012645
H	4.989755	−1.127686	−0.763967
H	4.608851	−0.905774	0.963967
H	3.783069	−2.189170	0.011186
H	−2.814692	1.976910	−0.622942
H	−0.707236	1.978137	−1.925424
H	−1.516421	0.392281	−2.105976
C	−2.402876	0.159615	0.470570
O	−1.890057	−0.205511	1.508354
O	−3.331719	−0.477957	−0.204309
C	−3.729219	−1.763455	0.319921

H	-4.514941	-2.113695	-0.343522
H	-2.871675	-2.437183	0.302143
H	-4.098440	-1.643589	1.338469
H	-1.266785	1.835589	1.546850

Structure (2S,5'S)-4H

O	-1.002155	2.040492	1.124331
O	1.288418	-1.372609	-0.636405
O	2.309388	-0.368213	1.186106
O	-0.838520	-1.493075	-1.418405
N	0.155646	0.556731	-0.887833
C	-1.047885	1.350364	-1.158336
C	1.287779	0.937460	-0.401244
C	1.742361	2.295466	-0.080851
C	0.113228	-0.910353	-1.017685
C	3.414557	-0.314615	-1.010518
H	3.861788	-1.309322	-0.979144
H	3.173219	-0.077272	-2.048649
H	4.124462	0.417710	-0.619539
C	-1.844047	1.491705	0.152899
C	2.153203	-0.296968	-0.169968
C	3.133857	-1.441048	1.673532
H	3.006825	-1.431596	2.753461
H	2.793306	-2.396539	1.267796
H	4.181632	-1.263203	1.421555
H	-2.701196	2.142549	-0.050941
H	-0.739530	2.330696	-1.521323
H	-1.621281	0.828290	-1.925880
C	-2.343162	0.120289	0.601581
O	-1.834235	-0.457500	1.537281
O	-3.317347	-0.332065	-0.157842
C	-3.777267	-1.669430	0.129816
H	-4.569909	-1.861372	-0.588067
H	-2.951748	-2.370080	-0.001658
H	-4.154085	-1.713931	1.151896
H	-0.984836	1.438796	1.887761
H	0.992000	3.054555	-0.283121
H	1.991075	2.306278	0.986782
H	2.668606	2.493867	-0.631980

Structure 2'

O	0.138091	0.542770	-1.541420
O	-1.107730	-0.621739	1.244700
O	-2.987669	-0.263277	-0.027970
O	0.237501	0.508960	2.635910
N	-0.334088	1.382301	0.562340
C	1.038962	1.834740	0.147660
C	-0.938618	0.807921	-0.642010
C	-1.889948	1.763172	-1.337420
H	-2.723467	2.032163	-0.689650
H	-1.327917	2.659291	-1.612210
H	-2.278358	1.299402	-2.248290
C	-0.342809	0.442191	1.574280
C	-1.234201	-1.751269	-0.859130
H	-1.668191	-1.714528	-1.862540
H	-0.146611	-1.804829	-0.947890
H	-1.605551	-2.634578	-0.334720
C	1.370552	0.790279	-0.862840
C	-1.613850	-0.496768	-0.102100
C	-3.777350	-1.345146	0.449010
H	-4.765730	-0.932366	0.651400
H	-3.865311	-2.134466	-0.304210
H	-3.361951	-1.758917	1.373250
H	0.929223	2.847220	-0.257100
H	1.698292	1.853269	1.014630
C	2.213151	-0.298401	-0.641890
O	2.196660	-1.422961	-1.192090
O	3.243881	0.003808	0.251110
C	4.216660	-1.010053	0.422170
H	4.942590	-0.612544	1.133280
H	3.774319	-1.927653	0.821420
H	4.716110	-1.248044	-0.521990

Structure 2'_TSinv

O	-0.292369	-1.230111	0.217309
O	1.497243	1.014112	-0.918284
O	3.129362	-0.157510	0.205359
O	0.191103	2.723566	-0.273468
N	0.390799	0.810515	1.031565
C	-1.038019	0.670233	1.413612
C	0.844086	-0.543149	0.714291
C	1.391099	-1.288913	1.917287
H	2.221961	-0.749516	2.370781
H	0.584050	-1.412604	2.643933
H	1.729401	-2.281158	1.606812
C	0.639642	1.620266	-0.062157
C	1.853501	-1.266125	-1.557358
H	2.204795	-2.240507	-1.207137
H	0.831853	-1.365284	-1.922267
H	2.495814	-0.922019	-2.370415
C	-1.449092	-0.445052	0.506909
C	1.882874	-0.278371	-0.411698
C	4.219155	0.156456	-0.653559
H	5.046680	0.437337	-0.002343
H	4.513043	-0.710413	-1.253751
H	3.973113	0.997340	-1.309185
H	-1.066401	0.448439	2.488934
H	-1.571697	1.603424	1.232208
C	-2.609780	-0.668972	-0.171506
O	-2.872377	-1.530918	-1.058197
O	-3.644875	0.186009	0.274862
C	-4.599877	0.495589	-0.722387
H	-5.327753	1.163138	-0.256782
H	-4.132682	1.007506	-1.574019
H	-5.103991	-0.402253	-1.088978

Structure 2'_epi

O	0.375272	-0.521911	0.883879
O	-2.310528	0.894919	0.564219
O	-2.728448	-0.851491	-0.874201

O	-1.443079	2.939429	0.230759
N	-0.373718	1.040129	-0.572431
C	1.031662	1.438999	-0.279151
C	-0.503358	-0.378461	-0.217751
C	-0.129828	-1.318061	-1.348761
H	-0.693558	-1.085221	-2.251871
H	0.940882	-1.219511	-1.542901
H	-0.338558	-2.348951	-1.050181
C	-1.365838	1.740149	0.076279
C	-2.217358	-1.324921	1.465379
H	-2.043348	-2.374621	1.214279
H	-1.527838	-1.027161	2.254319
H	-3.242848	-1.203281	1.819759
C	1.520972	0.249649	0.522309
C	-1.979378	-0.468191	0.240369
C	-4.133668	-0.941471	-0.674361
H	-4.576108	-1.019681	-1.667301
H	-4.395368	-1.832171	-0.094291
H	-4.518808	-0.046401	-0.176201
H	1.601002	1.581359	-1.202621
H	0.987461	2.397789	0.249009
C	2.717592	-0.410140	0.309659
O	3.054472	-1.576260	0.627459
O	3.662852	0.424690	-0.304351
C	4.983052	-0.083580	-0.342591
H	5.038752	-1.022050	-0.901511
H	5.585772	0.678230	-0.840241
H	5.371182	-0.258070	0.666369

Structure MeBr

C	0.000000	0.000000	-1.526468
H	-0.000000	1.035642	-1.854660
H	0.896892	-0.517821	-1.854660
H	-0.896892	-0.517821	-1.854660
Br	0.000000	0.000000	0.420651

Structure 2'_preTSMeBr

O	0.238142	-0.513021	-0.517439
O	3.369376	0.190254	0.439701
O	3.460792	-1.918396	-0.470791
O	2.830190	1.411383	2.241113
N	1.411861	-0.248223	1.458976
C	0.120073	0.505089	1.552530
C	1.340129	-1.034635	0.220551
C	1.064252	-2.502678	0.479061
H	1.866398	-2.955585	1.061441
H	0.121890	-2.575871	1.028363
H	0.965377	-3.035805	-0.469772
C	2.549311	0.531829	1.456377
C	2.603278	-0.067700	-1.814143
H	2.217074	-0.784266	-2.544074
H	1.904156	0.769533	-1.752800
H	3.581290	0.294749	-2.138243
C	-0.171186	0.692510	0.108721
C	2.721069	-0.739624	-0.461013
C	4.752263	-1.835486	-1.061404
H	5.265136	-2.760365	-0.797743
H	4.686126	-1.758987	-2.151183
H	5.312302	-0.984052	-0.662477
H	-0.593053	-0.142032	2.083401
H	0.262970	1.429313	2.111591
C	-0.309868	1.848542	-0.625481
O	-0.264621	1.999314	-1.872975
O	-0.630958	2.949302	0.183722
C	-0.779904	4.178159	-0.503149
H	-0.982649	4.927425	0.264067
H	0.129509	4.447011	-1.049247
H	-1.612639	4.144594	-1.212291
C	-2.902007	-0.269581	-0.011118
H	-2.810869	0.271583	-0.947973
H	-2.873970	0.403862	0.839255
H	-2.149423	-1.048527	0.066102
Br	-4.660585	-1.134121	-0.000386

Structure 2'_TSMeBr

O	-0.296432	-0.699831	0.519691
O	-3.191561	0.544611	-0.234869
O	-3.722122	-1.650939	0.194151
O	-2.490910	1.788820	-1.963939
N	-1.435311	-0.207800	-1.429789
C	-0.029801	0.283599	-1.558299
C	-1.450522	-1.063700	-0.243269
C	-1.336943	-2.539450	-0.570869
H	-2.172943	-2.872610	-1.184369
H	-0.396633	-2.697771	-1.105459
H	-1.314423	-3.120930	0.354741
C	-2.375841	0.796820	-1.280319
C	-2.634371	-0.310429	1.927651
H	-2.379002	-1.214080	2.488321
H	-1.830031	0.418750	2.046421
H	-3.567171	0.105571	2.314291
C	0.354689	0.390288	-0.117779
C	-2.784862	-0.654609	0.461191
C	-5.020182	-1.446578	0.739351
H	-5.668633	-2.178557	0.258171
H	-5.026652	-1.616108	1.820521
H	-5.385071	-0.438847	0.518261
H	0.536579	-0.475592	-2.113049
H	-0.011280	1.226739	-2.102949
C	0.384030	1.609258	0.594891
O	0.184390	1.782629	1.808051
O	0.833201	2.653248	-0.190819
C	1.016702	3.889568	0.483921
H	1.386262	4.587498	-0.267569
H	0.073642	4.254419	0.899651
H	1.744342	3.791827	1.293801
C	2.641089	-0.279253	-0.002779
H	2.787129	0.528337	-0.703969

H	2.537719	-0.021083	1.040511
H	2.131248	-1.167943	-0.344859
Br	4.671018	-1.058755	0.032451

Structure 5a_epi

O	0.406531	1.093060	-0.851831
O	-1.358260	-1.208559	0.443179
O	-2.994690	0.326722	-0.060081
O	0.018299	-1.586110	2.182059
N	-0.222410	0.536090	1.273329
C	1.150511	1.001650	1.389929
C	-0.735659	1.030641	0.010669
C	-1.353889	2.412741	0.098389
H	-2.190819	2.419901	0.794869
H	-0.590279	3.121731	0.428549
H	-1.704429	2.717871	-0.890571
C	-0.465600	-0.828720	1.376709
C	-1.588110	-0.556629	-1.850861
H	-1.897890	0.250511	-2.519981
H	-0.548880	-0.815239	-2.050121
H	-2.216231	-1.432129	-2.025341
C	1.617411	1.029370	-0.098191
C	-1.720940	-0.101709	-0.413851
C	-4.057140	-0.585958	-0.322391
H	-4.922410	-0.200928	0.215859
H	-4.286280	-0.626668	-1.391341
H	-3.814581	-1.586158	0.048229
H	1.178631	2.008200	1.814339
H	1.728600	0.327110	2.023789
C	2.321750	-0.297641	-0.397451
O	3.522800	-0.443321	-0.418001
O	1.443660	-1.286580	-0.550081
C	1.984589	-2.610501	-0.660991
H	1.122869	-3.270150	-0.736871
H	2.610339	-2.683851	-1.551611
H	2.573209	-2.844561	0.227629
C	2.514921	2.207519	-0.424131
H	3.421751	2.169709	0.183289
H	2.801491	2.190189	-1.477781
H	1.978011	3.135559	-0.215231

Structure 2'_epi_preTSMeBr

O	0.799347	0.444280	0.531389
O	-0.041817	-2.328571	-0.391497
O	2.009506	-2.927715	0.461788
O	-0.676012	-1.684813	-2.447562
N	1.142456	-0.696354	-1.391943
C	0.906500	0.708041	-1.826731
C	1.540223	-0.649561	0.020403
C	3.030393	-0.452499	0.223680
H	3.600245	-1.212307	-0.310384
H	3.303278	0.541345	-0.139218
H	3.262130	-0.512368	1.290577
C	0.084799	-1.568319	-1.512155
C	0.353941	-1.909085	1.936927
H	1.133481	-1.735278	2.683483
H	-0.356148	-1.083321	1.966179
H	-0.166974	-2.840045	2.169262
C	0.868591	1.433636	-0.499373
C	0.982994	-1.987794	0.561965
C	1.673854	-4.252751	0.856705
H	2.485199	-4.888480	0.502970
H	1.601833	-4.335232	1.945849
H	0.734905	-4.574254	0.395805
H	1.721919	1.071073	-2.461735
H	-0.015702	0.708167	-2.419540
C	1.568781	2.611981	-0.306245
O	1.874134	3.444202	-1.198827
O	1.895400	2.883107	1.018334
C	2.443185	4.168330	1.254031
H	1.741164	4.958842	0.970343
H	2.638549	4.218917	2.326517
H	3.375065	4.316444	0.701060
C	-2.156109	0.339496	0.050229
H	-1.959253	-0.729623	0.055266
H	-1.904241	0.787128	-0.907544
H	-1.630940	0.842117	0.855925
Br	-4.063688	0.572816	0.336376

Structure 2'_epi_TSMeBr

O	-0.513320	0.638881	-0.548451
O	-2.302180	-1.698209	0.114929
O	-3.989230	-0.206309	-0.368851
O	-1.265690	-2.194429	2.045489
N	-1.544370	0.011121	1.365329
C	-0.237340	0.541481	1.813869
C	-1.802570	0.585251	0.043019
C	-2.442790	1.958081	0.092739
H	-3.362380	1.936021	0.676489
H	-1.735650	2.660101	0.542769
H	-2.668230	2.292141	-0.923461
C	-1.660710	-1.361459	1.261979
C	-2.350070	-0.760669	-2.091631
H	-2.675560	0.101361	-2.680001
H	-1.277470	-0.900109	-2.220651
H	-2.871190	-1.653669	-2.441911
C	0.363200	1.019471	0.504879
C	-2.654430	-0.515689	-0.629381
C	-4.946810	-1.139769	-0.856121
H	-5.890620	-0.882978	-0.375821
H	-5.061930	-1.057788	-1.941461
H	-4.669910	-2.163459	-0.586511
H	-0.368630	1.355681	2.534049
H	0.303500	-0.273429	2.309779
C	1.079330	2.220391	0.440519
O	1.713190	2.724201	1.390969
O	1.166840	2.763281	-0.822571
C	2.070730	3.852111	-0.952291

H	3.089320	3.549731	-0.693811
H	2.026440	4.153081	-1.999221
H	1.778860	4.687061	-0.310561
C	2.174860	-0.433089	0.025719
H	2.207520	0.133911	-0.892631
H	2.601380	0.012581	0.912049
H	1.362170	-1.134869	0.147199
Br	3.771020	-1.854549	-0.400731

Structure 5a

O	0.294159	-0.480860	0.798271
O	-2.276921	1.033270	0.353591
O	-2.957571	-0.930320	-0.639439
O	-1.275111	2.847220	-0.522819
N	-0.489821	0.705270	-0.967299
C	0.937269	1.000060	-0.946249
C	-0.664681	-0.552500	-0.258339
C	-0.438101	-1.785110	-1.108039
H	-1.123341	-1.796650	-1.954339
H	0.594909	-1.801820	-1.462729
H	-0.598221	-2.676910	-0.497009
C	-1.338351	1.648990	-0.395859
C	-2.298891	-0.943020	1.712371
H	-2.224121	-2.033340	1.693351
H	-1.534291	-0.540280	2.375371
H	-3.282761	-0.656280	2.087691
C	1.383899	0.346010	0.404881
C	-2.097631	-0.401010	0.314721
C	-4.349121	-0.843540	-0.342789
H	-4.868301	-1.085160	-1.269349
H	-4.632471	-1.565550	0.428621
H	-4.619421	0.168570	-0.027889
H	1.427739	0.547050	-1.810269
H	1.103059	2.077660	-0.962959
C	2.623959	-0.517820	0.176521
O	2.671919	-1.721070	0.264601
O	3.661109	0.250231	-0.167499
C	4.889709	-0.439879	-0.443609
H	4.749329	-1.132029	-1.275349
H	5.608249	0.333881	-0.702869
H	5.214859	-0.988989	0.441231
C	1.626079	1.377470	1.502701
H	1.826729	0.874930	2.452251
H	2.471829	2.018630	1.248501
H	0.729459	1.993720	1.615311

Structure 3'

O	-0.375272	0.521911	-0.883879
O	2.310528	-0.894919	-0.564219
O	2.728448	0.851491	0.874201
O	1.443079	-2.939429	-0.230759
N	0.373718	-1.040129	0.572431
C	-1.031662	-1.438999	0.279151
C	0.503358	0.378461	0.217751
C	0.129828	1.318061	1.348761
H	0.693558	1.085221	2.251871
H	-0.940882	1.219511	1.542901
H	0.338558	2.348951	1.050181
C	1.365838	-1.740149	-0.076279
C	2.217358	1.324921	-1.465379
H	2.043348	2.374621	-1.214279
H	1.527838	1.027161	-2.254319
H	3.242848	1.203281	-1.819759
C	-1.520972	-0.249649	-0.522309
C	1.979378	0.468191	-0.240369
C	4.133668	0.941471	0.674361
H	4.576108	1.019681	1.667301
H	4.395368	1.832171	0.094291
H	4.518808	0.046401	0.176201
H	-1.601002	-1.581359	1.202621
H	-0.987461	-2.397789	-0.249009
C	-2.717592	0.410140	-0.309659
O	-3.054472	1.576260	-0.627459
O	-3.662852	-0.424690	0.304351
C	-4.983052	0.083580	0.342591
H	-5.038752	1.022050	0.901511
H	-5.585772	-0.678230	0.840241
H	-5.371182	0.258070	-0.666369

Structure 3'_TS$_{inv}$

O	0.292369	1.230111	-0.217309
O	-1.497243	-1.014112	0.918284
O	-3.129362	0.157510	-0.205359
O	-0.191103	-2.723566	0.273468
N	-0.390799	-0.810515	-1.031565
C	1.038019	-0.670233	-1.413612
C	-0.844086	0.543149	-0.714291
C	-1.391099	1.288913	-1.917287
H	-2.221961	0.749516	-2.370781
H	-0.584050	1.412604	-2.643933
H	-1.729401	2.281158	-1.606812
C	-0.639642	-1.620266	0.062157
C	-1.853501	1.266125	1.557358
H	-2.204795	2.240507	1.207137
H	-0.831853	1.365284	1.922267
H	-2.495814	0.922019	2.370415
C	1.449092	0.445052	-0.506909
C	-1.882874	0.278371	0.411698
C	-4.219155	-0.156456	0.653559
H	-5.046680	-0.437337	0.002343
H	-4.513043	0.710413	1.253751
H	-3.973113	-0.997340	1.309185
H	1.066401	-0.448439	-2.488934
H	1.571697	-1.603424	-1.232208
C	2.609780	0.668972	0.171506
O	2.872377	1.530918	1.058197
O	3.644875	-0.186009	-0.274862
C	4.599877	-0.495589	0.722387

H	5.327753	-1.163138	0.256782
H	4.132682	-1.007506	1.574019
H	5.103991	0.402253	1.088978

Structure 3'_epi

O	-0.138091	-0.542770	1.541420
O	1.107730	0.621739	-1.244700
O	2.987669	0.263277	0.027970
O	-0.237501	-0.508960	-2.635910
N	0.334088	-1.382301	-0.562340
C	-1.038962	-1.834740	-0.147660
C	0.938618	-0.807921	0.642010
C	1.889948	-1.763172	1.337420
H	2.723467	-2.032163	0.689650
H	1.327917	-2.659291	1.612210
H	2.278358	-1.299402	2.248290
C	0.342809	-0.442191	-1.574280
C	1.234201	1.751269	0.859130
H	1.668191	1.714528	1.862540
H	0.146611	1.804829	0.947890
H	1.605551	2.634578	0.334720
C	-1.370552	-0.790279	0.862840
C	1.613850	0.496768	0.102100
C	3.777350	1.345146	-0.449010
H	4.765730	0.932366	-0.651400
H	3.865311	2.134466	0.304210
H	3.361951	1.758917	-1.373250
H	-0.929223	-2.847220	0.257100
H	-1.698292	-1.853269	-1.014630
C	-2.213151	0.298401	0.641890
O	-2.196660	1.422961	1.192090
O	-3.243881	-0.003808	-0.251110
C	-4.216660	1.010053	-0.422170
H	-4.942590	0.612544	-1.133280
H	-3.774319	1.927653	-0.821420
H	-4.716110	1.248044	0.521990

Structure 3'_preTS$_{MeBr}$

O	-0.799347	-0.444280	-0.531389
O	0.041817	2.328571	0.391497
O	-2.009506	2.927715	-0.461788
O	0.676012	1.684813	2.447562
N	-1.142456	0.696354	1.391943
C	-0.906500	-0.708041	1.826731
C	-1.540223	0.649561	-0.020403
C	-3.030393	0.452499	-0.223680
H	-3.600245	1.212307	0.310384
H	-3.303278	-0.541345	0.139218
H	-3.262130	0.512368	-1.290577
C	-0.084799	1.568319	1.512155
C	-0.353941	1.909085	-1.936927
H	-1.133481	1.735278	-2.683483
H	0.356148	1.083321	-1.966179
H	0.166974	2.840045	-2.169262
C	-0.868591	-1.433636	0.499373
C	-0.982994	1.987794	-0.561965
C	-1.673854	4.252751	-0.856705
H	-2.485199	4.888480	-0.502970
H	-1.601833	4.335232	-1.945849
H	-0.734905	4.574254	-0.395805
H	-1.721919	-1.071073	2.461735
H	0.015702	-0.708167	2.419540
C	-1.568781	-2.611981	0.306245
O	-1.874134	-3.444202	1.198827
O	-1.895400	-2.883107	-1.018334
C	-2.443185	-4.168330	-1.254031
H	-1.741164	-4.958842	-0.970343
H	-2.638549	-4.218917	-2.326517
H	-3.375065	-4.316444	-0.701060
C	2.156109	-0.339496	-0.050229
H	1.959253	0.729623	-0.055266
H	1.904241	-0.787128	0.907544
H	1.630940	-0.842117	-0.855925
Br	4.063688	-0.572816	-0.336376

Structure 3'_TS$_{MeBr}$

O	0.513320	-0.638881	0.548451
O	2.302180	1.698209	-0.114929
O	3.989230	0.206309	0.368851
O	1.265690	2.194429	-2.045489
N	1.544370	-0.011121	-1.365329
C	0.237340	-0.541481	-1.813869
C	1.802570	-0.585251	-0.043019
C	2.442790	-1.958081	-0.092739
H	3.362380	-1.936021	-0.676489
H	1.735650	-2.660101	-0.542769
H	2.668230	-2.292141	0.923461
C	1.660710	1.361459	-1.261979
C	2.350070	0.760669	2.091631
H	2.675560	-0.101361	2.680001
H	1.277470	0.900109	2.220651
H	2.871190	1.653669	2.441911
C	-0.363200	-1.019471	-0.504879
C	2.654430	0.515689	0.629381
C	4.946810	1.139769	0.856121
H	5.890620	0.882978	0.375821
H	5.061930	1.057788	1.941461
H	4.669910	2.163459	0.586511
H	0.368630	-1.355681	-2.534049
H	-0.303500	0.273429	-2.309779
C	-1.079330	-2.220391	-0.440519
O	-1.713190	-2.724201	-1.390969
O	-1.166840	-2.763281	0.822571
C	-2.070730	-3.852111	0.952291
H	-3.089320	-3.549731	0.693811
H	-2.026440	-4.153081	1.999221
H	-1.778860	-4.687061	0.310561
C	-2.174860	0.433089	-0.025719

H	-2.207520	-0.133911	0.892631
H	-2.601380	-0.012581	-0.912049
H	-1.362170	1.134869	-0.147199
Br	-3.771020	1.854549	0.400731

Structure 6a

O	-0.294159	0.480860	-0.798271
O	2.276921	-1.033270	-0.353591
O	2.957571	0.930320	0.639439
O	1.275111	-2.847220	0.522819
N	0.489821	-0.705270	0.967299
C	-0.937269	-1.000060	0.946249
C	0.664681	0.552500	0.258339
C	0.438101	1.785110	1.108039
H	1.123341	1.796650	1.954339
H	-0.594909	1.801820	1.462729
H	0.598221	2.676910	0.497009
C	1.338351	-1.648990	0.395859
C	2.298891	0.943020	-1.712371
H	2.224121	2.033340	-1.693351
H	1.534291	0.540280	-2.375371
H	3.282761	0.656280	-2.087691
C	-1.383899	-0.346010	-0.404881
C	2.097631	0.401010	-0.314721
C	4.349121	0.843540	0.342789
H	4.868301	1.085160	1.269349
H	4.632471	1.565550	-0.428621
H	4.619421	-0.168570	0.027889
H	-1.427739	-0.547050	1.810269
H	-1.103059	-2.077660	0.962959
C	-2.623959	0.517820	-0.176521
O	-2.671919	1.721070	-0.264601
O	-3.661109	-0.250231	0.167499
C	-4.889709	0.439879	0.443609
H	-4.749329	1.132029	1.275349
H	-5.608249	-0.333881	0.702869
H	-5.214859	0.988989	-0.441231
C	-1.626079	-1.377470	-1.502701
H	-1.826729	-0.874930	-2.452251
H	-2.471829	-2.018630	-1.248501
H	-0.729459	-1.993720	-1.615311

Structure 3'_epi_preTS$_{MeBr}$

O	-0.238142	0.513021	0.517439
O	-3.369376	-0.190254	-0.439701
O	-3.460792	1.918396	0.470791
O	-2.830190	-1.411383	-2.241113
N	-1.411861	0.248223	-1.458976
C	-0.120073	-0.505089	-1.552530
C	-1.340129	1.034635	-0.220551
C	-1.064252	2.502678	-0.479061
H	-1.866398	2.955585	-1.061441
H	-0.121890	2.575871	-1.028363
H	-0.965377	3.035805	0.469772
C	-2.549311	-0.531829	-1.456377
C	-2.603278	0.067700	1.814143
H	-2.217074	0.784266	2.544074
H	-1.904156	-0.769533	1.752800
H	-3.581290	-0.294749	2.138243
C	0.171186	-0.692510	-0.108721
C	-2.721069	0.739624	0.461013
C	-4.752263	1.835486	1.061404
H	-5.265136	2.760365	0.797743
H	-4.686126	1.758987	2.151183
H	-5.312302	0.984052	0.662477
H	0.593053	0.142032	-2.083401
H	-0.262970	-1.429313	-2.111591
C	0.309868	-1.848542	0.625481
O	0.264621	-1.999314	1.872975
O	0.630958	-2.949302	-0.183722
C	0.779904	-4.178159	0.503149
H	0.982649	-4.927425	-0.264067
H	-0.129509	-4.447011	1.049247
H	1.612639	-4.144594	1.212291
C	2.902007	0.269581	0.011118
H	2.810869	-0.271583	0.947973
H	2.873970	-0.403862	-0.839255
H	2.149423	1.048527	-0.066102
Br	4.660585	1.134121	0.000386

Structure 3'_epi_TS$_{MeBr}$

O	0.296432	0.699831	-0.519691
O	3.191561	-0.544611	0.234869
O	3.722122	1.650939	-0.194151
O	2.490910	-1.788820	1.963939
N	1.435311	0.207800	1.429789
C	0.029801	-0.283599	1.558299
C	1.450522	1.063700	0.243269
C	1.336943	2.539450	0.570869
H	2.172943	2.872610	1.184369
H	0.396633	2.697771	1.105459
H	1.314423	3.120930	-0.354741
C	2.375841	-0.796820	1.280319
C	2.634371	0.310429	-1.927651
H	2.379002	1.214080	-2.488321
H	1.830031	-0.418750	-2.046421
H	3.567171	-0.105571	-2.314291
C	-0.354689	-0.390288	0.117779
C	2.784862	0.654609	-0.461191
C	5.020182	1.446578	-0.739351
H	5.668633	2.178557	-0.258171
H	5.026652	1.616108	-1.820521
H	5.385071	0.438847	-0.518261
H	-0.536579	0.475592	2.113049
H	0.011280	-1.226739	2.102949
C	-0.384030	-1.609258	-0.594891
O	-0.184390	-1.782629	-1.808051
O	-0.833201	-2.653248	0.190819

C	-1.016702	-3.889568	-0.483921
H	-1.386262	-4.587498	0.267569
H	-0.073642	-4.254419	-0.899651
H	-1.744342	-3.791827	-1.293801
C	-2.641089	0.279253	0.002779
H	-2.787129	-0.528337	0.703969
H	-2.537719	0.021083	-1.040511
H	-2.131248	1.167943	0.344859
Br	-4.671018	1.058755	-0.032451

Structure 6a_epi

O	-0.406531	-1.093060	0.851831
O	1.358260	1.208559	-0.443179
O	2.994690	-0.326722	0.060081
O	-0.018299	1.586110	-2.182059
N	0.222410	-0.536090	-1.273329
C	-1.150511	-1.001650	-1.389929
C	0.735659	-1.030641	-0.010669
C	1.353889	-2.412741	-0.098389
H	2.190819	-2.419901	-0.794869
H	0.590279	-3.121731	-0.428549
H	1.704429	-2.717871	0.890571
C	0.465600	0.828720	-1.376709
C	1.588110	0.556629	1.850861
H	1.897890	-0.250511	2.519981
H	0.548880	0.815239	2.050121
H	2.216231	1.432129	2.025341
C	-1.617411	-1.029370	0.098191
C	1.720940	0.101709	0.413851
C	4.057140	0.585958	0.322391
H	4.922410	0.200928	-0.215859
H	4.286280	0.626668	1.391341
H	3.814581	1.586158	-0.048229
H	-1.178631	-2.008200	-1.814339
H	-1.728600	-0.327110	-2.023789
C	-2.321750	0.297641	0.397451
O	-3.522800	0.443321	0.418001
O	-1.443660	1.286580	0.550081
C	-1.984589	2.610501	0.660991
H	-1.122869	3.270150	0.736871
H	-2.610339	2.683851	1.551611
H	-2.573209	2.844561	-0.227629
C	-2.514921	-2.207519	0.424131
H	-3.421751	-2.169709	-0.183289
H	-2.801491	-2.190189	1.477781
H	-1.978011	-3.135559	0.215231

6. X-Ray diffraction analysis

Details of the X-ray analyses are summarized in **Table S6**. Compound **2** was dissolved in dichloromethane and *n*-hexane was added carefully creating an interphase. The colourless crystal block was obtained by slow diffusion at 4 °C. Compound **10** was dissolved in dichloromethane and crystal needle was obtained at 4 °C. The formed crystals were analysed by X-ray diffraction.

The diffraction data were collected using graphite-monochromatic Mo-K_a radiation with a Bruker APEX-II diffractometer at a temperature of 298 K using the APEX3 software. The absorption correction was performed using MULTI-SCAN.[S1] The structures were solved with the WINGX program suite[S2] and refined by full-matrix least squares with SHELXL.[S3] Hydrogen atoms were located by mixed methods (electron-density maps and theoretical positions).

Mercury diagram of compounds **2** and **10** are represented in the **Figure S16**.

[S1] Blessing, R. H. *Acta Crystallogr.* **1995**, *A51*, 33.

[S2] Farrugia, L. J. *Appl. Crystallogr.* **2012**, *45*, 849.

[S3] Sheldrick, G. *Acta Crystallogr., Sect. C* **2015**, *71*, 3.

Compound **2** Compound **10**

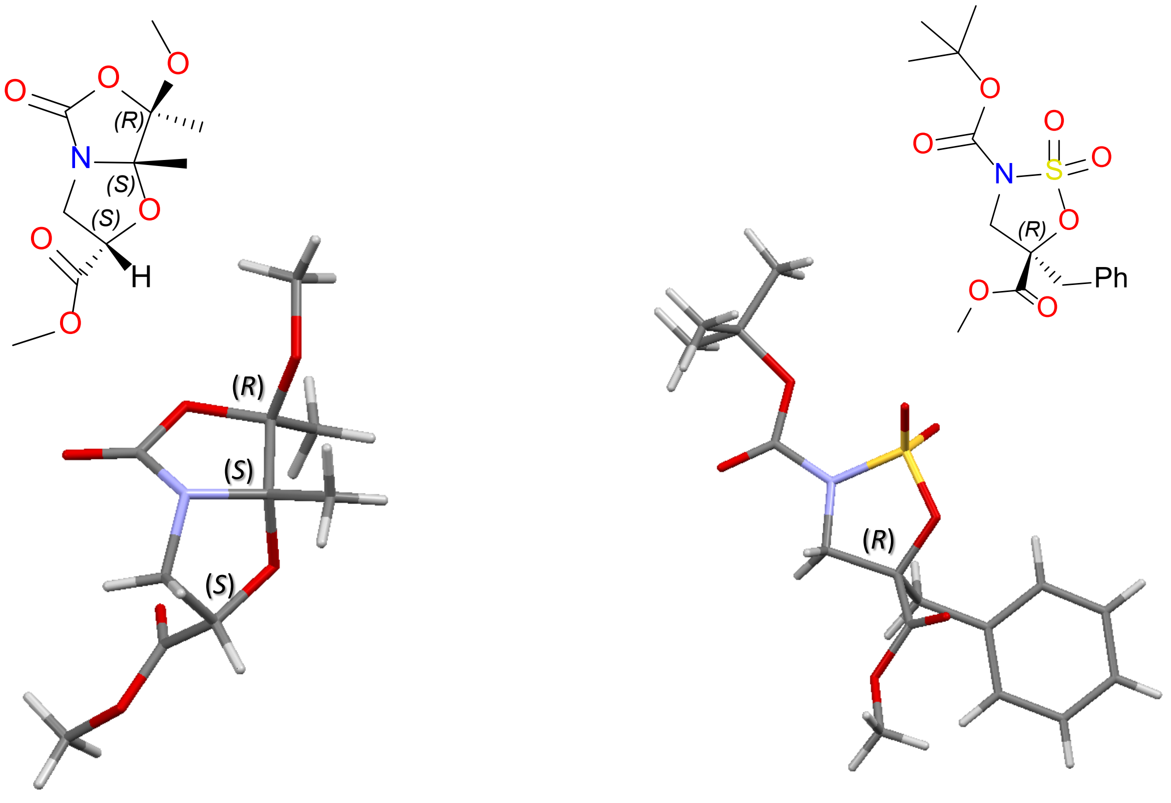

Figure S16. Mercury diagram of compounds **2** and **10** along with their 2D-ChemDraw structures.

Table S6. X-ray Crystallographic Data for **2** and **10**.

	**2**	**10**
**Empirical formula**	$C_{10} H_{15} N O_6$	$C_{16} H_{21} N O_7 S$
**F_w**	245.23	371.40
**T (K)**	298(2)	298(2)
**Wavelength (Å)**	0.71076	0.71076
**Crystal system**	Orthorhombic	Orthorhombic
**Space group**	P 21 21 21	P 21 21 21
**Crystal size (mm^3)**	0.2 x 0.1 x 0.07	0.27 x 0.19 x 0.1
**a (Å)**	9.145(5)	6.6726(2)
**b (Å)**	11.368(6)	12.7039(4)
**c (Å)**	11.977(7)	21.7090(7)
**α (°)**	90	90
**β (°)**	90	90
**γ (°)**	90	90
**V ($Å^3$)**	1245.2(12)	1840.23(10)
**Z**	4	4
**D_{calcd} (Mg/m3)**	1.308	1.341
**Absorption coefficient (mm^{-1})**	0.109	0.212
**F(000)**	520	784
**θ range for data collection (deg)**	2.859 to 28.106	3.240 to 27.928
**Index ranges**	-11<=h<=11, -15<=k<=14, -15<=l<=15	-8<=h<=8, -16<=k<=16, -28<=l<=28
**Reflections collected**	84726	88056
**Independent reflections**	2988 [R(int) = 0.0657]	4374 [R(int) = 0.0403]
**Data / restraints/ parameters**	2988 / 0 / 155	4374 / 0 / 226
**Goodness-of-fit on F^2 [a]**	1.149	1.054
**Final R index**	R_1 = 0.0594	R_1 = 0.0340
**[I > 2σ(I)][a]**	wR_2 = 0.1364	wR_2 = 0.1010
**R indexes (all data)[a]**	R_1 = 0.0742, wR_2 = 0.1439	R_1 = 0.0374, wR_2 = 0.1040
**Largest diff. peak and hole (e. $Å^{-3}$)**	0.194 and -0.141	0.234 and -0.202

[a] $R_1 = \sum(|F_o| - |F_c|)/\sum|F_o|$; $wR_2 = [\sum w(F_o^2 - F_c^2)^2/\sum wF_o^2]^{1/2}$; goodness of fit = $\{\sum[w(F_o^2 - F_c^2)^2]/(N_{obs} - N_{param})\}^{1/2}$;$w=[\sigma^2(F_o)+(g_1P)^2 + g_2P]^{-1}$; $P = [\max(F_o^2 ;0 + 2F_c^2)/3$.

7. Enantiomeric purity determination of 7c and 8c by NMR chiral shift reagents

Following a recent but slightly modified procedure,[S4,S5] the corresponding β-amino acid **7c**, **8c** or a mixture of known amounts of both was dissolved in D_2O to generate a 0.05 M solution. The pH of these three solutions was adjusted to 10 using 1 M KOH solution in D_2O. Then, a solution of 8 mg/mL of samarium(III) complex with (*S*,*S*)-ethylenediamine-*N*,*N′*-disuccinate in D_2O was prepared and 0.2 mL of this solution were added to each of the corresponding NMR tubes containing 0.5 mL of a solution of the amino acids **7c**, **8c** or the mixture of both. The ^1H NMR experiments were registered in a 400 MHz spectrometer.

[S4] Aizawa, S.-I.; Okano, M.; Kidani, T. *Chirality* **2017**, *29*, 273‑281.

[S5] Aizawa, S.-I.; Okano, M. *Magn. Reson. Chem.* **2020**, *58*, 941–948.

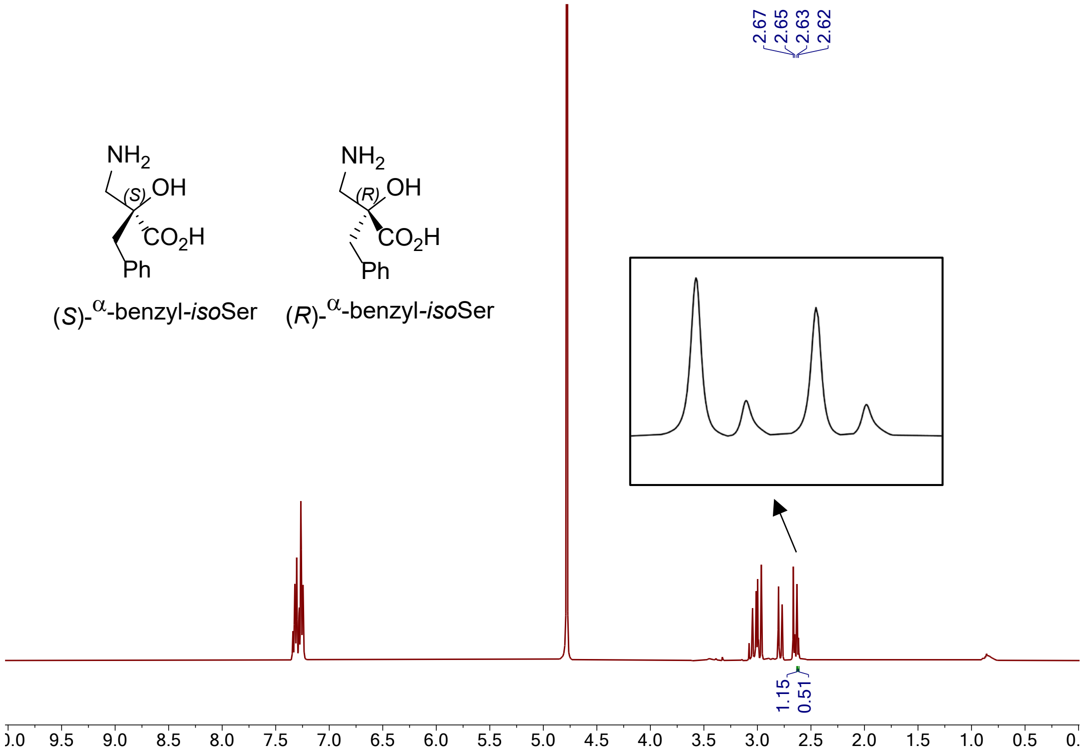

Figure S17. ^1H NMR 400 MHz in D_2O corresponding to a mixture of amino acids **8c** and **7c** (78:22) with samarium(III) complex.

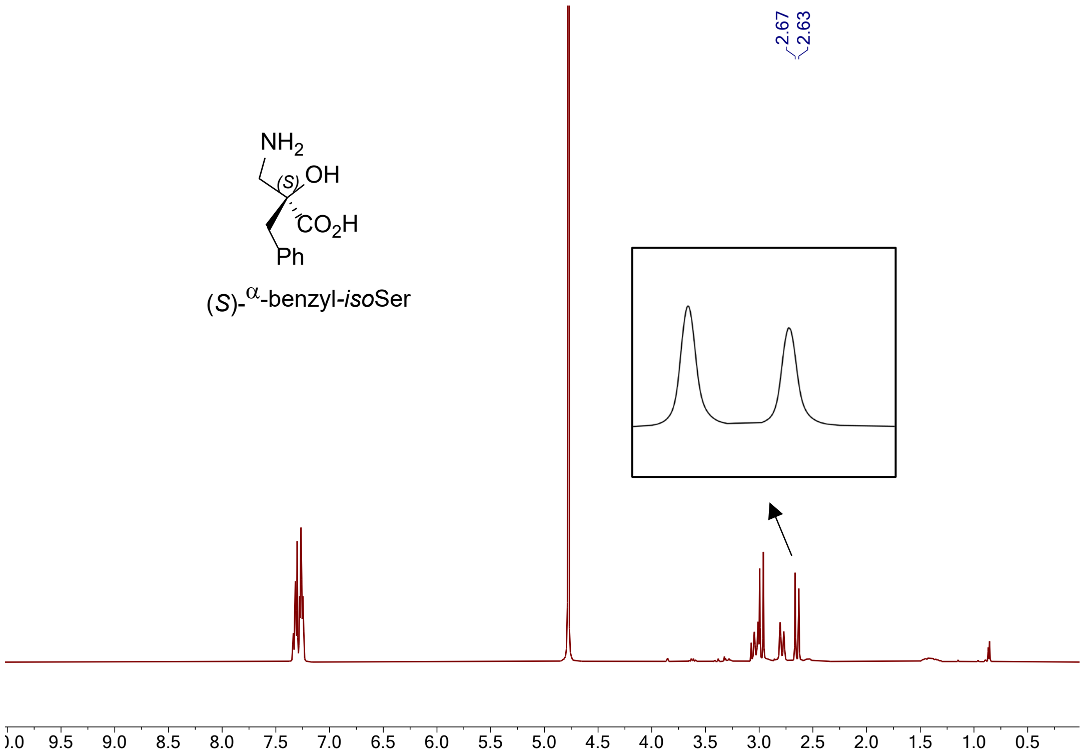

Figure S18. ^{1}H NMR 400 MHz in D_2O corresponding to amino acid **8c** with samarium(III) complex.

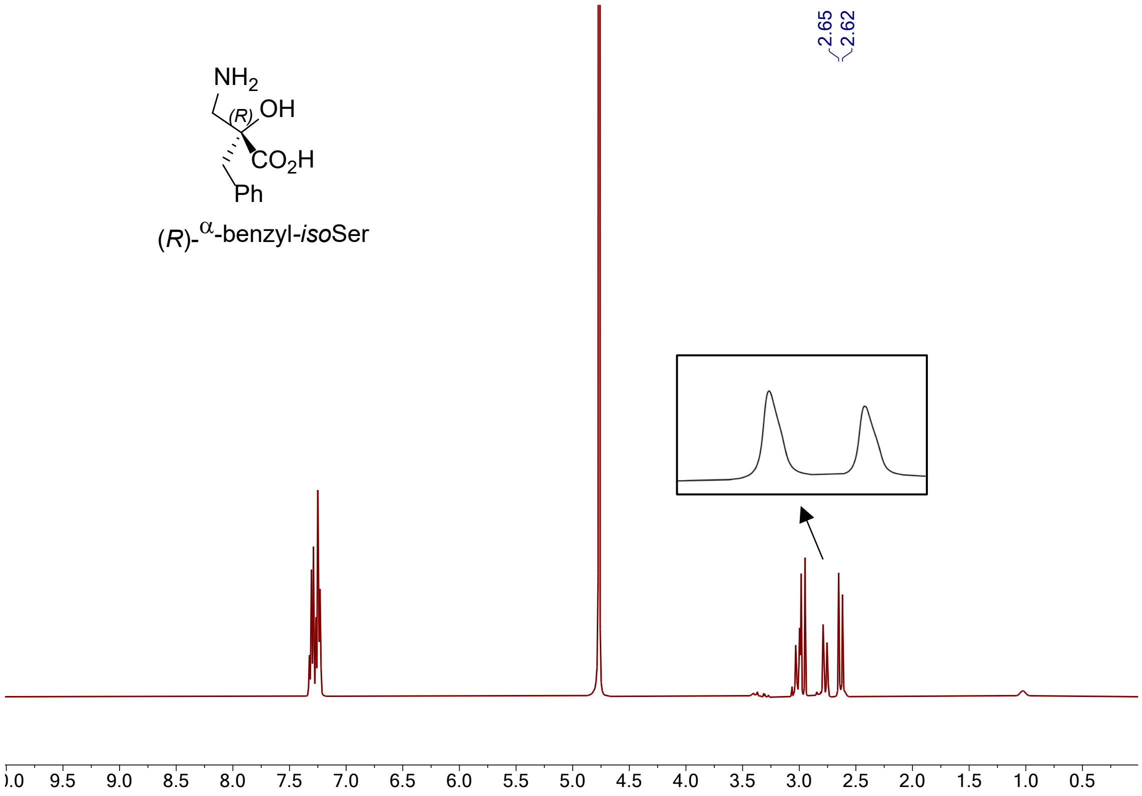

Figure S19. ^{1}H NMR 400 MHz in D_2O corresponding to amino acid **7c** with samarium(III) complex.

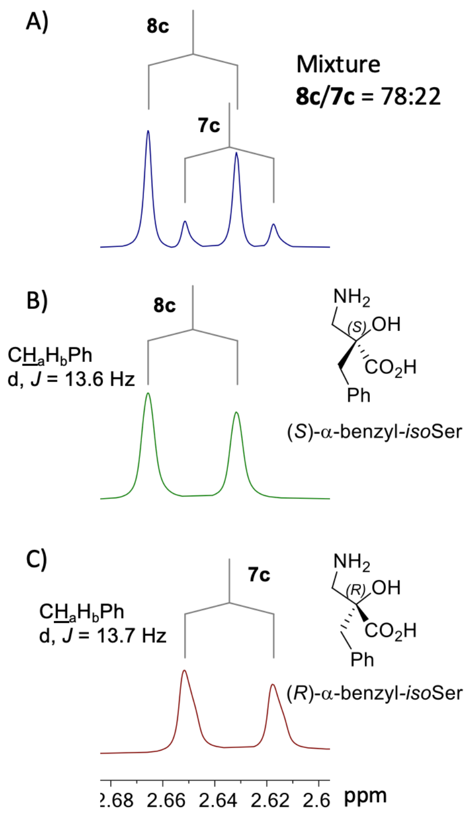

Figure S20. Aligned and expanded regions corresponding to C$\underline{H_a}$H$_b$Ph signal (doublet) extracted from the ^{1}H NMR (400 MHz) spectra in D_2O at 298 K of A) a mixture prepared by addition of amino acids (*R*)-α-benzyl-*iso*Ser **7c** and (*S*)-α-benzyl-*iso*Ser **8c** (enantiomers) in a 22:78 ratio, B) amino acid **8c** and C) its enantiomer: amino acid **7c**. All spectra were recorded in the presence of a chiral samarium(III) complex at pH 10 ([Sm(III) complex]/[amino acid] = 0.2).
